# Screening of the novel antimicrobial drug, XF-73, against 2,527 *Staphylococcus* species clinical isolates

**DOI:** 10.3389/fcimb.2023.1264456

**Published:** 2023-10-11

**Authors:** William Rhys-Williams, Helen Marie Galvin, William Guy Love

**Affiliations:** Destiny Pharma Plc, Brighton, United Kingdom

**Keywords:** exeporfinium chloride, XF-73, *Staphylococcus aureus*, MRSA, *Staphylococcus* species, MIC, antimicrobial

## Abstract

XF-73 (exeporfinium chloride) is a synthetic, di-cationic porphyrin derivative with rapid, potent bactericidal properties and a low propensity for engendering bacterial resistance. It is being developed clinically for the decolonization of *Staphylococcus aureus* in the nasal cavity to prevent post-operative staphylococcal infections. This study reports the minimum inhibitory concentration (MIC) of XF-73 in comparison to 22 antibiotics against a panel of >2,500 clinical isolates composed of 16 different Coagulase-positive and -negative *Staphylococcus* species from 33 countries. XF-73 was found to be effective against all isolates tested, with MICs ranging between ≤0.12 – 4 µg/ml (MIC_50_ and MIC_90_ values of 0.5 and 1 µg/ml respectively). XF-73 was found to be equally effective against antibiotic resistant isolates as antibiotic sensitive isolates, with no impact of pre-existing antibiotic resistance mechanisms to cell wall synthesis inhibitors (β-lactams, carbapenems, glycopeptides and cephalosporins), protein synthesis inhibitors (oxazolidinones, macrolides and tetracyclines), DNA synthesis inhibitors (fluoroquinolones) and a folate synthesis inhibitor. The panel selected also included examples of multidrug-resistant *S. aureus* isolates and, in all cases, the XF-73 MIC ranges were found to be similar against each of these groups. This dataset expands the knowledge of the breadth of activity of this novel antibacterial against a wide range of global *S. aureus* isolates and supports the potential utility of XF-73 for the treatment of patients who are *S. aureus* nasal carriers. Similar results were also obtained for multidrug-resistant isolates of other *Staphylococcus species* included in the study and collectively support the continued clinical development of XF-73 as an effective anti-staphylococcal drug.

## Introduction

Post-surgical site infections place a significant burden on health provision worldwide, and one of the major causative pathogens of such infections is *Staphylococcus aureus*. It is estimated that there are approximately 150,000 post-surgical infections in the US alone per year, representing a health burden and cost of $10 billion per year (Zabaglo and Sharman, 2022; Seidelman et al., 2023). *S. aureus* is the pathogen responsible for most surgical site infections (SSIs) with approximately 80% of *S. aureus* post-surgical infections auto infected from *S. aureus* carried within the patient’s nose (von Eiff et al., 2001; Wertheim et al., 2004). As a result, many current guidelines recommend the nasal decolonization of patients prior to surgery (Sway et al., 2018; Bratzler et al., 2013; Engelman et al., 2019; Ling et al., 2019; National Institute for Health and Care Excellence, 2019). A number of key surgical guidelines in the US, Europe, and other countries recommend preventative treatment of all *S. aureus* in patients undergoing high risk surgeries (Bratzler et al., 2013; Sway et al., 2018; Engelman et al., 2019; Ling et al., 2019; National Institute for Health and Care Excellence, 2019). It is recognized that, although screening for *S. aureus*, including methicillin-resistant *S. aureus* (MRSA) carriage is effective in identifying the majority of carriers, screening has a number of drawbacks, including cost, time, isolation while awaiting screening result and an inability to identify all carriers due to assay sensitivity and the impact of false positives. A study comparing the impact of universal decolonization (UD) versus targeted decolonization or active surveillance in a high risk patient population (ICU patients) demonstrated that UD decolonization was superior to the other arms, resulting in a 37% reduction in MRSA clinical isolates and a 44% reduction in all-cause bloodstream infections (Huang et al., 2013). As a result, the 2022 SHEA/IDSA/APIC Practice Recommendation update (Popovich et al., 2023) recommends the use of UD for all patients in adult ICUs and that UD should also be considered to be used in neonatal ICUs, burns units and hemodialysis patients.

At present, the most widely used intranasal decolonization agent is mupirocin, a bacteriostatic antibiotic that, due to its high potential to generate resistance, is not recommended for multiple administration (GlaxoSmithKline, 1987). The global prevalence of mupirocin resistance in *S. aureus* strains has risen to 7.6% and when looking at mupirocin resistant MRSA, the rate increases to 13.8%. (Dadashi et al., 2020) Although universal decolonization using mupirocin has in some studies been shown to be superior to targeted decolonization (Huang et al., 2013) there is an underlying concern that this approach may drive the more rapid development of mupirocin resistant staphylococcal strains to evolve and undermine the ability to deliver safe and effective infection prophylaxis and mupirocin resistance remains an unresolved issue in the 2022 SHEA/IDSA/APIC Practice Recommendation update (Popovich et al., 2023). There is an urgent need to identify and adopt the use of new antimicrobial agents, which can address resistance, thus allowing long-term and widespread effective decolonization.

XF-73 is a novel, synthetic di-cationic porphyrin derivative with rapid, potent Gram positive bactericidal properties which is being developed clinically as a gel for the nasal decolonization of *S. aureus* to prevent post-operative staphylococcal infections. XF-73 has been awarded Qualifying Infectious Disease Product status (Link) and Fast Track designation (Link) by the US Food and Drug Administration, identifying it as a product that is seeking to address a priority infectious disease. A recent Phase 2 study has been completed (Mangino et al., 2023) investigating the safety and microbiological efficacy of XF-73 in reducing *S. aureus* nasal carriage prior to open chest cardiac surgery, which demonstrated that patients administered intranasal XF-73 gel within 24 hours of cardiac surgery had significantly reduced nasal burden of *S. aureus* one hour before surgery compared to a placebo control.

The antimicrobial properties of XF-73 have been investigated and found to have broad spectrum activity against all Gram positive bacteria tested to date, including methicillin-sensitive *S. aureus* (MSSA) and antibiotic resistant strains such as MRSA and mupirocin-resistant *S. aureus* isolates (Farrell et al., 2010). XF-73 has also been demonstrated to be active against cultures of *S. aureus* in stationary phase, non-growing cultures expressing the stringent response and in cold cultures, demonstrating that antimicrobial activity is not dependent on metabolic or growth activity (Ooi et al., 2010). XF-73 was found to have a low propensity to generate resistance, with no mutational resistance observed after 55 serial passages at sub-inhibitory concentrations (Farrell et al., 2011; MacLean, 2020) whilst mupirocin, which was also included in the same study, rapidly generated mutational resistance in all four MRSA isolates tested, with MICs up to 512 µg/ml generated. Importantly, there was no evidence of cross-resistance with XF-73 in these mupirocin resistant isolates generated (Farrell et al., 2011). Furthermore, the antimicrobial activity of XF-73 has been demonstrated to be independent of all known existing antimicrobial resistance mechanisms (Farrell et al., 2010). The presence of existing cell wall, DNA and protein synthesis resistance mechanisms were not found to affect the susceptibility of antibiotic-resistant bacteria to XF-73 compared to antibiotic-sensitive isolates, suggesting that the mechanism of action (MOA) of XF-73 is novel. Investigations into the MOA of XF-73 has demonstrated that XF-73 exerts a rapid bacterial membrane-perturbing activity resulting in substantial loss of potassium and adenosine triphosphate from the cells, complete inhibition of DNA, RNA and protein synthesis but without inducing bacterial lysis (Ooi et al., 2009).

Here, we report the antimicrobial susceptibility of over 2,500 Coagulase-positive and -negative *Staphylococcus* species clinical isolates to XF-73. The majority of isolates tested were *S. aureus* but the panel also included examples of 15 other *Staphylococcus* species, including ones that have been previously reported to be isolated from the nose and examples of pathogens responsible for infections including endocarditis and bacteremia. The *Staphylococcus* species panel was constructed to included antibiotic resistant and sensitive isolates to further investigate the effect of existing antibiotic resistance mechanisms on the activity of XF-73.

## Materials and methods

### Microorganisms

2,527 clinical staphylococcal isolates, made up of 16 different *Staphylococcus* species, collected between 2010 and 2012 and provided by Oppilotech Ltd. (London, United Kingdom) were tested for their susceptibility to XF-73. The species selected included *S. epidermidis, S. saprophyticus, S. haemolyticus, S. capitis, S. hominis*, and *S. lugdunensis* which are believed to be associated with a high clinical significance (Michels et al., 2021). The screening panel were obtained from patients aged between 0 and 97 years old and located in 33 countries across Europe, North America, South America, Asia, Africa and Oceania (**Figure S1**). The isolates were obtained from a range of clinical indications including skin and soft tissue infections, respiratory tract infections, hospital-acquired pneumonia and complicated skin and soft tissue infections. Further information is provided in the **Supplementary Material**. Isolates were carefully selected to include antibiotic sensitive and resistant clinical isolates, including MRSA; MSSA; antibiotic-susceptible and resistant *Staphylococcus epidermidis* and examples of staphylococcal clinical isolates resistant to cell wall synthesis inhibitors (β-lactams, carbapenems, glycopeptides and cephalosporins), protein synthesis inhibitors (oxazolidinones, macrolides and tetracyclines), DNA synthesis inhibitors (fluoroquinolones) and folate synthesis inhibitors (antifolate) in the panel. *Staphylococcus* species previously reported to be present in the nose were also included in the panel, including *S. capitis, S. haemolyticus, S. hominis* and *S. lugdunensis* (Lina et al., 2003; McMurray et al., 2016). *S. aureus* and *S. intermedius* were Coagulase-positive *Staphylococcus* species whist the remainder were Coagulase-negative. Historic comparator MIC data against 21 antibiotics was provided by Oppilotech Ltd (London, United Kingdom) and contemporaneous MIC determination of a control antibiotic (dalbavancin) was included in this study and compared to historic data to confirm similar results.

### Antimicrobial drugs

#### XF drugs

XF-73 (exeporfinium chloride; C_44_H_50_Cl_2_N_6_O_2_) was provided by Destiny Pharma plc and resuspended in distilled water to generate stock concentrations of 10 µg/ml which was used to undertake serial dilutions in growth media to the required concentration ranges.

#### Antibiotics

Dalbavancin was prepared as stock solutions by standard methodologies.

### Susceptibility of bacteria to XF-73 using broth microdilution

The MIC of XF-73 was determined against test bacteria using a broth microdilution method. (Clinical and Laboratory Standards Institute (CLSI) – Methods for Dilution Antimicrobial Susceptibility Tests for Bacteria That Grow Aerobically: Approved Standard – Tenth Edition). Briefly, 100 µl of XF-73 at a range of concentrations (0 – 16 µg/ml) in Mueller Hinton Broth (MHB) was added to the wells of a 96-well microtiter plate. Overnight bacterial cultures in MHB were adjusted to a 0.5 McFarland standard and diluted 10-fold. Five ml of these cultures were added to each drug concentration to generate an inoculum ca. 5x10^5^ colony forming units (CFU)/ml, per well. All plates were subsequently incubated aerobically at 37°C for 16-20 h and visually analyzed for bacterial growth. The MIC was defined as the lowest concentration that completely inhibited visible growth as detected by unaided eye.

### Data analysis

Unpaired t test followed by Mann-Whitney test was performed on the differences in the XF-73 MIC distribution between MRSA and MSSA using GraphPad Prism version 10.0.1 for Windows, GraphPad Software, Boston, Massachusetts USA, www.graphpad.com.

## Results

### XF-73 antimicrobial activity

**Figure 1:**
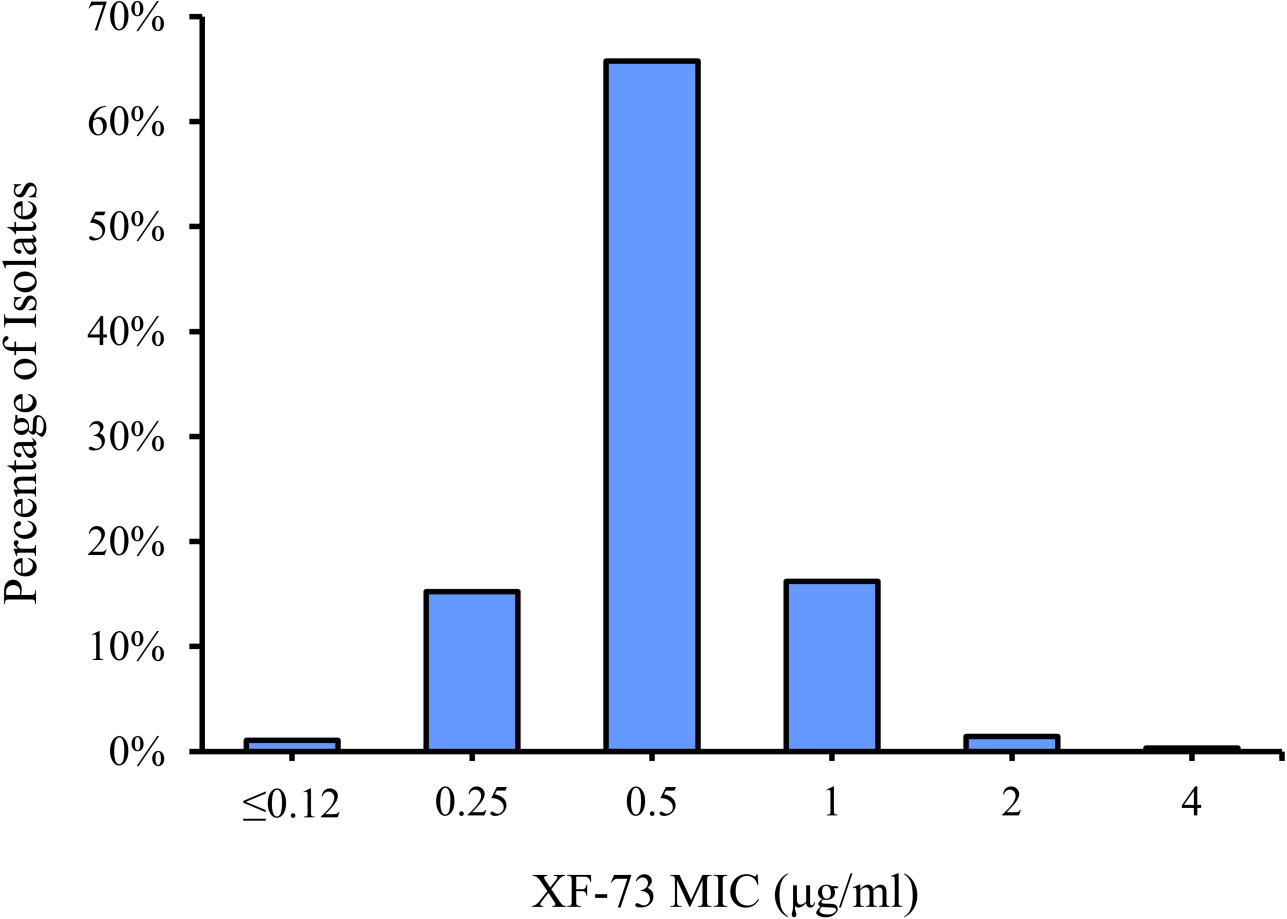
XF-73 MIC distribution for all 2,527 *Staphylococcus* isolates tested.

**Figure 2:**
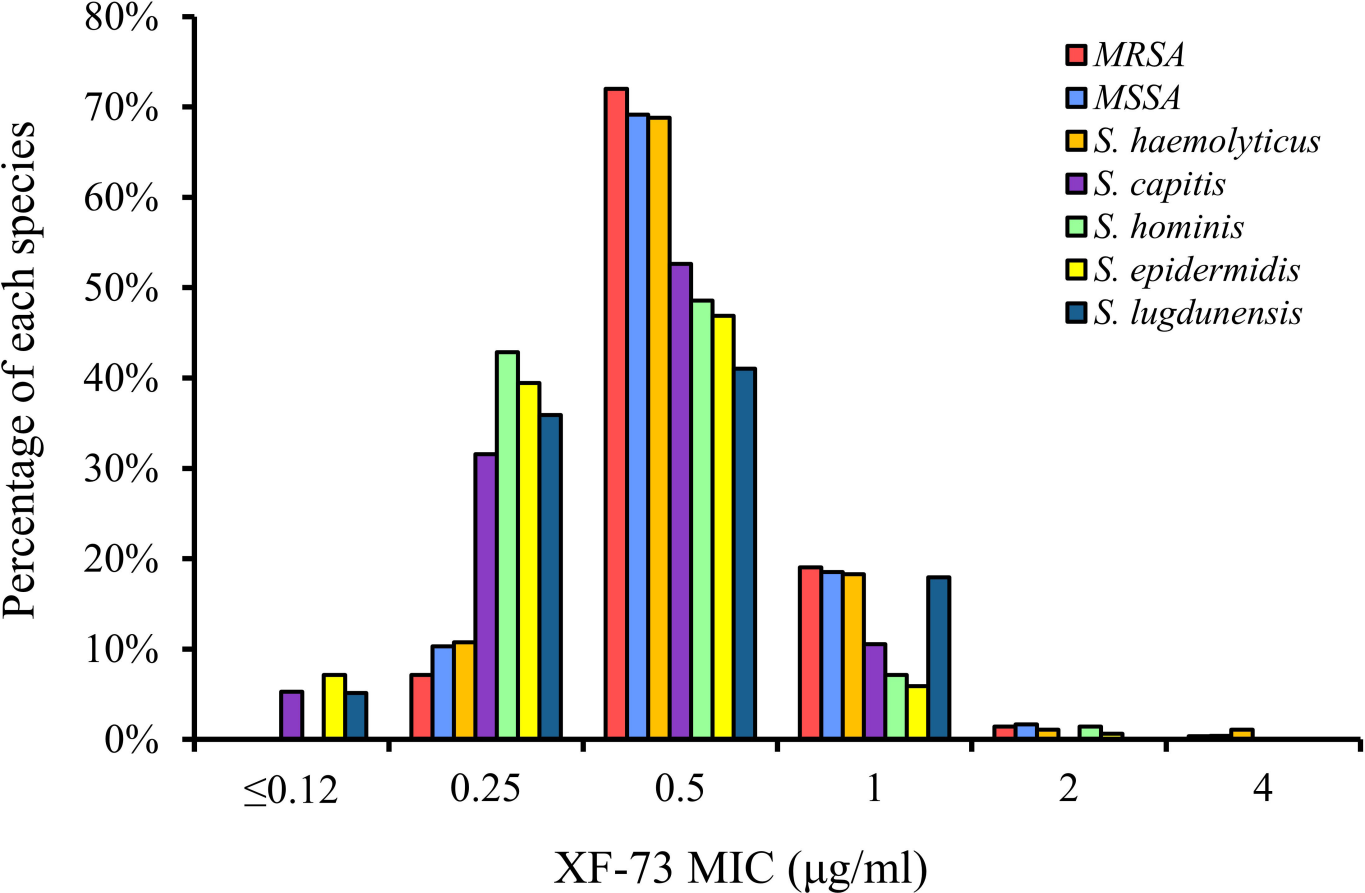
XF-73 MIC distribution for *Staphylococcus* species where >30 isolates per species tested.

The distribution of the MICs for all of the *Staphylococcus* species tested are shown in **Figure 1** and the XF-73 MIC ranges for the 2,527 clinical isolates consisting of 16 *Staphylococcus* species tested is presented in **Table 1**. Within the bacterial panel 1,919 clinical isolates of *S. aureus* were tested and the MIC range for XF-73 was found to be 0.25 - 4 µg/ml (MIC_50_ and MIC_90_ values were 0.5 and 1µg/ml respectively). Within this *S. aureus* panel, there were 1,079 MSSA and 840 MRSA isolates and both MSSA and MRSA had the same MIC range of 0.25 - 4 µg/ml and the same MIC_50_ and MIC_90_ values of 0.5 and 1 µg/ml respectively. There was no difference in the XF-73 MIC distribution between the MRSA and MSSA isolates (Mann-Whitney test; p= 0.21 and median values both 0.5 µg/ml). The panel also contained 322 *S. epidermidis* isolates and the MIC_50_ and MIC_90_ values were 0.5 and 0.5µg/ml respectively. The distribution of the MICs determined for the *Staphylococcus* species where >30 different isolates were screened is provided in **Figure 2**.

**Table 1:** XF-73 MIC ranges for the *Staphylococcus* species tested.

Staphylococcus species	Number of isolates	XF-73 MIC range (µg/ml)	MIC_50/90_ (µg/ml)	Dalbavancin MIC range (µg/ml)
All *Staphylococcus* species	2,527	≤0.12-4	0.5/1	≤0.06->0.5
*Staphylococcus aureus* **^$^** ^¥^	1,919	0.25-4	0.5/1	≤0.12->0.5
MRSA**^$^** ^¥^	840	0.25-4	0.5/1	≤0.12->0.5
MSSA**^$^** ^¥^	1,079	0.25-4	0.5/1	≤0.12->0.5
*Staphylococcus capitis* **^$^**	38	≤0.12-1	0.5/1	≤0.06->0.5
*Staphylococcus caprae*	7	0.25-2	ND*	≤0.06->0.25
*Staphylococcus cohnii* **^$^**	1	0.25	ND*	0.12
*Staphylococcus condimenti*	1	0.25	ND*	0.12
*Staphylococcus epidermidis* **^$^**	322	≤0.12-2	0.5/0.5	≤0.12-0.5
*Staphylococcus haemolyticus* **^$^**	93	0.25-4	0.5/1	≤0.06->0.5
*Staphylococcus hominis* **^$^**	70	0.25-2	0.5/0.5	≤0.06->0.5
*Staphylococcus intermedius* ^¥^	1	0.25	ND*	≤0.06
*Staphylococcus lugdunensis* **^$^**	39	≤0.12-1	0.5/1	≤0.06->0.25
*Staphylococcus pasteuri* **^$^**	3	0.5-1	ND*	≤0.06
*Staphylococcus pettenkoferi* **^$^**	11	0.25-0.5	ND*	≤0.06-0.12
*Staphylococcus saprophyticus* **^$^**	4	0.25-0.5	ND*	≤0.06-0.12
*Staphylococcus sciuri*	1	0.25	ND*	0.25
*Staphylococcus simulans* **^$^**	6	0.25-0.5	ND*	≤0.06-0.12
*Staphylococcus warneri* **^$^**	11	0.25-2	ND*	≤0.06-0.25

ND*, Not determined as number of isolates too low to obtain a MIC_50/90_ value; **^$^** *Staphylococcus* species associated with the nose (Lina et al., 2003; McMurray et al., 2016); ^¥^Coagulase-positive - all others are Coagulase-negative.

The MICs of the *Staphylococcus* species which have previously been isolated from the nose are identified within **Table 1**. The geographical location (**Table S3**), the infection type/location from which the *Staphylococcus* species were isolated from (**Tables S5**, **S6**), or the age of the patient (**Table S4**) did not impact the effectiveness of XF-73 (**Supplementary data**).

Of all the staphylococcal isolates tested, a small number (n=27; 1.1% of the total screened) had an MIC for XF-73 of ≤0.12 µg/ml. No *S. aureus* isolates were part of this group. This group was comprised of *S. epidermidis, S. capitis* and *S. lugdunensis* species, all of which are Coagulase-negative and have previously been isolated from the nose (Lina et al., 2003; McMurray et al., 2016) and are associated with biofilm production (Yong et al., 2019). Of the isolates that were most susceptible to XF-73, 91% were resistant to β-lactam (penicillin) a cell wall synthesis inhibitor, 83% were resistant to the fluoroquinolone levofloxacin, a DNA synthase inhibitor, 78% were resistant to the macrolide clarithromycin, a protein synthesis inhibitor and 56% were resistant to the antifolate trimethoprim/sulfmethoxazole, a folate synthesis inhibitor. Of all the staphylococcal isolates tested, a very small number (n=8; 0.3%) had an MIC of 4µg/ml. This is within 3 dilution concentrations of the MIC_90_ and therefore is within acceptable MIC QC ranges associated with the CLSI broth microdilution MIC assay procedure (CLSI, 2020).

### Impact of the presence of multidrug-resistance mechanisms on the activity of XF-73

The panel of *Staphylococcus* species tested included isolates that were sensitive and resistant to 22 different antibiotics (**Supplementary Table S1**). Of these, the antibiotic resistance profile for 10 antibiotics were common to those previously reported for the *Staphylococcus* species tested by Farrell et al. (2010). The presence of all pre-existing resistance mechanisms was found to not impact the antimicrobial activity of XF-73 (**Supplementary Table S2**).

MRSA is defined as MDR following a joint initiative by the European Centre for Disease Prevention and Control (ECDC) and the Centers for Disease Control and Prevention (CDC), to create a standardized international terminology with which to describe acquired resistance profiles in *S. aureus* (Magiorakos et al., 2012) and in the panel tested, both MRSA and MSSA had the same MIC range of 0.25 - 4 µg/ml, the same MIC_50_ and MIC_90_ values of 0.5 and 1 µg/ml respectively. The presence of additional antibiotic resistance within the MRSA isolates also had no impact on the XF-73 MIC range (**Supplementary Table S2**). The Global Antibiotic Research & Development Partnership (GARD-P) define MDR as a lack of susceptibility to at least one agent in three or more chemical classes of antibiotic (e.g. a β-lactam, cephalosporin and a macrolide) (Link). Applying this definition to the other *Staphylococcus* species tested, 6% of the MSSA isolates tested were MDR (65/1079 isolates) and the XF-73 MIC range was 0.25 - 2µg/ml, the MIC_50_ and MIC_90_ values were 0.5 and 1 µg/ml for MDR isolates and 0.25 - 4 µg/ml, 0.5 and 1 µg/ml respectively for non-MDR isolates. For *S. epidermidis*, 43% of the isolates tested were MDR (140/322 isolates) and the XF-73 MIC range was ≤0.12 - 2 µg/ml, the MIC_50_ and MIC_90_ values were 0.25 and 0.5 µg/ml for MDR isolates and ≤0.12 - 2 µg/ml, 0.5 and 0.5 µg/ml respectively for non-MDR isolates. For *S. haemolyticus*, 54% of the isolates tested were MDR (50/93 isolates) and the XF-73 MIC range was 0.25 – 2 µg/ml, the MIC_50_ and MIC_90_ values were 0.5 and 1 µg/ml for MDR isolates and 0.25 – 4 µg/ml, 0.5 and 1 µg/ml respectively for non-MDR isolates. Although 24% of the *S. hominis* isolates tested were MDR (17/70) there were insufficient numbers of isolates to determine MIC_50_ and MIC_90_ values for XF-73, but the MIC ranges for MDR and non-MDR isolates were similar (0.25 - 2 µg/ml and 0.25 - 1µg/ml respectively) (**Supplementary Table S2**).

## Discussion

SSIs are among the most common healthcare-associated infections and are associated with longer post-operative hospital stays, additional surgical procedures, treatment in intensive care units and higher mortality. *Staphylococcus* species are responsible for a substantial percentage of SSIs and therefore are a major focus in the prevention of SSIs. A recent European Centre for Disease Prevention and Control Report from 2023 identified that *S. aureus* and Coagulase-negative staphylococci were reported in 29.2% of all SSIs in a survey of 7,816 SSIs from 11 countries (ECDC, 2023).

The results reported in this study demonstrate that XF-73 has similar antimicrobial activity against all of the staphylococcal species tested, irrespective of the existing antibiotic resistance profile. A screen of 3,929 clinical isolates of MRSA and MSSA from 12 different countries against linezolid, tigecycline and vancomycin reported MIC_50_ and MIC_90_ values (Karlowsky et al., 2017) that were either identical, or within one MIC dilution factor, to those reported in this study. Similarly, another screen against 756 MRSA and MSSA clinical isolates obtained from across Europe and Russia against the same three antibiotics (Riccobono et al., 2022) also reported similar MIC_50_ and MIC_90_ ranges. The manuscript by Riccobono et al. (2022) also report MIC_50_ and MIC_90_ values for the same three antibiotics against 218 Coagulase negative *Staphylococcus* species isolates, which are also either identical, or within one MIC dilution factor, to those reported in this study. The comparator antibiotic MIC_50_ and MIC_90_ results in this study are therefore similar to other results reported in the literature.

The data presented here expands significantly on the antimicrobial activity of XF-73 against staphylococcal isolates previously reported (Farrell et al., 2010). In total, over 2,500 staphylococcal clinical isolates were tested within this study consisting of 16 different *Staphylococcus* species from 33 different countries and isolated from 5 different clinical presentations. The MIC range for XF-73 against all of the *Staphylococcus* species was within a narrow range (≤0.12 – 4 µg/ml) with an MIC_50_ and MIC_90_ (where determined) of 0.5 and 1 µg/ml respectively, demonstrating that XF-73 has potent antimicrobial activities against all 16 *Staphylococcus* species tested.

The *Staphylococcus* species isolates were carefully selected to include antibiotic sensitive and resistant isolates, including MRSA; MSSA; antibiotic-susceptible and resistant *Staphylococcus epidermidis* and examples of *Staphylococcus* species clinical isolates resistant to cell wall synthesis inhibitors (β-lactams, carbapenems, glycopeptides and cephalosporins), protein synthesis inhibitors (oxazolidinones, macrolides and tetracyclines) and DNA synthesis inhibitors (fluoroquinolones) and folate synthesis inhibitors (antifolate). The results demonstrate that all pre-existing antibiotic resistance mechanisms were not found to impact the antimicrobial activity of XF-73. The inclusion of MDR *S. aureus* isolates in the test panel confirmed that XF-73 has equivalent activity even against these highly antibiotic resistant isolates, which demonstrates that nasal decolonization using XF-73 is likely to be equally effective even when such *S. aureus* isolates are present in the nares. Such an attribute could disrupt the spread of such resistant isolates, as well as reducing the incidence of nasal decolonization treatment failures.

The broad anti-staphylococcal activity of XF-73, which includes 12 *Staphylococcus* species that have been reported to be isolated from the nose suggests that in addition to the ability of XF-73 to significantly reducing the nasal burden of *S. aureus* which has been demonstrated in clinical studies, including those in patients (Mangino et al., 2023), the application of XF-73 to the anterior nares is also likely to significantly reduce the nasal burden of all *Staphylococcus* species that may be present, providing additional protection against surgical infections caused by other *Staphylococcus* species. The mutational resistance profile for XF-73 has been previously demonstrated to be superior to that of mupirocin in *in vitro* multi-passage studies (Farrell et al., 2011), and a recent study has demonstrated that XF-73 has equal *in vitro* and *in vivo* antimicrobial activity against *S. aureus* isolates that are mupirocin-sensitive, low-level (MIC 16 µg/ml) and high-level (4,096 µg/ml) mupirocin resistant (Zhang et al., 2023), therefore XF-73 is a potential new candidate for nasal decolonization and the clinical trials undertaken to date have demonstrated an appropriate safety profile and rapid reduction in *S. aureus* nasal load (Yendewa et al., 2020; Mangino et al., 2023).

The attributes of XF-73 also suggest that it could have utility for the treatment of a range of *S. aureus* infections, and XF-73 is also currently being developed for the treatment of superficial skin infections and for the prevention and treatment of serious infections associated with burns and open wounds such as diabetic foot ulcers.

These findings support the continued clinical development of XF-73 as an effective anti-staphylococcal drug.

## Data availability statement

The original contributions presented in the study are included in the article/**Supplementary Material**. Further inquiries can be directed to the corresponding author.

## Author contributions

WR: Conceptualization, Project administration, Supervision, Writing – original draft, Writing – review & editing. HG: Formal Analysis, Writing – review & editing. WL: Conceptualization, Funding acquisition, Writing – review & editing.
