# Supplementary material for: Screening of the novel antimicrobial drug, XF-73, against 2,527 Staphylococcus species clinical isolates
Source: Front Cell Infect Microbiol. 2023 Oct 11;13:1264456. doi: 10.3389/fcimb.2023.1264456 (PMC10600368; doi:10.3389/fcimb.2023.1264456)
Supplement: Supplementary file 1 [file DataSheet_1.pdf]

SUPPLEMENTARY INFORMATION

Figure s1: Distribution of isolates tested by country

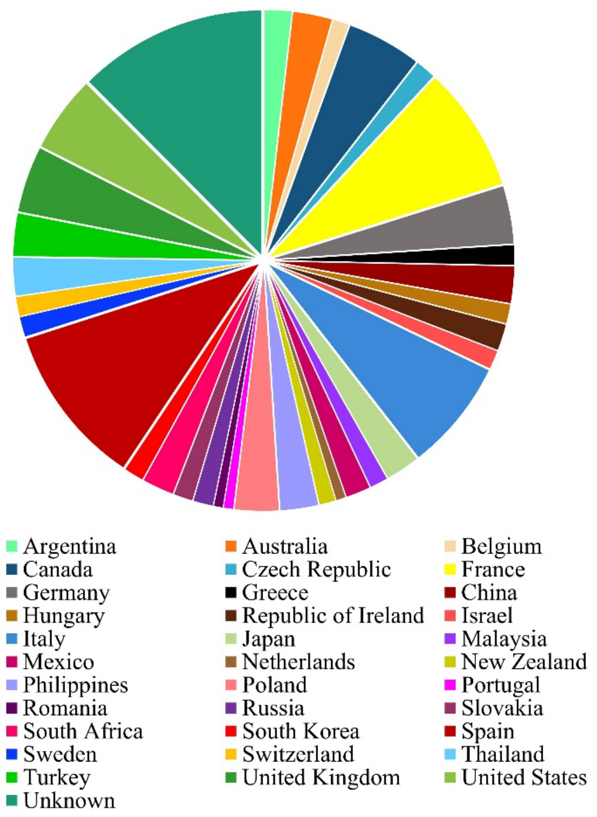

Figure s2: *Staphylococcal* species distribution

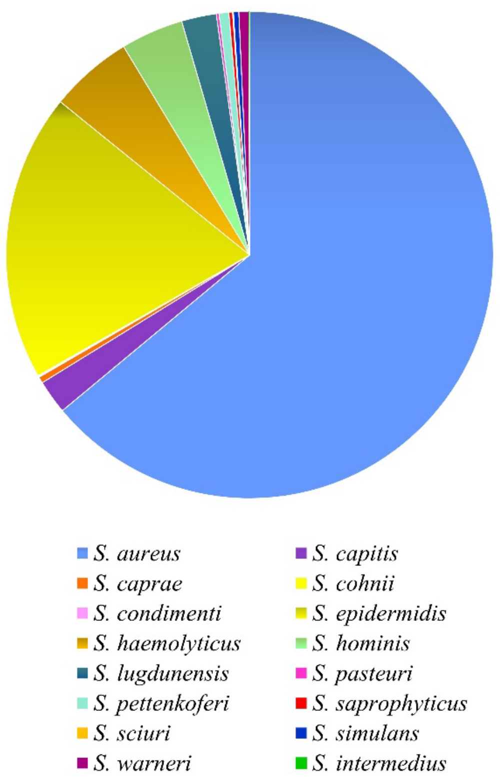

Table s1: Antibiotic Susceptibility Profile for the *Staphylococcal* species tested (where n>30 isolates present)

PEN – Penicillin; OXA- Oxacillin; AMC- Amoxicillin Clavulanate; PTZ – Piperacillin/Tazobactam; MEM- Meropenem; VAN- Vancomycin; DAL-Dalbavancin; TEI- Teicoplanin; ROX- Cefuroxime; AXO- Ceftriaxone; TAZ- Ceftadizime; LEV-Levofloxacin; CIP- Ciprofloxacin; MOX- Moxifloxacin; LZD- Linezolid; CLA- Clarithromycin; DA- Clindamycin; ERY- Erythromycin; TET- Tetracycline; TIG- Tigecycline; GEN- Gentamycin; SXT- Trimethoprim/Sulfamethoxazole; MOA = Mechanism of action; * = CLSI disk diffusion MIC breakpoints taken from "Performance Standards for Antimicrobial Susceptibility Testing, M100 32nd Edition". Clinical and Laboratory Standards Institute, Wayne, PA (2022); **= Disk diffusion MIC breakpoints for these staphylococcal species not included in the 2022 CLSI document and therefore the last noted breakpoint from "Performance Standards for Antimicrobial Susceptibility Testing, M100 22nd Edition". Clinical and Laboratory Standards Institute, Wayne, PA) used. No CLSI breakpoints are available for dalbavancin and tigecycline; None tested = antibiotic susceptibility not determined

**Antibiotic Class**	**Antibiotic**	***Antibiotic MOA***	***Staphylococcal species***							
			S. aureus	***MSSA***	***MRSA***	***S. epidermidis***	***S. haemolyticus***	***S. hominis***	***S. lugdunensis***	***S. capitis***
			**Percentage of Isolates that are resistant**							
Beta lactams	Penicillin*	Cell Wall Synthesis Inhibitor	87.6%	79.7%	99.5%	88.8%	85.9%	78.6%	38.5%	73.7%
	Oxacillin*	Cell Wall Synthesis Inhibitor	53.9%	0%	100%	None tested	100%	None tested	None tested	None tested
	Amoxicillin Clavulanate**	Cell Wall Synthesis Inhibitor	68.3% / 62.4 %	7.7%/ 6.2%	91.0%/ 79.4%	15.8% / 20.5%	59.8%/ 54.4%	7.1%/ 2.9%	2.6%/ 0%	31.6%/ 15.8%
	Piperacillin/ Tazobactam**	Cell Wall Synthesis Inhibitor	26.4 / 6.3%	9.9%/ 7.2%	96.4%/ 79.1%	17.7% / 33.9%	69.6%/ 65.2%	37.1%/ 5.7%	2.6%/ 0%	44.7%/ 34.2%

Carbapenems	Meropenem**	Cell Wall Synthesis Inhibitor	14.1%	1.8%	32.6%	22.7%	50%	7.1%	2.6%	18.4%
Glycopeptides	Vancomycin*	Cell Wall Synthesis Inhibitor	0%	0%	0%	0%	0%	0%	0%	0%
	Dalbavancin*	Cell Wall Synthesis Inhibitor	*No CLSI breakpoints for resistance in staphylococcal species'*							
	Teicoplanin**	Cell Wall Synthesis Inhibitor	0%	0%	0%	0%	1.1%	0%	0%	0%
Cephalosporins	Cefuroxime**	Cell Wall Synthesis Inhibitor	34.2%	5.7%	76.2%	23.3%	57.6%	4.3%	2.6%	29%
	Ceftriaxone**	Cell Wall Synthesis Inhibitor	0%	0%	0%	0%	0%,	0%	0%	0%
	Ceftazidime**	Cell Wall Synthesis Inhibitor	42.8%	8.7%	93.8%	50%	72.8%	47.1%	2.6%	42.1%
Flouroquinolones	Levofloxacin*	DNA Synthesis Inhibitor	35.5%	11.4%	71.5%	54.4%	58.7%	31.4%	2.6%	15.8%
	Ciprofloxacin*	DNA Synthesis Inhibitor	52.3%	7.2%	88.4%	None tested	0%	None tested	None tested	None tested
	Moxifloxacin*	DNA Synthesis Inhibitor	30.8%	9.9%	62.2%	33.2%	38%	28.6%	0%	18.4%
Oxazolidinones	Linezolid*	Protein Synthesis Inhibitor	0.6%	0.7%	0.1%	0.3%	1.1%	0%	0%	0%
Macrolides	Clarithromycin*	Protein Synthesis Inhibitor	42.6%	25.2%	68.6%	62.4%	87%	70%	15.4%	36.8%
	Clindamycin*	Protein Synthesis Inhibitor	22.3%	None tested	38.4%	None tested	0%	None tested	None tested	None tested

	Erythromycin*	Protein Synthesis Inhibitor	46.1%	19.5%	68.8%	None tested	0%	None tested	None tested	None tested
Tetracyclines	Tetracycline*	Protein Synthesis Inhibitor	14%	7.2%	24.1%	10.9%	22.8%	35.7%	5.1%	7.9%
	Tigecycline*	Protein Synthesis Inhibitor	*No CLSI breakpoints for resistance in staphylococcal species'*							
Aminoglycosides	Gentamycin*	Protein Synthesis Inhibitor	11.3%	3.8%	17.8%	None tested	0%	None tested	None tested	None tested
Antifolate	Trimethoprim/ Sulfmethoxazole*	Folate Synthesis Inhibitor	3.9%/ 0%	1.7%/ 0%	6.8%/ 0%	41.3%/ 0%	38.7%/0%	47.1%/0%	2.6%/0%	5.3%/0%

Table s3: MIC of XF-73 against all *Staphylococcal* species by country of origin

ND* = Not determined as number of isolates too low to get a reliable $MIC_{50/90}$ value

Country of origin	No of isolates	MIC_{50} (µg/ml)	MIC_{90} (µg/ml)	MIC range (µg/ml)
Argentina	46	0.5	1	0.25-4
Australia	66	0.5	1	≤0.12- 1
Belgium	28	ND*	ND*	0.25-1
Canada	125	0.5	1	≤0.12- 4
China	62	0.5	1	≤0.12- 1
Czech Republic	35	0.5	1	0.25-2
France	209	0.5	1	0.25-2
Germany	97	0.5	1	≤0.12- 1
Greece	34	0.5	0.5	0.25-1
Hungary	33	0.5	1	0.5-1
Israel	32	0.5	0.5	≤0.12- 2
Italy	186	0.5	1	0.12-4
Japan	58	0.5	1	0.25-4
Malaysia	31	0.5	1	≤0.12- 2
Mexico	42	0.5	1	0.25-2
Netherlands	17	ND*	ND*	≤0.12- 1
New Zealand	28	ND*	ND*	0.25-1
Philippines	64	0.5	0.5	0.03-1
Poland	74	0.5	1	≤0.12- 2
Portugal	17	ND*	ND*	≤0.12- 2
Republic of Ireland	45	0.5	1	0.25-1
Romania	16	ND*	ND*	0.25-1
Russia	34	0.5	1	≤0.12- 2
Slovakia	33	0.5	1	0.12-1
South Africa	54	0.5	1	0.25-2
South Korea	34	0.5	1	0.25-4
Spain	270	0.5	1	0.25-4
Sweden	34	0.5	1	0.25-2
Switzerland	33	0.5	0.5	0.25-2
Thailand	64	0.5	1	≤0.12- 2
Turkey	72	0.5	1	0.25-2
United Kingdom	111	0.5	0.5	≤0.12- 2
United States	128	0.5	1	≤0.12- 1

Table s4: *Staphylococcus* species. MIC of XF-73 Distribution by age of patients

Age	No of isolates	MIC_{50} (µg/ml)	MIC_{90} (µg/ml)
>5	139	0.5	1
5 to 17	124	0.5	1
18 to 65	1068	0.5	0.5
>65	834	0.5	1

Table s5: *Staphylococcus aureus* MIC of XF-73 Distribution by clinical presentation

ND* = Not determined as number of isolates too low to get a reliable $MIC_{50/90}$ value; SSTI = Skin and Soft Skin Infection; RTI = Respiratory Tract Infection; HAP = Hospital-Acquired Pneumonia; CSSTI = Complicated Skin and Soft Tissue Infection

	Total			MRSA			MSSA		
Indication (n)	MIC_{50} (µg/ml)	MIC_{90} (µg/ml)	MIC range (µg/ml)	MIC_{50} (µg/ml)	MIC_{90} (µg/ml)	MIC range (µg/ml)	MIC_{50} (µg/ml)	MIC_{90} (µg/ml)	MIC range (µg/ml)
SSTI (715)	0.5	1	0.25-4	0.5	1	0.25-4	0.5	1	0.25-4
UTI (1)	ND*	ND*	0.5	ND*	ND*	ND*	ND*	ND*	0.5
RTI (292)	0.5	1	0.25-4	0.5	1	0.25-4	0.5	1	0.25-4
HAP (168)	0.5	1	0.25-4	0.5	1	0.25-4	0.5	0.5	0.25-2
CSSTI (235)	0.5	1	0.25-2	0.5	1	0.25-2	0.5	1	0.25-2

Table s6: *Staphylococcus* species. MIC of XF-73 Distribution by clinical presentation

ND* = Not determined as number of isolates too low to get a reliable $MIC_{50/90}$ value; SSTI = Skin and Soft Skin Infection; RTI = Respiratory Tract Infection; HAP = Hospital-Acquired Pneumonia; CSSTI = Complicated Skin and Soft Tissue Infection

Indication	No of isolates	MIC_{50} (µg/ml)	MIC_{90} (µg/ml)	MIC range (µg/ml)
SSTI	1011	0.5	1	≤0.12-4
UTI	1	ND*	ND*	0.5
RTI	432	0.5	1	≤0.12-4
HAP	203	0.5	1	0.25-4
CSSTI	297	0.5	1	≤0.12-2

Isolate Number	*Staphylococcus* species	MIC (µg/ml) Values																							
			β Lactams					Carbapenem	Glycopeptides			Cephalosporins			Flouroquinolones			Oxazolidnone	Macrolides			Tetracyclines		Aminoglycoside	Antifolate
		XF-73	Penicillin	Oxacillin	Amoxicillin Clavulanate	Piperacillin/Tazobactam	Ampicillin	Meropenem	Vancomycin	Dalbavancin	Teicoplanin	Cefuroxime	Ceftriaxone	Ceftazidime	Levofloxacin	Ciprofloxacin	Moxifloxacin	Linezolid	Clindamycin	Clarithromycin	Erythromycin	Tetracycline	Tigecycline	Gentamicin	Trimethoprim/ Sulfmethoxazole
1	Methicillin-resistant *Staphylococcus aureus*	0.5	>64		16	>64	>32	8	1	≤0.06	0.25	>64	>32	>64	>8		4	2		>32		0.25	0.03		>32
2	Methicillin-resistant *Staphylococcus aureus*	0.5	64		8	16	>32	2	0.5	0.25	0.25	64	>32	64	8		1	2		>32		0.25	0.03		0.06
3	Methicillin-resistant *Staphylococcus aureus*	0.5	16		8	32	16	4	1	≤0.06	0.5	>64	>32	64	>8		4	2		>32		1	0.12		0.06
4	Methicillin-resistant *Staphylococcus aureus*	1	>64		2	16	>32	1	1	≤0.06	0.5	8	16	32	0.25		0.03	4		8		8	0.25		0.25
5	Methicillin-resistant *Staphylococcus aureus*	0.5	8		2	4	8	1	2	≤0.06	0.5	16	32	32	0.25		0.03	4		>32		0.5	0.25		0.12
6	Methicillin-resistant *Staphylococcus aureus*	0.5	64		8	32	>32	2	1	≤0.06	0.5	16	32	64	8		2	1		32		0.12	0.25		0.03
7	Methicillin-resistant *Staphylococcus aureus*	0.5	64		8	16	>32	0.5	1	≤0.06	0.5	16	32	4	4		1	2		32		0.12	0.12		0.03
8	Methicillin-resistant *Staphylococcus aureus*	0.5	16		8	16	16	1	1	≤0.06	0.25	16	32	8	>8		>8	2		>32		0.12	0.12		0.03
9	Methicillin-resistant *Staphylococcus aureus*	0.5	64		8	32	1	0.5	1	≤0.06	0.5	64	32	16	0.25		0.03	2		>32		0.12	0.25		0.06
10	Methicillin-resistant *Staphylococcus aureus*	1	64		8	32	>32	0.5	1	≤0.06	0.5	32	32	8	0.25		0.06	1		0.12		0.12	0.12		0.03
11	Methicillin-resistant *Staphylococcus aureus*	0.5	4		8	16	8	0.5	0.5	≤0.06	0.5	32	>32	8	8		1	1		>32		0.12	0.12		0.12
12	Methicillin-resistant *Staphylococcus aureus*	0.5	32		16	64	32	0.5	1	≤0.06	0.5	>64	>32	16	>8		2	2		>32		0.12	0.25		0.06
13	Methicillin-resistant *Staphylococcus aureus*	1	32		4	8	32	1	4	≤0.06	0.5	8	16	64	8		2	2		32		0.25	0.12		0.06
14	Methicillin-resistant *Staphylococcus aureus*	0.5	32		8	32	16	2	1	0.25	1	16	32	32	0.25		0.03	4		>32		2	0.12		0.06
15	Methicillin-resistant *Staphylococcus aureus*	0.5	32		8	32	32	2	2	≤0.06	1	64	32	64	0.25		0.06	2		0.25		0.25	0.12		0.12
16	Methicillin-resistant *Staphylococcus aureus*	0.5	8		8	64	4	4	2	≤0.06	1	>64	>32	64	>8		2	2		>32		32	0.25		0.5
17	Methicillin-resistant *Staphylococcus aureus*	0.5	32		8	64	16	4	1	≤0.06	0.5	>64	>32	64	0.25		0.25	4		>32		4	0.12		0.25
18	Methicillin-resistant *Staphylococcus aureus*	0.5	64		32	>64	>32	>16	1	≤0.06	2	>64	>32	>64	>8		8	2		>32		64	0.5		0.25
19	Methicillin-resistant *Staphylococcus aureus*	0.5	32		8	32	32	4	2	≤0.06	1	64	32	64	0.12		0.03	4		>32		32	0.12		0.06
20	Methicillin-resistant *Staphylococcus aureus*	0.5	64		32	>64	>32	>16	1	≤0.06	2	>64	>32	>64	>8		4	2		>32		64	0.5		0.25
21	Methicillin-resistant *Staphylococcus aureus*	0.5	32		16	64	32	4	2	≤0.06	1	>64	>32	32	0.25		0.06	2		>32		32	0.12		0.06
22	Methicillin-resistant *Staphylococcus aureus*	1	16		8	32	16	4	1	≤0.06	1	64	>32	64	0.25		0.06	2		>32		32	0.12		0.12
23	Methicillin-resistant *Staphylococcus aureus*	1	32		8	64	32	4	2	≤0.06	2	>64	>32	64	0.25		0.06	4		>32		32	0.25		0.06
24	Methicillin-resistant *Staphylococcus aureus*	0.5	64		32	>64	>32	>16	1	≤0.06	4	>64	>32	>64	8		1	2		>32		64	0.25		0.25
25	Methicillin-resistant *Staphylococcus aureus*	0.5	64		32	>64	>32	0.12	1	≤0.06	2	>64	>32	>64	8		1	2		>32		64	0.25		0.25
26	Methicillin-resistant *Staphylococcus aureus*	0.5	64		32	>64	>32	>16	1	≤0.06	2	>64	>32	>64	8		1	2		>32		64	0.25		0.25
27	Methicillin-resistant *Staphylococcus aureus*	0.5	16		8	16	16	4	2	≤0.06	1	>64	32	64	8		2	2		>32		64	0.25		0.06
28	Methicillin-resistant *Staphylococcus aureus*	0.5	32		8	8	32	2	1	≤0.06	1	64	32	64	8		2	2		>32		32	0.12		0.06
29	Methicillin-resistant *Staphylococcus aureus*	0.5	64		>32	>64	>32	>16	1	≤0.06	1	>64	>32	>64	8		1	2		>32		64	0.25		0.25
30	Methicillin-resistant *Staphylococcus aureus*	0.5	64		32	>64	>32	>16	1	≤0.06	1	>64	>32	>64	8		1	2		>32		64	0.25		0.25
31	Methicillin-resistant *Staphylococcus aureus*	0.5	32		8	8	32	2	1	≤0.06	1	64	>32	64	8		2	2		0.25		0.06	0.06		0.06
32	Methicillin-resistant *Staphylococcus aureus*	0.5	16		16	>64	16	16	2	≤0.06	2	>64	>32	>64	8		2	2		0.25		0.12	0.12		0.06
33	Methicillin-resistant *Staphylococcus aureus*	1	32		8	8	32	2	2	≤0.06	1	64	>32	64	8		2	2		0.25		0.12	0.06		0.06
34	Methicillin-resistant *Staphylococcus aureus*	0.5	32		8	8	32	2	1	≤0.06	0.5	64	32	64	8		2	2		0.25		0.25	0.06		0.06
35	Methicillin-resistant *Staphylococcus aureus*	1	32		8	16	>32	2	1	≤0.06	0.5	>64	>32	>64	8		2	2		>32		0.25	0.12		0.12
36	Methicillin-resistant *Staphylococcus aureus*	0.5	64		8	32	>32	2	1	≤0.06	0.5	>64	>32	>64	8		2	2		0.25		32	0.12		0.06
37	Methicillin-resistant *Staphylococcus aureus*	1	32		8	16	32	2	1	≤0.06	0.5	>64	>32	64	8		2	2		>32		32	0.12		0.06
38	Methicillin-resistant *Staphylococcus aureus*	0.5	32		8	16	16	4	1	0.12	0.5	32	32	32	4		1	2		0.25		32	0.12		0.06
39	Methicillin-resistant *Staphylococcus aureus*	1	32		16	32	>32	8	1	≤0.06	1	>64	>32	>64	8		2	2		>32		0.12	0.06		0.06
40	Methicillin-resistant *Staphylococcus aureus*	0.5	32		8	16	32	2	1	0.12	0.5	>64	>32	64	4		1	2		16		64	0.25		0.06
41	Methicillin-resistant *Staphylococcus aureus*	1	32		4	8	32	2	2	≤0.06	0.5	32	>32	32	4		2	2		>32		0.12	0.06		0.06
42	Methicillin-resistant *Staphylococcus aureus*	0.5	32		8	16	32	2	2	≤0.06	2	>64	32	64	8		2	2		>32		0.25	0.12		0.12
43	Methicillin-resistant *Staphylococcus aureus*	1	64		32	>64	>32	>16	2	≤0.06	2	>64	>32	>64	4		2	2		>32		32	0.25		1
44	Methicillin-resistant *Staphylococcus aureus*	1	64		32	64	>32	16	2	≤0.06	1	>64	>32	>64	4		2	2		>32		32	0.25		2
45	Methicillin-resistant *Staphylococcus aureus*	0.5	64		32	>64	>32	>16	2	≤0.06	2	>64	>32	>64	8		2	1		>32		32	0.25		2
46	Methicillin-resistant *Staphylococcus aureus*	0.5	2		0.5	4	1	8	1	≤0.06	1	>64	32	>64	1		0.12	2		4		32	0.25		8
47	Methicillin-resistant *Staphylococcus aureus*	2	32		32	64	32	16	1	≤0.06	0.5	>64	>32	>64	>8		4	2		>32		0.25	0.12		0.06

Isolate Number	*Staphylococcus* species	MIC (µg/ml) Values																							
			β Lactams					Carbapenem	Glycopeptides			Cephalosporins			Flouroquinolones			Oxazolidnone	Macrolides			Tetracyclines		Aminoglycoside	Antifolate
		XF-73	Penicillin	Oxacillin	Amoxicillin Clavulanate	Piperacillin/Tazobactam	Ampicillin	Meropenem	Vancomycin	Dalbavancin	Teicoplanin	Cefuroxime	Ceftriaxone	Ceftazidime	Levofloxacin	Ciprofloxacin	Moxifloxacin	Linezolid	Clindamycin	Clarithromycin	Erythromycin	Tetracycline	Tigecycline	Gentamicin	Trimethoprim/ Sulfmethoxazole
48	Methicillin-resistant *Staphylococcus aureus*	0.5	64		>32	>64	>32	>16	0.5	≤0.06	1	>64	>32	>64	8		2	2		>32		0.25	0.25		0.25
49	Methicillin-resistant *Staphylococcus aureus*	0.5	8		8	16	8	2	1	0.25	0.5	32	32	64	>8		4	4		>32		0.25	0.12		0.06
50	Methicillin-resistant *Staphylococcus aureus*	0.5	16		8	32	16	4	0.5	≤0.06	0.5	64	32	64	>8		4	4		16		0.25	0.12		0.06
51	Methicillin-resistant *Staphylococcus aureus*	1	>64		>32	>64	>32	>16	0.5	≤0.06	1	>64	>32	>64	8		2	2		>32		0.25	0.25		0.25
52	Methicillin-resistant *Staphylococcus aureus*	0.5	32		32	>64	32	16	1	≤0.06	0.5	>64	>32	>64	>8		4	2		>32		0.25	0.12		0.12
53	Methicillin-resistant *Staphylococcus aureus*	0.5	32		32	64	32	16	0.5	≤0.06	0.5	>64	>32	>64	>8		4	2		>32		0.5	0.25		0.06
54	Methicillin-resistant *Staphylococcus aureus*	0.5	64		32	>64	>32	>16	0.5	≤0.06	1	>64	>32	>64	8		4	2		>32		0.5	0.25		0.5
55	Methicillin-resistant *Staphylococcus aureus*	0.5	64		32	>64	>32	>16	1	≤0.06	0.5	>64	>32	>64	8		2	4		>32		32	0.25		32
56	Methicillin-resistant *Staphylococcus aureus*	1	2		0.5	4	1	4	2	≤0.06	0.25	32	32	>64	1		0.25	2		1		0.5	0.25		0.06
57	Methicillin-resistant *Staphylococcus aureus*	1	64		32	>64	>32	1	1	≤0.06	2	>64	>32	32	8		2	1		>32		64	0.5		>32
58	Methicillin-resistant *Staphylococcus aureus*	1	64		32	>64	>32	1	0.5	≤0.06	1	>64	>32	16	>8		4	2		>32		32	0.25		>32
59	Methicillin-resistant *Staphylococcus aureus*	1	64		32	64	32	0.5	0.5	≤0.06	0.25	>64	>32	16	8		4	2		>32		0.25	0.25		0.12
60	Methicillin-resistant *Staphylococcus aureus*	0.5	64		32	64	16	1	0.5	≤0.06	0.5	>64	>32	32	8		2	2		>32		0.25	0.25		0.06
61	Methicillin-resistant *Staphylococcus aureus*	1	64		32	>64	32	0.5	0.5	≤0.06	0.5	>64	>32	16	>8		4	2		>32		0.25	0.25		0.06
62	Methicillin-resistant *Staphylococcus aureus*	0.5	64		32	64	32	0.5	0.5	≤0.06	0.25	>64	>32	16	>8		4	2		0.25		0.5	0.25		0.06
63	Methicillin-resistant *Staphylococcus aureus*	1	64		32	>64	32	0.5	0.5	≤0.06	0.5	>64	>32	32	>8		8	2		0.25		0.25	0.25		0.06
64	Methicillin-resistant *Staphylococcus aureus*	0.5	64		32	>64	32	1	0.5	≤0.06	0.25	>64	>32	32	>8		4	2		>32		0.25	0.25		0.06
65	Methicillin-resistant *Staphylococcus aureus*	1	16		8	16	16	0.5	1	≤0.06	0.5	8	32	8	8		2	2		>32		0.5	0.25		0.06
66	Methicillin-resistant *Staphylococcus aureus*	0.5	64		32	>64	32	0.5	1	≤0.06	0.5	>64	>32	>64	>8		4	2		0.25		0.25	0.12		0.06
67	Methicillin-resistant *Staphylococcus aureus*	1	16		16	64	16	4	1	≤0.06	0.5	16	>32	64	>8		8	4		>32		0.5	0.12		0.06
68	Methicillin-resistant *Staphylococcus aureus*	0.5	1		4	8	2	1	1	≤0.06	0.25	8	16	64	0.25		0.12	4		0.25		>64	0.5		1
69	Methicillin-resistant *Staphylococcus aureus*	1	32		16	32	32	4	1	≤0.06	0.5	>64	32	64	0.5		0.06	2		0.12		0.25	0.25		0.06
70	Methicillin-resistant *Staphylococcus aureus*	0.5	8		4	4	8	1	1	≤0.06	0.5	8	16	32	0.25		0.06	2		0.25		0.12	0.12		0.06
71	Methicillin-resistant *Staphylococcus aureus*	0.5	16		8	32	16	2	0.5	0.25	0.5	16	32	64	0.25		0.03	2		>32		64	0.12		0.5
72	Methicillin-resistant *Staphylococcus aureus*	0.5	32		8	16	32	1	1	0.12	0.5	8	16	32	4		1	2		32		0.25	0.12		0.06
73	Methicillin-resistant *Staphylococcus aureus*	0.5	16		8	16	16	2	1	≤0.06	0.5	64	32	64	0.5		0.06	2		>32		16	0.25		0.06
74	Methicillin-resistant *Staphylococcus aureus*	1	64		16	32	>32	2	1	≤0.06	0.5	64	16	64	4		2	2		32		0.06	0.03		0.06
75	Methicillin-resistant *Staphylococcus aureus*	0.25	8		4	8	16	0.5	1	≤0.06	0.5	8	16	32	0.25		0.03	2		0.25		>64	0.25		0.06
76	Methicillin-resistant *Staphylococcus aureus*	0.5	16		4	8	8	0.5	1	≤0.06	0.5	16	16	16	0.25		0.03	2		>32		32	0.25		0.06
77	Methicillin-resistant *Staphylococcus aureus*	1	32		16	32	16	2	1	0.25	0.5	>64	32	64	0.25		0.06	2		>32		32	1		1
78	Methicillin-resistant *Staphylococcus aureus*	0.5	64		8	16	>32	2	1	≤0.06	0.5	32	16	64	4		1	2		0.25		0.25	0.12		0.06
79	Methicillin-resistant *Staphylococcus aureus*	0.5	4		0.5	8	1	8	1	≤0.06	0.5	>64	>32	>64	>8		>8	2		>32		64	0.25		32
80	Methicillin-resistant *Staphylococcus aureus*	0.5	64		>32	>64	>32	>16	0.5	≤0.06	1	>64	>32	>64	8		2	2		>32		0.5	0.12		0.12
81	Methicillin-resistant *Staphylococcus aureus*	0.5	32		32	>64	32	>16	1	≤0.06	2	>64	>32	>64	>8		8	2		>32		0.25	0.12		0.12
82	Methicillin-resistant *Staphylococcus aureus*	1	>64		>32	>64	>32	>16	0.5	≤0.06	1	>64	>32	>64	8		0.015	2		>32		1	0.015		0.06
83	Methicillin-resistant *Staphylococcus aureus*	0.5	64		32	>64	32	16	1	≤0.06	0.5	>64	>32	>64	8		2	2		>32		>64	0.25		16
84	Methicillin-resistant *Staphylococcus aureus*	0.5	64		32	64	>32	8	1	0.25	0.5	>64	>32	>64	0.5		0.12	1		0.25		32	0.12		32
85	Methicillin-resistant *Staphylococcus aureus*	0.5	64		16	64	>32	4	0.5	0.25	0.25	64	>32	>64	4		1	2		>32		64	0.12		>32
86	Methicillin-resistant *Staphylococcus aureus*	0.5	64		8	32	>32	2	1	≤0.06	1	64	32	64	4		1	2		>32		32	0.06		>32
87	Methicillin-resistant *Staphylococcus aureus*	0.5	>64		>32	>64	>32	>16	0.5	≤0.06	1	>64	>32	>64	>8		4	2		>32		0.25	0.12		0.12
88	Methicillin-resistant *Staphylococcus aureus*	0.5	64		32	>64	>32	>16	0.5	≤0.06	1	>64	>32	>64	8		1	1		>32		0.12	0.06		0.06
89	Methicillin-resistant *Staphylococcus aureus*	0.5	64		>32	>64	>32	>16	1	≤0.06	0.5	>64	>32	>64	>8		4	1		>32		0.5	0.12		0.06
90	Methicillin-resistant *Staphylococcus aureus*	0.5	64		32	>64	>32	>16	0.5	≤0.06	0.5	>64	>32	>64	8		4	1		>32		0.12	0.12		0.06
91	Methicillin-resistant *Staphylococcus aureus*	0.5	64		32	>64	>32	>16	0.5	≤0.06	0.5	>64	>32	>64	8		2	1		>32		0.12	0.06		0.06
92	Methicillin-resistant *Staphylococcus aureus*	0.5	32		16	64	32	16	0.5	≤0.06	0.5	>64	>32	>64	8		2	1		>32		0.12	0.12		0.06
93	Methicillin-resistant *Staphylococcus aureus*	0.5	8		8	16	16	2	0.5	≤0.06	0.25	>64	32	>64	>8		8	1		>32		0.25	0.25		0.06
94	Methicillin-resistant *Staphylococcus aureus*	1	32		16	64	32	8	0.5	≤0.06	0.5	>64	>32	>64	>8		2	1		0.12		0.25	0.12		0.03

Table s2: MIC Data for the Staphylococcus species tested

Isolate Number	*Staphylococcus* species	MIC (µg/ml) Values	β Lactams					Carbapenem	Glycopeptides			Cephalosporins			Flouroquinolones			Oxazolidnone	Macrolides			Tetracyclines		Aminoglycoside	Antifolate
		XF-73	Penicillin	Oxacillin	Amoxicillin Clavulanate	Piperacillin/Tazobactam	Ampicillin	Meropenem	Vancomycin	Dalbavancin	Teicoplanin	Cefuroxime	Ceftriaxone	Ceftazidime	Levofloxacin	Ciprofloxacin	Moxifloxacin	Linezolid	Clindamycin	Clarithromycin	Erythromycin	Tetracycline	Tigecycline	Gentamicin	Trimethoprim/ Sulfmethoxazole
95	Methicillin-resistant *Staphylococcus aureus*	0.5	16		16	32	16	4	0.5	≤0.06	0.25	>64	>32	>64	>8		8	1		0.12		0.12	0.12		0.06
96	Methicillin-resistant *Staphylococcus aureus*	0.5	8		4	8	8	2	0.5	≤0.06	0.5	32	32	>64	>8		8	2		0.12		0.12	0.12		0.06
97	Methicillin-resistant *Staphylococcus aureus*	1	32		32	>64	32	>16	0.5	≤0.06	0.5	>64	>32	>64	8		2	2		>32		0.12	0.12		0.12
98	Methicillin-resistant *Staphylococcus aureus*	0.5	32		16	64	32	>16	0.5	≤0.06	0.5	>64	>32	>64	8		2	2		>32		0.12	0.12		0.06
99	Methicillin-resistant *Staphylococcus aureus*	0.5	4		1	4	4	0.12	1	≤0.06	0.5	8	4	16	0.25		0.06	2		0.25		0.25	0.06		0.06
100	Methicillin-resistant *Staphylococcus aureus*	0.5	8		8	32	8	2	1	0.12	0.5	2	32	64	0.25		0.06	2		>32		0.25	0.12		0.12
101	Methicillin-resistant *Staphylococcus aureus*	0.5	64		2	8	32	0.25	1	0.12	0.5	8	16	64	0.25		0.06	2		0.25		0.25	0.12		0.06
102	Methicillin-resistant *Staphylococcus aureus*	0.25	0.03		0.12	0.5	0.06	0.12	1	≤0.06	0.5	1	4	16	0.12		0.03	2		0.06		0.25	0.12		0.25
103	Methicillin-resistant *Staphylococcus aureus*	0.25	4		2	4	8	1	1	≤0.06	0.5	8	16	32	4		2	2		0.12		0.12	0.06		0.06
104	Methicillin-resistant *Staphylococcus aureus*	0.5	4		4	16	4	1	1	≤0.06	0.5	32	32	32	>8		8	1		>32		0.25	0.12		0.06
105	Methicillin-resistant *Staphylococcus aureus*	1	64		16	32	32	16	2	0.25	1	>64	>32	>64	8		2	4		>32		0.5	0.12		0.06
106	Methicillin-resistant *Staphylococcus aureus*	0.5	32		16	64	32	16	1	≤0.06	1	>64	>32	>64	8		2	2		>32		0.25	0.12		0.06
107	Methicillin-resistant *Staphylococcus aureus*	1	32		16	64	32	8	1	≤0.06	1	64	32	32	0.25		0.03	2		0.25		32	0.12		0.06
108	Methicillin-resistant *Staphylococcus aureus*	1	32		16	64	32	4	1	≤0.06	0.5	>64	>32	>64	>8		4	2		>32		0.25	0.12		0.06
109	Methicillin-resistant *Staphylococcus aureus*	1	64		>32	>64	32	>16	1	≤0.06	2	>64	>32	>64	8		2	2		>32		0.25	0.06		0.12
110	Methicillin-resistant *Staphylococcus aureus*	0.5	32		16	64	32	4	1	≤0.06	0.5	>64	>32	>64	>8		4	2		>32		0.25	0.12		0.06
111	Methicillin-resistant *Staphylococcus aureus*	1	8		4	8	8	2	1	0.12	1	8	16	32	4		2	2		0.25		0.25	0.06		0.06
112	Methicillin-resistant *Staphylococcus aureus*	1	32		16	32	32	8	2	≤0.06	1	>64	>32	>64	8		2	4		>32		0.25	0.12		0.06
113	Methicillin-resistant *Staphylococcus aureus*	1	16		16	64	16	8	1	≤0.06	0.5	>64	>32	>64	>8		8	2		>32		0.25	0.06		0.06
114	Methicillin-resistant *Staphylococcus aureus*	1	32		8	8	16	2	0.5	≤0.06	0.5	16	16	32	8		1	2		16		0.25	0.12		0.06
115	Methicillin-resistant *Staphylococcus aureus*	1	32		16	32	32	2	1	≤0.06	0.5	>64	>32	64	0.25		0.03	2		0.25		0.12	0.12		0.12
116	Methicillin-resistant *Staphylococcus aureus*	0.5	32		8	32	32	2	1	≤0.06	0.5	16	32	64	4		2	2		0.25		0.12	0.06		0.06
117	Methicillin-resistant *Staphylococcus aureus*	0.5	64		16	32	>32	16	1	≤0.06	0.5	>64	>32	>64	8		2	2		32		0.25	0.25		0.12
118	Methicillin-resistant *Staphylococcus aureus*	0.5	64		16	16	>32	8	1	≤0.06	0.25	>64	>32	>64	8		2	1		32		0.25	0.12		0.12
119	Methicillin-resistant *Staphylococcus aureus*	0.5	64		8	32	32	2	1	≤0.06	0.5	16	32	64	4		2	2		32		0.12	0.12		0.12
120	Methicillin-resistant *Staphylococcus aureus*	0.5	64		16	32	32	16	1	≤0.06	0.5	>64	>32	>64	8		2	2		0.25		0.12	0.06		0.06
121	Methicillin-resistant *Staphylococcus aureus*	0.5	32		16	64	32	16	1	≤0.06	1	>64	>32	>64	8		2	2		>32		0.25	0.25		0.12
122	Methicillin-resistant *Staphylococcus aureus*	0.5	64		16	64	32	16	0.5	0.25	0.5	>64	>32	>64	>8		2	2		32		0.25	0.25		0.06
123	Methicillin-resistant *Staphylococcus aureus*	0.5	64		16	64	32	16	1	≤0.06	0.5	>64	>32	>64	8		2	2		>32		0.25	0.25		0.5
124	Methicillin-resistant *Staphylococcus aureus*	0.5	32		8	16	8	1	1	≤0.06	1	16	32	64	0.12		0.03	2		0.25		0.25	0.25		0.06
125	Methicillin-resistant *Staphylococcus aureus*	0.5	64		16	64	>32	16	2	0.25	1	>64	>32	>64	>8		4	2		32		0.25	0.12		0.06
126	Methicillin-resistant *Staphylococcus aureus*	1	32		16	32	32	8	2	≤0.06	1	>64	>32	>64	>8		8	2		>32		0.25	0.12		0.06
127	Methicillin-resistant *Staphylococcus aureus*	0.5	32		16	16	32	8	1	≤0.06	0.5	>64	16	>64	>8		2	2		32		0.12	0.03		0.06
128	Methicillin-resistant *Staphylococcus aureus*	0.5	8		2	4	8	0.5	2	≤0.06	1	8	16	32	0.06		0.03	2		0.25		0.25	0.12		0.06
129	Methicillin-resistant *Staphylococcus aureus*	0.5	16		32	>64	16	>16	2	≤0.06	1	>64	>32	>64	>8		8	32		0.25		0.25	0.06		0.06
130	Methicillin-resistant *Staphylococcus aureus*	0.5	64		16	64	32	16	2	≤0.06	1	>64	>32	>64	8		2	2		>32		0.25	0.06		0.06
131	Methicillin-resistant *Staphylococcus aureus*	0.5	64		16	64	32	16	1	≤0.06	0.5	>64	>32	>64	>8		4	2		>32		0.25	0.06		0.06
132	Methicillin-resistant *Staphylococcus aureus*	0.5	32		16	64	32	16	1	≤0.06	0.5	>64	>32	>64	>8		4	2		0.25		0.12	0.06		0.06
133	Methicillin-resistant *Staphylococcus aureus*	1	32		16	>64	32	16	1	0.12	1	>64	>32	>64	>8		4	2		0.25		0.25	0.12		0.06
134	Methicillin-resistant *Staphylococcus aureus*	0.5	32		16	32	32	8	1	≤0.06	0.5	>64	>32	>64	8		2	2		>32		0.12	0.06		0.06
135	Methicillin-resistant *Staphylococcus aureus*	0.5	32		16	32	32	1	1	≤0.06	0.5	>64	32	16	8		2	2		>32		32	0.25		0.06
136	Methicillin-resistant *Staphylococcus aureus*	0.5	16		16	64	16	4	1	0.12	0.5	>64	>32	>64	>8		2	2		>32		0.25	0.25		0.06
137	Methicillin-resistant *Staphylococcus aureus*	1	16		16	64	16	1	1	0.12	0.5	>64	>32	32	>8		8	2		>32		0.25	0.25		0.06
138	Methicillin-resistant *Staphylococcus aureus*	1	64		16	16	32	16	0.5	≤0.06	0.25	>64	>32	>64	>8		4	2		>32		0.25	0.12		0.06
139	Methicillin-resistant *Staphylococcus aureus*	0.5	32		32	64	32	8	1	0.12	0.5	>64	>32	>64	>8		4	2		>32		0.25	0.25		0.12
140	Methicillin-resistant *Staphylococcus aureus*	0.5	32		32	64	32	4	0.5	≤0.06	0.25	>64	>32	>64	>8		8	2		0.25		0.25	0.12		0.12
141	Methicillin-resistant *Staphylococcus aureus*	0.5	32		16	64	32	4	1	≤0.06	1	>64	>32	>64	>8		4	2		>32		0.25	0.12		0.06

Isolate Number	*Staphylococcus* species	MIC (µg/ml) Values																							
			β Lactams					Carbapenem	Glycopeptides			Cephalosporins			Flouroquinolones			Oxazolidnone	Macrolides			Tetracyclines		Aminoglycoside	Antifolate
		XF-73	Penicillin	Oxacillin	Amoxicillin Clavulanate	Piperacillin/Tazobactam	Ampicillin	Meropenem	Vancomycin	Dalbavancin	Teicoplanin	Cefuroxime	Ceftriaxone	Ceftazidime	Levofloxacin	Ciprofloxacin	Moxifloxacin	Linezolid	Clindamycin	Clarithromycin	Erythromycin	Tetracycline	Tigecycline	Gentamicin	Trimethoprim/ Sulfmethoxazole
142	Methicillin-resistant *Staphylococcus aureus*	0.5	32		16	64	32	4	1	≤0.06	0.5	>64	>32	>64	>8		4	2		>32		0.25	0.12		0.06
143	Methicillin-resistant *Staphylococcus aureus*	0.5	16		32	>64	32	16	0.5	≤0.06	0.25	>64	>32	>64	>8		8	4		0.25		0.25	0.12		0.12
144	Methicillin-resistant *Staphylococcus aureus*	1	64		16	32	>32	2	1	≤0.06	0.5	64	32	64	8		2	2		>32		64	0.25		0.06
145	Methicillin-resistant *Staphylococcus aureus*	0.5	32		32	>64	32	16	0.5	≤0.06	0.5	>64	>32	>64	>8		4	2		0.12		0.12	0.12		0.06
146	Methicillin-resistant *Staphylococcus aureus*	0.5	32		0.5	8	8	4	1	≤0.06	0.5	>64	>32	>64	0.25		0.06	2		0.25		0.25	0.12		0.06
147	Methicillin-resistant *Staphylococcus aureus*	1	32		8	64	32	8	2	0.12	2	>64	>32	64	>8		2	2		0.25		0.25	0.06		0.06
148	Methicillin-resistant *Staphylococcus aureus*	0.5	32		1	32	16	4	1	≤0.06	0.5	>64	>32	>64	>8		4	2		>32		0.25	0.06		0.06
149	Methicillin-resistant *Staphylococcus aureus*	1	32		16	64	32	8	1	≤0.06	0.5	>64	>32	>64	>8		8	2		>32		0.12	0.06		0.06
150	Methicillin-resistant *Staphylococcus aureus*	0.5	32		16	64	32	8	1	≤0.06	1	>64	>32	>64	>8		4	2		>32		0.25	0.06		0.06
151	Methicillin-resistant *Staphylococcus aureus*	0.5	32		8	32	16	4	1	≤0.06	0.25	>64	>32	>64	>8		4	1		>32		0.12	0.06		0.06
152	Methicillin-resistant *Staphylococcus aureus*	0.5	16		8	32	16	2	1	≤0.06	0.5	64	>32	>64	>8		4	2		>32		0.25	0.06		0.06
153	Methicillin-resistant *Staphylococcus aureus*	0.5	4		2	8	4	1	1	≤0.06	0.5	8	16	32	>8		4	2		0.5		16	0.12		0.06
154	Methicillin-resistant *Staphylococcus aureus*	0.5	8		2	4	8	0.5	1	≤0.06	0.5	8	16	32	0.12		0.03	2		0.25		16	0.12		0.06
155	Methicillin-resistant *Staphylococcus aureus*	0.5	64		8	32	32	2	1	≤0.06	0.5	>64	>32	64	4		2	2		>32		0.12	0.06		0.06
156	Methicillin-resistant *Staphylococcus aureus*	0.5	16		16	32	16	16	1	≤0.06	0.5	>64	>32	64	>8		4	2		>32		0.25	0.06		0.06
157	Methicillin-resistant *Staphylococcus aureus*	0.5	64		8	16	>32	2	1	≤0.06	1	64	32	32	0.25		0.03	2		4		64	0.25		0.12
158	Methicillin-resistant *Staphylococcus aureus*	0.5	8		16	32	8	4	0.5	≤0.06	0.25	64	32	64	>8		4	2		>32		0.25	0.25		0.06
159	Methicillin-resistant *Staphylococcus aureus*	0.5	8		2	4	8	0.5	1	0.12	0.5	8	16	64	0.12		0.015	2		0.25		0.12	0.12		0.12
160	Methicillin-resistant *Staphylococcus aureus*	0.5	16		16	32	16	4	0.5	≤0.06	0.25	>64	>32	64	>8		4	2		>32		0.25	0.12		0.12
161	Methicillin-resistant *Staphylococcus aureus*	0.5	32		16	64	32	8	0.5	≤0.06	0.25	>64	>32	>64	>8		4	2		0.25		0.5	0.5		0.12
162	Methicillin-resistant *Staphylococcus aureus*	0.5	8		8	32	8	1	0.5	≤0.06	0.25	32	32	64	>8		4	2		>32		0.25	0.25		0.06
163	Methicillin-resistant *Staphylococcus aureus*	0.5	16		16	64	16	4	1	≤0.06	0.5	>64	>32	>64	>8		4	2		>32		0.25	0.25		0.12
164	Methicillin-resistant *Staphylococcus aureus*	0.5	32		16	32	16	4	0.5	≤0.06	0.5	>64	>32	64	>8		4	2		>32		0.25	0.12		0.06
165	Methicillin-resistant *Staphylococcus aureus*	0.5	8		8	16	8	2	0.5	≤0.06	0.25	16	32	8	>8		4	2		>32		0.25	0.25		0.06
166	Methicillin-resistant *Staphylococcus aureus*	0.5	16		16	32	16	4	0.5	≤0.06	0.5	>64	>32	64	>8		4	2		>32		0.25	0.12		0.06
167	Methicillin-resistant *Staphylococcus aureus*	1	16		16	32	16	4	1	≤0.06	0.25	>64	>32	>64	>8		4	2		>32		0.25	0.25		0.12
168	Methicillin-resistant *Staphylococcus aureus*	0.5	32		16	64	32	8	1	≤0.06	0.5	>64	>32	>64	>8		4	2		16		0.25	0.12		0.06
169	Methicillin-resistant *Staphylococcus aureus*	0.5	16		8	32	16	4	0.5	≤0.06	0.25	>64	32	64	>8		4	2		>32		0.12	0.06		0.06
170	Methicillin-resistant *Staphylococcus aureus*	0.5	64		32	>64	32	16	0.5	≤0.06	0.25	>64	>32	>64	>8		>8	2		>32		0.12	0.06		0.06
171	Methicillin-resistant *Staphylococcus aureus*	0.5	16		8	32	16	4	0.5	≤0.06	0.25	32	32	64	>8		2	2		>32		0.12	0.06		0.06
172	Methicillin-resistant *Staphylococcus aureus*	0.5	32		16	64	32	16	0.5	≤0.06	0.25	>64	>32	>64	>8		>8	2		>32		0.12	0.06		0.06
173	Methicillin-resistant *Staphylococcus aureus*	0.5	16		16	32	16	4	0.5	≤0.06	0.25	>64	>32	64	>8		4	2		>32		0.12	0.06		0.03
174	Methicillin-resistant *Staphylococcus aureus*	0.5	16		16	64	16	4	0.5	≤0.06	0.25	>64	>32	>64	8		4	2		>32		0.12	0.12		0.06
175	Methicillin-resistant *Staphylococcus aureus*	0.5	16		8	32	16	2	0.5	≤0.06	0.25	32	32	64	8		4	2		>32		0.12	0.12		0.06
176	Methicillin-resistant *Staphylococcus aureus*	0.5	32		16	64	32	4	0.5	≤0.06	0.25	>64	>32	>64	8		4	2		>32		0.25	0.12		0.06
177	Methicillin-resistant *Staphylococcus aureus*	0.5	8		16	64	8	2	0.5	0.12	0.25	>64	>32	>64	8		4	2		>32		0.12	0.12		0.06
178	Methicillin-resistant *Staphylococcus aureus*	0.5	32		16	32	16	4	0.5	≤0.06	0.25	>64	>32	>64	8		4	2		>32		0.12	0.12		0.06
179	Methicillin-resistant *Staphylococcus aureus*	0.5	16		8	64	16	4	0.5	≤0.06	0.5	>64	>32	>64	8		2	2		0.12		0.12	0.12		0.06
180	Methicillin-resistant *Staphylococcus aureus*	0.5	32		16	64	32	16	1	0.12	0.25	>64	>32	>64	>8		4	2		>32		0.25	0.12		0.06
181	Methicillin-resistant *Staphylococcus aureus*	0.5	16		8	16	8	4	1	≤0.06	1	>64	>32	64	>8		>8	2		>32		0.25	0.06		0.06
182	Methicillin-resistant *Staphylococcus aureus*	0.5	64		32	64	32	16	1	≤0.06	0.5	>64	>32	>64	>8		2	2		0.25		0.25	0.06		0.06
183	Methicillin-resistant *Staphylococcus aureus*	0.5	8		16	32	8	2	1	≤0.06	1	>64	>32	64	>8		>8	4		>32		0.25	0.12		0.06
184	Methicillin-resistant *Staphylococcus aureus*	1	64		8	32	32	2	2	≤0.06	0.5	>64	>32	>64	4		1	2		32		64	0.06		0.25
185	Methicillin-resistant *Staphylococcus aureus*	1	32		16	64	32	16	1	≤0.06	0.25	>64	>32	>64	>8		4	2		>32		0.25	0.06		0.06
186	Methicillin-resistant *Staphylococcus aureus*	1	8		8	16	16	2	1	≤0.06	0.5	>64	>32	64	>8		>8	2		>32		0.25	0.06		0.06
187	Methicillin-resistant *Staphylococcus aureus*	0.5	8		4	8	8	2	2	≤0.06	1	32	>32	64	0.25		0.06	2		>32		0.25	0.06		0.06
188	Methicillin-resistant *Staphylococcus aureus*	0.5	64		16	64	>32	16	1	≤0.06	0.5	>64	>32	>64	>8		2	2		>32		0.12	0.06		0.06

Isolate Number	*Staphylococcus* species	MIC (µg/ml) Values																							
			β Lactams					Carbapenem	Glycopeptides			Cephalosporins			Flouroquinolones			Oxazolidnone	Macrolides			Tetracyclines		Aminoglycoside	Antifolate
		XF-73	Penicillin	Oxacillin	Amoxicillin Clavulanate	Piperacillin/Tazobactam	Ampicillin	Meropenem	Vancomycin	Dalbavancin	Teicoplanin	Cefuroxime	Ceftriaxone	Ceftazidime	Levofloxacin	Ciprofloxacin	Moxifloxacin	Linezolid	Clindamycin	Clarithromycin	Erythromycin	Tetracycline	Tigecycline	Gentamicin	Trimethoprim/ Sulfmethoxazole
189	Methicillin-resistant *Staphylococcus aureus*	0.5	32		16	>64	32	16	1	≤0.06	0.5	>64	>32	>64	>8		2	2		>32		0.25	0.12		0.06
190	Methicillin-resistant *Staphylococcus aureus*	0.5	32		32	4	32	16	1	0.12	0.25	>64	>32	64	8		4	2		>32		0.25	0.12		0.06
191	Methicillin-resistant *Staphylococcus aureus*	0.5	32		8	32	32	2	1	≤0.06	0.5	>64	32	64	0.25		0.03	2		0.12		0.25	0.12		0.06
192	Methicillin-resistant *Staphylococcus aureus*	0.5	64		8	16	16	1	0.5	≤0.06	0.5	32	32	32	0.12		0.03	2		0.12		0.12	0.12		0.06
193	Methicillin-resistant *Staphylococcus aureus*	1	32		8	32	32	2	0.5	≤0.06	0.25	16	16	64	0.25		0.03	1		0.12		0.12	0.12		0.06
194	Methicillin-resistant *Staphylococcus aureus*	0.5	64		32	>64	32	16	0.5	≤0.06	0.25	>64	>32	>64	>8		4	1		>32		0.5	0.06		0.25
195	Methicillin-resistant *Staphylococcus aureus*	0.5	32		4	4	16	0.5	0.5	≤0.06	0.25	8	8	32	0.25		0.06	4		0.12		0.25	0.12		0.06
196	Methicillin-resistant *Staphylococcus aureus*	0.5	32		32	>64	32	16	0.5	≤0.06	0.25	>64	>32	>64	>8		4	2		0.25		0.25	0.25		0.06
197	Methicillin-resistant *Staphylococcus aureus*	0.5	32		8	16	32	1	0.5	≤0.06	0.5	32	32	32	0.12		0.015	2		0.12		0.25	0.12		0.06
198	Methicillin-resistant *Staphylococcus aureus*	2	32		8	32	32	2	0.5	≤0.06	0.25	64	32	64	4		1	2		32		0.25	0.12		0.06
199	Methicillin-resistant *Staphylococcus aureus*	1	16		8	16	8	2	0.5	≤0.06	0.25	32	16	64	0.12		0.03	2		0.12		16	0.25		2
200	Methicillin-resistant *Staphylococcus aureus*	0.5	32		4	4	32	0.5	0.5	≤0.06	0.5	4	8	32	0.25		0.06	2		0.12		0.25	0.12		0.06
201	Methicillin-resistant *Staphylococcus aureus*	0.5	32		8	32	16	4	0.5	≤0.06	0.25	32	32	64	0.25		0.06	4		0.25		0.25	0.12		0.06
202	Methicillin-resistant *Staphylococcus aureus*	1	8		8	32	4	4	0.5	≤0.06	0.25	>64	>32	64	>8		4	2		>32		0.25	0.12		0.06
203	Methicillin-resistant *Staphylococcus aureus*	0.5	32		2	4	16	0.25	2	≤0.06	0.5	2	8	32	>8		4	4		>32		0.25	0.25		0.12
204	Methicillin-resistant *Staphylococcus aureus*	0.5	16		4	4	16	0.25	0.5	≤0.06	0.25	8	32	16	>8		2	2		>32		0.25	0.12		0.12
205	Methicillin-resistant *Staphylococcus aureus*	1	8		4	4	8	2	1	≤0.06	1	16	16	64	4		2	4		4		0.5	0.12		32
206	Methicillin-resistant *Staphylococcus aureus*	0.25	16		4	8	8	2	1	≤0.06	1	16	32	64	4		1	4		4		0.5	0.25		>32
207	Methicillin-resistant *Staphylococcus aureus*	0.5	16		16	32	16	4	0.5	≤0.06	0.25	>64	>32	64	>8		4	2		>32		0.25	0.25		0.12
208	Methicillin-resistant *Staphylococcus aureus*	0.5	32		2	32	32	0.25	1	≤0.06	0.5	2	8	32	>8		4	2		>32		0.5	0.25		0.12
209	Methicillin-resistant *Staphylococcus aureus*	1	0.06		0.12	1	0.25	2	0.5	0.12	0.25	16	16	64	>8		2	2		>32		0.25	0.12		0.12
210	Methicillin-resistant *Staphylococcus aureus*	0.5	16		16	32	16	4	1	≤0.06	0.5	>64	>32	64	>8		4	2		0.25		0.25	0.25		0.06
211	Methicillin-resistant *Staphylococcus aureus*	0.5	64		16	32	32	4	1	≤0.06	1	>64	>32	64	>8		8	2		>32		0.25	0.25		0.06
212	Methicillin-resistant *Staphylococcus aureus*	1	64		16	64	32	2	1	≤0.06	0.5	>64	32	64	8		2	2		32		0.25	0.25		0.12
213	Methicillin-resistant *Staphylococcus aureus*	0.5	16		4	8	8	2	1	≤0.06	0.5	16	32	64	>8		4	2		0.12		0.25	0.12		0.12
214	Methicillin-resistant *Staphylococcus aureus*	4	8		8	16	16	8	1	0.12	1	64	>32	64	>8		2	2		0.25		0.25	0.25		0.06
215	Methicillin-resistant *Staphylococcus aureus*	0.5	>64		>32	>64	>32	>16	2	≤0.06	4	>64	>32	>64	>8		8	1		>32		32	0.12		0.5
216	Methicillin-resistant *Staphylococcus aureus*	0.5	32		8	32	32	2	1	≤0.06	0.5	16	32	32	0.12		0.03	1		0.12		0.25	0.12		0.06
217	Methicillin-resistant *Staphylococcus aureus*	0.25	16		4	8	16	1	1	≤0.06	0.25	8	16	32	8		4	1		>32		0.12	0.06		0.06
218	Methicillin-resistant *Staphylococcus aureus*	0.5	32		32	>64	32	>16	1	≤0.06	0.25	>64	>32	>64	>8		4	1		>32		0.12	0.12		0.12
219	Methicillin-resistant *Staphylococcus aureus*	0.5	32		8	8	32	2	1	0.12	0.25	16	32	32	8		2	2		0.25		0.25	0.12		0.06
220	Methicillin-resistant *Staphylococcus aureus*	0.5	0.5		2	8	2	0.5	0.5	≤0.06	1	8	16	64	>8		2	2		>32		0.25	0.12		0.06
221	Methicillin-resistant *Staphylococcus aureus*	1	1		2	8	2	0.5	0.5	≤0.06	0.25	8	16	64	8		2	2		>32		0.25	0.12		0.12
222	Methicillin-resistant *Staphylococcus aureus*	0.5	16		16	32	8	4	0.5	≤0.06	0.5	>64	>32	64	>8		4	2		0.12		4	0.06		0.12
223	Methicillin-resistant *Staphylococcus aureus*	1	64		8	8	>32	0.5	1	≤0.06	0.5	4	16	32	8		8	1		>32		0.12	0.06		0.12
224	Methicillin-resistant *Staphylococcus aureus*	0.5	64		4	4	32	0.5	0.5	≤0.06	0.25	8	16	32	>8		2	2		>32		0.25	0.12		0.06
225	Methicillin-resistant *Staphylococcus aureus*	0.5	2		8	16	4	1	0.5	≤0.06	0.5	32	32	64	4		1	2		>32		0.25	0.12		0.06
226	Methicillin-resistant *Staphylococcus aureus*	0.5	1		1	4	1	0.5	0.25	≤0.06	0.25	8	16	32	8		2	2		>32		0.25	0.12		0.12
227	Methicillin-resistant *Staphylococcus aureus*	0.25	32		1	1	32	0.06	1	0.12	0.5	2	4	8	8		2	2		>32		0.25	0.12		0.12
228	Methicillin-resistant *Staphylococcus aureus*	1	16		16	64	8	8	1	0.12	1	>64	>32	>64	>8		4	2		>32		0.25	0.12		0.06
229	Methicillin-resistant *Staphylococcus aureus*	0.25	8		16	64	8	4	1	≤0.06	1	>64	>32	>64	>8		4	2		0.25		0.25	0.12		0.06
230	Methicillin-resistant *Staphylococcus aureus*	0.25	4		1	4	4	0.25	1	≤0.06	0.5	8	16	32	0.25		0.03	2		0.25		0.25	0.06		0.06
231	Methicillin-resistant *Staphylococcus aureus*	0.25	2		4	8	2	1	1	≤0.06	1	16	32	64	8		4	2		>32		0.25	0.12		0.12
232	Methicillin-resistant *Staphylococcus aureus*	0.25	64		16	64	>32	8	1	≤0.06	2	>64	>32	>64	8		2	2		>32		0.25	0.06		0.06
233	Methicillin-resistant *Staphylococcus aureus*	0.5	8		4	8	8	1	1	0.12	1	16	16	64	>8		4	2		0.25		0.25	0.06		0.12
234	Methicillin-resistant *Staphylococcus aureus*	0.25	16		8	16	8	2	1	≤0.06	1	32	>32	64	>8		4	2		0.25		0.12	0.03		0.06
235	Methicillin-resistant *Staphylococcus aureus*	0.25	16		8	16	16	1	2	≤0.06	1	64	32	32	1		0.25	2		0.25		0.25	0.12		0.06

Isolate Number	*Staphylococcus* species	MIC (µg/ml) Values	β Lactams					Carbapenem	Glycopeptides			Cephalosporins			Flouroquinolones			Oxazolidnone	Macrolides			Tetracyclines		Aminoglycoside	Antifolate
		XF-73	Penicillin	Oxacillin	Amoxicillin Clavulanate	Piperacillin/Tazobactam	Ampicillin	Meropenem	Vancomycin	Dalbavancin	Teicoplanin	Cefuroxime	Ceftriaxone	Ceftazidime	Levofloxacin	Ciprofloxacin	Moxifloxacin	Linezolid	Clindamycin	Clarithromycin	Erythromycin	Tetracycline	Tigecycline	Gentamicin	Trimethoprim/ Sulfmethoxazole
236	Methicillin-resistant *Staphylococcus aureus*	0.5	16		8	16	16	4	1	≤0.06	1	32	>32	64	8		2	2		0.25		0.25	0.12		0.06
237	Methicillin-resistant *Staphylococcus aureus*	2	8		2	4	8	1	2	≤0.06	1	8	16	64	4		1	2		4		0.25	0.12		1
238	Methicillin-resistant *Staphylococcus aureus*	0.5	4		1	4	2	0.5	1	≤0.06	0.5	4	8	32	1		0.25	1		>32		64	0.25		0.06
239	Methicillin-resistant *Staphylococcus aureus*	0.5	32		8	16	32	4	1	≤0.06	0.5	64	32	64	0.25		0.06	4		>32		32	0.25		0.12
240	Methicillin-resistant *Staphylococcus aureus*	0.5	4		2	4	4	0.5	1	≤0.06	0.5	4	8	16	0.25		0.03	2		0.25		0.25	0.12		0.12
241	Methicillin-resistant *Staphylococcus aureus*	0.5	8		4	8	8	1	0.5	≤0.06	0.5	8	16	64	0.12		0.03	2		0.25		16	0.5		0.12
242	Methicillin-resistant *Staphylococcus aureus*	0.25	>64		>32	>64	>32	16	1	≤0.06	1	>64	>32	>64	8		2	2		>32		32	0.5		4
243	Methicillin-resistant *Staphylococcus aureus*	0.25	64		>32	>64	>32	16	1	≤0.06	1	>64	>32	>64	8		4	4		>32		64	0.25		4
244	Methicillin-resistant *Staphylococcus aureus*	0.25	16		16	64	16	8	1	≤0.06	0.5	>64	>32	>64	>8		4	2		>32		64	0.5		0.12
245	Methicillin-resistant *Staphylococcus aureus*	0.25	32		16	64	32	8	0.5	0.12	0.25	>64	>32	>64	>8		4	2		>32		32	0.5		0.12
246	Methicillin-resistant *Staphylococcus aureus*	0.5	0.5		2	4	1	0.5	0.5	≤0.06	0.25	8	16	32	0.12		0.03	2		0.25		0.25	0.12		0.12
247	Methicillin-resistant *Staphylococcus aureus*	0.5	8		2	4	8	0.5	1	≤0.06	0.5	8	16	32	0.25		0.06	2		0.25		16	0.12		0.12
248	Methicillin-resistant *Staphylococcus aureus*	1	16		8	32	16	4	1	0.25	0.5	16	>32	64	>8		4	2		>32		0.25	0.12		0.12
249	Methicillin-resistant *Staphylococcus aureus*	0.5	1		1	8	1	0.5	1	0.12	0.5	2	32	32	0.12		0.03	2		>32		0.25	0.12		0.12
250	Methicillin-resistant *Staphylococcus aureus*	0.5	32		16	64	16	8	0.5	≤0.06	0.25	>64	>32	>64	8		2	2		>32		2	1		0.06
251	Methicillin-resistant *Staphylococcus aureus*	0.5	32		16	64	32	16	0.5	≤0.06	0.5	>64	>32	>64	>8		8	2		>32		0.25	0.25		0.06
252	Methicillin-resistant *Staphylococcus aureus*	0.5	64		32	64	32	16	0.5	≤0.06	0.25	>64	>32	>64	>8		8	2		0.12		0.5	0.5		0.06
253	Methicillin-resistant *Staphylococcus aureus*	0.5	32		16	64	32	4	0.5	0.12	0.25	>64	32	>64	>8		8	2		0.12		0.12	0.12		0.06
254	Methicillin-resistant *Staphylococcus aureus*	0.5	64		16	64	32	4	0.5	0.12	1	>64	>32	>64	>8		8	2		>32		0.25	0.25		0.12
255	Methicillin-resistant *Staphylococcus aureus*	0.5	32		16	32	32	16	1	≤0.06	0.5	>64	>32	>64	>8		8	4		>32		0.5	0.5		0.06
256	Methicillin-resistant *Staphylococcus aureus*	0.25	32		8	64	32	0.5	1	≤0.06	0.5	16	32	64	0.12		0.03	4		>32		0.12	0.12		0.12
257	Methicillin-resistant *Staphylococcus aureus*	0.25	64		32	>64	32	16	0.5	≤0.06	0.5	>64	>32	>64	>8		8	2		>32		0.5	0.25		0.06
258	Methicillin-resistant *Staphylococcus aureus*	0.25	64		32	64	32	16	0.5	≤0.06	0.5	>64	>32	>64	>8		8	2		>32		0.25	0.25		0.06
259	Methicillin-resistant *Staphylococcus aureus*	0.5	32		16	64	32	4	1	≤0.06	0.5	>64	>32	64	>8		8	2		>32		0.5	0.5		0.12
260	Methicillin-resistant *Staphylococcus aureus*	0.5	32		32	>64	32	4	0.5	≤0.06	0.25	>64	>32	>64	>8		8	2		>32		0.12	0.12		0.06
261	Methicillin-resistant *Staphylococcus aureus*	2	32		8	64	32	8	0.5	≤0.06	0.25	>64	>32	>64	8		2	4		>32		0.25	0.25		0.06
262	Methicillin-resistant *Staphylococcus aureus*	0.5	32		4	32	32	4	1	0.12	0.25	>64	>32	>64	8		2	2		>32		0.25	0.12		0.06
263	Methicillin-resistant *Staphylococcus aureus*	0.25	32		8	64	32	8	0.5	≤0.06	0.5	>64	>32	>64	>8		4	2		0.25		0.25	0.25		0.06
264	Methicillin-resistant *Staphylococcus aureus*	0.5	32		8	32	32	4	0.5	≤0.06	0.25	>64	>32	>64	8		1	2		>32		1	0.25		0.06
265	Methicillin-resistant *Staphylococcus aureus*	0.5	32		4	32	32	2	0.5	≤0.06	0.5	>64	>32	64	8		2	2		>32		0.25	0.12		0.06
266	Methicillin-resistant *Staphylococcus aureus*	0.5	32		4	32	32	4	0.5	≤0.06	0.25	>64	>32	>64	8		2	2		>32		0.25	0.12		0.06
267	Methicillin-resistant *Staphylococcus aureus*	0.5	16		4	8	8	2	0.5	≤0.06	0.25	16	32	64	0.12		0.03	2		0.25		0.25	0.12		0.06
268	Methicillin-resistant *Staphylococcus aureus*	0.5	32		8	64	32	8	0.5	0.12	0.25	>64	>32	>64	8		2	1		0.25		0.25	0.06		0.06
269	Methicillin-resistant *Staphylococcus aureus*	0.5	32		8	64	32	8	0.5	≤0.06	0.25	>64	>32	>64	8		4	2		0.25		1	0.12		0.06
270	Methicillin-resistant *Staphylococcus aureus*	0.5	16		8	8	16	2	1	≤0.06	0.5	16	16	64	>8		4	2		>32		0.25	0.25		0.12
271	Methicillin-resistant *Staphylococcus aureus*	0.5	32		32	>64	32	16	0.5	≤0.06	0.5	>64	>32	>64	4		1	2		0.12		32	0.12		2
272	Methicillin-resistant *Staphylococcus aureus*	0.5	32		>32	>64	32	>16	1	0.12	2	>64	>32	>64	>8		8	2		>32		0.25	0.12		0.25
273	Methicillin-resistant *Staphylococcus aureus*	1	1		4	8	1	1	1	0.25	0.5	4	32	64	>8		4	2		0.12		0.12	0.12		0.12
274	Methicillin-resistant *Staphylococcus aureus*	0.25	64		32	>64	32	16	1	≤0.06	0.5	>64	>32	>64	4		1	2		>32		0.25	0.25		0.12
275	Methicillin-resistant *Staphylococcus aureus*	0.5	16		2	4	8	1	1	≤0.06	0.5	8	16	64	>8		4	2		>32		0.25	0.25		0.12
276	Methicillin-resistant *Staphylococcus aureus*	0.5	64		32	>64	>32	>16	1	0.25	1	>64	>32	>64	8		2	1		>32		32	0.25		0.25
277	Methicillin-resistant *Staphylococcus aureus*	0.5	32		32	>64	32	16	1	0.12	0.5	>64	>32	>64	8		2	1		0.12		64	0.5		0.25
278	Methicillin-resistant *Staphylococcus aureus*	0.5	32		16	32	32	4	1	≤0.06	0.5	>64	>32	>64	0.12		0.06	2		0.12		0.25	0.12		0.25
279	Methicillin-resistant *Staphylococcus aureus*	0.5	32		16	32	32	16	0.5	≤0.06	0.12	>64	>32	>64	>8		4	2		>32		0.25	0.12		0.12
280	Methicillin-resistant *Staphylococcus aureus*	0.25	16		8	16	8	>16	2	≤0.06	2	64	>32	64	8		1	1		>32		2	0.25		8
281	Methicillin-resistant *Staphylococcus aureus*	0.5	4		0.5	0.5	2	2	2	≤0.06	2	4	8	16	0.06		0.03	1		>32		32	0.12		4
282	Methicillin-resistant *Staphylococcus aureus*	0.5	16		2	2	16	1	1	0.25	0.5	8	16	16	0.12		0.03	2		32		16	0.12		0.03

Isolate Number	*Staphylococcus* species	MIC (µg/ml) Values																							
			β Lactams					Carbapenem	Glycopeptides			Cephalosporins			Flouroquinolones			Oxazolidnone	Macrolides			Tetracyclines		Aminoglycoside	Antifolate
		XF-73	Penicillin	Oxacillin	Amoxicillin Clavulanate	Piperacillin/Tazobactam	Ampicillin	Meropenem	Vancomycin	Dalbavancin	Teicoplanin	Cefuroxime	Ceftriaxone	Ceftazidime	Levofloxacin	Ciprofloxacin	Moxifloxacin	Linezolid	Clindamycin	Clarithromycin	Erythromycin	Tetracycline	Tigecycline	Gentamicin	Trimethoprim/ Sulfmethoxazole
283	Methicillin-resistant *Staphylococcus aureus*	0.25	2		4	4	4	16	2	≤0.06	1	32	32	64	2		0.5	0.5		>32		1	0.25		8
284	Methicillin-resistant *Staphylococcus aureus*	0.25	0.5		1	2	1	1	1	≤0.06	0.5	2	16	32	0.12		0.03	1		16		>64	0.25		4
285	Methicillin-resistant *Staphylococcus aureus*	0.5	64		32	>64	32	>16	1	≤0.06	1	>64	>32	>64	8		2	1		>32		0.12	0.06		0.12
286	Methicillin-resistant *Staphylococcus aureus*	0.5	16		16	64	16	>16	2	≤0.06	8	>64	>32	>64	8		2	2		>32		0.5	0.06		4
287	Methicillin-resistant *Staphylococcus aureus*	0.5	64		>32	>64	32	>16	1	≤0.06	1	>64	>32	>64	>8		4	2		>32		0.5	0.5		0.12
288	Methicillin-resistant *Staphylococcus aureus*	0.25	32		4	8	32	2	1	≤0.06	0.5	8	32	64	8		2	2		>32		0.25	0.12		0.06
289	Methicillin-resistant *Staphylococcus aureus*	0.25	64		>32	>64	32	>16	1	0.12	1	>64	>32	>64	8		2	2		>32		0.25	0.12		0.12
290	Methicillin-resistant *Staphylococcus aureus*	0.5	32		16	64	32	4	1	≤0.06	0.5	>64	>32	>64	>8		4	2		>32		0.25	0.12		0.06
291	Methicillin-resistant *Staphylococcus aureus*	0.5	64		>32	>64	32	>16	1	≤0.06	2	>64	>32	>64	8		2	2		>32		0.25	0.12		0.12
292	Methicillin-resistant *Staphylococcus aureus*	0.25	64		32	>64	32	>16	1	0.12	1	>64	>32	>64	8		2	2		>32		0.5	0.12		0.12
293	Methicillin-resistant *Staphylococcus aureus*	0.5	64		>32	>64	32	>16	1	0.12	1	>64	>32	>64	8		2	1		>32		0.25	0.12		0.12
294	Methicillin-resistant *Staphylococcus aureus*	0.5	32		>32	>64	32	>16	1	≤0.06	1	>64	>32	>64	8		2	2		>32		0.25	0.12		0.12
295	Methicillin-resistant *Staphylococcus aureus*	0.5	64		>32	>64	32	>16	0.5	≤0.06	1	>64	>32	>64	8		2	2		>32		0.25	0.12		0.12
296	Methicillin-resistant *Staphylococcus aureus*	0.25	16		1	4	2	2	1	≤0.06	0.25	2	8	32	0.06		0.015	1		>32		0.25	0.12		8
297	Methicillin-resistant *Staphylococcus aureus*	0.5	16		1	1	8	4	2	≤0.06	4	8	16	32	8		4	1		>32		1	0.25		0.25
298	Methicillin-resistant *Staphylococcus aureus*	0.5	64		>32	>64	32	>16	1	≤0.06	1	>64	>32	>64	8		2	2		>32		0.25	0.12		0.12
299	Methicillin-resistant *Staphylococcus aureus*	0.5	64		>32	>64	32	>16	1	≤0.06	1	>64	>32	>64	8		2	1		>32		0.25	0.25		0.12
300	Methicillin-resistant *Staphylococcus aureus*	0.5	64		>32	>64	32	>16	1	0.12	1	>64	>32	>64	8		2	2		>32		0.25	0.12		0.12
301	Methicillin-resistant *Staphylococcus aureus*	0.5	64		>32	>64	32	>16	1	≤0.06	1	>64	>32	>64	>8		>8	2		>32		0.25	0.06		0.12
302	Methicillin-resistant *Staphylococcus aureus*	1	64		32	>64	32	16	1	≤0.06	0.5	>64	>32	>64	>8		4	2		>32		0.12	0.06		0.06
303	Methicillin-resistant *Staphylococcus aureus*	1	32		16	64	16	4	1	≤0.06	0.5	>64	>32	64	>8		>8	2		>32		0.25	0.12		0.06
304	Methicillin-resistant *Staphylococcus aureus*	0.5	32		16	64	32	4	1	≤0.06	0.25	>64	>32	>64	>8		8	2		>32		0.25	0.12		0.06
305	Methicillin-resistant *Staphylococcus aureus*	0.25	64		32	>64	32	>16	2	>0.5	0.5	>64	>32	>64	8		2	2		>32		0.12	0.06		0.06
306	Methicillin-resistant *Staphylococcus aureus*	0.5	64		8	16	16	1	1	≤0.06	0.5	64	32	32	0.25		0.03	1		0.25		32	0.12		0.06
307	Methicillin-resistant *Staphylococcus aureus*	0.5	64		32	>64	32	16	1	0.25	0.5	>64	>32	>64	>8		4	2		0.25		0.25	0.06		0.06
308	Methicillin-resistant *Staphylococcus aureus*	0.5	16		8	32	16	4	1	≤0.06	0.5	>64	>32	64	8		4	2		>32		0.12	0.06		0.06
309	Methicillin-resistant *Staphylococcus aureus*	0.5	8		2	8	8	1	2	≤0.06	0.5	2	8	32	4		2	2		0.25		0.25	0.12		0.06
310	Methicillin-resistant *Staphylococcus aureus*	0.5	32		32	>64	32	16	1	≤0.06	0.5	>64	>32	>64	>8		4	2		0.25		0.12	0.06		0.06
311	Methicillin-resistant *Staphylococcus aureus*	0.5	64		32	>64	>32	16	1	≤0.06	2	>64	>32	>64	>8		8	2		2		32	0.06		1
312	Methicillin-resistant *Staphylococcus aureus*	2	16		8	16	16	1	1	≤0.06	0.25	16	32	32	0.25		0.03	2		0.25		0.12	0.12		0.12
313	Methicillin-resistant *Staphylococcus aureus*	0.5	64		>32	>64	>32	16	1	≤0.06	2	>64	>32	>64	>8		4	1		32		64	0.25		0.25
314	Methicillin-resistant *Staphylococcus aureus*	0.5	64		>32	>64	>32	16	1	0.12	1	>64	>32	>64	>8		8	1		>32		64	0.25		0.25
315	Methicillin-resistant *Staphylococcus aureus*	0.5	>64		>32	>64	>32	16	1	≤0.06	2	>64	>32	>64	>8		4	1		16		64	0.25		0.25
316	Methicillin-resistant *Staphylococcus aureus*	0.5	64		32	64	>32	16	1	≤0.06	2	>64	>32	>64	>8		4	1		>32		32	0.12		0.25
317	Methicillin-resistant *Staphylococcus aureus*	0.5	64		32	>64	>32	>16	1	≤0.06	2	>64	>32	>64	>8		4	1		>32		64	0.25		0.25
318	Methicillin-resistant *Staphylococcus aureus*	0.5	32		4	8	32	1	1	≤0.06	0.25	8	16	64	4		1	1		32		0.25	0.12		0.06
319	Methicillin-resistant *Staphylococcus aureus*	0.5	>64		32	>64	>32	16	1	≤0.06	2	>64	>32	>64	>8		4	1		32		64	0.25		0.25
320	Methicillin-resistant *Staphylococcus aureus*	0.5	32		4	8	32	1	1	≤0.06	0.25	8	32	32	4		2	2		32		0.12	0.12		0.12
321	Methicillin-resistant *Staphylococcus aureus*	0.5	2		0.5	4	0.5	2	2	≤0.06	0.5	16	16	>64	1		0.25	4		0.25		0.25	0.25		0.12
322	Methicillin-resistant *Staphylococcus aureus*	0.5	1		0.5	4	0.5	2	2	0.12	0.5	16	16	>64	1		0.25	2		0.25		32	0.25		0.12
323	Methicillin-resistant *Staphylococcus aureus*	0.5	>64		32	64	>32	16	0.5	0.12	0.5	>64	>32	>64	4		1	1		>32		32	0.5		0.25
324	Methicillin-resistant *Staphylococcus aureus*	0.5	64		32	64	>32	16	0.5	≤0.06	0.5	>64	>32	>64	4		2	1		>32		32	1		0.25
325	Methicillin-resistant *Staphylococcus aureus*	0.5	4		2	4	4	0.5	0.5	≤0.06	0.25	8	16	32	0.12		0.03	2		0.12		0.25	0.25		0.06
326	Methicillin-resistant *Staphylococcus aureus*	0.5	8		8	8	8	1	0.5	0.25	0.25	8	16	32	0.12		0.015	2		0.12		0.25	0.12		0.06
327	Methicillin-resistant *Staphylococcus aureus*	0.5	64		32	>64	>32	16	1	0.12	2	>64	>32	>64	8		2	2		>32		64	0.5		0.25
328	Methicillin-resistant *Staphylococcus aureus*	0.5	>64		32	64	>32	16	1	0.25	2	>64	>32	>64	>8		8	2		32		64	0.5		0.25
329	Methicillin-resistant *Staphylococcus aureus*	0.5	64		32	>64	>32	16	1	0.12	2	>64	>32	64	>8		8	2		>32		64	0.5		0.25

Isolate Number	*Staphylococcus* species	MIC (µg/ml) Values																							
			β Lactams					Carbapenem	Glycopeptides			Cephalosporins			Flouroquinolones			Oxazolidnone	Macrolides			Tetracyclines		Aminoglycoside	Antifolate
		XF-73	Penicillin	Oxacillin	Amoxicillin Clavulanate	Piperacillin/Tazobactam	Ampicillin	Meropenem	Vancomycin	Dalbavancin	Teicoplanin	Cefuroxime	Ceftriaxone	Ceftazidime	Levofloxacin	Ciprofloxacin	Moxifloxacin	Linezolid	Clindamycin	Clarithromycin	Erythromycin	Tetracycline	Tigecycline	Gentamicin	Trimethoprim/ Sulfmethoxazole
330	Methicillin-resistant *Staphylococcus aureus*	0.5	64		32	>64	>32	16	1	0.12	2	>64	>32	64	>8		4	1		>32		64	0.5		0.12
331	Methicillin-resistant *Staphylococcus aureus*	0.5	8		4	8	8	1	0.5	0.12	0.5	16	32	64	0.12		0.03	2		>32		32	0.5		0.06
332	Methicillin-resistant *Staphylococcus aureus*	0.5	64		32	>64	32	16	1	0.25	2	>64	>32	>64	>8		8	2		>32		64	0.5		0.25
333	Methicillin-resistant *Staphylococcus aureus*	0.5	8		4	8	8	1	0.5	0.12	0.25	16	16	64	0.25		0.03	2		>32		32	0.5		0.06
334	Methicillin-resistant *Staphylococcus aureus*	0.5	32		16	64	32	8	0.5	0.12	0.25	>64	>32	64	8		1	2		0.12		0.25	0.25		0.06
335	Methicillin-resistant *Staphylococcus aureus*	0.5	8		16	64	8	4	0.5	≤0.06	0.5	>64	>32	>64	0.25		0.06	1		0.06		0.12	0.12		0.12
336	Methicillin-resistant *Staphylococcus aureus*	0.5	32		32	>64	32	16	0.5	≤0.06	0.25	>64	>32	>64	>8		2	2		>32		0.25	0.12		0.06
337	Methicillin-resistant *Staphylococcus aureus*	1	16		16	32	16	4	0.5	≤0.06	0.25	>64	>32	64	>8		4	2		0.12		0.25	0.25		0.06
338	Methicillin-resistant *Staphylococcus aureus*	1	32		16	64	32	8	0.5	≤0.06	0.25	>64	>32	>64	8		1	2		>32		0.25	0.12		0.12
339	Methicillin-resistant *Staphylococcus aureus*	0.5	16		8	32	16	4	0.5	≤0.06	0.25	>64	>32	64	8		2	1		>32		0.25	0.25		0.06
340	Methicillin-resistant *Staphylococcus aureus*	0.5	32		16	64	16	8	0.5	≤0.06	0.25	>64	>32	>64	8		1	2		0.12		0.25	0.06		0.06
341	Methicillin-resistant *Staphylococcus aureus*	0.5	64		8	16	32	1	0.5	≤0.06	0.25	16	16	32	8		1	2		16		64	1		0.06
342	Methicillin-resistant *Staphylococcus aureus*	0.5	32		4	8	32	1	0.5	0.12	0.5	16	16	32	4		1	1		16		32	0.5		0.06
343	Methicillin-resistant *Staphylococcus aureus*	0.5	16		16	64	16	4	0.5	0.12	0.25	>64	>32	>64	4		1	2		>32		32	0.5		2
344	Methicillin-resistant *Staphylococcus aureus*	0.5	2		1	1	2	0.06	1	0.12	1	2	4	8	0.12		0.03	2		0.25		0.25	0.12		0.06
345	Methicillin-resistant *Staphylococcus aureus*	0.5	32		16	64	16	8	0.5	≤0.06	0.25	>64	>32	64	8		2	2		>32		0.25	0.25		0.12
346	Methicillin-resistant *Staphylococcus aureus*	0.5	32		16	64	32	8	0.5	≤0.06	0.25	>64	>32	>64	8		2	1		0.12		0.25	0.12		0.06
347	Methicillin-resistant *Staphylococcus aureus*	0.5	32		16	32	32	4	0.5	≤0.06	0.25	>64	>32	>64	4		1	1		>32		0.25	0.12		0.06
348	Methicillin-resistant *Staphylococcus aureus*	0.5	64		8	16	32	2	0.5	≤0.06	0.5	16	32	32	0.25		0.06	2		>32		0.25	0.12		0.5
349	Methicillin-resistant *Staphylococcus aureus*	1	32		16	64	32	8	0.5	≤0.06	0.25	>64	>32	>64	8		2	2		>32		0.25	0.12		0.25
350	Methicillin-resistant *Staphylococcus aureus*	0.5	2		8	8	2	0.5	1	≤0.06	0.5	16	32	32	1		0.12	2		>32		0.25	0.12		0.12
351	Methicillin-resistant *Staphylococcus aureus*	0.5	32		16	64	32	16	0.5	0.12	0.5	>64	>32	>64	>8		8	2		>32		0.5	0.06		0.06
352	Methicillin-resistant *Staphylococcus aureus*	0.5	32		16	>64	16	>16	1	0.12	0.5	>64	>32	>64	>8		4	2		>32		0.5	0.06		0.06
353	Methicillin-resistant *Staphylococcus aureus*	0.5	64		32	>64	>32	>16	1	0.12	1	>64	>32	>64	>8		4	2		>32		0.25	0.12		0.12
354	Methicillin-resistant *Staphylococcus aureus*	0.5	64		>32	>64	>32	>16	1	0.12	1	>64	>32	>64	>8		4	2		>32		0.25	0.12		0.12
355	Methicillin-resistant *Staphylococcus aureus*	0.5	16		4	16	16	1	1	≤0.06	0.5	8	16	32	1		0.12	2		>32		0.5	0.12		0.12
356	Methicillin-resistant *Staphylococcus aureus*	0.5	>64		16	64	>32	>16	1	≤0.06	0.5	>64	>32	>64	>8		4	4		0.25		0.5	0.12		0.06
357	Methicillin-resistant *Staphylococcus aureus*	0.5	64		>32	>64	>32	>16	1	0.12	2	>64	>32	>64	8		2	2		>32		0.5	0.12		0.25
358	Methicillin-resistant *Staphylococcus aureus*	0.5	64		32	>64	>32	>16	1	0.12	1	>64	>32	>64	>8		4	4		>32		0.5	0.25		0.06
359	Methicillin-resistant *Staphylococcus aureus*	0.5	>64		4	16	>32	1	1	0.12	0.5	64	32	64	>8		4	2		0.25		64	0.12		0.25
360	Methicillin-resistant *Staphylococcus aureus*	0.5	64		8	32	>32	1	1	≤0.06	0.25	64	32	64	4		2	2		>32		0.25	0.12		0.06
361	Methicillin-resistant *Staphylococcus aureus*	0.5	64		32	>64	32	16	0.5	≤0.06	0.5	>64	>32	>64	>8		8	4		0.25		0.25	0.12		0.06
362	Methicillin-resistant *Staphylococcus aureus*	0.5	≤0.03		0.12	1	0.25	0.12	0.5	0.25	0.5	2	2	16	0.25		0.03	2		0.12		0.06	0.12		0.03
363	Methicillin-resistant *Staphylococcus aureus*	0.5	32		1	2	16	0.12	1	0.12	0.5	4	4	16	0.25		0.06	0.5		0.12		0.25	0.12		0.06
364	Methicillin-resistant *Staphylococcus aureus*	0.5	1		0.5	2	1	0.12	1	0.12	0.5	2	8	16	0.25		0.06	2		>32		0.25	0.12		0.06
365	Methicillin-resistant *Staphylococcus aureus*	1	8		8	32	16	4	1	≤0.06	0.5	>64	>32	>64	>8		8	2		>32		0.25	0.12		0.03
366	Methicillin-resistant *Staphylococcus aureus*	0.5	16		8	16	16	4	1	≤0.06	0.5	>64	32	>64	>8		8	2		>32		0.25	0.12		0.06
367	Methicillin-resistant *Staphylococcus aureus*	1	64		4	16	>32	1	1	≤0.06	1	>64	16	64	0.25		0.06	2		32		0.06	0.12		0.03
368	Methicillin-resistant *Staphylococcus aureus*	0.5	32		16	>64	32	16	2	≤0.06	0.5	>64	>32	>64	>8		8	2		>32		0.06	0.12		0.06
369	Methicillin-resistant *Staphylococcus aureus*	0.5	64		4	8	>32	1	1	0.12	0.5	16	16	64	8		2	2		32		0.06	0.12		0.06
370	Methicillin-resistant *Staphylococcus aureus*	1	64		4	16	16	4	1	≤0.06	0.5	32	32	64	>8		>8	2		>32		0.25	0.12		0.06
371	Methicillin-resistant *Staphylococcus aureus*	1	32		4	8	16	2	0.5	≤0.06	0.25	16	16	64	4		1	1		32		0.06	0.12		0.06
372	Methicillin-resistant *Staphylococcus aureus*	1	64		8	16	>32	2	1	≤0.06	0.5	32	>32	64	4		2	2		32		0.06	0.12		0.06
373	Methicillin-resistant *Staphylococcus aureus*	0.5	16		8	32	8	8	1	0.12	1	>64	>32	>64	>8		8	2		>32		0.25	0.12		0.03
374	Methicillin-resistant *Staphylococcus aureus*	0.5	16		8	16	16	4	1	0.25	0.5	>64	32	>64	>8		8	2		>32		0.25	0.12		0.06
375	Methicillin-resistant *Staphylococcus aureus*	0.5	16		8	32	32	4	1	0.12	0.5	>64	32	64	>8		8	2		>32		0.5	0.12		0.06
376	Methicillin-resistant *Staphylococcus aureus*	0.5	64		16	>64	>32	16	2	0.25	1	>64	>32	>64	>8		2	2		32		0.25	0.12		0.25

Isolate Number	*Staphylococcus* species	MIC (µg/ml) Values																							
			β Lactams					Carbapenem	Glycopeptides			Cephalosporins			Flouroquinolones			Oxazolidnone	Macrolides			Tetracyclines		Aminoglycoside	Antifolate
		XF-73	Penicillin	Oxacillin	Amoxicillin Clavulanate	Piperacillin/Tazobactam	Ampicillin	Meropenem	Vancomycin	Dalbavancin	Teicoplanin	Cefuroxime	Ceftriaxone	Ceftazidime	Levofloxacin	Ciprofloxacin	Moxifloxacin	Linezolid	Clindamycin	Clarithromycin	Erythromycin	Tetracycline	Tigecycline	Gentamicin	Trimethoprim/ Sulfmethoxazole
377	Methicillin-resistant *Staphylococcus aureus*	1	64		8	32	>32	1	1	0.12	0.5	64	>32	16	8		2	2		32		0.25	0.12		0.06
378	Methicillin-resistant *Staphylococcus aureus*	0.5	16		16	32	16	4	1	0.12	0.5	>64	>32	>64	>8		>8	4		>32		0.5	0.12		0.12
379	Methicillin-resistant *Staphylococcus aureus*	0.5	32		32	64	32	4	1	0.12	1	>64	>32	>64	>8		>8	4		>32		0.25	0.12		0.12
380	Methicillin-resistant *Staphylococcus aureus*	0.5	64		8	32	>32	2	1	0.12	1	64	>32	>64	8		2	2		>32		0.25	0.12		0.12
381	Methicillin-resistant *Staphylococcus aureus*	0.5	64		8	32	>32	2	1	0.12	0.5	64	>32	64	4		2	2		32		0.12	0.06		0.06
382	Methicillin-resistant *Staphylococcus aureus*	0.5	16		32	>64	16	16	1	0.12	0.5	>64	>32	>64	>8		>8	2		>32		0.25	0.12		0.12
383	Methicillin-resistant *Staphylococcus aureus*	0.5	16		8	16	16	2	1	≤0.06	4	8	16	16	>8		>8	2		>32		0.12	0.25		0.12
384	Methicillin-resistant *Staphylococcus aureus*	0.5	32		8	32	32	2	1	≤0.06	0.5	16	32	32	0.25		0.03	2		>32		16	0.25		0.12
385	Methicillin-resistant *Staphylococcus aureus*	1	32		8	32	32	2	1	≤0.06	0.5	>64	32	>64	0.12		0.03	2		0.25		0.25	0.12		0.12
386	Methicillin-resistant *Staphylococcus aureus*	0.5	32		8	32	32	2	1	0.12	0.5	32	32	64	0.25		0.03	2		0.25		0.25	0.25		0.12
387	Methicillin-resistant *Staphylococcus aureus*	0.5	32		16	64	16	16	1	≤0.06	0.5	>64	>32	>64	>8		8	2		>32		0.25	0.12		0.06
388	Methicillin-resistant *Staphylococcus aureus*	0.5	32		8	64	32	2	1	≤0.06	0.5	64	32	64	0.25		0.03	2		0.06		0.25	0.12		0.12
389	Methicillin-resistant *Staphylococcus aureus*	0.5	32		8	32	32	2	1	0.12	1	32	32	64	4		1	2		32		0.25	0.12		0.5
390	Methicillin-resistant *Staphylococcus aureus*	4	64		8	32	>32	2	1	≤0.06	0.5	64	16	64	4		1	0.5		32		0.25	0.12		0.5
391	Methicillin-resistant *Staphylococcus aureus*	0.5	32		8	>64	32	4	1	≤0.06	0.5	16	32	64	0.25		0.03	2		0.25		0.25	0.25		0.12
392	Methicillin-resistant *Staphylococcus aureus*	0.5	64		8	16	32	2	1	≤0.06	0.5	16	32	32	4		1	2		>32		0.25	0.12		0.12
393	Methicillin-resistant *Staphylococcus aureus*	0.5	4		8	32	2	0.25	0.5	≤0.06	0.5	32	8	2	0.25		0.03	4		0.25		0.25	0.12		0.12
394	Methicillin-resistant *Staphylococcus aureus*	0.5	32		16	64	32	0.25	0.5	0.5	0.25	>64	8	2	>8		4	2		>32		2	0.5		0.12
395	Methicillin-resistant *Staphylococcus aureus*	0.5	64		8	32	32	2	0.5	0.12	0.12	32	32	64	4		1	0.06		32		0.25	0.12		0.06
396	Methicillin-resistant *Staphylococcus aureus*	0.5	32		8	16	32	1	0.5	≤0.06	0.25	16	16	32	4		2	1		0.12		0.25	0.06		0.06
397	Methicillin-resistant *Staphylococcus aureus*	0.5	>64		16	>64	>32	2	0.5	≤0.06	0.5	>64	32	>64	>8		2	2		32		0.5	0.25		0.12
398	Methicillin-resistant *Staphylococcus aureus*	0.5	64		16	64	>32	1	1	≤0.06	0.5	>64	32	>64	4		1	2		32		0.25	0.25		0.06
399	Methicillin-resistant *Staphylococcus aureus*	0.5	32		16	32	32	4	1	0.25	0.5	>64	32	>64	4		1	2		>32		0.25	0.25		0.06
400	Methicillin-resistant *Staphylococcus aureus*	0.5	32		16	32	16	>16	1	≤0.06	0.5	>64	32	>64	>8		8	2		>32		0.5	0.5		0.06
401	Methicillin-resistant *Staphylococcus aureus*	0.5	>64		8	32	>32	4	1	≤0.06	0.5	>64	>32	>64	8		2	2		>32		0.5	1		0.12
402	Methicillin-resistant *Staphylococcus aureus*	0.5	>64		16	64	>32	2	1	0.25	0.5	64	32	64	8		2	4		32		0.5	0.5		0.12
403	Methicillin-resistant *Staphylococcus aureus*	1	>64		16	64	>32	8	1	0.25	0.5	>64	>32	>64	8		2	4		>32		4	1		4
404	Methicillin-resistant *Staphylococcus aureus*	0.5	>64		16	64	>32	4	1	0.25	0.5	>64	32	64	4		2	4		>32		16	0.5		0.5
405	Methicillin-resistant *Staphylococcus aureus*	1	>64		16	64	>32	8	1	0.25	0.5	>64	32	>64	8		>8	2		>32		64	1		0.12
406	Methicillin-resistant *Staphylococcus aureus*	0.5	32		2	2	32	1	1	≤0.06	1	4	32	32	0.25		0.03	2		0.25		0.25	0.06		0.06
407	Methicillin-resistant *Staphylococcus aureus*	0.5	>64		>32	>64	>32	>16	2	≤0.06	1	>64	>32	>64	8		2	2		>32		64	0.06		8
408	Methicillin-resistant *Staphylococcus aureus*	1	>64		>32	>64	>32	>16	2	≤0.06	1	>64	>32	>64	8		2	2		>32		64	0.12		32
409	Methicillin-resistant *Staphylococcus aureus*	0.5	64		8	32	>32	4	1	0.5	0.5	>64	>32	>64	0.25		0.06	2		0.25		0.25	0.12		0.12
410	Methicillin-resistant *Staphylococcus aureus*	0.5	>64		>32	>64	>32	>16	2	≤0.06	1	>64	>32	>64	8		2	1		>32		64	0.12		32
411	Methicillin-resistant *Staphylococcus aureus*	0.5	32		8	16	32	2	1	0.12	0.5	16	32	64	0.25		0.03	2		0.25		0.25	0.12		0.06
412	Methicillin-resistant *Staphylococcus aureus*	1	64		8	16	>32	4	1	≤0.06	1	32	32	64	0.25		0.06	2		0.25		0.25	0.12		0.12
413	Methicillin-resistant *Staphylococcus aureus*	1	32		8	16	32	2	1	0.25	0.5	16	32	64	0.25		0.03	4		0.25		0.5	0.12		0.06
414	Methicillin-resistant *Staphylococcus aureus*	0.5	64		16	64	>32	4	1	≤0.06	1	>64	>32	>64	0.12		0.03	2		>32		0.25	0.06		0.06
415	Methicillin-resistant *Staphylococcus aureus*	0.5	32		32	>64	32	>16	1	≤0.06	1	>64	>32	>64	8		2	1		>32		64	0.06		8
416	Methicillin-resistant *Staphylococcus aureus*	0.5	64		16	64	>32	8	1	0.12	1	>64	>32	>64	0.12		0.03	2		>32		16	0.06		0.06
417	Methicillin-resistant *Staphylococcus aureus*	0.5	16		4	8	16	1	1	≤0.06	0.25	8	16	32	0.25		0.06	2		0.25		0.5	0.25		0.12
418	Methicillin-resistant *Staphylococcus aureus*	0.5	32		16	64	32	8	0.5	≤0.06	0.25	>64	>32	>64	>8		2	4		>32		0.5	0.5		0.12
419	Methicillin-resistant *Staphylococcus aureus*	0.5	32		16	64	32	8	0.5	≤0.06	0.25	>64	>32	>64	8		2	4		0.25		1	0.5		2
420	Methicillin-resistant *Staphylococcus aureus*	0.5	64		32	>64	>32	>16	1	≤0.06	0.5	>64	>32	>64	8		>8	2		>32		32	0.25		16
421	Methicillin-resistant *Staphylococcus aureus*	1	32		4	8	32	2	1	≤0.06	0.5	8	32	64	0.25		0.06	4		0.25		0.5	0.5		0.12
422	Methicillin-resistant *Staphylococcus aureus*	1	32		4	8	32	1	1	≤0.06	2	16	32	32	0.25		0.03	4		0.25		0.5	0.25		0.12
423	Methicillin-resistant *Staphylococcus aureus*	1	32		16	64	32	8	0.5	0.25	0.5	>64	>32	>64	>8		8	4		>32		2	1		0.06

Isolate Number	*Staphylococcus* species	MIC (µg/ml) Values																							
			β Lactams					Carbapenem	Glycopeptides			Cephalosporins			Flouroquinolones			Oxazolidnone	Macrolides			Tetracyclines		Aminoglycoside	Antifolate
		XF-73	Penicillin	Oxacillin	Amoxicillin Clavulanate	Piperacillin/Tazobactam	Ampicillin	Meropenem	Vancomycin	Dalbavancin	Teicoplanin	Cefuroxime	Ceftriaxone	Ceftazidime	Levofloxacin	Ciprofloxacin	Moxifloxacin	Linezolid	Clindamycin	Clarithromycin	Erythromycin	Tetracycline	Tigecycline	Gentamicin	Trimethoprim/ Sulfmethoxazole
424	Methicillin-resistant *Staphylococcus aureus*	0.5	8		4	4	8	0.5	1	0.12	0.5	4	8	16	0.12		0.06	4		0.25		0.5	0.12		0.12
425	Methicillin-resistant *Staphylococcus aureus*	0.5	16		2	4	16	1	0.5	0.25	0.5	4	8	32	8		4	2		0.25		0.25	0.12		0.12
426	Methicillin-resistant *Staphylococcus aureus*	0.5	8		8	64	8	8	0.5	0.12	0.25	>64	>32	>64	0.25		0.06	4		0.25		0.5	0.25		0.25
427	Methicillin-resistant *Staphylococcus aureus*	0.5	64		16	64	16	8	0.5	0.12	0.5	>64	>32	>64	>8		2	2		>32		0.5	0.25		0.06
428	Methicillin-resistant *Staphylococcus aureus*	0.5	64		8	16	>32	2	0.5	0.25	0.12	64	32	32	0.12		0.06	2		0.25		0.25	0.25		0.06
429	Methicillin-resistant *Staphylococcus aureus*	0.5	32		4	8	32	0.5	0.5	0.25	0.25	4	32	32	0.25		0.03	2		0.25		0.25	0.12		0.06
430	Methicillin-resistant *Staphylococcus aureus*	0.5	16		4	8	16	2	1	0.12	0.5	16	32	32	0.25		0.06	2		>32		0.25	0.25		0.06
431	Methicillin-resistant *Staphylococcus aureus*	0.5	8		8	8	8	4	1	>0.5	0.25	32	32	64	0.25		0.03	2		32		0.25	0.12		0.06
432	Methicillin-resistant *Staphylococcus aureus*	0.5	32		8	16	32	1	1	0.12	0.5	16	16	32	0.12		0.03	2		0.25		0.25	0.25		0.12
433	Methicillin-resistant *Staphylococcus aureus*	0.5	32		4	8	32	1	1	≤0.06	0.25	8	16	32	0.25		0.03	2		0.25		0.5	0.25		0.06
434	Methicillin-resistant *Staphylococcus aureus*	0.5	64		4	8	>32	2	1	0.25	0.5	32	32	64	0.25		0.03	2		0.25		0.25	0.25		0.06
435	Methicillin-resistant *Staphylococcus aureus*	0.5	64		8	32	>32	1	1	≤0.06	0.5	16	32	64	4		1	2		0.25		0.25	0.12		0.06
436	Methicillin-resistant *Staphylococcus aureus*	0.5	64		8	16	>32	2	0.5	0.25	0.25	16	32	64	0.25		0.03	2		0.25		0.25	0.25		0.06
437	Methicillin-resistant *Staphylococcus aureus*	0.5	64		8	32	>32	4	0.5	0.25	0.25	64	32	64	0.25		0.03	2		0.25		0.25	0.25		0.06
438	Methicillin-resistant *Staphylococcus aureus*	0.5	32		32	64	32	16	1	0.25	2	>64	>32	64	>8		>8	2		>32		64	4		0.06
439	Methicillin-resistant *Staphylococcus aureus*	0.5	8		1	2	8	0.5	0.5	0.25	0.5	4	8	16	>8		4	4		>32		0.25	0.12		0.5
440	Methicillin-resistant *Staphylococcus aureus*	1	8		1	2	4	0.5	0.5	0.12	0.25	4	8	32	>8		4	2		>32		1	0.25		0.25
441	Methicillin-resistant *Staphylococcus aureus*	0.5	64		4	4	>32	2	0.5	0.25	0.5	16	16	32	>8		4	2		0.25		0.5	0.5		0.06
442	Methicillin-resistant *Staphylococcus aureus*	0.5	4		8	16	4	2	0.5	≤0.06	0.5	64	32	64	0.25		0.06	2		0.25		0.25	0.25		0.12
443	Methicillin-resistant *Staphylococcus aureus*	0.5	32		1	4	16	0.5	0.5	0.25	0.5	16	8	16	>8		4	2		>32		32	0.25		0.12
444	Methicillin-resistant *Staphylococcus aureus*	1	>64		8	32	>32	2	2	0.12	0.5	16	16	32	>8		4	2		0.25		0.25	0.25		0.5
445	Methicillin-resistant *Staphylococcus aureus*	0.5	32		4	16	32	1	1	0.12	0.5	64	32	64	8		1	1		0.12		32	0.12		0.5
446	Methicillin-resistant *Staphylococcus aureus*	0.5	16		4	8	8	0.5	1	0.12	0.5	16	16	32	0.25		0.03	2		>32		0.25	0.12		0.12
447	Methicillin-resistant *Staphylococcus aureus*	0.5	>64		>32	>64	>32	>16	1	0.12	1	>64	>32	>64	>8		4	2		>32		>64	0.5		0.5
448	Methicillin-resistant *Staphylococcus aureus*	1	64		16	64	>32	>16	1	0.25	2	>64	>32	>64	8		2	2		>32		>64	0.5		>32
449	Methicillin-resistant *Staphylococcus aureus*	0.5	32		16	>64	32	8	4	0.12	1	>64	>32	>64	8		2	2		>32		32	0.06		4
450	Methicillin-resistant *Staphylococcus aureus*	0.5	64		32	>64	>32	1	2	0.12	1	>64	>32	>64	8		2	2		>32		32	0.12		8
451	Methicillin-resistant *Staphylococcus aureus*	0.5	64		32	>64	>32	>16	1	0.12	1	>64	>32	>64	4		1	2		>32		>64	0.5		>32
452	Methicillin-resistant *Staphylococcus aureus*	0.5	64		32	>64	>32	>16	1	≤0.06	1	>64	>32	>64	8		2	2		>32		64	0.25		>32
453	Methicillin-resistant *Staphylococcus aureus*	0.5	>64		32	>64	>32	>16	1	0.12	1	>64	>32	>64	8		2	2		>32		32	0.25		>32
454	Methicillin-resistant *Staphylococcus aureus*	0.5	>64		>32	>64	>32	>16	1	≤0.06	1	>64	>32	>64	4		1	2		>32		32	0.12		>32
455	Methicillin-resistant *Staphylococcus aureus*	1	32		8	16	32	2	1	0.25	0.5	32	32	32	0.25		0.06	2		0.25		0.25	0.12		0.06
456	Methicillin-resistant *Staphylococcus aureus*	0.5	64		32	>64	>32	>16	1	≤0.06	1	>64	>32	>64	8		2	2		>32		64	0.12		>32
457	Methicillin-resistant *Staphylococcus aureus*	0.5	32		16	64	32	16	0.5	≤0.06	0.5	>64	>32	>64	>8		2	2		>32		0.25	0.12		0.06
458	Methicillin-resistant *Staphylococcus aureus*	0.5	64		32	>64	>32	>16	1	0.12	1	>64	>32	>64	8		2	2		>32		64	0.12		>32
459	Methicillin-resistant *Staphylococcus aureus*	0.5	>64		32	>64	>32	>16	2	≤0.06	1	>64	>32	>64	8		2	2		>32		64	0.12		>32
460	Methicillin-resistant *Staphylococcus aureus*	0.5	>64		4	8	>32	2	1	0.12	0.5	16	32	64	0.25		0.06	2		0.25		0.5	0.25		0.06
461	Methicillin-resistant *Staphylococcus aureus*	1	64		4	≤0.03	>32	2	1	0.12	0.5	32	16	64	0.25		0.25	2		0.25		0.5	0.25		0.25
462	Methicillin-resistant *Staphylococcus aureus*	0.25	>64		>32	>64	>32	>16	1	≤0.06	4	>64	>32	>64	8		2	1		>32		64	0.25		0.25
463	Methicillin-resistant *Staphylococcus aureus*	0.5	64		8	0.06	>32	1	1	0.06	0.25	16	16	32	0.25		0.06	2		0.25		0.5	0.25		0.12
464	Methicillin-resistant *Staphylococcus aureus*	0.5	64		32	>64	>32	>16	1	≤0.06	1	>64	>32	>64	>8		4	2		>32		>64	0.25		>32
465	Methicillin-resistant *Staphylococcus aureus*	0.5	64		4	≤0.03	32	1	0.5	≤0.06	0.25	16	16	64	0.25		0.06	4		0.25		0.5	0.25		0.12
466	Methicillin-resistant *Staphylococcus aureus*	0.5	64		4	0.25	>32	1	0.5	≤0.06	0.5	16	16	64	0.25		0.03	2		0.12		0.5	0.25		0.06
467	Methicillin-resistant *Staphylococcus aureus*	0.5	64		8	8	>32	2	0.5	0.5	0.25	>64	32	64	0.25		0.06	2		0.25		0.5	0.25		0.06
468	Methicillin-resistant *Staphylococcus aureus*	1	64		8	16	>32	2	1	0.12	0.5	>64	32	64	0.25		0.06	2		0.25		0.25	0.25		0.06
469	Methicillin-resistant *Staphylococcus aureus*	0.5	64		16	32	>32	4	1	≤0.06	0.5	>64	32	>64	0.25		0.06	2		0.25		0.25	0.25		0.12
470	Methicillin-resistant *Staphylococcus aureus*	0.5	64		8	32	>32	4	1	≤0.06	1	>64	>32	>64	0.25		0.06	2		0.25		0.25	0.25		0.06

Table s2: MIC Data for the Staphylococcus species tested

Isolate Number	*Staphylococcus* species	MIC (µg/ml) Values																							
			β Lactams					Carbapenem	Glycopeptides			Cephalosporins			Flouroquinolones			Oxazolidnone	Macrolides			Tetracyclines		Aminoglycoside	Antifolate
		XF-73	Penicillin	Oxacillin	Amoxicillin Clavulanate	Piperacillin/Tazobactam	Ampicillin	Meropenem	Vancomycin	Dalbavancin	Teicoplanin	Cefuroxime	Ceftriaxone	Ceftazidime	Levofloxacin	Ciprofloxacin	Moxifloxacin	Linezolid	Clindamycin	Clarithromycin	Erythromycin	Tetracycline	Tigecycline	Gentamicin	Trimethoprim/ Sulfmethoxazole
471	Methicillin-resistant *Staphylococcus aureus*	0.5	64		8	16	>32	2	1	0.12	0.5	64	>32	64	0.5		0.06	2		0.25		0.25	0.25		0.06
472	Methicillin-resistant *Staphylococcus aureus*	0.5	64		8	16	>32	4	1	0.12	0.5	32	32	64	0.25		0.06	2		0.25		0.25	0.25		0.06
473	Methicillin-resistant *Staphylococcus aureus*	0.25	64		8	16	>32	2	1	0.12	0.5	>64	32	64	0.25		0.06	2		0.25		0.25	0.25		0.06
474	Methicillin-resistant *Staphylococcus aureus*	0.5	>64		8	16	>32	2	1	0.12	0.5	64	32	64	0.25		0.06	2		0.25		0.5	0.25		0.12
475	Methicillin-resistant *Staphylococcus aureus*	0.5	64		8	16	>32	2	1	0.25	0.5	64	32	64	0.25		0.06	2		0.25		0.5	0.25		0.06
476	Methicillin-resistant *Staphylococcus aureus*	0.5	64		8	16	>32	2	1	0.25	1	32	32	64	0.25		0.06	2		0.25		0.25	0.25		0.06
477	Methicillin-resistant *Staphylococcus aureus*	0.25	64		8	16	>32	4	1	≤0.06	0.5	32	32	32	0.25		0.03	2		0.25		0.25	0.25		0.06
478	Methicillin-resistant *Staphylococcus aureus*	0.5	64		8	16	>32	2	1	0.12	1	64	32	64	0.25		0.06	2		0.25		0.5	0.25		0.12
479	Methicillin-resistant *Staphylococcus aureus*	0.5	16		16	16	16	4	1	≤0.06	0.25	32	32	>64	>8		8	2		>32		0.25	0.25		0.06
480	Methicillin-resistant *Staphylococcus aureus*	0.5	64		8	16	>32	1	1	≤0.06	0.5	32	32	64	0.5		0.06	2		0.25		0.25	0.25		0.06
481	Methicillin-resistant *Staphylococcus aureus*	0.5	64		8	32	>32	2	1	0.12	0.5	>64	>32	>64	0.25		0.06	2		0.25		0.5	0.25		0.12
482	Methicillin-resistant *Staphylococcus aureus*	1	>64		16	32	>32	4	2	0.12	0.5	>64	>32	>64	0.5		0.06	2		0.25		0.5	0.12		0.06
483	Methicillin-resistant *Staphylococcus aureus*	0.5	>64		8	16	>32	2	2	0.5	0.5	>64	>32	64	0.25		0.06	2		0.25		1	0.25		0.06
484	Methicillin-resistant *Staphylococcus aureus*	0.5	>64		8	8	>32	2	1	≤0.06	0.5	32	32	64	0.25		0.06	2		0.25		0.5	0.12		0.12
485	Methicillin-resistant *Staphylococcus aureus*	0.5	64		8	16	>32	2	1	≤0.06	0.5	>64	>32	64	0.25		0.06	2		0.25		0.25	0.12		0.12
486	Methicillin-resistant *Staphylococcus aureus*	0.25	64		8	16	>32	1	0.5	≤0.06	0.5	>64	>32	64	0.25		0.06	2		0.12		0.5	0.12		0.12
487	Methicillin-resistant *Staphylococcus aureus*	0.25	64		32	>64	>32	>16	1	0.12	0.5	>64	>32	>64	>8		4	1		>32		0.25	0.25		0.06
488	Methicillin-resistant *Staphylococcus aureus*	0.5	8		4	16	8	2	0.5	0.12	0.5	>64	>32	>64	0.12		0.03	1		>32		0.12	0.12		0.12
489	Methicillin-resistant *Staphylococcus aureus*	0.25	64		32	>64	>32	>16	1	0.25	0.5	>64	>32	>64	4		2	1		>32		64	0.5		0.06
490	Methicillin-resistant *Staphylococcus aureus*	0.5	64		>32	>64	32	>16	1	0.12	4	>64	>32	>64	>8		8	2		>32		>64	0.25		0.06
491	Methicillin-resistant *Staphylococcus aureus*	0.5	16		16	64	8	16	1	0.25	0.5	>64	>32	>64	>8		>8	1		>32		>64	1		0.06
492	Methicillin-resistant *Staphylococcus aureus*	0.5	64		>32	>64	32	>16	2	0.12	2	>64	>32	>64	>8		8	2		>32		>64	0.5		0.06
493	Methicillin-resistant *Staphylococcus aureus*	0.5	32		>32	>64	32	>16	1	0.12	1	>64	>32	>64	>8		>8	2		>32		2	1		0.06
494	Methicillin-resistant *Staphylococcus aureus*	2	32		>32	>64	32	>16	2	0.25	4	>64	>32	>64	>8		8	2		>32		0.25	0.25		0.06
495	Methicillin-resistant *Staphylococcus aureus*	1	32		>32	>64	32	>16	1	0.5	4	>64	>32	>64	>8		8	2		>32		>64	0.5		0.12
496	Methicillin-resistant *Staphylococcus aureus*	0.5	64		16	32	>32	4	1	0.12	1	>64	>32	32	0.25		0.03	2		>32		0.25	0.25		0.12
497	Methicillin-resistant *Staphylococcus aureus*	0.5	64		32	>64	32	>16	1	0.25	0.5	>64	>32	>64	>8		>8	1		>32		>64	2		0.06
498	Methicillin-resistant *Staphylococcus aureus*	0.25	64		32	>64	>32	>16	1	0.25	2	>64	>32	>64	>8		>8	2		>32		0.5	0.25		0.25
499	Methicillin-resistant *Staphylococcus aureus*	0.5	32		32	>64	32	>16	1	0.12	0.5	>64	>32	>64	>8		8	2		>32		1	0.12		0.06
500	Methicillin-resistant *Staphylococcus aureus*	0.5	64		32	>64	>32	>16	1	0.12	2	>64	>32	>64	>8		>8	2		>32		0.25	0.25		0.25
501	Methicillin-resistant *Staphylococcus aureus*	0.5	64		32	>64	>32	16	1	0.25	1	>64	>32	>64	>8		4	2		>32		64	0.12		0.12
502	Methicillin-resistant *Staphylococcus aureus*	0.5	64		16	64	>32	16	1	0.12	0.5	>64	>32	>64	>8		4	2		>32		0.12	0.12		0.12
503	Methicillin-resistant *Staphylococcus aureus*	0.5	32		32	64	32	>16	1	≤0.06	0.25	>64	>32	>64	>8		8	2		>32		0.12	0.12		0.06
504	Methicillin-resistant *Staphylococcus aureus*	1	32		32	>64	32	16	1	0.25	1	>64	>32	>64	>8		>8	2		>32		64	0.12		0.12
505	Methicillin-resistant *Staphylococcus aureus*	1	64		32	>64	>32	>16	4	0.5	1	>64	>32	>64	>8		>8	2		>32		8	0.12		0.12
506	Methicillin-resistant *Staphylococcus aureus*	2	64		32	>64	>32	>16	1	0.12	1	>64	>32	>64	8		2	2		>32		0.25	0.12		0.12
507	Methicillin-resistant *Staphylococcus aureus*	0.5	16		4	8	8	1	0.5	0.12	0.25	8	16	32	0.12		0.03	2		0.25		0.25	0.25		0.06
508	Methicillin-resistant *Staphylococcus aureus*	0.5	32		16	>64	16	2	0.5	0.12	0.25	>64	32	64	0.5		0.12	2		0.25		64	0.25		0.12
509	Methicillin-resistant *Staphylococcus aureus*	0.5	16		2	4	16	0.5	1	0.25	0.5	4	16	32	8		2	2		0.25		64	0.5		0.06
510	Methicillin-resistant *Staphylococcus aureus*	0.5	16		4	16	8	0.5	1	>0.5	0.5	32	>32	64	0.12		0.06	2		>32		0.25	0.12		0.06
511	Methicillin-resistant *Staphylococcus aureus*	0.5	32		8	16	16	1	0.5	0.06	0.5	8	16	32	0.25		0.03	2		>32		16	0.25		0.06
512	Methicillin-resistant *Staphylococcus aureus*	0.5	64		8	16	>32	2	1	0.5	0.5	>64	32	64	0.25		0.06	2		0.25		0.5	0.25		0.06
513	Methicillin-resistant *Staphylococcus aureus*	0.5	8		4	4	8	1	0.5	0.12	1	8	16	32	0.12		0.03	2		>32		0.5	0.25		0.06
514	Methicillin-resistant *Staphylococcus aureus*	0.5	32		4	8	16	1	0.5	0.12	0.5	8	32	64	0.25		0.015	2		>32		0.5	0.12		0.12
515	Methicillin-resistant *Staphylococcus aureus*	2	64		32	>64	>32	>16	1	0.06	1	>64	>32	>64	>8		4	2		>32		32	0.25		>32
516	Methicillin-resistant *Staphylococcus aureus*	1	0.25		2	0.25	1	2	0.5	≤0.06	0.5	4	16	16	0.25		0.03	2		>32		0.25	0.12		0.12
517	Methicillin-resistant *Staphylococcus aureus*	0.5	64		32	>64	>32	>16	1	0.5	1	>64	>32	>64	>8		4	1		>32		32	0.12		>32

Isolate Number	*Staphylococcus* species	MIC (µg/ml) Values	β Lactams					Carbapenem	Glycopeptides			Cephalosporins			Flouroquinolones			Oxazolidnone	Macrolides			Tetracyclines		Aminoglycoside	Antifolate
		XF-73	Penicillin	Oxacillin	Amoxicillin Clavulanate	Piperacillin/Tazobactam	Ampicillin	Meropenem	Vancomycin	Dalbavancin	Teicoplanin	Cefuroxime	Ceftriaxone	Ceftazidime	Levofloxacin	Ciprofloxacin	Moxifloxacin	Linezolid	Clindamycin	Clarithromycin	Erythromycin	Tetracycline	Tigecycline	Gentamicin	Trimethoprim/ Sulfmethoxazole
518	Methicillin-resistant *Staphylococcus aureus*	2	32		>32	>64	32	>16	2	0.5	4	>64	>32	>64	>8		>8	2		>32		4	2		0.06
519	Methicillin-resistant *Staphylococcus aureus*	0.5	32		32	>64	16	16	1	≤0.06	0.5	>64	>32	>64	>8		8	2		>32		64	1		0.25
520	Methicillin-resistant *Staphylococcus aureus*	0.5	32		32	>64	16	>16	1	0.12	1	>64	>32	>64	>8		8	2		>32		64	0.5		0.12
521	Methicillin-resistant *Staphylococcus aureus*	0.5	64		32	>64	32	4	0.5	≤0.06	0.5	>64	>32	>64	>8		>8	2		>32		64	0.25		0.12
522	Methicillin-resistant *Staphylococcus aureus*	0.5	32		16	>64	32	8	1	≤0.06	0.5	>64	>32	>64	0.25		0.06	2		>32		0.5	0.12		0.06
523	Methicillin-resistant *Staphylococcus aureus*	1	8		4	8	8	1	0.5	0.12	0.5	16	16	16	0.25		0.03	2		>32		0.12	0.12		0.06
524	Methicillin-resistant *Staphylococcus aureus*	0.25	32		8	64	16	2	1	0.25	0.5	>64	32	>64	0.25		0.06	2		>32		0.25	0.25		0.06
525	Methicillin-resistant *Staphylococcus aureus*	1	16		8	16	16	2	0.5	≤0.06	0.5	>64	32	64	0.25		0.06	2		>32		0.25	0.12		0.06
526	Methicillin-resistant *Staphylococcus aureus*	0.25	32		16	64	32	8	0.5	0.12	0.5	>64	>32	>64	0.25		0.06	2		0.25		0.25	0.25		0.06
527	Methicillin-resistant *Staphylococcus aureus*	0.5	16		32	>64	16	>16	1	0.12	2	>64	>32	>64	8		2	2		>32		64	0.5		0.06
528	Methicillin-resistant *Staphylococcus aureus*	0.5	64		>32	>64	32	>16	1	≤0.06	1	>64	>32	>64	>8		4	1		>32		0.12	0.06		0.12
529	Methicillin-resistant *Staphylococcus aureus*	0.5	64		>32	>64	32	>16	1	≤0.06	1	>64	>32	>64	>8		4	2		>32		0.12	0.12		0.12
530	Methicillin-resistant *Staphylococcus aureus*	0.5	64		>32	>64	32	>16	1	0.12	1	>64	>32	>64	>8		4	1		>32		0.12	0.06		0.12
531	Methicillin-resistant *Staphylococcus aureus*	0.5	32		32	>64	32	>16	1	≤0.06	1	>64	>32	>64	>8		>8	2		>32		>64	1		0.03
532	Methicillin-resistant *Staphylococcus aureus*	0.5	64		>32	>64	32	>16	1	≤0.06	1	>64	>32	>64	>8		4	1		>32		0.12	0.06		0.12
533	Methicillin-resistant *Staphylococcus aureus*	1	64		>32	>64	32	>16	1	≤0.06	1	>64	>32	>64	>8		4	1		>32		0.25	0.06		0.12
534	Methicillin-resistant *Staphylococcus aureus*	0.5	16		32	>64	16	>16	1	≤0.06	0.5	>64	>32	>64	>8		>8	1		>32		2	0.5		0.06
535	Methicillin-resistant *Staphylococcus aureus*	0.5	64		32	>64	>32	>16	2	≤0.06	2	>64	>32	>64	>8		4	2		>32		64	0.12		>32
536	Methicillin-resistant *Staphylococcus aureus*	0.5	32		32	>64	32	>16	1	≤0.06	1	>64	>32	>64	>8		4	1		>32		0.12	0.12		0.12
537	Methicillin-resistant *Staphylococcus aureus*	1	64		>32	>64	>32	>16	1	≤0.06	1	>64	>32	>64	>8		4	1		>32		0.12	0.06		0.12
538	Methicillin-resistant *Staphylococcus aureus*	0.5	64		32	>64	>32	>16	1	≤0.06	1	>64	>32	>64	>8		4	2		>32		>64	1		>32
539	Methicillin-resistant *Staphylococcus aureus*	2	64		32	>64	>32	>16	2	0.12	1	>64	>32	>64	8		2	1		>32		>64	1		32
540	Methicillin-resistant *Staphylococcus aureus*	0.5	64		32	>64	>32	>16	1	≤0.06	1	>64	>32	>64	8		2	1		>32		>64	1		32
541	Methicillin-resistant *Staphylococcus aureus*	0.5	64		32	>64	>32	16	2	≤0.06	1	>64	>32	>64	8		1	2		>32		>64	0.5		32
542	Methicillin-resistant *Staphylococcus aureus*	0.5	64		32	>64	>32	16	2	≤0.06	1	>64	>32	>64	8		2	1		>32		>64	0.5		32
543	Methicillin-resistant *Staphylococcus aureus*	1	>64		>32	>64	>32	>16	1	≤0.06	1	>64	>32	>64	>8		4	2		>32		>64	0.5		32
544	Methicillin-resistant *Staphylococcus aureus*	0.5	64		32	>64	>32	>16	1	≤0.06	1	>64	>32	>64	4		1	1		>32		>64	0.5		>32
545	Methicillin-resistant *Staphylococcus aureus*	1	64		32	>64	>32	16	2	0.12	1	>64	>32	>64	8		2	2		>32		>64	0.25		>32
546	Methicillin-resistant *Staphylococcus aureus*	0.5	64		32	>64	>32	>16	1	≤0.06	0.5	>64	>32	>64	4		1	1		>32		>64	2		32
547	Methicillin-resistant *Staphylococcus aureus*	0.5	64		32	>64	>32	16	1	≤0.06	0.5	>64	>32	>64	4		1	1		>32		>64	1		32
548	Methicillin-resistant *Staphylococcus aureus*	0.5	>64		32	>64	>32	>16	2	0.12	2	>64	>32	>64	>8		4	2		>32		64	0.5		0.5
549	Methicillin-resistant *Staphylococcus aureus*	0.5	64		4	4	>32	2	1	0.25	0.5	8	16	32	0.12		0.03	2		0.12		0.25	0.12		0.06
550	Methicillin-resistant *Staphylococcus aureus*	1	32		8	16	16	2	0.5	0.12	0.5	8	16	32	0.12		0.03	2		>32		0.25	0.25		0.06
551	Methicillin-resistant *Staphylococcus aureus*	0.5	64		4	8	>32	1	1	≤0.06	0.5	8	16	32	0.12		0.03	2		>32		0.25	0.12		0.06
552	Methicillin-resistant *Staphylococcus aureus*	1	32		8	32	32	4	1	≤0.06	0.5	64	16	64	0.12		0.03	2		0.12		0.12	0.12		0.12
553	Methicillin-resistant *Staphylococcus aureus*	0.5	32		8	8	>32	1	0.5	≤0.06	0.25	64	16	64	0.25		0.06	2		0.12		0.25	0.25		0.06
554	Methicillin-resistant *Staphylococcus aureus*	0.25	64		8	8	>32	1	1	≤0.06	1	64	16	64	0.25		0.03	2		0.12		0.25	0.25		0.06
555	Methicillin-resistant *Staphylococcus aureus*	0.25	64		4	8	32	1	0.5	≤0.06	0.5	32	16	32	0.25		0.03	2		0.12		0.25	0.12		0.06
556	Methicillin-resistant *Staphylococcus aureus*	0.5	>64		8	8	>32	1	1	0.12	0.5	32	16	64	0.25		0.06	2		>32		0.25	0.12		0.06
557	Methicillin-resistant *Staphylococcus aureus*	0.25	64		4	16	>32	2	0.5	0.12	0.5	8	16	32	0.25		0.03	2		0.12		0.25	0.25		0.06
558	Methicillin-resistant *Staphylococcus aureus*	0.25	64		8	16	>32	2	1	≤0.06	1	>64	>32	64	0.12		0.015	1		0.12		0.25	0.06		0.06
559	Methicillin-resistant *Staphylococcus aureus*	0.5	64		32	>64	>32	>16	1	≤0.06	0.5	>64	>32	>64	8		1	2		>32		0.25	0.12		0.25
560	Methicillin-resistant *Staphylococcus aureus*	0.5	32		4	16	32	0.5	0.5	0.12	0.25	16	32	64	2		0.5	2		0.25		0.12	0.12		0.06
561	Methicillin-resistant *Staphylococcus aureus*	0.5	64		16	32	>32	2	1	0.12	0.5	>64	32	32	0.06		0.015	2		>32		0.25	0.12		0.06
562	Methicillin-resistant *Staphylococcus aureus*	0.5	64		32	>64	>32	>16	1	≤0.06	0.5	>64	>32	>64	4		1	2		>32		0.25	0.12		0.12
563	Methicillin-resistant *Staphylococcus aureus*	0.5	>64		8	16	32	2	1	0.12	0.5	>64	>32	>64	0.12		0.015	2		0.25		0.25	0.12		0.06
564	Methicillin-resistant *Staphylococcus aureus*	0.5	16		0.5	1	8	0.12	0.5	0.12	0.5	2	2	16	0.25		0.03	2		0.12		0.12	1		0.06

		MIC (µg/ml) Values																							
			β Lactams					Carbapenem	Glycopeptides			Cephalosporins			Flouroquinolones			Oxazolidnone	Macrolides			Tetracyclines		Aminoglycoside	Antifolate
Isolate Number	***Staphylococcus* species**	XF-73	Penicillin	Oxacillin	Amoxicillin Clavulanate	Piperacillin/Tazobactam	Ampicillin	Meropenem	Vancomycin	Dalbavancin	Teicoplanin	Cefuroxime	Ceftriaxone	Ceftazidime	Levofloxacin	Ciprofloxacin	Moxifloxacin	Linezolid	Clindamycin	Clarithromycin	Erythromycin	Tetracycline	Tigecycline	Gentamicin	Trimethoprim/ Sulfmethoxazole
565	Methicillin-resistant *Staphylococcus aureus*	0.5		> 4					1	≤0.06	0.25					> 8		2	≤ 0.5		> 16		0.12	0.25	≤ 0.5
566	Methicillin-resistant *Staphylococcus aureus*	0.5		> 4					1	0.12	0.5					> 8		2	> 4		> 16		0.06	1	≤ 0.5
567	Methicillin-resistant *Staphylococcus aureus*	0.5		> 4					1	≤0.06	0.5					> 8		2	> 4		> 16		0.12	0.25	≤ 0.5
568	Methicillin-resistant *Staphylococcus aureus*	0.5		> 4					1	0.12	0.5					0.5		2	≤ 0.5		0.25		0.06	1	≤ 0.5
569	Methicillin-resistant *Staphylococcus aureus*	0.5		> 4					1	≤0.06	0.25					> 8		2	> 4		> 16		0.12	1	≤ 0.5
570	Methicillin-resistant *Staphylococcus aureus*	0.5		> 4					0.5	≤0.06	1					> 8		2	> 4		> 16		0.06	0.25	≤ 0.5
571	Methicillin-resistant *Staphylococcus aureus*	1		> 4					1	≤0.06	0.25					> 8		2	≤ 0.5		> 16		0.12	0.5	≤ 0.5
572	Methicillin-resistant *Staphylococcus aureus*	0.5		> 4					1	0.12	0.25					> 8		2	≤ 0.5		> 16		0.25	0.25	≤ 0.5
573	Methicillin-resistant *Staphylococcus aureus*	0.5		> 4					1	≤0.06	0.5					4		2	≤ 0.5		> 16		0.12	0.5	≤ 0.5
574	Methicillin-resistant *Staphylococcus aureus*	0.5		> 4					1	0.12	0.5					> 8		2	≤ 0.5		0.5		0.25	0.25	≤ 0.5
575	Methicillin-resistant *Staphylococcus aureus*	1		> 4					1	≤0.06	0.25					> 8		2	≤ 0.5		> 16		0.06	0.25	≤ 0.5
576	Methicillin-resistant *Staphylococcus aureus*	1		> 4					1	≤0.06	0.25					0.12		4	≤ 0.5		0.5		0.12	0.25	≤ 0.5
577	Methicillin-resistant *Staphylococcus aureus*	0.5		> 4					1	0.12	0.25					> 8		2	≤ 0.5		> 16		0.25	0.25	≤ 0.5
578	Methicillin-resistant *Staphylococcus aureus*	0.5		> 4					1	≤0.06	0.5					> 8		2	≤ 0.5		> 16		0.12	0.25	≤ 0.5
579	Methicillin-resistant *Staphylococcus aureus*	0.5		> 4					1	≤0.06	0.25					> 8		4	≤ 0.5		> 16		0.25	0.25	≤ 0.5
580	Methicillin-resistant *Staphylococcus aureus*	0.5		> 4					1	0.12	0.25					> 8		2	≤ 0.5		> 16		0.12	0.25	≤ 0.5
581	Methicillin-resistant *Staphylococcus aureus*	0.5		> 4					1	≤0.06	0.25					> 8		2	≤ 0.5		> 16		0.12	0.25	≤ 0.5
582	Methicillin-resistant *Staphylococcus aureus*	0.5		> 4					2	0.12	1					8		1	> 4		> 16		0.06	> 16	≤ 0.5
583	Methicillin-resistant *Staphylococcus aureus*	1		> 4					1	0.12	1					> 8		4	≤ 0.5		0.5		0.25	0.5	≤ 0.5
584	Methicillin-resistant *Staphylococcus aureus*	0.5		> 4					1	≤0.06	1					> 8		4	> 4		> 16		0.25	> 16	≤ 0.5
585	Methicillin-resistant *Staphylococcus aureus*	0.5		> 4					1	0.25	4					> 8		2	> 4		> 16		0.25	> 16	> 4
586	Methicillin-resistant *Staphylococcus aureus*	0.5		> 4					1	0.12	1					> 8		2	≤ 0.5		0.5		0.25	0.5	≤ 0.5
587	Methicillin-resistant *Staphylococcus aureus*	0.5		> 4					0.5	≤0.06	0.25					> 8		4	≤ 0.5		0.5		0.12	0.5	≤ 0.5
588	Methicillin-resistant *Staphylococcus aureus*	0.5		> 4					1	0.12	0.5					> 8		2	≤ 0.5		0.5		0.12	0.5	≤ 0.5
589	Methicillin-resistant *Staphylococcus aureus*	0.5		> 4					1	0.12	1					> 8		2	≤ 0.5		> 16		0.12	1	≤ 0.5
590	Methicillin-resistant *Staphylococcus aureus*	0.5		> 4					1	0.12	0.5					> 8		2	≤ 0.5		> 16		0.12	0.25	≤ 0.5
591	Methicillin-resistant *Staphylococcus aureus*	0.5		> 4					1	0.12	0.5					2		2	≤ 0.5		> 16		0.06	0.5	≤ 0.5
592	Methicillin-resistant *Staphylococcus aureus*	0.5		> 4					1	0.12	1					> 8		4	≤ 0.5		1		0.25	0.5	≤ 0.5
593	Methicillin-resistant *Staphylococcus aureus*	0.5		> 4					1	≤0.06	0.5					> 8		4	> 4		> 16		0.12	> 16	≤ 0.5
594	Methicillin-resistant *Staphylococcus aureus*	0.5		> 4					1	≤0.06	0.25					> 8		4	> 4		> 16		0.12	0.5	≤ 0.5
595	Methicillin-resistant *Staphylococcus aureus*	0.5		> 4					1	0.12	0.5					> 8		4	> 4		> 16		0.25	1	≤ 0.5
596	Methicillin-resistant *Staphylococcus aureus*	0.5		> 4					1	≤0.06	1					> 8		4	≤ 0.5		1		0.5	0.25	≤ 0.5
597	Methicillin-resistant *Staphylococcus aureus*	0.5		> 4					1	0.25	0.5					> 8		4	≤ 0.5		> 16		0.25	0.5	≤ 0.5
598	Methicillin-resistant *Staphylococcus aureus*	0.5		> 4					1	≤0.06	0.25					> 8		2	> 4		> 16		0.12	0.5	≤ 0.5
599	Methicillin-resistant *Staphylococcus aureus*	0.5		> 4					1	≤0.06	0.5					> 8		2	≤ 0.5		> 16		0.12	0.5	≤ 0.5
600	Methicillin-resistant *Staphylococcus aureus*	0.5		> 4					1	≤0.06	0.25					0.5		4	≤ 0.5		0.5		0.12	0.5	2
601	Methicillin-resistant *Staphylococcus aureus*	0.5		> 4					1	≤0.06	0.5					> 8		2	> 4		> 16		0.12	0.5	≤ 0.5
602	Methicillin-resistant *Staphylococcus aureus*	0.25		> 4					1	≤0.06	0.25					> 8		2	≤ 0.5		0.5		0.12	0.5	1
603	Methicillin-resistant *Staphylococcus aureus*	0.5		> 4					2	≤0.06	2					> 8		2	> 4		> 16		0.12	> 16	≤ 0.5
604	Methicillin-resistant *Staphylococcus aureus*	0.5		> 4					1	0.12	1					> 8		2	> 4		> 16		0.25	> 16	> 4
605	Methicillin-resistant *Staphylococcus aureus*	0.5		> 4					1	≤0.06	0.5					> 8		2	≤ 0.5		> 16		0.12	0.5	≤ 0.5
606	Methicillin-resistant *Staphylococcus aureus*	0.5		> 4					1	0.12	1					> 8		4	> 4		> 16		0.25	> 16	≤ 0.5
607	Methicillin-resistant *Staphylococcus aureus*	0.5		> 4					1	≤0.06	0.5					> 8		2	≤ 0.5		> 16		0.12	0.5	≤ 0.5
608	Methicillin-resistant *Staphylococcus aureus*	0.5		> 4					1	≤0.06	1					> 8		4	≤ 0.5		> 16		0.25	1	≤ 0.5
609	Methicillin-resistant *Staphylococcus aureus*	0.5		> 4					0.5	≤0.06	0.25					> 8		2	≤ 0.5		0.25		0.12	0.25	≤ 0.5
610	Methicillin-resistant *Staphylococcus aureus*	1		> 4					0.5	≤0.06	0.25					> 8		4	≤ 0.5		0.5		0.12	0.25	≤ 0.5
611	Methicillin-resistant *Staphylococcus aureus*	1		> 4					2	≤0.06	0.5					> 8		2	≤ 0.5		> 16		0.12	> 16	≤ 0.5

Isolate Number	*Staphylococcus* species	MIC (µg/ml) Values																							
			β Lactams					Carbapenem	Glycopeptides			Cephalosporins			Flouroquinolones			Oxazolidnone	Macrolides			Tetracyclines		Aminoglycoside	Antifolate
		XF-73	Penicillin	Oxacillin	Amoxicillin Clavulanate	Piperacillin/Tazobactam	Ampicillin	Meropenem	Vancomycin	Dalbavancin	Teicoplanin	Cefuroxime	Ceftriaxone	Ceftazidime	Levofloxacin	Ciprofloxacin	Moxifloxacin	Linezolid	Clindamycin	Clarithromycin	Erythromycin	Tetracycline	Tigecycline	Gentamicin	Trimethoprim/ Sulfmethoxazole
612	Methicillin-resistant *Staphylococcus aureus*	0.5		> 4					0.5	≤0.06	≤ 0.12					> 8		4	≤ 0.5		0.5		0.12	0.25	≤ 0.5
613	Methicillin-resistant *Staphylococcus aureus*	0.5		> 4					0.5	0.12	0.25					> 8		2	> 4		> 16		0.12	0.25	≤ 0.5
614	Methicillin-resistant *Staphylococcus aureus*	0.5		> 4					0.5	≤0.06	≤ 0.12					> 8		2	≤ 0.5		0.5		0.12	0.25	≤ 0.5
615	Methicillin-resistant *Staphylococcus aureus*	0.5		> 4					1	≤0.06	0.25					> 8		4	≤ 0.5		0.25		0.25	1	≤ 0.5
616	Methicillin-resistant *Staphylococcus aureus*	0.5		> 4					1	≤0.06	1					> 8		2	≤ 0.5		0.25		0.12	0.5	≤ 0.5
617	Methicillin-resistant *Staphylococcus aureus*	0.5		> 4					1	0.12	0.5					> 8		2	≤ 0.5		0.25		0.12	0.25	≤ 0.5
618	Methicillin-resistant *Staphylococcus aureus*	0.5		> 4					1	≤0.06	0.5					> 8		2	≤ 0.5		> 16		0.12	0.5	≤ 0.5
619	Methicillin-resistant *Staphylococcus aureus*	1		> 4					1	≤0.06	0.5					> 8		2	≤ 0.5		> 16		0.12	0.5	≤ 0.5
620	Methicillin-resistant *Staphylococcus aureus*	0.5		> 4					1	≤0.06	0.5					> 8		2	≤ 0.5		0.25		0.12	> 16	≤ 0.5
621	Methicillin-resistant *Staphylococcus aureus*	0.5		> 4					1	0.12	0.5					> 8		4	≤ 0.5		0.5		0.12	0.5	≤ 0.5
622	Methicillin-resistant *Staphylococcus aureus*	0.5		> 4					1	≤0.06	0.5					> 8		2	≤ 0.5		0.5		0.25	0.5	≤ 0.5
623	Methicillin-resistant *Staphylococcus aureus*	1		> 4					1	≤0.06	0.5					> 8		4	≤ 0.5		> 16		0.5	0.5	≤ 0.5
624	Methicillin-resistant *Staphylococcus aureus*	0.5		> 4					1	0.12	0.5					0.5		2	≤ 0.5		0.25		0.12	0.25	≤ 0.5
625	Methicillin-resistant *Staphylococcus aureus*	0.5		> 4					1	≤0.06	0.25					> 8		2	≤ 0.5		0.25		0.25	0.25	≤ 0.5
626	Methicillin-resistant *Staphylococcus aureus*	0.5		> 4					1	≤0.06	0.5					0.25		2	≤ 0.5		> 16		0.06	0.25	≤ 0.5
627	Methicillin-resistant *Staphylococcus aureus*	0.5		> 4					1	≤0.06	0.5					> 8		2	≤ 0.5		> 16		0.25	0.25	≤ 0.5
628	Methicillin-resistant *Staphylococcus aureus*	0.5		> 4					1	≤0.06	0.5					> 8		4	≤ 0.5		> 16		0.25	1	≤ 0.5
629	Methicillin-resistant *Staphylococcus aureus*	1		> 4					1	≤0.06	0.25					> 8		2	> 4		> 16		0.12	0.25	≤ 0.5
630	Methicillin-resistant *Staphylococcus aureus*	0.5		> 4					0.5	≤0.06	0.25					> 8		2	≤ 0.5		> 16		0.06	0.25	≤ 0.5
631	Methicillin-resistant *Staphylococcus aureus*	0.5		> 4					1	0.12	0.5					8		2	≤ 0.5		> 16		0.25	1	≤ 0.5
632	Methicillin-resistant *Staphylococcus aureus*	0.5		> 4					2	0.12	1					> 8		2	≤ 0.5		0.25		0.25	0.25	≤ 0.5
633	Methicillin-resistant *Staphylococcus aureus*	0.5		> 4					0.5	0.12	0.25					> 8		2	≤ 0.5		> 16		0.12	0.5	≤ 0.5
634	Methicillin-resistant *Staphylococcus aureus*	0.5		> 4					1	0.12	0.5					> 8		2	≤ 0.5		0.5		0.12	0.12	≤ 0.5
635	Methicillin-resistant *Staphylococcus aureus*	0.5		> 4					1	≤0.06	0.5					> 8		2	≤ 0.5		> 16		0.12	> 16	≤ 0.5
636	Methicillin-resistant *Staphylococcus aureus*	1		> 4					1	≤0.06	0.5					> 8		4	≤ 0.5		> 16		0.25	0.5	≤ 0.5
637	Methicillin-resistant *Staphylococcus aureus*	1		> 4					1	0.12	0.25					> 8		2	> 4		> 16		0.12	0.5	≤ 0.5
638	Methicillin-resistant *Staphylococcus aureus*	0.5		> 4					1	0.12	0.5					> 8		4	≤ 0.5		> 16		0.12	0.5	≤ 0.5
639	Methicillin-resistant *Staphylococcus aureus*	1		> 4					1	≤0.06	0.25					0.25		2	≤ 0.5		0.25		0.25	0.25	≤ 0.5
640	Methicillin-resistant *Staphylococcus aureus*	0.5		> 4					1	0.12	0.5					> 8		2	> 4		> 16		0.25	0.25	≤ 0.5
641	Methicillin-resistant *Staphylococcus aureus*	0.5		> 4					2	0.12	0.5					> 8		2	> 4		> 16		0.25	0.5	≤ 0.5
642	Methicillin-resistant *Staphylococcus aureus*	0.5		> 4					1	0.12	0.5					> 8		4	> 4		> 16		0.12	> 16	≤ 0.5
643	Methicillin-resistant *Staphylococcus aureus*	1		> 4					1	≤0.06	0.5					> 8		4	> 4		> 16		0.12	> 16	≤ 0.5
644	Methicillin-resistant *Staphylococcus aureus*	1		> 4					1	≤0.06	0.25					> 8		2	≤ 0.5		> 16		0.25	> 16	≤ 0.5
645	Methicillin-resistant *Staphylococcus aureus*	0.5		> 4					1	0.25	0.5					8		2	≤ 0.5		> 16		0.12	0.25	≤ 0.5
646	Methicillin-resistant *Staphylococcus aureus*	0.5		> 4					0.5	0.12	0.25					0.25		2	≤ 0.5		0.25		0.12	0.25	≤ 0.5
647	Methicillin-resistant *Staphylococcus aureus*	1		> 4					1	≤0.06	0.25					> 8		2	≤ 0.5		16		0.25	1	≤ 0.5
648	Methicillin-resistant *Staphylococcus aureus*	0.5		> 4					2	0.12	2					> 8		2	> 4		> 16		0.12	> 16	≤ 0.5
649	Methicillin-resistant *Staphylococcus aureus*	4		> 4					1	0.5	0.5					0.5		2	≤ 0.5		0.25		0.25	0.25	≤ 0.5
650	Methicillin-resistant *Staphylococcus aureus*	0.5		> 4					1	0.12	0.25					> 8		1	> 4		> 16		0.25	0.25	≤ 0.5
651	Methicillin-resistant *Staphylococcus aureus*	0.5		> 4					2	0.12	2					> 8		2	≤ 0.5		0.5		0.12	0.5	≤ 0.5
652	Methicillin-resistant *Staphylococcus aureus*	0.5		> 4					1	0.12	0.25					> 8		2	≤ 0.5		> 16		0.12	0.5	≤ 0.5
653	Methicillin-resistant *Staphylococcus aureus*	0.5		> 4					1	≤0.06	0.5					2		2	≤ 0.5		> 16		0.12	0.5	≤ 0.5
654	Methicillin-resistant *Staphylococcus aureus*	0.5		> 4					1	≤0.06	1					> 8		2	≤ 0.5		> 16		0.12	1	≤ 0.5
655	Methicillin-resistant *Staphylococcus aureus*	0.5		> 4					1	≤0.06	0.25					> 8		2	≤ 0.5		0.25		0.25	0.5	≤ 0.5
656	Methicillin-resistant *Staphylococcus aureus*	1		> 4					1	≤0.06	0.5					> 8		2	≤ 0.5		> 16		0.12	> 16	≤ 0.5
657	Methicillin-resistant *Staphylococcus aureus*	1		> 4					1	≤0.06	0.5					> 8		2	≤ 0.5		> 16		0.25	0.5	≤ 0.5
658	Methicillin-resistant *Staphylococcus aureus*	0.5		> 4					1	≤0.06	0.5					> 8		2	≤ 0.5		> 16		0.12	0.25	≤ 0.5

Table s2: MIC Data for the Staphylococcus species tested

Isolate Number	*Staphylococcus* species	MIC (µg/ml) Values	β Lactams					Carbapenem	Glycopeptides			Cephalosporins			Flouroquinolones			Oxazolidnone	Macrolides			Tetracyclines		Aminoglycoside	Antifolate
		XF-73	Penicillin	Oxacillin	Amoxicillin Clavulanate	Piperacillin/Tazobactam	Ampicillin	Meropenem	Vancomycin	Dalbavancin	Teicoplanin	Cefuroxime	Ceftriaxone	Ceftazidime	Levofloxacin	Ciprofloxacin	Moxifloxacin	Linezolid	Clindamycin	Clarithromycin	Erythromycin	Tetracycline	Tigecycline	Gentamicin	Trimethoprim/ Sulfmethoxazole
659	Methicillin-resistant *Staphylococcus aureus*	1		> 4					1	≤0.06	1					> 8		2	≤ 0.5		> 16		0.12	0.5	≤ 0.5
660	Methicillin-resistant *Staphylococcus aureus*	0.5		> 4					0.5	0.12	0.25					> 8		2	> 4		> 16		0.12	0.5	≤ 0.5
661	Methicillin-resistant *Staphylococcus aureus*	0.5		> 4					1	≤0.06	0.25					> 8		2	≤ 0.5		> 16		0.12	0.25	≤ 0.5
662	Methicillin-resistant *Staphylococcus aureus*	0.5		> 4					1	0.12	0.25					> 8		1	> 4		> 16		0.12	1	4
663	Methicillin-resistant *Staphylococcus aureus*	0.5		> 4					1	≤0.06	0.25					> 8		2	> 4		> 16		0.12	0.25	≤ 0.5
664	Methicillin-resistant *Staphylococcus aureus*	2		> 4					1	≤0.06	1					> 8		2	≤ 0.5		1		0.12	> 16	≤ 0.5
665	Methicillin-resistant *Staphylococcus aureus*	2		> 4					0.5	≤0.06	0.25					> 8		2	≤ 0.5		0.25		0.06	0.5	≤ 0.5
666	Methicillin-resistant *Staphylococcus aureus*	0.5		> 4					1	≤0.06	0.5					> 8		2	≤ 0.5		0.5		0.25	0.5	≤ 0.5
667	Methicillin-resistant *Staphylococcus aureus*	0.5		> 4					1	0.12	0.5					> 8		2	≤ 0.5		0.5		0.06	1	≤ 0.5
668	Methicillin-resistant *Staphylococcus aureus*	0.5		> 4					1	≤0.06	1					> 8		2	> 4		> 16		0.12	0.5	≤ 0.5
669	Methicillin-resistant *Staphylococcus aureus*	0.5		> 4					1	0.12	0.5					> 8		1	> 4		> 16		0.06	2	≤ 0.5
670	Methicillin-resistant *Staphylococcus aureus*	0.5		4					1	≤0.06	0.5					> 8		2	≤ 0.5		0.25		0.25	0.5	≤ 0.5
671	Methicillin-resistant *Staphylococcus aureus*	0.5		> 4					1	≤0.06	0.5					> 8		2	≤ 0.5		0.25		0.12	0.5	≤ 0.5
672	Methicillin-resistant *Staphylococcus aureus*	0.5		> 4					1	≤0.06	0.5					> 8		2	≤ 0.5		1		0.12	0.5	≤ 0.5
673	Methicillin-resistant *Staphylococcus aureus*	0.5		> 4					1	≤0.06	0.25					0.5		2	≤ 0.5		0.5		0.06	2	≤ 0.5
674	Methicillin-resistant *Staphylococcus aureus*	0.5		> 4					1	≤0.06	0.5					0.5		2	≤ 0.5		> 16		0.12	0.5	≤ 0.5
675	Methicillin-resistant *Staphylococcus aureus*	0.5		> 4					1	≤0.06	0.5					> 8		2	> 4		> 16		0.12	1	≤ 0.5
676	Methicillin-resistant *Staphylococcus aureus*	0.5		> 4					1	≤0.06	0.5					> 8		2	≤ 0.5		0.25		0.25	0.5	≤ 0.5
677	Methicillin-resistant *Staphylococcus aureus*	0.5		> 4					1	≤0.06	0.25					0.5		2	≤ 0.5		0.5		0.12	2	≤ 0.5
678	Methicillin-resistant *Staphylococcus aureus*	0.5		4					1	≤0.06	0.25					0.5		4	≤ 0.5		0.25		0.12	0.5	≤ 0.5
679	Methicillin-resistant *Staphylococcus aureus*	0.5		> 4					1	≤0.06	0.5					4		2	≤ 0.5		> 16		0.25	0.5	≤ 0.5
680	Methicillin-resistant *Staphylococcus aureus*	0.5		4					1	≤0.06	0.5					> 8		2	≤ 0.5		0.25		0.06	0.5	≤ 0.5
681	Methicillin-resistant *Staphylococcus aureus*	0.5		> 4					1	≤0.06	0.5					8		2	≤ 0.5		0.25		0.06	2	≤ 0.5
682	Methicillin-resistant *Staphylococcus aureus*	0.5		> 4					1	≤0.06	0.25					0.5		4	≤ 0.5		0.5		0.25	0.5	≤ 0.5
683	Methicillin-resistant *Staphylococcus aureus*	0.5		> 4					1	0.12	0.5					8		2	≤ 0.5		0.25		0.12	0.5	≤ 0.5
684	Methicillin-resistant *Staphylococcus aureus*	0.5		> 4					1	0.12	0.5					8		2	≤ 0.5		0.25		0.12	0.5	≤ 0.5
685	Methicillin-resistant *Staphylococcus aureus*	1		> 4					1	≤0.06	0.5					8		2	≤ 0.5		> 16		0.25	0.5	≤ 0.5
686	Methicillin-resistant *Staphylococcus aureus*	0.5		> 4					1	≤0.06	0.5					8		2	≤ 0.5		> 16		0.25	0.5	≤ 0.5
687	Methicillin-resistant *Staphylococcus aureus*	0.5		> 4					1	0.12	0.25					> 8		2	> 4		> 16		0.12	0.5	≤ 0.5
688	Methicillin-resistant *Staphylococcus aureus*	0.5		> 4					1	≤0.06	0.5					> 8		2	> 4		> 16		0.12	0.5	≤ 0.5
689	Methicillin-resistant *Staphylococcus aureus*	0.5		> 4					1	0.12	0.5					> 8		2	> 4		> 16		0.12	0.5	≤ 0.5
690	Methicillin-resistant *Staphylococcus aureus*	0.5		> 4					1	≤0.06	0.5					> 8		2	≤ 0.5		0.25		0.12	1	≤ 0.5
691	Methicillin-resistant *Staphylococcus aureus*	0.5		> 4					1	≤0.06	0.5					> 8		2	> 4		> 16		0.25	0.5	≤ 0.5
692	Methicillin-resistant *Staphylococcus aureus*	0.5		> 4					1	≤0.06	0.5					> 8		2	> 4		> 16		0.12	0.5	≤ 0.5
693	Methicillin-resistant *Staphylococcus aureus*	0.5		> 4					1	0.12	0.25					> 8		2	≤ 0.5		0.25		0.25	0.5	≤ 0.5
694	Methicillin-resistant *Staphylococcus aureus*	0.25		> 4					1	≤0.06	0.5					> 8		4	≤ 0.5		0.25		0.12	1	≤ 0.5
695	Methicillin-resistant *Staphylococcus aureus*	0.5		> 4					1	≤0.06	0.5					> 8		2	> 4		> 16		0.12	0.5	≤ 0.5
696	Methicillin-resistant *Staphylococcus aureus*	0.5		> 4					1	0.12	0.5					> 8		4	≤ 0.5		0.5		0.25	0.5	≤ 0.5
697	Methicillin-resistant *Staphylococcus aureus*	1		> 4					1	≤0.06	0.5					> 8		2	> 4		> 16		0.12	1	≤ 0.5
698	Methicillin-resistant *Staphylococcus aureus*	0.5		> 4					1	≤0.06	0.5					> 8		2	≤ 0.5		0.25		0.25	0.5	≤ 0.5
699	Methicillin-resistant *Staphylococcus aureus*	0.5		> 4					1	≤0.06	0.5					> 8		2	> 4		> 16		0.12	0.5	≤ 0.5
700	Methicillin-resistant *Staphylococcus aureus*	1		> 4					1	≤0.06	0.5					> 8		2	> 4		> 16		0.12	0.5	≤ 0.5
701	Methicillin-resistant *Staphylococcus aureus*	0.5		> 4					1	≤0.06	0.5					> 8		2	≤ 0.5		0.5		0.25	0.5	≤ 0.5
702	Methicillin-resistant *Staphylococcus aureus*	0.5		> 4					1	≤0.06	0.5					0.25		2	≤ 0.5		> 16		0.12	0.5	≤ 0.5
703	Methicillin-resistant *Staphylococcus aureus*	0.25		> 4					1	≤0.06	0.5					0.5		2	≤ 0.5		0.25		0.25	> 16	≤ 0.5
704	Methicillin-resistant *Staphylococcus aureus*	0.5		> 4					0.5	0.12	0.25					> 8		2	≤ 0.5		> 16		0.12	0.5	≤ 0.5
705	Methicillin-resistant *Staphylococcus aureus*	0.25		> 4					1	≤0.06	1					> 8		2	> 4		8		0.25	2	4

Table s2: MIC Data for the Staphylococcus species tested

Isolate Number	*Staphylococcus* species	MIC (µg/ml) Values	β Lactams					Carbapenem	Glycopeptides			Cephalosporins			Flouroquinolones			Oxazolidnone	Macrolides			Tetracyclines		Aminoglycoside	Antifolate
		XF-73	Penicillin	Oxacillin	Amoxicillin Clavulanate	Piperacillin/Tazobactam	Ampicillin	Meropenem	Vancomycin	Dalbavancin	Teicoplanin	Cefuroxime	Ceftriaxone	Ceftazidime	Levofloxacin	Ciprofloxacin	Moxifloxacin	Linezolid	Clindamycin	Clarithromycin	Erythromycin	Tetracycline	Tigecycline	Gentamicin	Trimethoprim/ Sulfmethoxazole
706	Methicillin-resistant *Staphylococcus aureus*	0.25		> 4					1	≤0.06	2					> 8		2	≤ 0.5		4		0.25	> 16	≤ 0.5
707	Methicillin-resistant *Staphylococcus aureus*	0.5		> 4					1	0.25	1					> 8		2	> 4		> 16		0.25	8	≤ 0.5
708	Methicillin-resistant *Staphylococcus aureus*	0.5		4					1	0.12	0.5					> 8		2	≤ 0.5		0.25		0.25	0.5	≤ 0.5
709	Methicillin-resistant *Staphylococcus aureus*	0.5		> 4					1	≤0.06	1					> 8		2	> 4		> 16		0.12	0.5	≤ 0.5
710	Methicillin-resistant *Staphylococcus aureus*	0.5		> 4					1	≤0.06	0.5					8		2	≤ 0.5		0.25		0.12	0.25	≤ 0.5
711	Methicillin-resistant *Staphylococcus aureus*	0.5		> 4					1	≤0.06	0.5					> 8		2	> 4		> 16		0.25	0.5	≤ 0.5
712	Methicillin-resistant *Staphylococcus aureus*	0.5		> 4					1	≤0.06	0.5					> 8		2	> 4		> 16		0.25	0.5	≤ 0.5
713	Methicillin-resistant *Staphylococcus aureus*	0.5		> 4					1	≤0.06	0.5					> 8		2	> 4		> 16		0.12	0.25	≤ 0.5
714	Methicillin-resistant *Staphylococcus aureus*	0.25		> 4					1	≤0.06	1					> 8		2	> 4		> 16		0.25	0.5	≤ 0.5
715	Methicillin-resistant *Staphylococcus aureus*	0.5		> 4					0.5	≤0.06	0.25					> 8		2	≤ 0.5		> 16		0.12	0.25	≤ 0.5
716	Methicillin-resistant *Staphylococcus aureus*	0.5		> 4					1	≤0.06	0.25					0.25		2	≤ 0.5		0.25		0.06	0.5	≤ 0.5
717	Methicillin-resistant *Staphylococcus aureus*	0.5		> 4					1	≤0.06	0.25					0.5		2	≤ 0.5		> 16		0.06	0.5	≤ 0.5
718	Methicillin-resistant *Staphylococcus aureus*	0.5		> 4					1	0.25	1					> 8		2	> 4		> 16		0.12	0.5	≤ 0.5
719	Methicillin-resistant *Staphylococcus aureus*	0.5		> 4					1	0.25	0.5					> 8		2	≤ 0.5		> 16		0.12	0.5	≤ 0.5
720	Methicillin-resistant *Staphylococcus aureus*	0.5		> 4					1	≤0.06	0.5					> 8		2	≤ 0.5		0.25		0.12	0.5	≤ 0.5
721	Methicillin-resistant *Staphylococcus aureus*	0.5		> 4					1	≤0.06	1					> 8		2	> 4		> 16		0.25	0.5	≤ 0.5
722	Methicillin-resistant *Staphylococcus aureus*	0.25		> 4					1	0.12	0.5					> 8		2	> 4		> 16		0.12	0.5	≤ 0.5
723	Methicillin-resistant *Staphylococcus aureus*	0.5		> 4					2	≤0.06	1					> 8		2	> 4		> 16		0.25	0.5	≤ 0.5
724	Methicillin-resistant *Staphylococcus aureus*	0.5		> 4					1	≤0.06	0.5					> 8		2	≤ 0.5		> 16		0.12	0.25	≤ 0.5
725	Methicillin-resistant *Staphylococcus aureus*	0.5		> 4					0.5	≤0.06	0.5					> 8		4	≤ 0.5		> 16		0.12	0.25	≤ 0.5
726	Methicillin-resistant *Staphylococcus aureus*	0.5		> 4					0.5	≤0.06	0.25					> 8		2	≤ 0.5		> 16		0.06	0.25	≤ 0.5
727	Methicillin-resistant *Staphylococcus aureus*	1		> 4					1	>0.5	0.25					> 8		2	≤ 0.5		> 16		0.06	0.5	≤ 0.5
728	Methicillin-resistant *Staphylococcus aureus*	0.5		> 4					1	0.12	0.25					> 8		2	> 4		> 16		0.12	1	≤ 0.5
729	Methicillin-resistant *Staphylococcus aureus*	0.5		4					1	0.12	0.5					1		2	> 4		> 16		0.12	1	≤ 0.5
730	Methicillin-resistant *Staphylococcus aureus*	0.5		> 4					1	≤0.06	0.5					> 8		2	> 4		> 16		0.12	0.5	≤ 0.5
731	Methicillin-resistant *Staphylococcus aureus*	0.5		> 4					1	≤0.06	0.5					> 8		2	> 4		> 16		0.12	0.5	≤ 0.5
732	Methicillin-resistant *Staphylococcus aureus*	0.5		> 4					0.5	≤0.06	0.25					> 8		2	≤ 0.5		> 16		0.12	0.5	≤ 0.5
733	Methicillin-resistant *Staphylococcus aureus*	0.5		> 4					1	≤0.06	0.5					> 8		2	≤ 0.5		> 16		0.06	0.5	≤ 0.5
734	Methicillin-resistant *Staphylococcus aureus*	0.5		> 4					1	0.12	0.5					> 8		2	≤ 0.5		> 16		0.12	> 16	≤ 0.5
735	Methicillin-resistant *Staphylococcus aureus*	0.5		> 4					1	0.12	0.5					0.5		2	≤ 0.5		0.25		0.06	> 16	≤ 0.5
736	Methicillin-resistant *Staphylococcus aureus*	0.5		> 4					0.5	≤0.06	0.25					> 8		2	≤ 0.5		0.25		0.06	0.5	≤ 0.5
737	Methicillin-resistant *Staphylococcus aureus*	0.5		> 4					1	≤0.06	0.5					8		2	≤ 0.5		0.25		0.12	0.5	≤ 0.5
738	Methicillin-resistant *Staphylococcus aureus*	0.5		> 4					1	≤0.06	0.5					> 8		2	> 4		> 16		0.25	0.25	≤ 0.5
739	Methicillin-resistant *Staphylococcus aureus*	0.5		> 4					1	0.12	0.25					> 8		2	> 4		> 16		0.25	0.5	≤ 0.5
740	Methicillin-resistant *Staphylococcus aureus*	0.5		> 4					1	≤0.06	0.5					0.12		2	≤ 0.5		0.25		0.25	0.5	≤ 0.5
741	Methicillin-resistant *Staphylococcus aureus*	0.5		> 4					1	≤0.06	1					> 8		2	> 4		> 16		0.12	0.5	≤ 0.5
742	Methicillin-resistant *Staphylococcus aureus*	0.5		> 4					1	≤0.06	0.5					> 8		2	> 4		> 16		0.12	0.5	≤ 0.5
743	Methicillin-resistant *Staphylococcus aureus*	0.5		> 4					1	≤0.06	0.5					> 8		2	> 4		> 16		0.06	1	≤ 0.5
744	Methicillin-resistant *Staphylococcus aureus*	0.5		> 4					1	0.12	0.5					> 8		2	> 4		> 16		0.12	1	≤ 0.5
745	Methicillin-resistant *Staphylococcus aureus*	0.5		> 4					1	≤0.06	0.5					> 8		2	> 4		> 16		0.12	0.5	≤ 0.5
746	Methicillin-resistant *Staphylococcus aureus*	0.5		> 4					1	≤0.06	0.25					0.12		2	≤ 0.5		0.25		0.06	0.5	≤ 0.5
747	Methicillin-resistant *Staphylococcus aureus*	0.5		> 4					1	≤0.06	0.5					> 8		2	> 4		> 16		0.12	0.5	≤ 0.5
748	Methicillin-resistant *Staphylococcus aureus*	0.5		> 4					1	0.25	1					> 8		2	> 4		> 16		0.12	0.5	≤ 0.5
749	Methicillin-resistant *Staphylococcus aureus*	0.5		> 4					1	≤0.06	1					> 8		2	≤ 0.5		> 16		0.12	0.5	≤ 0.5
750	Methicillin-resistant *Staphylococcus aureus*	0.5		4					1	≤0.06	0.5					0.5		2	> 4		> 16		0.12	0.5	≤ 0.5
751	Methicillin-resistant *Staphylococcus aureus*	1		> 4					1	0.12	0.5					> 8		2	≤ 0.5		> 16		0.12	0.5	≤ 0.5
752	Methicillin-resistant *Staphylococcus aureus*	1		> 4					1	0.12	0.5					> 8		2	≤ 0.5		> 16		0.12	0.5	≤ 0.5

Isolate Number	*Staphylococcus* species	MIC (µg/ml) Values																							
			β Lactams					Carbapenem	Glycopeptides			Cephalosporins			Flouroquinolones			Oxazolidnone	Macrolides			Tetracyclines		Aminoglycoside	Antifolate
		XF-73	Penicillin	Oxacillin	Amoxicillin Clavulanate	Piperacillin/Tazobactam	Ampicillin	Meropenem	Vancomycin	Dalbavancin	Teicoplanin	Cefuroxime	Ceftriaxone	Ceftazidime	Levofloxacin	Ciprofloxacin	Moxifloxacin	Linezolid	Clindamycin	Clarithromycin	Erythromycin	Tetracycline	Tigecycline	Gentamicin	Trimethoprim/ Sulfmethoxazole
753	Methicillin-resistant *Staphylococcus aureus*	1		> 4					1	0.12	0.5					> 8		1	> 4		> 16		0.06	0.5	≤ 0.5
754	Methicillin-resistant *Staphylococcus aureus*	1		> 4					1	≤0.06	1					> 8		2	> 4		> 16		0.25	0.5	≤ 0.5
755	Methicillin-resistant *Staphylococcus aureus*	1		> 4					1	≤0.06	0.25					> 8		2	> 4		> 16		0.12	0.5	≤ 0.5
756	Methicillin-resistant *Staphylococcus aureus*	1		> 4					1	≤0.06	0.5					> 8		2	> 4		> 16		0.12	0.5	≤ 0.5
757	Methicillin-resistant *Staphylococcus aureus*	1		> 4					1	0.12	0.5					8		1	≤ 0.5		> 16		0.12	> 16	> 4
758	Methicillin-resistant *Staphylococcus aureus*	1		> 4					1	≤0.06	1					> 8		2	> 4		> 16		0.12	> 16	> 4
759	Methicillin-resistant *Staphylococcus aureus*	1		4					1	0.12	0.5					0.25		2	> 4		> 16		0.12	1	≤ 0.5
760	Methicillin-resistant *Staphylococcus aureus*	1		> 4					0.5	0.12	1					> 8		2	> 4		> 16		0.25	0.25	≤ 0.5
761	Methicillin-resistant *Staphylococcus aureus*	1		> 4					1	0.12	0.25					> 8		2	> 4		> 16		0.12	0.5	≤ 0.5
762	Methicillin-resistant *Staphylococcus aureus*	1		> 4					1	≤0.06	1					> 8		2	> 4		> 16		0.12	0.5	≤ 0.5
763	Methicillin-resistant *Staphylococcus aureus*	1		> 4					1	0.12	0.5					> 8		2	> 4		> 16		0.12	0.25	≤ 0.5
764	Methicillin-resistant *Staphylococcus aureus*	1		> 4					1	0.12	0.5					> 8		2	> 4		> 16		0.25	> 16	1
765	Methicillin-resistant *Staphylococcus aureus*	1		> 4					1	0.12	0.5					8		2	≤ 0.5		0.25		0.5	0.5	≤ 0.5
766	Methicillin-resistant *Staphylococcus aureus*	1		> 4					1	0.12	0.5					8		2	≤ 0.5		0.25		0.12	0.5	≤ 0.5
767	Methicillin-resistant *Staphylococcus aureus*	1		> 4					1	0.12	0.5					> 8		2	> 4		> 16		0.12	0.5	≤ 0.5
768	Methicillin-resistant *Staphylococcus aureus*	1		> 4					1	0.12	0.25					> 8		2	≤ 0.5		0.25		0.06	0.5	≤ 0.5
769	Methicillin-resistant *Staphylococcus aureus*	1		> 4					1	0.12	0.25					8		2	≤ 0.5		0.25		0.12	0.5	≤ 0.5
770	Methicillin-resistant *Staphylococcus aureus*	0.5		> 4					1	≤0.06	0.5					> 8		1	≤ 0.5		> 16		0.12	> 16	> 4
771	Methicillin-resistant *Staphylococcus aureus*	0.5		> 4					1	0.12	0.25					> 8		2	≤ 0.5		> 16		0.25	1	≤ 0.5
772	Methicillin-resistant *Staphylococcus aureus*	0.5		> 4					1	0.12	0.5					0.5		4	≤ 0.5		> 16		0.12	0.5	≤ 0.5
773	Methicillin-resistant *Staphylococcus aureus*	0.5		> 4					1	0.12	0.5					8		2	≤ 0.5		> 16		0.06	0.5	≤ 0.5
774	Methicillin-resistant *Staphylococcus aureus*	0.5		> 4					2	≤0.06	2					4		≤ 0.5	≤ 0.5		≤ 0.12		0.06	0.25	≤ 0.5
775	Methicillin-resistant *Staphylococcus aureus*	0.25		> 4					1	0.12	2					> 8		2	> 4		> 16		0.12	> 16	≤ 0.5
776	Methicillin-resistant *Staphylococcus aureus*	0.25		> 4					1	0.12	1					0.5		4	≤ 0.5		> 16		0.12	1	≤ 0.5
777	Methicillin-resistant *Staphylococcus aureus*	0.5		> 4					1	≤0.06	0.5					0.5		2	≤ 0.5		> 16		0.12	0.5	≤ 0.5
778	Methicillin-resistant *Staphylococcus aureus*	0.25		> 4					1	0.12	1					> 8		2	> 4		> 16		0.12	> 16	≤ 0.5
779	Methicillin-resistant *Staphylococcus aureus*	0.25		> 4					1	0.12	1					> 8		2	> 4		> 16		0.12	> 16	≤ 0.5
780	Methicillin-resistant *Staphylococcus aureus*	0.25		> 4					1	0.12	0.5					> 8		2	> 4		> 16		0.12	> 16	≤ 0.5
781	Methicillin-resistant *Staphylococcus aureus*	0.5		> 4					1	0.12	1					> 8		2	> 4		> 16		0.5	> 16	≤ 0.5
782	Methicillin-resistant *Staphylococcus aureus*	0.5		> 4					1	0.12	2					> 8		2	> 4		> 16		0.5	> 16	≤ 0.5
783	Methicillin-resistant *Staphylococcus aureus*	0.25		> 4					1	0.12	2					> 8		2	> 4		> 16		0.12	> 16	≤ 0.5
784	Methicillin-resistant *Staphylococcus aureus*	0.25		> 4					1	0.12	2					> 8		2	> 4		> 16		0.12	> 16	≤ 0.5
785	Methicillin-resistant *Staphylococcus aureus*	0.25		> 4					1	0.12	1					> 8		2	> 4		> 16		0.25	> 16	≤ 0.5
786	Methicillin-resistant *Staphylococcus aureus*	0.25		> 4					1	≤0.06	1					> 8		2	≤ 0.5		0.5		0.25	> 16	≤ 0.5
787	Methicillin-resistant *Staphylococcus aureus*	0.5		> 4					1	0.12	0.5					> 8		2	≤ 0.5		> 16		0.12	0.5	≤ 0.5
788	Methicillin-resistant *Staphylococcus aureus*	0.25		> 4					1	0.12	1					> 8		2	> 4		> 16		0.12	> 16	≤ 0.5
789	Methicillin-resistant *Staphylococcus aureus*	0.5		> 4					2	0.12	2					> 8		2	> 4		> 16		0.12	> 16	≤ 0.5
790	Methicillin-resistant *Staphylococcus aureus*	0.5		> 4					1	0.12	2					> 8		2	> 4		> 16		0.25	> 16	≤ 0.5
791	Methicillin-resistant *Staphylococcus aureus*	0.5		> 4					1	0.12	1					> 8		2	> 4		> 16		0.12	> 16	≤ 0.5
792	Methicillin-resistant *Staphylococcus aureus*	0.5		> 4					1	≤0.06	0.5					> 8		4	≤ 0.5		> 16		0.12	0.5	≤ 0.5
793	Methicillin-resistant *Staphylococcus aureus*	1		> 4					2	0.12	2					> 8		4	≤ 0.5		> 16		1	0.25	≤ 0.5
794	Methicillin-resistant *Staphylococcus aureus*	1		> 4					2	≤0.06	2					> 8		2	> 4		> 16		0.5	> 16	≤ 0.5
795	Methicillin-resistant *Staphylococcus aureus*	0.5		> 4					1	0.25	2					> 8		2	> 4		> 16		0.12	> 16	≤ 0.5
796	Methicillin-resistant *Staphylococcus aureus*	1		> 4					1	0.12	0.5					> 8		4	≤ 0.5		> 16		0.25	0.5	≤ 0.5
797	Methicillin-resistant *Staphylococcus aureus*	1		> 4					1	≤0.06	0.5					> 8		2	≤ 0.5		0.25		0.12	0.25	≤ 0.5
798	Methicillin-resistant *Staphylococcus aureus*	1		> 4					1	0.12	0.5					> 8		1	≤ 0.5		0.25		0.12	0.5	≤ 0.5
799	Methicillin-resistant *Staphylococcus aureus*	0.5		> 4					0.5	≤0.06	0.25					> 8		2	> 4		> 16		0.06	> 16	≤ 0.5

Table s2: MIC Data for the Staphylococcus species tested

Isolate Number	*Staphylococcus* species	MIC (µg/ml) Values																							
			β Lactams					Carbapenem	Glycopeptides			Cephalosporins			Flouroquinolones			Oxazolidnone	Macrolides			Tetracyclines		Aminoglycoside	Antifolate
		XF-73	Penicillin	Oxacillin	Amoxicillin Clavulanate	Piperacillin/Tazobactam	Ampicillin	Meropenem	Vancomycin	Dalbavancin	Teicoplanin	Cefuroxime	Ceftriaxone	Ceftazidime	Levofloxacin	Ciprofloxacin	Moxifloxacin	Linezolid	Clindamycin	Clarithromycin	Erythromycin	Tetracycline	Tigecycline	Gentamicin	Trimethoprim/ Sulfmethoxazole
800	Methicillin-resistant *Staphylococcus aureus*	1		> 4					1	0.25	0.5					8		2	≤ 0.5		0.5		0.12	0.5	≤ 0.5
801	Methicillin-resistant *Staphylococcus aureus*	1		> 4					0.5	0.12	0.25					> 8		2	≤ 0.5		> 16		0.06	0.25	≤ 0.5
802	Methicillin-resistant *Staphylococcus aureus*	1		> 4					1	0.12	1					> 8		2	> 4		> 16		0.12	> 16	≤ 0.5
803	Methicillin-resistant *Staphylococcus aureus*	0.5		> 4					1	≤0.06	0.5					> 8		2	≤ 0.5		0.25		0.12	1	≤ 0.5
804	Methicillin-resistant *Staphylococcus aureus*	1		> 4					1	≤0.06	0.5					> 8		2	≤ 0.5		> 16		0.12	1	≤ 0.5
805	Methicillin-resistant *Staphylococcus aureus*	1		> 4					0.5	≤0.06	0.25					> 8		2	≤ 0.5		> 16		0.12	0.25	≤ 0.5
806	Methicillin-resistant *Staphylococcus aureus*	1		> 4					2	0.12	1					8		2	≤ 0.5		> 16		0.06	> 16	≤ 0.5
807	Methicillin-resistant *Staphylococcus aureus*	0.5		> 4					1	0.12	0.5					> 8		2	≤ 0.5		0.25		0.12	0.5	≤ 0.5
808	Methicillin-resistant *Staphylococcus aureus*	1		> 4					1	≤0.06	0.25					> 8		2	> 4		> 16		0.06	0.5	≤ 0.5
809	Methicillin-resistant *Staphylococcus aureus*	1		> 4					2	0.12	0.5					> 8		2	> 4		> 16		0.12	1	≤ 0.5
810	Methicillin-resistant *Staphylococcus aureus*	1		> 4					1	≤0.06	0.5					> 8		2	≤ 0.5		0.25		0.25	0.5	≤ 0.5
811	Methicillin-resistant *Staphylococcus aureus*	0.25		> 4					1	0.12	0.5					> 8		2	≤ 0.5		0.25		0.12	0.5	≤ 0.5
812	Methicillin-resistant *Staphylococcus aureus*	0.5		> 4					1	≤0.06	0.5					> 8		2	≤ 0.5		0.5		0.12	0.5	≤ 0.5
813	Methicillin-resistant *Staphylococcus aureus*	0.5		> 4					1	0.12	0.5					> 8		2	> 4		> 16		0.12	0.5	≤ 0.5
814	Methicillin-resistant *Staphylococcus aureus*	1		> 4					1	0.12	1					> 8		2	> 4		> 16		0.12	0.5	≤ 0.5
815	Methicillin-resistant *Staphylococcus aureus*	0.5		> 4					1	≤0.06	0.5					> 8		2	≤ 0.5		0.25		0.25	0.25	≤ 0.5
816	Methicillin-resistant *Staphylococcus aureus*	0.5		> 4					1	0.12	0.25					> 8		2	> 4		> 16		0.12	2	≤ 0.5
817	Methicillin-resistant *Staphylococcus aureus*	0.5		> 4					1	≤0.06	≤ 0.12					> 8		2	≤ 0.5		0.25		0.12	0.5	≤ 0.5
818	Methicillin-resistant *Staphylococcus aureus*	0.5		> 4					1	≤0.06	1					> 8		2	> 4		> 16		0.12	0.5	≤ 0.5
819	Methicillin-resistant *Staphylococcus aureus*	0.5		> 4					1	0.12	0.25					4		2	≤ 0.5		0.25		0.12	0.5	≤ 0.5
820	Methicillin-resistant *Staphylococcus aureus*	0.5		> 4					1	0.12	2					> 8		2	> 4		> 16		0.12	0.5	≤ 0.5
821	Methicillin-resistant *Staphylococcus aureus*	0.5		> 4					1	0.12	0.25					> 8		2	≤ 0.5		> 16		0.12	1	≤ 0.5
822	Methicillin-resistant *Staphylococcus aureus*	0.5		> 4					1	0.12	0.25					> 8		2	≤ 0.5		> 16		0.12	> 16	≤ 0.5
823	Methicillin-resistant *Staphylococcus aureus*	0.5		> 4					1	0.12	1					> 8		2	> 4		> 16		0.12	0.5	≤ 0.5
824	Methicillin-resistant *Staphylococcus aureus*	0.5		> 4					0.5	0.12	1					> 8		2	> 4		> 16		0.06	> 16	≤ 0.5
825	Methicillin-resistant *Staphylococcus aureus*	0.5		> 4					1	0.12	0.5					0.5		2	≤ 0.5		> 16		0.06	> 16	≤ 0.5
826	Methicillin-resistant *Staphylococcus aureus*	0.5		> 4					1	0.12	0.5					> 8		2	≤ 0.5		> 16		0.06	> 16	1
827	Methicillin-resistant *Staphylococcus aureus*	0.5		> 4					1	≤0.06	0.25					> 8		4	≤ 0.5		> 16		0.5	> 16	≤ 0.5
828	Methicillin-resistant *Staphylococcus aureus*	0.5		> 4					1	≤0.06	0.25					> 8		2	≤ 0.5		0.25		0.12	1	≤ 0.5
829	Methicillin-resistant *Staphylococcus aureus*	0.5		> 4					1	0.12	0.5					0.25		4	≤ 0.5		0.5		0.12	0.5	≤ 0.5
830	Methicillin-resistant *Staphylococcus aureus*	0.5		> 4					1	0.12	0.25					> 8		2	> 4		> 16		0.12	0.5	≤ 0.5
831	Methicillin-resistant *Staphylococcus aureus*	0.5		> 4					1	0.12	0.5					0.25		4	≤ 0.5		> 16		0.25	0.5	≤ 0.5
832	Methicillin-resistant *Staphylococcus aureus*	1		> 4					1	≤0.06	0.5					0.5		2	≤ 0.5		> 16		0.25	0.25	≤ 0.5
833	Methicillin-resistant *Staphylococcus aureus*	1		> 4					1	0.12	0.5					> 8		2	> 4		> 16		0.12	0.5	≤ 0.5
834	Methicillin-resistant *Staphylococcus aureus*	1		> 4					1	0.12	0.5					> 8		2	≤ 0.5		> 16		0.12	0.25	≤ 0.5
835	Methicillin-resistant *Staphylococcus aureus*	1		> 4					1	0.12	0.5					8		2	≤ 0.5		0.25		0.06	0.25	≤ 0.5
836	Methicillin-resistant *Staphylococcus aureus*	1		> 4					1	0.25	0.25					> 8		2	≤ 0.5		0.25		0.12	0.25	≤ 0.5
837	Methicillin-resistant *Staphylococcus aureus*	0.5		> 4					1	0.12	0.25					> 8		2	> 4		> 16		0.12	0.25	≤ 0.5
838	Methicillin-resistant *Staphylococcus aureus*	1		> 4					1	0.12	0.5					> 8		2	> 4		> 16		0.06	> 16	≤ 0.5
839	Methicillin-resistant *Staphylococcus aureus*	1		> 4					1	0.12	0.25					> 8		4	≤ 0.5		0.5		0.12	0.5	≤ 0.5
840	Methicillin-resistant *Staphylococcus aureus*	0.5		> 4					1	0.12	0.5					> 8		2	≤ 0.5		0.25		0.12	0.25	≤ 0.5
841	*Staphylococcus aureus*	0.5	≤0.03		0.12	0.5	0.06	0.06	1	0.12	0.5	1	2	8	>8		4	1		0.06		0.25	0.12		0.06
842	*Staphylococcus aureus*	0.5	16		1	1	8	0.25	1	0.12	0.25	1	2	8	>8		4	2		32		0.25	0.12		0.03
843	*Staphylococcus aureus*	1	≤0.03		0.12	1	0.12	0.12	0.5	0.12	0.25	1	4	8	8		2	2		0.25		0.25	0.12		0.06
844	*Staphylococcus aureus*	0.5	≤0.03		0.12	1	0.12	0.06	1	0.12	1	2	4	8	0.25		0.06	4		8		0.25	0.12		0.06
845	*Staphylococcus aureus*	0.5	≤0.03		0.25	0.5	0.06	0.12	0.5	0.12	0.5	1	4	8	0.25		0.03	2		>32		0.25	0.12		0.5
846	*Staphylococcus aureus*	0.5	8		1	1	4	0.12	0.5	0.12	0.25	1	4	16	0.25		0.03	2		>32		0.25	0.12		0.06

		MIC (µg/ml) Values																							
			β Lactams					Carbapenem	Glycopeptides			Cephalosporins			Flouroquinolones			Oxazolidnone	Macrolides			Tetracyclines		Aminoglycoside	Antifolate
**Isolate Number**	***Staphylococcus* species**	**XF-73**	**Penicillin**	**Oxacillin**	**Amoxicillin Clavulanate**	**Piperacillin/Tazobactam**	**Ampicillin**	**Meropenem**	**Vancomycin**	**Dalbavancin**	**Teicoplanin**	**Cefuroxime**	**Ceftriaxone**	**Ceftazidime**	**Levofloxacin**	**Ciprofloxacin**	**Moxifloxacin**	**Linezolid**	**Clindamycin**	**Clarithromycin**	**Erythromycin**	**Tetracycline**	**Tigecycline**	**Gentamicin**	**Trimethoprim/ Sulfmethoxazole**
847	*Staphylococcus aureus*	0.5	0.12		1	8	0.25	0.12	1	0.12	0.5	4	16	16	0.12		0.03	2		4		8	0.12		0.25
848	*Staphylococcus aureus*	0.5	≤0.03		0.25	1	0.12	0.06	1	0.06	0.5	1	4	8	0.25		0.03	2		0.25		0.25	0.12		0.06
849	*Staphylococcus aureus*	0.5	8		0.5	1	4	0.5	0.5	0.12	0.5	1	4	32	0.25		0.06	1		0.12		0.12	0.06		0.12
850	*Staphylococcus aureus*	0.5	0.12		0.5	1	0.12	0.12	1	0.12	0.5	1	4	8	8		2	2		0.25		0.25	0.12		0.06
851	*Staphylococcus aureus*	0.5	32		32	>64	32	>16	1	0.12	0.5	>64	>32	>64	>8		>8	2		>32		0.25	0.12		0.06
852	*Staphylococcus aureus*	0.5	0.25		2	16	2	0.5	1	0.12	0.5	8	32	64	>8		4	1		>32		0.25	0.12		32
853	*Staphylococcus aureus*	0.5	1		2	4	2	0.5	1	0.12	0.5	8	16	32	0.25		0.03	4		0.25		0.25	0.06		0.06
854	*Staphylococcus aureus*	1	64		16	64	>32	16	1	0.12	0.5	>64	>32	>64	8		2	2		>32		0.25	0.12		>32
855	*Staphylococcus aureus*	1	32		4	8	32	1	0.5	0.12	0.25	8	16	32	1		0.25	1		1		0.12	0.06		0.06
856	*Staphylococcus aureus*	0.5	64		8	16	>32	1	0.5	0.12	0.25	32	16	64	>8		4	2		32		0.12	0.06		0.06
857	*Staphylococcus aureus*	0.5	64		8	16	32	1	0.5	0.12	0.25	32	16	64	0.12		0.03	2		16		0.12	0.12		0.06
858	*Staphylococcus aureus*	0.25	8		2	4	4	0.5	0.5	0.12	0.25	4	8	32	0.25		0.06	2		>32		0.12	0.06		0.06
859	*Staphylococcus aureus*	0.5	64		8	32	32	2	1	0.12	0.5	64	16	64	8		2	2		32		0.25	0.12		0.12
860	*Staphylococcus aureus*	0.5	64		8	32	>32	2	1	0.12	0.5	>64	32	>64	>8		8	2		0.25		0.25	0.12		>32
861	*Staphylococcus aureus*	0.5	64		16	64	32	8	1	0.12	0.5	>64	>32	>64	>8		8	2		>32		1	0.25		0.06
862	*Staphylococcus aureus*	0.5	0.06		0.25	1	0.12	0.12	0.5	0.12	0.25	1	2	8	>8		4	2		>32		0.12	0.06		0.06
863	*Staphylococcus aureus*	0.5	1		0.5	1	1	0.12	1	0.12	1	2	4	8	0.12		0.03	2		0.25		0.25	0.12		0.12
864	*Staphylococcus aureus*	0.5	16		1	2	8	0.25	1	0.12	0.5	2	4	8	0.25		0.06	2		0.25		0.25	0.12		0.25
865	*Staphylococcus aureus*	0.5	8		0.5	0.5	8	0.12	1	0.12	0.25	1	2	8	4		2	2		0.5		0.25	0.12		0.06
866	*Staphylococcus aureus*	0.5	0.25		0.25	0.5	0.5	0.03	1	0.12	0.25	1	2	8	0.12		0.03	2		16		0.12	0.06		0.12
867	*Staphylococcus aureus*	0.25	0.06		0.12	1	0.12	0.12	1	0.12	0.5	1	2	8	0.25		0.06	2		0.25		0.25	0.25		0.12
868	*Staphylococcus aureus*	0.5	16		1	1	8	0.12	1	0.12	0.5	2	4	16	0.12		0.03	4		0.25		0.25	0.12		0.12
869	*Staphylococcus aureus*	0.5	8		0.5	1	4	0.12	1	0.12	0.5	1	4	8	0.25		0.03	2		0.25		0.25	0.12		0.12
870	*Staphylococcus aureus*	0.5	0.5		0.5	1	1	0.06	0.5	0.12	0.5	1	2	8	0.06		0.015	2		0.12		0.25	0.12		0.06
871	*Staphylococcus aureus*	0.5	1		0.5	1	1	0.12	1	0.12	0.25	1	4	8	0.25		0.03	2		0.25		0.25	0.12		0.12
872	*Staphylococcus aureus*	0.5	8		1	2	4	0.25	0.5	0.12	0.5	2	4	16	0.12		0.03	2		0.25		0.25	0.12		0.06
873	*Staphylococcus aureus*	1	0.5		0.12	0.25	0.25	0.06	1	0.12	0.25	0.5	2	8	0.12		0.03	2		0.25		0.25	0.12		0.06
874	*Staphylococcus aureus*	0.5	32		32	>64	32	16	1	0.12	0.25	>64	>32	>64	>8		4	4		>32		0.5	0.25		0.06
875	*Staphylococcus aureus*	1	32		16	64	32	8	0.5	0.12	0.25	>64	>32	>64	8		2	2		>32		0.25	0.12		0.06
876	*Staphylococcus aureus*	1	>64		16	32	>32	2	0.5	0.12	0.5	>64	>32	64	0.5		0.03	2		32		32	0.25		0.06
877	*Staphylococcus aureus*	0.5	32		16	64	32	16	1	0.12	0.25	>64	>32	>64	>8		>8	2		>32		0.25	0.25		0.06
878	*Staphylococcus aureus*	0.5	32		32	>64	32	16	1	0.12	0.5	>64	>32	>64	>8		8	2		>32		0.5	0.25		0.06
879	*Staphylococcus aureus*	0.25	64		>32	>64	>32	>16	1	0.12	0.5	>64	>32	>64	>8		>8	2		>32		0.5	0.25		0.12
880	*Staphylococcus aureus*	0.25	32		16	32	16	4	2	0.12	0.5	>64	>32	>64	>8		8	2		>32		0.25	0.12		0.06
881	*Staphylococcus aureus*	0.5	64		16	64	>32	4	1	0.12	0.5	>64	>32	>64	8		2	2		32		0.25	0.12		0.06
882	*Staphylococcus aureus*	0.5	32		16	64	32	16	1	0.12	1	>64	>32	>64	>8		4	2		>32		0.25	0.12		0.12
883	*Staphylococcus aureus*	0.5	32		32	>64	32	>16	1	0.12	0.5	>64	>32	>64	>8		>8	2		>32		0.25	0.12		0.06
884	*Staphylococcus aureus*	0.5	32		16	64	16	4	1	0.06	0.5	>64	>32	>64	>8		8	2		>32		0.25	0.25		0.06
885	*Staphylococcus aureus*	0.5	2		0.5	1	2	0.12	1	0.12	1	1	4	16	0.5		0.12	4		8		0.25	0.12		0.06
886	*Staphylococcus aureus*	0.5	16		1	2	8	0.12	1	0.12	0.5	2	4	16	0.25		0.06	2		0.12		0.25	0.5		0.06
887	*Staphylococcus aureus*	0.5	0.5		0.5	1	1	0.06	1	0.12	0.5	1	4	4	0.12		0.03	2		8		0.12	0.25		0.06
888	*Staphylococcus aureus*	0.5	≤0.03		0.12	0.5	0.03	0.06	1	0.12	0.5	1	2	16	0.25		0.03	1		0.25		0.12	0.25		0.06
889	*Staphylococcus aureus*	0.5	0.5		0.25	0.5	0.5	0.12	1	0.12	0.5	1	2	16	0.25		0.03	2		0.25		0.12	0.25		0.06
890	*Staphylococcus aureus*	0.5	32		0.25	0.5	2	0.06	1	0.12	1	1	2	8	0.12		0.03	1		0.12		0.12	0.25		0.06
891	*Staphylococcus aureus*	0.5	16		0.5	0.5	8	0.12	0.5	0.12	0.5	1	1	8	0.25		0.06	2		>32		0.06	0.12		0.06
892	*Staphylococcus aureus*	0.5	16		1	1	8	0.06	0.5	0.12	0.5	1	2	8	0.12		0.03	2		0.12		0.12	0.12		0.06
893	*Staphylococcus aureus*	0.5	1		0.5	1	1	0.06	1	0.12	0.5	1	4	16	0.25		0.03	2		>32		0.06	0.06		0.06

Isolate Number	*Staphylococcus* species	MIC (µg/ml) Values	β Lactams					Carbapenem	Glycopeptides			Cephalosporins			Flouroquinolones			Oxazolidnone	Macrolides			Tetracyclines		Aminoglycoside	Antifolate
		XF-73	Penicillin	Oxacillin	Amoxicillin Clavulanate	Piperacillin/Tazobactam	Ampicillin	Meropenem	Vancomycin	Dalbavancin	Teicoplanin	Cefuroxime	Ceftriaxone	Ceftazidime	Levofloxacin	Ciprofloxacin	Moxifloxacin	Linezolid	Clindamycin	Clarithromycin	Erythromycin	Tetracycline	Tigecycline	Gentamicin	Trimethoprim/ Sulfmethoxazole
894	*Staphylococcus aureus*	0.5	16		1	1	8	0.12	0.5	0.12	0.5	2	4	16	0.25		0.06	2		0.25		0.12	0.25		0.06
895	*Staphylococcus aureus*	0.5	≤0.03		0.12	0.5	0.06	0.06	0.5	0.12	0.25	1	2	16	0.25		0.12	1		0.12		0.12	0.25		0.06
896	*Staphylococcus aureus*	1	16		0.5	1	8	0.12	0.5	0.5	0.5	2	4	16	0.12		0.03	2		>32		0.25	0.12		0.06
897	*Staphylococcus aureus*	1	8		0.5	1	4	0.06	1	0.5	0.25	1	2	8	0.12		0.03	2		0.12		0.12	0.12		0.06
898	*Staphylococcus aureus*	0.5	64		8	32	>32	1	1	0.5	1	64	>32	64	0.25		0.06	2		0.12		0.12	0.25		0.12
899	*Staphylococcus aureus*	1	8		2	4	8	1	1	0.12	0.5	8	16	32	0.5		0.03	2		0.12		0.25	0.12		0.06
900	*Staphylococcus aureus*	1	16		16	64	16	>16	2	0.12	2	>64	>32	>64	>8		8	2		>32		0.25	0.25		0.06
901	*Staphylococcus aureus*	1	32		4	8	32	0.25	1	≤0.06	0.25	8	16	64	0.5		1	2		32		0.12	0.12		0.06
902	*Staphylococcus aureus*	0.5	32		4	16	32	1	0.5	0.25	0.5	8	16	64	0.25		0.03	2		32		0.12	0.12		0.06
903	*Staphylococcus aureus*	0.5	32		8	16	32	1	1	≤0.06	0.25	16	32	64	0.25		0.06	2		32		0.12	0.12		0.06
904	*Staphylococcus aureus*	1	16		4	16	16	2	1	0.5	0.25	16	32	64	>8		4	1		>32		0.12	0.12		0.06
905	*Staphylococcus aureus*	0.5	16		16	32	8	0.5	1	≤0.06	0.5	>64	>32	16	>8		8	2		>32		1	0.5		0.06
906	*Staphylococcus aureus*	0.5	32		32	64	32	0.5	1	0.5	0.5	>64	>32	16	>8		8	2		>32		0.12	0.12		0.03
907	*Staphylococcus aureus*	0.5	64		8	16	32	0.5	0.5	0.25	0.5	8	32	8	0.12		0.03	1		32		0.12	0.12		0.03
908	*Staphylococcus aureus*	1	64		8	32	>32	0.5	1	≤0.06	1	64	>32	8	0.25		0.03	2		32		0.12	0.12		0.06
909	*Staphylococcus aureus*	1	0.5		0.5	1	1	0.06	0.5	≤0.06	0.5	2	4	4	0.25		0.06	2		0.12		0.12	0.12		0.06
910	*Staphylococcus aureus*	1	0.25		0.25	1	0.5	0.12	0.5	≤0.06	0.25	1	4	16	0.25		0.06	2		0.25		0.06	0.12		0.06
911	*Staphylococcus aureus*	1	0.5		0.25	0.5	1	0.06	1	≤0.06	0.5	1	2	8	>8		8	1		1		0.12	0.12		0.06
912	*Staphylococcus aureus*	1	16		1	1	16	0.12	0.5	0.25	0.25	1	2	4	4		1	2		16		0.12	0.12		0.03
913	*Staphylococcus aureus*	0.5	≤0.03		0.12	0.5	0.06	0.06	1	≤0.06	0.5	1	2	8	1		0.25	2		0.25		0.12	0.25		0.06
914	*Staphylococcus aureus*	1	16		1	1	8	0.12	0.5	0.12	0.5	2	4	16	0.12		0.03	2		0.12		0.12	0.06		0.03
915	*Staphylococcus aureus*	1	8		1	1	8	0.12	0.5	≤0.06	0.25	4	4	32	0.12		0.015	2		32		0.12	0.25		0.03
916	*Staphylococcus aureus*	1	8		1	1	4	0.12	0.5	≤0.06	0.5	2	4	16	4		1	2		32		0.12	0.25		0.03
917	*Staphylococcus aureus*	1	≤0.03		0.12	0.5	0.12	0.06	1	≤0.06	0.5	1	4	16	0.25		0.06	2		0.12		0.25	0.12		0.06
918	*Staphylococcus aureus*	0.5	1		0.5	1	1	0.12	0.5	≤0.06	0.25	1	2	16	0.12		0.03	2		0.12		0.12	0.12		0.25
919	*Staphylococcus aureus*	1	0.25		0.25	0.5	0.25	0.25	1	≤0.06	0.5	1	4	4	0.25		0.03	2		>32		0.12	0.25		0.06
920	*Staphylococcus aureus*	0.5	4		0.5	1	2	0.12	0.5	0.25	0.5	1	2	8	0.25		0.06	2		0.12		0.12	0.12		0.06
921	*Staphylococcus aureus*	0.5	32		8	32	32	0.25	2	0.25	0.5	64	>32	4	4		1	2		>32		0.12	0.12		0.03
922	*Staphylococcus aureus*	1	2		0.5	2	2	0.25	1	0.25	0.5	4	4	8	>8		4	2		0.12		0.06	0.12		0.03
923	*Staphylococcus aureus*	0.5	64		8	32	>32	0.5	1	0.25	0.5	32	>32	32	>8		4	2		>32		0.12	0.25		32
924	*Staphylococcus aureus*	0.5	4		2	16	2	0.5	1	≤0.06	0.5	64	16	16	>8		4	2		>32		0.12	0.25		0.03
925	*Staphylococcus aureus*	0.5	8		2	8	2	0.5	1	≤0.06	0.5	16	32	16	>8		8	2		>32		0.12	0.12		0.03
926	*Staphylococcus aureus*	0.5	16		8	32	16	0.5	1	≤0.06	0.5	32	>32	8	>8		8	2		>32		0.12	0.25		4
927	*Staphylococcus aureus*	0.5	64		4	32	>32	1	1	0.25	0.5	64	32	64	4		1	2		0.12		0.12	0.12		0.06
928	*Staphylococcus aureus*	1	16		2	8	16	1	1	0.25	0.5	4	32	32	8		2	2		>32		0.12	0.12		0.06
929	*Staphylococcus aureus*	1	32		4	16	32	1	1	0.12	0.5	8	16	32	8		2	2		32		0.12	0.25		0.06
930	*Staphylococcus aureus*	0.5	64		8	32	>32	2	1	0.12	0.5	>64	>32	>64	8		2	2		32		0.12	0.12		0.06
931	*Staphylococcus aureus*	1	64		8	16	>32	1	0.5	≤0.06	0.5	64	>32	64	8		4	1		>32		0.12	0.12		>32
932	*Staphylococcus aureus*	1	8		1	2	8	0.12	1	≤0.06	0.25	2	4	8	0.06		0.015	1		0.12		0.12	0.06		0.06
933	*Staphylococcus aureus*	0.5	16		16	32	32	4	0.5	≤0.06	0.5	>64	>32	>64	>8		>8	2		>32		0.5	0.25		0.06
934	*Staphylococcus aureus*	0.5	16		1	1	16	0.06	1	≤0.06	0.25	1	4	8	0.12		0.03	2		32		0.12	0.12		0.06
935	*Staphylococcus aureus*	0.5	≤0.03		0.12	0.5	0.03	0.06	0.5	≤0.06	0.25	1	2	8	0.5		0.06	2		0.12		0.12	0.12		0.03
936	*Staphylococcus aureus*	0.5	8		0.5	1	8	0.12	0.5	≤0.06	0.25	1	8	16	0.25		0.06	2		0.12		0.12	0.12		0.03
937	*Staphylococcus aureus*	1	0.25		0.25	0.5	0.25	0.12	0.5	≤0.06	0.5	2	4	4	0.25		0.06	2		0.12		0.12	0.12		0.06
938	*Staphylococcus aureus*	0.5	2		0.5	1	2	0.12	0.5	≤0.06	0.25	2	4	4	0.25		0.06	2		>32		>64	0.25		0.06
939	*Staphylococcus aureus*	1	16		0.5	1	8	0.12	2	0.25	0.5	2	4	4	0.25		0.03	2		>32		0.12	0.12		0.06
940	*Staphylococcus aureus*	0.5	2		1	1	2	0.06	1	0.25	0.5	2	4	16	0.25		0.03	2		>32		0.12	0.12		0.06

Isolate Number	*Staphylococcus* species	MIC (µg/ml) Values																							
			β Lactams					Carbapenem	Glycopeptides			Cephalosporins			Flouroquinolones			Oxazolidnone	Macrolides			Tetracyclines		Aminoglycoside	Antifolate
		XF-73	Penicillin	Oxacillin	Amoxicillin Clavulanate	Piperacillin/Tazobactam	Ampicillin	Meropenem	Vancomycin	Dalbavancin	Teicoplanin	Cefuroxime	Ceftriaxone	Ceftazidime	Levofloxacin	Ciprofloxacin	Moxifloxacin	Linezolid	Clindamycin	Clarithromycin	Erythromycin	Tetracycline	Tigecycline	Gentamicin	Trimethoprim/ Sulfmethoxazole
941	*Staphylococcus aureus*	1	16		1	1	16	0.06	1	≤0.06	0.5	1	4	16	4		1	2		32		0.25	0.12		0.03
942	*Staphylococcus aureus*	0.5	0.06		0.12	≤0.03	≤0.015	0.12	0.5	≤0.06	0.5	1	2	16	0.12		0.015	2		0.25		0.12	0.12		0.06
943	*Staphylococcus aureus*	1	≤0.03		0.06	0.5	0.03	0.03	1	≤0.06	0.25	1	2	8	0.25		0.06	2		0.25		0.12	0.12		0.06
944	*Staphylococcus aureus*	1	0.06		0.12	0.12	0.12	0.06	0.5	≤0.06	0.25	0.25	2	4	0.12		0.03	1		0.06		0.12	0.06		0.03
945	*Staphylococcus aureus*	1	32		8	16	32	1	1	0.25	1	32	>32	64	4		2	2		32		0.25	0.12		0.06
946	*Staphylococcus aureus*	0.5	64		4	8	32	1	1	≤0.06	0.5	8	32	32	4		1	2		32		0.12	0.06		0.12
947	*Staphylococcus aureus*	0.5	16		16	64	16	8	1	≤0.06	0.5	>64	>32	>64	>8		4	2		>32		0.25	0.12		0.06
948	*Staphylococcus aureus*	1	4		8	32	4	8	1	≤0.06	1	>64	>32	>64	>8		8	2		>32		0.25	0.12		0.03
949	*Staphylococcus aureus*	0.5	32		16	16	32	2	1	≤0.06	0.5	>64	>32	>64	8		2	2		>32		0.25	0.12		0.06
950	*Staphylococcus aureus*	1	64		4	8	>32	1	1	≤0.06	0.5	16	32	64	0.25		0.06	4		16		0.25	0.25		0.06
951	*Staphylococcus aureus*	1	64		8	16	32	2	1	≤0.06	0.5	16	32	64	0.25		0.03	1		16		0.25	0.12		0.06
952	*Staphylococcus aureus*	2	32		4	8	32	0.5	1	≤0.06	0.5	8	16	64	0.25		0.12	2		16		0.25	0.12		0.06
953	*Staphylococcus aureus*	1	64		4	8	>32	2	0.5	0.25	0.5	16	16	64	0.25		0.06	2		16		0.25	0.25		0.12
954	*Staphylococcus aureus*	1	32		4	4	16	1	1	≤0.06	0.5	8	16	64	0.5		0.12	2		32		0.25	0.06		0.06
955	*Staphylococcus aureus*	1	8		4	16	16	4	1	≤0.06	0.5	>64	>32	64	>8		8	2		>32		0.5	0.12		0.06
956	*Staphylococcus aureus*	1	0.5		0.5	0.5	1	0.06	1	≤0.06	0.5	1	2	16	0.25		0.06	1		4		0.25	0.12		0.06
957	*Staphylococcus aureus*	0.25	0.12		0.25	0.5	0.25	0.06	1	≤0.06	0.5	1	2	8	0.25		0.06	1		0.12		0.12	0.12		0.06
958	*Staphylococcus aureus*	1	≤0.03		0.12	0.5	0.25	0.06	1	≤0.06	0.5	1	2	16	0.5		0.06	1		0.25		0.12	0.06		0.06
959	*Staphylococcus aureus*	0.5	8		0.5	1	16	0.06	1	≤0.06	0.5	1	2	2	0.25		0.06	1		0.12		0.25	0.12		0.06
960	*Staphylococcus aureus*	0.5	4		0.5	1	4	0.25	1	0.25	0.5	2	4	8	0.25		0.06	2		0.25		32	0.25		0.06
961	*Staphylococcus aureus*	0.5	16		0.25	0.25	4	0.06	1	≤0.06	0.5	0.5	2	4	0.25		0.03	1		0.12		0.12	0.12		0.06
962	*Staphylococcus aureus*	0.5	16		0.25	0.5	4	0.06	1	0.12	0.5	1	2	8	0.12		0.03	1		>32		0.12	0.06		0.06
963	*Staphylococcus aureus*	1	0.12		0.12	0.5	0.25	0.06	0.5	≤0.06	0.5	1	2	8	0.12		0.03	1		0.12		0.12	0.12		0.06
964	*Staphylococcus aureus*	0.5	4		0.5	0.5	2	0.12	0.5	≤0.06	0.25	1	2	8	0.06		0.015	1		0.12		0.12	0.12		0.03
965	*Staphylococcus aureus*	1	≤0.03		0.06	0.5	0.06	0.06	1	0.12	1	1	2	8	0.25		0.06	1		0.12		0.25	0.25		0.06
966	*Staphylococcus aureus*	1	8		1	1	4	0.06	0.5	≤0.06	0.25	1	2	8	0.12		0.03	1		0.12		0.12	0.25		0.06
967	*Staphylococcus aureus*	0.5	16		1	2	16	0.12	0.5	≤0.06	0.5	1	2	8	0.25		0.06	2		0.12		0.25	0.25		0.03
968	*Staphylococcus aureus*	1	32		16	64	16	4	1	≤0.06	0.5	>64	>32	>64	>8		>8	2		>32		0.25	0.5		0.06
969	*Staphylococcus aureus*	0.5	32		16	32	32	4	1	≤0.06	0.5	>64	>32	>64	>8		8	2		>32		0.25	0.25		0.06
970	*Staphylococcus aureus*	0.5	32		32	>64	32	>16	1	≤0.06	0.5	>64	>32	>64	>8		8	1		>32		0.12	0.25		0.12
971	*Staphylococcus aureus*	0.5	32		8	64	16	8	1	≤0.06	0.5	>64	>32	>64	0.25		0.06	1		16		0.06	0.25		0.06
972	*Staphylococcus aureus*	2	32		8	16	32	2	1	≤0.06	0.5	16	32	64	4		2	2		32		0.12	0.25		0.06
973	*Staphylococcus aureus*	1	64		8	32	>32	1	1	0.25	0.5	32	32	64	0.25		0.03	2		32		0.12	0.12		0.06
974	*Staphylococcus aureus*	1	64		8	32	32	1	1	0.25	1	16	32	64	0.25		0.03	2		0.25		0.12	0.12		0.06
975	*Staphylococcus aureus*	1	32		16	>64	32	16	1	≤0.06	0.5	>64	>32	>64	>8		>8	2		>32		0.25	0.12		0.06
976	*Staphylococcus aureus*	1	32		8	16	32	2	1	≤0.06	0.5	16	32	64	8		2	2		32		0.12	0.25		0.06
977	*Staphylococcus aureus*	0.5	16		8	32	16	8	1	0.12	0.5	>64	32	64	>8		8	2		>32		0.12	0.12		0.06
978	*Staphylococcus aureus*	1	64		8	32	>32	1	0.5	≤0.06	0.5	64	32	>64	4		2	1		32		0.12	0.12		0.06
979	*Staphylococcus aureus*	0.25	2		0.5	1	1	0.12	1	0.25	0.5	1	4	8	0.12		0.03	4		>32		0.5	0.12		0.12
980	*Staphylococcus aureus*	0.25	64		0.5	1	>32	0.12	1	0.25	0.5	1	2	8	0.12		0.03	2		0.25		0.5	0.12		0.06
981	*Staphylococcus aureus*	0.5	16		0.5	0.5	4	0.06	0.5	0.25	0.25	1	2	8	0.12		0.03	2		>32		0.25	0.03		0.12
982	*Staphylococcus aureus*	0.5	4		0.5	1	1	0.12	1	≤0.06	0.5	1	4	8	0.12		0.03	2		>32		0.5	0.06		0.06
983	*Staphylococcus aureus*	0.5	16		0.5	1	4	0.12	1	≤0.06	0.5	2	4	8	>8		8	2		>32		0.25	0.12		0.06
984	*Staphylococcus aureus*	0.25	32		1	1	32	0.12	0.5	0.25	0.5	2	4	8	0.12		0.03	2		0.12		0.25	0.03		0.06
985	*Staphylococcus aureus*	2	8		1	1	8	0.12	2	0.25	2	2	4	16	4		2	4		>32		1	0.12		2
986	*Staphylococcus aureus*	0.5	4		0.25	1	1	0.12	1	≤0.06	0.5	1	4	8	0.25		0.06	2		0.25		0.5	0.06		0.06
987	*Staphylococcus aureus*	0.5	64		0.5	1	32	0.25	1	≤0.06	0.5	2	4	8	0.25		0.03	4		>32		0.5	0.06		0.06

Table s2: MIC Data for the Staphylococcus species tested

Isolate Number	*Staphylococcus* species	MIC (µg/ml) Values	β Lactams					Carbapenem	Glycopeptides			Cephalosporins			Flouroquinolones			Oxazolidnone	Macrolides			Tetracyclines		Aminoglycoside	Antifolate
		XF-73	Penicillin	Oxacillin	Amoxicillin Clavulanate	Piperacillin/Tazobactam	Ampicillin	Meropenem	Vancomycin	Dalbavancin	Teicoplanin	Cefuroxime	Ceftriaxone	Ceftazidime	Levofloxacin	Ciprofloxacin	Moxifloxacin	Linezolid	Clindamycin	Clarithromycin	Erythromycin	Tetracycline	Tigecycline	Gentamicin	Trimethoprim/ Sulfmethoxazole
988	*Staphylococcus aureus*	0.5	0.12		0.25	0.5	0.12	0.06	1	≤0.06	0.5	1	2	8	0.12		0.06	2		0.5		0.5	0.06		0.03
989	*Staphylococcus aureus*	0.25	1		0.5	1	1	0.06	1	0.25	0.5	1	4	8	0.25		0.06	4		0.25		0.5	0.12		0.06
990	*Staphylococcus aureus*	1	16		0.5	1	8	0.12	1	0.12	1	2	4	8	0.25		0.06	2		0.25		0.25	0.03		0.06
991	*Staphylococcus aureus*	0.5	>64		16	64	>32	>16	1	0.12	0.5	>64	>32	>64	8		2	2		>32		0.5	0.06		0.06
992	*Staphylococcus aureus*	0.5	32		32	>64	32	>16	4	≤0.06	1	>64	>32	>64	>8		>8	2		>32		4	0.5		0.06
993	*Staphylococcus aureus*	0.5	0.06		0.25	1	0.12	0.12	1	0.12	0.5	2	4	32	>8		>8	4		>32		4	1		0.06
994	*Staphylococcus aureus*	0.5	64		16	>64	>32	>16	1	0.12	0.5	>64	>32	>64	>8		>8	4		>32		4	1		0.06
995	*Staphylococcus aureus*	1	4		0.5	1	2	0.12	1	≤0.06	0.5	2	4	16	0.25		0.06	4		>32		0.5	0.12		0.06
996	*Staphylococcus aureus*	0.5	8		0.25	0.5	4	0.06	1	0.25	1	1	2	8	0.12		0.03	2		0.12		0.12	0.25		0.06
997	*Staphylococcus aureus*	0.5	1		0.5	1	1	0.12	1	≤0.06	0.5	1	4	16	0.25		0.03	2		0.12		0.12	0.25		0.06
998	*Staphylococcus aureus*	0.5	0.25		0.25	0.5	0.5	0.06	1	≤0.06	0.5	1	2	8	0.12		0.03	1		>32		0.06	0.12		0.06
999	*Staphylococcus aureus*	0.5	8		0.5	0.5	2	0.12	0.5	≤0.06	0.5	1	2	16	0.25		0.06	2		0.12		0.12	0.12		0.06
1000	*Staphylococcus aureus*	0.5	8		0.25	0.5	4	0.12	0.5	≤0.06	0.5	1	2	8	0.25		0.06	2		0.12		0.12	0.12		0.06
1001	*Staphylococcus aureus*	0.5	8		0.25	0.5	4	0.12	1	0.25	0.5	1	2	8	0.25		0.06	2		0.12		0.12	0.12		0.12
1002	*Staphylococcus aureus*	0.25	16		0.5	0.5	8	0.12	1	0.25	2	1	4	16	0.12		0.03	1		>32		0.12	0.12		0.06
1003	*Staphylococcus aureus*	0.5	8		0.5	1	4	0.12	1	0.12	0.5	1	8	16	0.25		0.03	2		>32		64	0.12		0.06
1004	*Staphylococcus aureus*	0.5	8		0.5	0.5	4	0.12	1	≤0.06	0.5	1	2	16	0.12		0.03	1		>32		0.12	0.12		0.06
1005	*Staphylococcus aureus*	0.5	≤0.03		0.12	0.5	0.06	0.12	1	0.25	0.25	1	2	8	0.25		0.03	2		0.25		0.12	0.12		0.06
1006	*Staphylococcus aureus*	2	1		2	4	2	0.12	1	≤0.06	0.5	4	8	8	0.25		0.03	2		4		0.12	0.12		0.06
1007	*Staphylococcus aureus*	0.5	≤0.03		0.12	1	0.06	0.12	1	≤0.06	0.5	1	4	8	0.5		0.06	2		8		0.25	0.12		0.06
1008	*Staphylococcus aureus*	0.5	8		0.5	1	4	0.06	1	≤0.06	0.5	1	2	16	0.25		0.06	2		0.25		32	0.25		0.06
1009	*Staphylococcus aureus*	0.5	32		0.5	0.5	32	0.12	1	≤0.06	0.5	1	2	8	0.12		0.03	2		0.25		0.25	0.12		0.06
1010	*Staphylococcus aureus*	0.5	32		1	1	32	0.12	2	≤0.06	0.5	1	2	16	4		1	4		>32		32	1		0.06
1011	*Staphylococcus aureus*	0.5	16		0.5	0.5	8	0.06	1	≤0.06	0.5	1	2	8	0.12		0.03	2		0.25		64	0.12		0.06
1012	*Staphylococcus aureus*	0.5	0.5		0.25	0.5	1	0.06	1	≤0.06	0.5	1	2	8	0.25		0.03	2		>32		64	0.25		0.12
1013	*Staphylococcus aureus*	0.5	32		1	2	16	0.12	1	≤0.06	4	1	4	16	0.25		0.06	4		0.25		0.25	0.12		0.06
1014	*Staphylococcus aureus*	0.5	16		1	4	16	0.06	1	0.12	0.5	1	2	8	1		0.25	2		>32		0.25	0.12		2
1015	*Staphylococcus aureus*	0.5	32		1	2	32	0.06	1	≤0.06	0.5	1	2	16	0.25		0.06	2		0.25		0.25	0.12		0.12
1016	*Staphylococcus aureus*	0.5	2		0.5	1	2	0.06	1	0.12	0.5	1	2	8	0.25		0.03	2		0.25		0.12	0.12		0.06
1017	*Staphylococcus aureus*	0.5	8		1	1	8	0.12	2	0.25	0.5	1	4	16	0.25		0.06	2		>32		0.25	0.12		0.06
1018	*Staphylococcus aureus*	1	16		1	2	16	0.12	1	≤0.06	1	1	4	16	0.5		0.12	2		0.25		0.25	0.12		1
1019	*Staphylococcus aureus*	0.5	2		0.5	1	1	0.06	1	≤0.06	0.5	1	4	16	0.12		0.03	4		8		64	0.25		0.06
1020	*Staphylococcus aureus*	0.5	2		0.5	0.5	1	0.12	1	≤0.06	0.5	1	2	8	0.25		0.03	4		0.25		0.5	0.5		0.06
1021	*Staphylococcus aureus*	0.5	32		1	1	16	0.12	1	≤0.06	0.5	1	2	4	0.25		0.06	2		0.25		64	0.25		0.12
1022	*Staphylococcus aureus*	0.5	2		1	1	2	0.12	1	≤0.06	0.5	1	2	4	0.25		0.06	2		0.25		0.25	0.12		0.12
1023	*Staphylococcus aureus*	0.5	0.5		0.5	0.5	0.5	0.06	1	≤0.06	0.5	1	2	4	0.25		0.06	2		0.12		0.12	0.12		0.06
1024	*Staphylococcus aureus*	0.5	32		1	1	8	0.12	1	≤0.06	0.5	4	4	16	0.12		0.03	2		>32		0.25	0.12		0.06
1025	*Staphylococcus aureus*	0.5	0.03		0.12	0.5	0.06	0.25	1	≤0.06	0.5	1	2	8	0.25		0.06	2		0.25		0.25	0.12		0.06
1026	*Staphylococcus aureus*	0.5	2		0.5	1	2	0.06	1	≤0.06	0.5	1	2	8	0.25		0.03	2		0.25		0.25	0.12		0.12
1027	*Staphylococcus aureus*	0.5	16		0.5	1	16	0.12	1	≤0.06	0.25	1	2	8	0.25		0.06	2		0.25		0.25	0.25		0.06
1028	*Staphylococcus aureus*	0.5	16		0.5	0.5	8	0.12	0.5	≤0.06	1	1	2	4	0.12		0.06	2		0.25		0.25	0.12		0.12
1029	*Staphylococcus aureus*	0.5	≤0.03		0.12	0.5	0.03	0.12	0.5	≤0.06	0.5	1	2	4	0.12		0.03	2		0.12		0.25	0.12		0.06
1030	*Staphylococcus aureus*	0.5	0.5		0.25	0.5	0.5	0.06	1	≤0.06	0.5	1	2	4	0.12		0.03	2		0.25		0.12	0.06		0.06
1031	*Staphylococcus aureus*	2	64		1	1	16	0.12	1	≤0.06	0.5	2	4	4	0.5		0.06	2		0.25		64	0.25		0.06
1032	*Staphylococcus aureus*	0.5	4		0.5	1	2	0.12	1	≤0.06	0.5	1	2	2	0.25		0.06	2		0.25		0.25	0.12		0.06
1033	*Staphylococcus aureus*	0.5	16		0.5	1	8	0.12	1	≤0.06	0.5	1	2	4	0.12		0.06	4		0.12		0.25	0.12		0.06
1034	*Staphylococcus aureus*	1	16		0.5	1	4	0.12	0.5	≤0.06	0.5	2	4	4	0.25		0.06	2		0.25		0.25	0.12		0.06

Table s2: MIC Data for the Staphylococcus species tested

Isolate Number	*Staphylococcus* species	MIC (µg/ml) Values	β Lactams					Carbapenem	Glycopeptides			Cephalosporins			Flouroquinolones			Oxazolidnone	Macrolides			Tetracyclines		Aminoglycoside	Antifolate
		XF-73	Penicillin	Oxacillin	Amoxicillin Clavulanate	Piperacillin/Tazobactam	Ampicillin	Meropenem	Vancomycin	Dalbavancin	Teicoplanin	Cefuroxime	Ceftriaxone	Ceftazidime	Levofloxacin	Ciprofloxacin	Moxifloxacin	Linezolid	Clindamycin	Clarithromycin	Erythromycin	Tetracycline	Tigecycline	Gentamicin	Trimethoprim/ Sulfmethoxazole
1035	*Staphylococcus aureus*	0.5	64		1	1	32	0.12	1	≤0.06	0.5	2	2	4	0.25		0.06	2		0.25		0.5	0.12		0.06
1036	*Staphylococcus aureus*	0.5	32		2	2	32	0.25	1	≤0.06	0.5	2	4	4	0.25		0.06	1		0.12		0.06	0.06		0.06
1037	*Staphylococcus aureus*	1	2		0.5	1	2	0.12	2	≤0.06	0.5	1	2	4	0.12		0.03	2		0.25		0.25	0.12		0.03
1038	*Staphylococcus aureus*	0.5	0.06		0.25	0.5	0.12	0.06	1	≤0.06	1	2	4	8	0.25		0.06	2		0.25		0.25	0.12		0.06
1039	*Staphylococcus aureus*	0.5	2		0.5	1	2	0.12	1	≤0.06	0.5	1	2	4	0.12		0.03	2		0.12		0.25	0.25		0.12
1040	*Staphylococcus aureus*	0.5	32		0.5	1	32	0.06	0.5	≤0.06	0.5	1	2	4	0.25		0.06	2		0.25		64	0.25		0.12
1041	*Staphylococcus aureus*	0.5	2		1	1	2	0.12	1	≤0.06	1	2	4	4	0.25		0.03	2		0.25		0.25	0.12		0.06
1042	*Staphylococcus aureus*	1	2		1	1	2	0.06	1	≤0.06	0.5	2	4	4	0.25		0.06	2		>32		0.25	0.12		0.12
1043	*Staphylococcus aureus*	1	32		1	1	32	0.12	0.5	≤0.06	0.5	1	4	4	0.25		0.03	2		0.25		0.25	0.12		0.12
1044	*Staphylococcus aureus*	1	0.06		0.12	0.5	0.03	0.12	1	≤0.06	0.5	1	2	4	0.25		0.06	2		0.5		0.25	0.12		0.06
1045	*Staphylococcus aureus*	0.5	16		1	1	16	0.12	1	≤0.06	0.5	1	2	4	0.25		0.06	2		0.25		0.25	0.12		0.06
1046	*Staphylococcus aureus*	0.5	4		0.5	1	2	0.12	0.5	0.5	0.5	1	2	8	0.12		0.03	2		0.25		0.25	0.12		0.06
1047	*Staphylococcus aureus*	0.5	0.06		0.12	0.5	0.06	0.12	0.5	≤0.06	0.5	1	4	8	0.25		0.12	2		>32		0.25	0.25		2
1048	*Staphylococcus aureus*	1	4		0.5	1	2	0.06	0.5	≤0.06	0.25	2	4	8	0.12		0.03	4		0.25		0.25	0.12		0.25
1049	*Staphylococcus aureus*	0.5	32		0.5	1	32	0.12	1	≤0.06	0.5	2	4	8	0.25		0.03	2		0.12		0.12	0.06		0.25
1050	*Staphylococcus aureus*	0.5	0.06		0.25	1	0.12	0.12	0.5	0.12	0.25	2	4	8	0.25		0.03	2		0.25		0.25	0.12		0.06
1051	*Staphylococcus aureus*	0.5	0.06		0.12	1	0.06	0.12	0.5	≤0.06	0.25	2	4	8	0.25		0.03	2		0.25		0.25	0.12		0.06
1052	*Staphylococcus aureus*	0.5	0.06		0.25	1	0.12	0.12	2	≤0.06	0.25	1	4	16	0.25		0.06	4		0.25		0.5	0.25		0.06
1053	*Staphylococcus aureus*	0.5	1		0.5	1	2	0.12	0.5	≤0.06	0.5	2	4	8	0.12		0.03	2		0.25		0.25	0.25		0.06
1054	*Staphylococcus aureus*	0.5	4		0.5	1	2	0.06	1	≤0.06	1	1	2	8	0.12		0.03	2		0.25		64	0.25		0.06
1055	*Staphylococcus aureus*	0.5	0.06		0.12	0.5	0.12	0.12	1	≤0.06	0.25	0.5	2	8	0.12		0.03	2		0.25		0.25	0.12		0.06
1056	*Staphylococcus aureus*	1	0.06		0.12	0.5	0.12	0.12	1	≤0.06	0.25	0.5	2	4	0.25		0.03	2		0.12		0.25	0.12		0.06
1057	*Staphylococcus aureus*	0.5	0.03		0.12	1	0.06	0.12	1	≤0.06	0.5	1	4	16	0.25		0.06	2		0.25		0.25	0.12		0.06
1058	*Staphylococcus aureus*	1	64		2	2	32	0.25	1	≤0.06	0.5	2	8	16	0.5		0.06	2		0.25		0.12	0.12		0.06
1059	*Staphylococcus aureus*	0.5	16		1	2	16	0.12	0.5	≤0.06	0.5	1	4	16	0.06		0.015	2		0.25		0.25	0.12		0.06
1060	*Staphylococcus aureus*	0.5	4		1	1	4	0.25	1	0.12	1	2	4	16	0.25		0.06	2		0.25		0.5	0.5		0.06
1061	*Staphylococcus aureus*	1	1		1	2	2	0.12	0.5	≤0.06	0.25	1	4	16	8		1	1		>32		0.12	0.12		0.06
1062	*Staphylococcus aureus*	1	16		1	2	16	0.12	0.5	≤0.06	0.25	2	4	8	0.25		0.06	2		0.25		0.25	0.12		0.06
1063	*Staphylococcus aureus*	1	32		1	1	32	0.12	1	≤0.06	0.25	2	4	16	1		0.12	2		4		0.5	0.25		0.12
1064	*Staphylococcus aureus*	1	32		1	2	32	0.12	1	≤0.06	0.5	2	4	16	0.25		0.06	2		0.25		0.5	0.5		0.12
1065	*Staphylococcus aureus*	1	16		1	2	8	0.25	1	≤0.06	0.5	2	4	8	0.25		0.03	2		0.25		0.25	0.25		0.5
1066	*Staphylococcus aureus*	1	16		1	1	32	0.25	0.5	≤0.06	0.5	2	4	8	0.25		0.03	2		0.25		0.25	0.25		0.06
1067	*Staphylococcus aureus*	0.5	16		0.5	1	32	0.12	1	≤0.06	1	1	4	16	0.12		0.03	1		0.25		0.25	0.25		0.06
1068	*Staphylococcus aureus*	0.5	1		0.5	1	1	0.12	1	0.12	0.5	1	2	8	0.25		0.03	2		0.25		0.25	0.25		0.06
1069	*Staphylococcus aureus*	1	16		0.5	1	32	0.12	1	≤0.06	0.5	1	4	8	0.12		0.03	2		0.25		0.12	0.12		0.06
1070	*Staphylococcus aureus*	1	32		1	1	16	0.12	1	≤0.06	0.5	2	4	8	>8		4	2		>32		0.25	0.25		>32
1071	*Staphylococcus aureus*	1	16		0.5	1	4	0.12	0.5	≤0.06	0.5	1	4	8	0.5		0.03	1		0.12		0.25	0.12		0.12
1072	*Staphylococcus aureus*	0.5	0.06		0.25	1	0.12	0.12	0.5	0.12	1	1	4	8	0.12		0.03	2		0.25		0.25	0.12		0.06
1073	*Staphylococcus aureus*	0.5	16		0.5	1	16	0.12	1	≤0.06	0.5	1	2	8	0.25		0.03	2		0.12		0.25	0.12		0.12
1074	*Staphylococcus aureus*	1	16		0.5	1	16	0.06	1	≤0.06	0.5	1	4	16	0.5		0.06	2		0.12		0.25	0.12		0.06
1075	*Staphylococcus aureus*	1	0.5		0.25	0.5	0.5	0.12	1	≤0.06	0.5	1	2	8	0.25		0.06	2		0.25		0.12	0.12		0.06
1076	*Staphylococcus aureus*	1	16		0.5	0.5	16	0.06	1	≤0.06	1	1	2	8	0.12		0.03	2		0.12		0.25	0.12		0.06
1077	*Staphylococcus aureus*	0.5	0.06		0.25	0.5	0.06	0.12	1	≤0.06	0.5	2	4	16	0.25		0.06	2		0.25		0.25	0.12		0.06
1078	*Staphylococcus aureus*	0.5	8		0.5	0.5	4	0.12	1	≤0.06	0.5	1	2	16	8		2	2		0.12		0.25	0.12		>32
1079	*Staphylococcus aureus*	0.5	16		0.5	0.5	8	0.12	1	≤0.06	0.5	1	2	8	0.25		0.06	2		0.12		0.5	0.12		0.06
1080	*Staphylococcus aureus*	0.5	16		1	1	16	0.12	0.5	≤0.06	0.5	2	4	4	0.25		0.06	2		0.12		0.25	0.12		0.06
1081	*Staphylococcus aureus*	1	16		1	1	8	0.12	1	≤0.06	0.5	2	4	4	0.25		0.06	2		0.5		0.25	0.12		0.06

Isolate Number	*Staphylococcus* species	MIC (µg/ml) Values																							
			β Lactams					Carbapenem	Glycopeptides			Cephalosporins			Flouroquinolones			Oxazolidnone	Macrolides			Tetracyclines		Aminoglycoside	Antifolate
		XF-73	Penicillin	Oxacillin	Amoxicillin Clavulanate	Piperacillin/Tazobactam	Ampicillin	Meropenem	Vancomycin	Dalbavancin	Teicoplanin	Cefuroxime	Ceftriaxone	Ceftazidime	Levofloxacin	Ciprofloxacin	Moxifloxacin	Linezolid	Clindamycin	Clarithromycin	Erythromycin	Tetracycline	Tigecycline	Gentamicin	Trimethoprim/ Sulfmethoxazole
1082	*Staphylococcus aureus*	0.5	16		0.5	1	4	0.06	0.5	≤0.06	0.25	1	4	8	0.25		0.03	1		0.12		0.25	0.12		0.06
1083	*Staphylococcus aureus*	0.5	2		1	1	1	0.12	1	≤0.06	0.5	4	4	16	0.25		0.03	2		0.25		0.5	0.25		0.12
1084	*Staphylococcus aureus*	1	32		1	1	>32	0.12	1	0.12	0.5	2	2	8	0.12		0.03	0.25		0.25		16	0.25		0.12
1085	*Staphylococcus aureus*	0.5	64		0.5	1	32	0.12	0.5	0.12	0.5	1	2	8	0.12		0.03	2		0.12		64	0.12		0.5
1086	*Staphylococcus aureus*	0.5	4		1	1	4	0.06	0.5	0.12	0.5	1	2	8	0.12		0.03	8		0.25		0.25	0.12		0.12
1087	*Staphylococcus aureus*	0.5	≤0.03		0.12	1	0.06	0.06	0.5	0.12	1	1	2	1	0.25		0.06	2		0.25		0.25	0.12		0.06
1088	*Staphylococcus aureus*	1	4		0.5	1	4	0.06	1	0.12	0.5	1	2	1	0.25		0.03	2		0.25		0.25	0.25		0.12
1089	*Staphylococcus aureus*	1	16		0.5	1	8	0.12	1	≤0.06	1	1	2	1	0.12		0.03	2		0.25		1	0.12		0.06
1090	*Staphylococcus aureus*	1	16		1	1	8	0.06	1	0.12	0.5	1	2	2	0.25		0.06	0.5		0.25		0.25	0.12		0.06
1091	*Staphylococcus aureus*	0.5	2		1	1	2	0.12	1	≤0.06	0.5	2	2	1	0.25		0.03	4		0.25		32	0.5		4
1092	*Staphylococcus aureus*	0.5	32		0.5	1	16	0.12	1	≤0.06	0.5	1	1	0.5	0.12		0.015	1		0.12		0.25	0.12		0.06
1093	*Staphylococcus aureus*	0.5	32		1	1	32	0.12	1	0.12	0.5	4	2	1	0.25		0.03	2		0.25		0.25	0.12		0.06
1094	*Staphylococcus aureus*	0.5	1		0.5	0.5	0.5	0.06	1	≤0.06	0.5	1	2	8	0.06		0.015	1		0.12		0.12	0.06		0.06
1095	*Staphylococcus aureus*	0.5	64		1	1	>32	0.12	0.5	≤0.06	0.5	2	4	8	0.06		0.015	2		0.12		16	0.12		0.06
1096	*Staphylococcus aureus*	0.25	≤0.03		0.015	0.06	0.015	≤0.008	0.25	≤0.06	0.06	≤0.03	0.015	0.12	0.5		0.12	1		≤0.03		0.12	0.03		0.06
1097	*Staphylococcus aureus*	0.5	8		0.5	1	4	0.06	1	≤0.06	0.5	1	2	8	0.25		0.03	1		0.12		0.06	0.12		0.06
1098	*Staphylococcus aureus*	0.5	1		0.5	1	1	0.25	1	0.12	0.5	2	4	16	0.12		0.03	1		0.25		0.06	0.12		0.03
1099	*Staphylococcus aureus*	0.5	1		0.5	1	2	0.12	1	≤0.06	0.5	1	2	8	0.12		0.03	1		0.12		0.06	0.12		0.5
1100	*Staphylococcus aureus*	0.5	32		0.5	1	32	0.12	1	≤0.06	0.5	1	2	8	0.25		0.06	2		0.25		0.25	0.25		0.06
1101	*Staphylococcus aureus*	0.5	1		0.5	1	1	0.12	1	≤0.06	0.5	1	2	16	0.25		0.03	1		0.12		0.06	0.12		0.06
1102	*Staphylococcus aureus*	0.5	8		1	1	4	0.25	2	≤0.06	0.5	2	4	16	0.5		0.12	1		0.12		0.12	0.12		0.06
1103	*Staphylococcus aureus*	0.5	8		0.5	1	4	0.06	1	≤0.06	0.25	1	2	8	0.06		0.015	1		0.12		0.12	0.12		0.03
1104	*Staphylococcus aureus*	0.5	8		0.5	1	8	0.12	0.5	0.12	0.5	1	2	8	0.12		0.03	1		0.12		0.12	0.12		0.06
1105	*Staphylococcus aureus*	0.5	1		0.5	1	2	0.12	1	0.12	0.5	1	2	32	>8		4	1		0.12		0.06	0.06		0.03
1106	*Staphylococcus aureus*	0.5	32		0.5	1	16	0.12	1	≤0.06	0.5	1	4	16	0.25		0.03	1		0.12		0.12	0.12		0.06
1107	*Staphylococcus aureus*	0.5	1		0.5	1	1	0.06	0.5	≤0.06	0.25	1	2	8	0.25		0.03	1		0.12		0.12	0.12		0.25
1108	*Staphylococcus aureus*	0.5	≤0.03		0.12	0.5	0.06	0.12	0.5	≤0.06	0.5	1	2	16	0.25		0.06	1		0.12		0.12	0.25		0.06
1109	*Staphylococcus aureus*	0.5	64		32	>64	>32	>16	0.5	≤0.06	0.5	>64	>32	>64	4		1	2		>32		0.25	0.12		0.06
1110	*Staphylococcus aureus*	0.5	4		0.25	0.5	0.5	0.12	1	≤0.06	0.5	1	2	2	0.12		0.03	4		0.25		0.25	0.12		0.06
1111	*Staphylococcus aureus*	0.5	≤0.03		0.25	0.5	0.06	0.12	1	≤0.06	0.5	1	2	4	0.12		0.03	2		0.25		0.12	0.12		0.06
1112	*Staphylococcus aureus*	0.5	32		0.5	0.5	32	0.12	1	≤0.06	0.5	1	2	4	0.25		0.06	2		0.25		0.25	0.25		0.06
1113	*Staphylococcus aureus*	2	4		1	1	2	0.06	1	≤0.06	0.5	1	2	2	0.12		0.03	2		0.25		0.12	0.06		0.06
1114	*Staphylococcus aureus*	0.5	32		1	1	16	0.12	1	≤0.06	0.5	1	2	4	0.25		0.06	4		0.25		0.12	0.12		0.06
1115	*Staphylococcus aureus*	1	32		0.5	0.5	16	0.06	1	0.12	0.5	1	2	4	0.12		0.015	2		>32		0.12	0.12		0.06
1116	*Staphylococcus aureus*	1	2		0.12	0.5	0.25	0.12	1	0.25	1	1	2	1	0.25		0.03	2		0.25		0.25	0.06		0.06
1117	*Staphylococcus aureus*	2	1		0.5	0.5	1	0.06	1	≤0.06	0.5	0.5	2	2	0.12		0.06	2		0.25		0.12	0.12		0.06
1118	*Staphylococcus aureus*	1	32		0.5	0.5	8	0.12	1	≤0.06	0.5	1	2	2	0.12		0.015	2		>32		0.25	0.06		0.06
1119	*Staphylococcus aureus*	0.5	0.06		0.12	0.5	0.12	0.06	1	0.12	0.5	1	2	4	0.25		0.06	2		0.25		0.12	0.06		0.06
1120	*Staphylococcus aureus*	0.5	4		0.5	0.5	1	0.12	1	≤0.06	0.25	1	2	1	0.12		0.03	2		0.25		0.12	0.12		0.06
1121	*Staphylococcus aureus*	0.5	8		0.5	1	4	0.06	1	≤0.06	0.5	2	4	16	0.25		0.06	2		0.25		0.25	0.25		0.06
1122	*Staphylococcus aureus*	0.5	0.03		0.12	0.5	0.12	0.12	1	≤0.06	0.5	1	2	16	0.12		0.06	2		>32		0.12	0.12		0.06
1123	*Staphylococcus aureus*	0.5	0.06		0.25	0.5	0.12	0.25	1	≤0.06	0.5	1	2	8	0.25		0.03	2		>32		0.12	0.06		0.06
1124	*Staphylococcus aureus*	0.5	0.06		0.12	0.5	0.12	0.06	1	≤0.06	0.5	1	4	8	0.5		0.12	2		>32		64	0.12		0.06
1125	*Staphylococcus aureus*	0.25	64		1	1	32	0.12	1	≤0.06	0.25	1	2	16	0.12		0.03	4		0.25		0.25	0.12		0.06
1126	*Staphylococcus aureus*	0.5	32		1	1	16	0.12	1	≤0.06	0.5	1	2	16	0.25		0.06	4		0.25		0.25	0.12		0.06
1127	*Staphylococcus aureus*	0.25	4		1	1	2	0.12	1	≤0.06	1	2	4	32	>8		4	4		0.25		0.12	0.12		0.06
1128	*Staphylococcus aureus*	1	32		2	1	8	0.06	0.5	0.12	0.5	1	2	16	0.25		0.06	1		0.12		0.12	0.12		0.03

Isolate Number	*Staphylococcus* species	MIC (µg/ml) Values																							
			β Lactams					Carbapenem	Glycopeptides			Cephalosporins			Flouroquinolones			Oxazolidnone	Macrolides			Tetracyclines		Aminoglycoside	Antifolate
		XF-73	Penicillin	Oxacillin	Amoxicillin Clavulanate	Piperacillin/Tazobactam	Ampicillin	Meropenem	Vancomycin	Dalbavancin	Teicoplanin	Cefuroxime	Ceftriaxone	Ceftazidime	Levofloxacin	Ciprofloxacin	Moxifloxacin	Linezolid	Clindamycin	Clarithromycin	Erythromycin	Tetracycline	Tigecycline	Gentamicin	Trimethoprim/ Sulfmethoxazole
1129	*Staphylococcus aureus*	0.5	64		0.5	1	8	0.12	1	≤0.06	1	2	4	8	0.12		0.015	2		8		0.25	0.25		0.06
1130	*Staphylococcus aureus*	1	0.03		0.12	0.5	0.03	0.12	0.5	0.12	0.5	4	4	4	0.25		0.06	2		0.12		0.25	0.12		0.06
1131	*Staphylococcus aureus*	0.5	64		0.5	0.5	>32	0.12	1	0.12	0.5	1	2	2	0.25		0.06	2		0.12		0.5	0.25		0.12
1132	*Staphylococcus aureus*	1	0.03		0.12	0.5	0.03	0.06	0.5	≤0.06	0.5	1	2	4	0.25		0.03	2		0.12		0.25	0.12		0.06
1133	*Staphylococcus aureus*	1	16		0.5	1	8	0.06	0.5	≤0.06	0.25	1	2	4	0.25		0.06	2		0.12		0.25	0.12		0.06
1134	*Staphylococcus aureus*	0.25	16		0.5	1	8	0.06	1	≤0.06	0.5	1	2	4	0.25		0.06	2		0.25		0.25	0.12		0.06
1135	*Staphylococcus aureus*	0.5	1		0.5	1	2	0.12	0.5	≤0.06	0.25	2	4	4	0.25		0.06	2		0.12		0.12	0.12		0.06
1136	*Staphylococcus aureus*	1	≤0.03		0.06	0.5	0.03	0.12	1	≤0.06	0.25	1	2	4	0.25		0.06	2		0.12		0.25	0.12		0.12
1137	*Staphylococcus aureus*	0.25	16		0.5	1	16	0.12	1	0.12	0.5	1	4	2	0.12		0.06	1		0.12		0.12	0.12		0.06
1138	*Staphylococcus aureus*	0.5	16		1	2	8	0.12	0.5	≤0.06	0.25	2	4	2	4		1	1		32		0.25	0.25		0.06
1139	*Staphylococcus aureus*	0.5	8		0.12	0.5	0.03	0.12	0.5	≤0.06	0.25	2	4	16	0.25		0.06	2		0.12		0.25	0.12		0.06
1140	*Staphylococcus aureus*	1	0.03		0.12	0.5	0.12	0.12	1	≤0.06	0.5	1	4	8	0.25		0.06	2		0.25		0.25	0.12		0.12
1141	*Staphylococcus aureus*	0.5	16		1	1	8	0.06	1	≤0.06	0.5	1	4	8	0.12		0.06	2		0.25		0.25	0.12		0.25
1142	*Staphylococcus aureus*	1	4		0.5	1	2	0.12	1	≤0.06	0.25	1	2	8	0.06		0.03	2		0.25		0.25	0.06		0.25
1143	*Staphylococcus aureus*	1	0.03		0.12	1	0.12	0.12	1	≤0.06	0.5	2	4	8	0.12		0.03	2		0.25		0.25	0.12		0.06
1144	*Staphylococcus aureus*	0.5	32		1	1	32	0.25	1	0.25	0.5	2	4	8	0.25		0.06	4		0.25		0.5	0.12		0.25
1145	*Staphylococcus aureus*	0.25	0.12		0.25	2	0.25	0.12	1	0.12	0.25	1	4	8	0.25		0.06	2		0.25		0.25	0.12		0.06
1146	*Staphylococcus aureus*	1	2		1	1	2	0.25	1	≤0.06	0.5	2	4	8	0.25		0.06	2		0.25		0.25	0.12		0.12
1147	*Staphylococcus aureus*	0.5	4		0.25	1	1	0.12	1	0.12	0.5	1	4	8	0.25		0.03	2		0.25		0.25	0.12		0.06
1148	*Staphylococcus aureus*	0.25	4		0.5	1	4	0.12	1	0.12	0.5	1	4	8	0.12		0.03	2		0.25		0.25	0.12		0.12
1149	*Staphylococcus aureus*	0.5	16		0.5	0.5	32	0.06	1	≤0.06	0.5	1	2	4	0.12		0.015	2		0.12		0.25	0.12		0.06
1150	*Staphylococcus aureus*	0.5	≤0.03		0.25	0.5	0.12	0.12	1	≤0.06	0.25	1	2	8	0.12		0.06	2		0.25		0.25	0.06		0.12
1151	*Staphylococcus aureus*	0.5	0.03		0.25	0.5	0.12	0.12	1	≤0.06	0.5	1	4	8	0.25		0.06	4		0.25		0.5	0.12		0.5
1152	*Staphylococcus aureus*	0.5	≤0.03		0.12	1	0.06	0.12	1	≤0.06	0.5	2	4	8	8		2	2		0.12		0.12	0.12		0.06
1153	*Staphylococcus aureus*	0.5	≤0.03		0.06	0.25	0.03	0.06	1	≤0.06	0.5	1	2	4	0.25		0.06	2		0.25		0.25	0.12		0.06
1154	*Staphylococcus aureus*	0.5	32		0.5	1	16	0.12	1	≤0.06	0.5	2	2	8	0.12		0.03	1		0.12		0.12	0.06		0.06
1155	*Staphylococcus aureus*	0.5	32		0.5	1	32	0.12	1	≤0.06	1	1	2	4	0.25		0.06	2		0.25		0.12	0.12		0.06
1156	*Staphylococcus aureus*	0.5	32		1	2	32	0.12	1	≤0.06	0.5	2	4	8	0.25		0.03	2		0.25		0.25	0.25		0.06
1157	*Staphylococcus aureus*	0.5	4		0.5	1	2	0.12	1	≤0.06	0.5	2	4	8	0.25		0.12	2		0.25		0.5	0.12		0.06
1158	*Staphylococcus aureus*	0.5	64		0.5	1	32	0.12	1	≤0.06	0.5	2	4	8	0.25		0.03	2		0.12		0.25	0.12		0.06
1159	*Staphylococcus aureus*	0.25	64		0.5	1	32	0.12	1	≤0.06	0.5	1	2	8	0.12		0.03	2		0.12		0.5	0.12		0.06
1160	*Staphylococcus aureus*	0.5	0.03		0.12	0.5	0.06	0.12	2	≤0.06	0.5	1	2	8	0.25		0.06	2		0.12		0.25	0.12		0.06
1161	*Staphylococcus aureus*	0.5	32		0.25	0.5	16	0.06	1	≤0.06	0.5	1	2	8	0.12		0.03	2		0.12		0.12	0.06		0.06
1162	*Staphylococcus aureus*	0.5	1		0.5	1	1	0.12	1	≤0.06	0.5	2	4	8	0.25		0.12	2		0.25		0.25	0.25		0.06
1163	*Staphylococcus aureus*	0.5	0.03		0.25	0.5	0.06	0.06	1	≤0.06	0.5	1	2	8	>8		2	2		0.25		0.12	0.06		0.06
1164	*Staphylococcus aureus*	0.5	64		1	1	32	0.12	1	≤0.06	0.5	2	4	16	0.25		0.06	2		0.25		0.5	0.12		0.06
1165	*Staphylococcus aureus*	0.5	≤0.03		0.12	0.5	0.06	0.06	1	≤0.06	0.5	1	2	8	0.12		0.06	2		0.25		0.5	0.12		0.06
1166	*Staphylococcus aureus*	0.25	0.5		0.5	1	1	0.12	1	0.12	0.5	2	4	16	>8		4	4		>32		0.25	0.12		0.06
1167	*Staphylococcus aureus*	0.5	0.06		0.25	1	0.12	0.12	1	≤0.06	2	2	8	16	0.25		0.06	4		0.25		0.5	0.25		0.12
1168	*Staphylococcus aureus*	0.5	0.12		0.25	1	0.25	0.12	2	≤0.06	0.5	1	4	8	0.12		0.03	2		0.25		0.25	0.12		0.12
1169	*Staphylococcus aureus*	0.5	0.5		0.5	1	1	0.06	1	≤0.06	0.5	2	4	8	>8		4	2		>32		0.25	0.12		0.06
1170	*Staphylococcus aureus*	1	≤0.03		0.25	0.5	0.06	0.06	1	≤0.06	0.5	1	2	8	0.25		0.06	2		0.25		0.25	0.12		0.06
1171	*Staphylococcus aureus*	0.5	1		0.5	1	1	0.06	1	≤0.06	0.5	2	4	16	>8		4	4		>32		0.25	0.12		0.06
1172	*Staphylococcus aureus*	0.5	0.25		0.25	1	0.25	0.12	1	≤0.06	0.5	2	4	16	0.12		0.03	2		0.25		0.25	0.12		0.06
1173	*Staphylococcus aureus*	1	16		1	1	8	0.12	1	≤0.06	0.5	1	2	16	0.12		0.03	2		0.25		0.25	0.12		0.06
1174	*Staphylococcus aureus*	2	0.25		0.25	1	0.25	0.12	1	≤0.06	0.5	2	4	16	0.12		0.06	2		0.25		0.12	0.12		0.06
1175	*Staphylococcus aureus*	0.5	8		1	1	8	0.12	1	≤0.06	0.25	1	4	8	0.25		0.06	2		0.25		0.25	0.25		0.06

		MIC (µg/ml) Values																							
			β Lactams					Carbapenem	Glycopeptides			Cephalosporins			Flouroquinolones			Oxazolidnone	Macrolides			Tetracyclines		Aminoglycoside	Antifolate
Isolate Number	***Staphylococcus* species**	XF-73	Penicillin	Oxacillin	Amoxicillin Clavulanate	Piperacillin/Tazobactam	Ampicillin	Meropenem	Vancomycin	Dalbavancin	Teicoplanin	Cefuroxime	Ceftriaxone	Ceftazidime	Levofloxacin	Ciprofloxacin	Moxifloxacin	Linezolid	Clindamycin	Clarithromycin	Erythromycin	Tetracycline	Tigecycline	Gentamicin	Trimethoprim/ Sulfmethoxazole
1176	*Staphylococcus aureus*	0.5	≤0.03		0.12	0.5	0.12	0.06	0.5	0.12	0.5	1	2	8	0.25		0.06	2		0.25		0.12	0.12		0.06
1177	*Staphylococcus aureus*	1	32		1	2	32	0.06	1	≤0.06	0.5	2	4	16	0.5		0.06	2		0.25		0.12	0.12		0.06
1178	*Staphylococcus aureus*	2	0.12		0.25	1	0.12	0.12	1	≤0.06	0.5	2	4	16	>8		2	2		>32		0.25	0.12		0.06
1179	*Staphylococcus aureus*	0.5	4		0.5	2	2	0.06	0.5	≤0.06	0.25	2	4	16	0.12		0.03	2		0.25		0.12	0.06		0.12
1180	*Staphylococcus aureus*	0.5	2		0.5	1	1	0.12	1	≤0.06	0.5	2	4	16	0.5		0.12	2		0.25		0.5	0.5		0.06
1181	*Staphylococcus aureus*	0.5	4		0.5	1	4	0.12	1	≤0.06	0.5	1	2	8	>8		4	2		>32		0.25	0.12		0.12
1182	*Staphylococcus aureus*	0.25	2		0.5	0.5	1	0.12	1	≤0.06	0.5	1	2	8	0.5		0.06	2		0.25		0.25	0.25		2
1183	*Staphylococcus aureus*	0.5	0.06		0.25	1	0.12	0.12	1	≤0.06	0.5	2	4	16	0.25		0.03	2		>32		0.25	0.12		0.06
1184	*Staphylococcus aureus*	1	32		1	2	32	0.12	1	≤0.06	0.25	2	4	16	0.5		0.12	2		>32		0.25	0.12		0.03
1185	*Staphylococcus aureus*	0.5	0.5		0.25	0.5	1	0.06	2	≤0.06	0.5	1	2	8	0.25		0.03	2		0.25		0.12	0.12		0.12
1186	*Staphylococcus aureus*	0.5	16		1	1	16	0.12	2	≤0.06	0.5	1	4	8	0.25		0.06	2		1		0.25	0.12		0.06
1187	*Staphylococcus aureus*	1	1		0.5	1	1	0.12	1	≤0.06	1	1	2	8	0.25		0.06	2		>32		0.12	0.12		0.06
1188	*Staphylococcus aureus*	1	16		0.5	1	32	0.12	1	0.12	0.5	1	4	8	0.12		0.03	2		0.25		0.25	0.12		0.12
1189	*Staphylococcus aureus*	0.5	32		1	2	8	0.12	1	≤0.06	0.5	2	4	8	0.25		0.06	2		0.25		0.25	0.12		0.12
1190	*Staphylococcus aureus*	0.5	0.06		0.25	0.5	0.25	0.12	1	≤0.06	0.5	1	2	4	0.25		0.03	2		0.25		0.12	0.06		0.06
1191	*Staphylococcus aureus*	2	16		0.5	1	32	0.12	1	0.12	0.5	1	4	8	0.12		0.03	2		0.25		0.25	0.12		0.12
1192	*Staphylococcus aureus*	1	16		1	1	16	0.25	1	≤0.06	0.5	2	4	32	0.5		0.12	2		0.25		0.25	0.12		0.06
1193	*Staphylococcus aureus*	0.5	0.06		0.25	1	0.25	0.06	1	≤0.06	1	1	4	8	8		1	16		1		0.25	0.25		0.06
1194	*Staphylococcus aureus*	0.5	0.12		0.25	0.5	0.25	0.06	1	≤0.06	0.5	2	4	16	0.25		0.06	2		0.25		0.25	0.12		0.06
1195	*Staphylococcus aureus*	0.5	1		0.5	1	1	0.12	1	≤0.06	0.5	1	4	8	0.25		0.03	1		0.5		0.25	0.12		0.06
1196	*Staphylococcus aureus*	0.5	16		1	1	8	0.06	0.5	0.12	0.25	2	4	8	0.5		0.03	2		0.25		0.25	0.12		0.06
1197	*Staphylococcus aureus*	0.5	16		1	1	16	0.12	1	≤0.06	0.25	1	4	8	0.12		0.03	2		32		0.25	0.12		0.12
1198	*Staphylococcus aureus*	0.5	0.06		0.25	1	0.12	0.25	1	≤0.06	0.5	1	4	16	0.5		0.06	2		0.25		1	0.12		0.12
1199	*Staphylococcus aureus*	0.5	0.5		0.25	1	0.5	0.06	1	≤0.06	0.5	2	4	8	0.25		0.03	2		0.25		0.25	0.06		0.12
1200	*Staphylococcus aureus*	0.5	0.5		0.5	2	1	0.12	1	0.25	0.25	2	4	32	0.12		0.03	2		0.25		0.25	0.12		0.06
1201	*Staphylococcus aureus*	0.5	64		0.5	1	32	0.12	1	≤0.06	0.5	2	4	8	0.12		0.03	2		0.25		0.25	0.12		0.12
1202	*Staphylococcus aureus*	0.5	8		1	1	4	0.12	0.5	≤0.06	0.5	1	4	8	0.25		0.06	2		0.12		0.25	0.25		0.06
1203	*Staphylococcus aureus*	0.5	8		1	1	8	0.12	0.5	≤0.06	0.25	1	4	8	0.12		0.03	2		0.12		0.12	0.12		0.06
1204	*Staphylococcus aureus*	0.5	4		1	2	4	0.06	1	≤0.06	0.5	2	4	8	0.12		0.03	4		0.25		0.5	0.12		0.06
1205	*Staphylococcus aureus*	0.5	0.06		0.25	1	0.06	0.06	0.5	≤0.06	0.5	1	4	8	0.12		0.015	2		0.25		0.12	0.06		0.06
1206	*Staphylococcus aureus*	0.5	4		1	1	2	0.12	0.5	≤0.06	0.25	2	4	8	0.25		0.03	2		0.12		0.12	0.12		0.06
1207	*Staphylococcus aureus*	0.5	4		1	1	4	0.12	0.5	≤0.06	0.25	1	4	8	0.25		0.03	4		0.25		0.12	0.12		0.06
1208	*Staphylococcus aureus*	0.5	0.25		0.25	1	0.5	0.06	1	0.12	0.5	1	2	8	0.06		0.015	2		0.12		0.5	0.25		0.03
1209	*Staphylococcus aureus*	0.5	4		0.5	1	2	0.06	0.5	≤0.06	0.5	1	2	8	0.5		0.06	2		0.12		0.12	0.12		0.06
1210	*Staphylococcus aureus*	0.5	2		1	1	2	0.06	0.5	≤0.06	0.25	1	4	8	0.12		0.03	1		0.12		0.12	0.12		0.06
1211	*Staphylococcus aureus*	0.5	1		0.5	1	1	0.06	1	≤0.06	0.5	1	2	4	0.12		0.03	2		32		0.12	0.06		0.06
1212	*Staphylococcus aureus*	0.5	0.03		0.25	1	0.06	0.06	0.5	≤0.06	0.5	1	4	8	0.12		0.015	2		0.12		0.25	0.12		0.03
1213	*Staphylococcus aureus*	0.5	32		1	1	32	0.12	0.5	0.12	0.25	1	2	8	0.06		0.008	2		4		0.12	0.06		0.03
1214	*Staphylococcus aureus*	0.5	0.06		0.06	0.25	0.12	0.06	1	≤0.06	0.5	1	2	8	0.5		0.03	4		0.25		0.12	0.12		0.06
1215	*Staphylococcus aureus*	1	1		0.5	1	1	0.06	1	≤0.06	1	1	2	16	0.5		0.06	4		0.25		0.25	0.12		0.06
1216	*Staphylococcus aureus*	1	1		0.25	0.5	1	0.06	1	≤0.06	0.5	1	2	8	0.12		0.06	2		0.25		0.25	0.12		0.06
1217	*Staphylococcus aureus*	1	0.5		0.5	0.5	1	0.06	1	0.25	0.5	1	2	16	0.12		0.03	4		0.25		0.12	0.06		0.06
1218	*Staphylococcus aureus*	0.5	16		0.5	1	16	0.06	1	≤0.06	0.5	1	2	8	0.25		0.06	2		0.25		0.12	0.06		0.06
1219	*Staphylococcus aureus*	0.5	0.06		0.12	0.5	0.12	0.06	1	≤0.06	0.5	1	2	16	0.25		0.06	4		0.25		0.25	0.12		0.06
1220	*Staphylococcus aureus*	0.5	0.12		0.12	0.5	0.25	0.06	1	0.12	0.5	1	2	16	0.12		0.03	2		0.25		0.12	0.12		0.06
1221	*Staphylococcus aureus*	1	4		0.5	1	4	0.12	1	≤0.06	0.25	1	2	8	0.25		0.06	2		0.25		0.12	0.12		0.06
1222	*Staphylococcus aureus*	1	0.06		0.12	0.5	0.12	0.06	1	≤0.06	0.5	1	4	8	0.25		0.06	2		0.25		0.12	0.12		0.06

Isolate Number	*Staphylococcus* species	MIC (µg/ml) Values																							
			β Lactams					Carbapenem	Glycopeptides			Cephalosporins			Flouroquinolones			Oxazolidnone	Macrolides			Tetracyclines		Aminoglycoside	Antifolate
		XF-73	Penicillin	Oxacillin	Amoxicillin Clavulanate	Piperacillin/Tazobactam	Ampicillin	Meropenem	Vancomycin	Dalbavancin	Teicoplanin	Cefuroxime	Ceftriaxone	Ceftazidime	Levofloxacin	Ciprofloxacin	Moxifloxacin	Linezolid	Clindamycin	Clarithromycin	Erythromycin	Tetracycline	Tigecycline	Gentamicin	Trimethoprim/ Sulfmethoxazole
1223	*Staphylococcus aureus*	0.5	0.06		0.25	0.5	0.12	0.12	2	0.12	0.5	1	2	8	0.25		0.06	2		0.25		0.25	0.12		0.06
1224	*Staphylococcus aureus*	1	16		0.5	1	8	0.06	1	≤0.06	1	1	2	8	0.25		0.06	2		0.25		0.12	0.12		0.06
1225	*Staphylococcus aureus*	1	0.25		0.25	0.5	0.25	0.12	2	≤0.06	0.5	1	2	16	0.25		0.06	2		0.25		0.25	0.12		0.06
1226	*Staphylococcus aureus*	0.5	8		0.5	1	8	0.06	0.5	≤0.06	0.25	1	2	4	0.12		0.015	2		0.12		0.25	0.12		0.06
1227	*Staphylococcus aureus*	0.5	16		0.5	1	8	0.06	0.5	≤0.06	0.25	1	2	8	0.12		0.015	2		0.12		0.12	0.12		0.06
1228	*Staphylococcus aureus*	1	2		0.5	1	2	0.06	0.5	0.12	0.25	1	4	8	0.25		0.06	2		0.12		0.12	0.12		0.06
1229	*Staphylococcus aureus*	1	0.06		0.25	1	0.06	0.06	0.5	0.25	0.5	1	4	16	0.5		0.06	2		0.12		0.12	0.12		0.06
1230	*Staphylococcus aureus*	0.5	1		0.25	1	0.5	0.06	0.5	≤0.06	0.5	1	8	8	0.25		0.03	2		0.12		0.12	0.12		0.06
1231	*Staphylococcus aureus*	0.5	16		0.5	1	4	0.12	0.5	0.12	0.5	1	2	4	0.25		0.03	1		0.12		0.12	0.06		0.06
1232	*Staphylococcus aureus*	1	0.06		0.12	0.25	0.06	0.12	0.5	≤0.06	0.25	1	4	8	0.25		0.03	2		0.12		0.12	0.12		0.12
1233	*Staphylococcus aureus*	0.5	8		1	2	4	0.06	0.5	≤0.06	0.5	2	4	16	0.25		0.06	2		0.12		0.25	0.12		0.06
1234	*Staphylococcus aureus*	0.5	4		1	2	2	0.12	0.5	≤0.06	0.5	1	4	8	0.25		0.06	4		0.25		0.25	0.25		0.12
1235	*Staphylococcus aureus*	0.5	0.06		0.12	0.5	0.06	0.06	0.5	≤0.06	0.5	1	2	8	0.25		0.03	2		0.12		0.25	0.12		0.06
1236	*Staphylococcus aureus*	1	2		1	1	2	0.12	0.5	≤0.06	0.25	1	4	8	0.12		0.015	2		0.12		16	0.25		0.06
1237	*Staphylococcus aureus*	4	0.03		0.12	0.5	0.06	0.06	0.5	≤0.06	0.25	1	4	8	0.12		0.03	2		0.12		0.25	0.12		0.06
1238	*Staphylococcus aureus*	0.5	1		0.5	2	1	0.06	0.5	≤0.06	0.25	2	4	8	0.25		0.03	2		0.12		0.12	0.12		0.06
1239	*Staphylococcus aureus*	2	8		1	1	4	0.06	1	≤0.06	1	1	2	8	0.25		0.06	2		0.25		0.25	0.12		0.06
1240	*Staphylococcus aureus*	0.5	0.12		0.12	0.5	0.12	0.03	0.5	≤0.06	0.25	1	2	4	0.12		0.03	2		0.12		0.12	0.12		0.12
1241	*Staphylococcus aureus*	0.5	0.06		0.12	0.5	0.12	0.06	0.5	0.12	0.06	0.5	1	8	0.25		0.03	2		0.12		0.25	0.12		0.06
1242	*Staphylococcus aureus*	0.5	1		1	1	1	0.12	0.5	≤0.06	0.5	2	4	16	0.25		0.06	2		0.25		0.25	0.12		0.12
1243	*Staphylococcus aureus*	0.5	1		1	1	2	0.06	0.5	≤0.06	0.25	1	4	8	0.25		0.03	1		0.12		0.12	0.12		0.06
1244	*Staphylococcus aureus*	1	4		0.5	1	2	0.06	0.5	≤0.06	0.5	1	2	8	0.5		0.12	2		0.12		0.25	0.12		0.12
1245	*Staphylococcus aureus*	0.5	0.06		0.25	1	0.06	0.12	0.5	≤0.06	0.25	1	2	4	0.12		0.015	2		0.12		0.25	0.12		0.12
1246	*Staphylococcus aureus*	0.5	32		0.5	1	16	0.12	0.5	≤0.06	0.25	2	4	8	0.12		0.03	2		0.12		0.25	0.12		0.12
1247	*Staphylococcus aureus*	0.5	8		1	2	4	0.12	0.5	0.12	0.25	2	4	16	0.25		0.03	2		16		0.25	0.12		0.06
1248	*Staphylococcus aureus*	0.5	0.06		0.25	1	0.12	0.06	1	≤0.06	0.5	1	4	16	0.25		0.06	2		0.25		0.25	0.12		0.25
1249	*Staphylococcus aureus*	2	0.06		0.25	1	0.25	0.25	1	≤0.06	0.5	2	4	16	0.25		0.06	2		>32		0.12	0.12		0.06
1250	*Staphylococcus aureus*	0.5	32		0.5	1	16	0.12	1	0.12	0.5	2	4	8	0.25		0.03	2		0.25		32	1		0.06
1251	*Staphylococcus aureus*	0.5	2		1	1	2	0.12	1	≤0.06	0.5	2	4	16	0.25		0.03	2		0.25		0.5	0.5		0.06
1252	*Staphylococcus aureus*	1	8		1	1	8	0.06	1	≤0.06	0.5	2	4	8	0.12		0.03	2		0.25		0.25	0.12		0.06
1253	*Staphylococcus aureus*	0.5	4		0.5	1	1	0.12	1	≤0.06	1	1	4	8	0.25		0.12	2		0.25		0.25	0.25		0.06
1254	*Staphylococcus aureus*	0.5	32		1	2	32	0.25	1	≤0.06	0.5	2	4	16	0.25		0.03	2		0.25		0.25	0.25		0.12
1255	*Staphylococcus aureus*	0.5	0.06		0.25	1	0.12	0.06	1	≤0.06	0.5	1	4	8	4		1	2		0.25		0.25	0.25		>32
1256	*Staphylococcus aureus*	0.5	32		0.5	1	16	0.12	1	≤0.06	1	2	4	8	0.5		0.06	2		0.5		0.5	0.25		0.06
1257	*Staphylococcus aureus*	0.5	4		1	2	4	0.25	1	≤0.06	1	2	8	16	0.25		0.06	2		0.25		0.25	0.25		0.06
1258	*Staphylococcus aureus*	0.5	0.12		0.25	0.5	0.25	0.12	1	≤0.06	0.5	1	4	8	0.25		0.03	2		0.25		0.25	0.25		0.06
1259	*Staphylococcus aureus*	0.5	0.06		0.12	1	0.06	0.12	1	≤0.06	0.5	1	4	16	0.12		0.03	2		0.25		0.25	0.12		0.12
1260	*Staphylococcus aureus*	0.5	0.03		0.25	1	0.12	0.06	0.5	≤0.06	0.5	1	4	8	8		2	1		>32		0.25	0.12		0.03
1261	*Staphylococcus aureus*	0.5	0.25		0.25	1	0.5	0.12	1	≤0.06	0.5	2	4	16	0.25		0.06	2		32		0.25	0.25		0.12
1262	*Staphylococcus aureus*	0.5	0.03		0.12	0.5	0.06	0.06	1	≤0.06	0.5	2	4	8	0.25		0.03	2		>32		32	0.25		0.5
1263	*Staphylococcus aureus*	0.5	0.06		0.25	1	0.06	0.12	1	≤0.06	0.5	4	4	8	0.25		0.06	2		0.25		0.25	0.12		0.12
1264	*Staphylococcus aureus*	0.5	4		1	2	4	0.12	0.5	≤0.06	0.5	2	4	8	0.25		0.06	2		>32		16	0.25		0.06
1265	*Staphylococcus aureus*	0.5	32		0.5	1	16	0.12	0.5	≤0.06	0.25	2	4	16	0.12		0.03	2		0.25		0.25	0.12		0.12
1266	*Staphylococcus aureus*	0.5	32		1	1	16	0.12	0.5	≤0.06	0.5	1	8	8	0.5		0.03	2		>32		0.25	0.12		0.12
1267	*Staphylococcus aureus*	0.5	0.03		0.25	0.5	0.12	0.06	0.5	≤0.06	0.5	1	4	8	0.12		0.03	2		0.25		0.25	0.12		0.06
1268	*Staphylococcus aureus*	1	32		0.5	0.5	16	0.06	1	≤0.06	1	1	2	8	0.25		0.06	2		0.25		0.25	0.12		0.12
1269	*Staphylococcus aureus*	0.5	0.06		0.12	1	0.06	0.06	0.5	≤0.06	0.25	4	4	16	0.12		0.03	2		0.25		0.25	0.12		0.12

Table s2: MIC Data for the Staphylococcus species tested

Isolate Number	*Staphylococcus* species	MIC (µg/ml) Values																							
			β Lactams					Carbapenem	Glycopeptides			Cephalosporins			Flouroquinolones			Oxazolidnone	Macrolides			Tetracyclines		Aminoglycoside	Antifolate
		XF-73	Penicillin	Oxacillin	Amoxicillin Clavulanate	Piperacillin/Tazobactam	Ampicillin	Meropenem	Vancomycin	Dalbavancin	Teicoplanin	Cefuroxime	Ceftriaxone	Ceftazidime	Levofloxacin	Ciprofloxacin	Moxifloxacin	Linezolid	Clindamycin	Clarithromycin	Erythromycin	Tetracycline	Tigecycline	Gentamicin	Trimethoprim/ Sulfmethoxazole
1270	*Staphylococcus aureus*	1	0.03		0.12	0.5	0.06	0.06	0.5	≤0.06	0.25	1	2	8	0.25		0.06	1		>32		0.25	0.12		0.12
1271	*Staphylococcus aureus*	0.5	2		0.5	1	1	0.12	0.5	≤0.06	0.25	1	4	16	0.25		0.06	2		0.25		0.25	0.25		0.12
1272	*Staphylococcus aureus*	0.5	4		1	2	4	0.06	1	≤0.06	0.5	8	4	8	0.25		0.03	2		0.5		0.25	0.12		0.06
1273	*Staphylococcus aureus*	1	4		1	2	2	0.25	1	0.25	0.5	2	8	8	0.5		0.06	2		>32		0.25	0.12		0.12
1274	*Staphylococcus aureus*	1	32		1	2	16	0.12	2	≤0.06	1	2	4	16	0.5		0.06	2		0.25		1	0.25		0.12
1275	*Staphylococcus aureus*	0.5	0.06		0.25	1	0.12	0.06	0.25	0.12	0.25	1	4	8	8		2	2		>32		1	0.12		0.06
1276	*Staphylococcus aureus*	0.25	8		1	1	8	0.12	0.5	≤0.06	0.25	2	4	2	0.25		0.03	2		16		0.25	0.25		0.06
1277	*Staphylococcus aureus*	0.5	0.03		0.12	1	0.25	0.12	0.5	≤0.06	0.5	2	4	16	0.12		0.03	2		0.12		32	0.25		0.03
1278	*Staphylococcus aureus*	0.5	8		0.5	1	4	0.06	0.5	≤0.06	0.5	1	2	8	0.12		0.03	2		0.12		0.25	0.12		0.06
1279	*Staphylococcus aureus*	0.5	0.03		0.12	1	0.12	0.12	1	≤0.06	0.5	4	2	16	0.25		0.06	2		0.12		0.25	0.25		0.06
1280	*Staphylococcus aureus*	0.25	0.5		0.25	1	1	0.12	1	≤0.06	0.5	1	2	16	0.5		0.12	2		0.25		0.25	0.12		0.06
1281	*Staphylococcus aureus*	0.25	0.5		0.5	1	1	0.12	1	≤0.06	0.5	1	2	16	0.12		0.06	2		0.25		0.25	0.25		0.03
1282	*Staphylococcus aureus*	0.25	1		0.5	2	1	0.12	1	0.25	0.5	2	4	16	0.25		0.06	2		32		0.25	0.25		0.12
1283	*Staphylococcus aureus*	0.5	8		0.5	2	4	0.12	1	≤0.06	0.5	1	2	16	0.12		0.03	2		0.12		0.12	0.12		0.06
1284	*Staphylococcus aureus*	0.5	0.03		0.25	1	0.12	0.12	1	≤0.06	0.5	1	2	16	0.25		0.06	4		0.12		0.25	0.12		0.06
1285	*Staphylococcus aureus*	0.25	2		0.5	2	2	0.12	1	≤0.06	0.5	2	4	16	0.25		0.12	2		>32		0.5	0.12		0.06
1286	*Staphylococcus aureus*	0.5	16		1	1	8	0.12	1	≤0.06	0.5	2	2	16	0.12		0.03	2		0.25		0.25	0.12		0.06
1287	*Staphylococcus aureus*	0.5	0.5		0.25	0.5	1	0.12	2	≤0.06	1	1	2	8	0.25		0.06	4		0.25		0.12	0.12		0.06
1288	*Staphylococcus aureus*	0.5	0.12		0.5	1	0.06	0.06	1	0.12	0.5	2	4	8	0.25		0.06	2		>32		0.25	0.12		0.5
1289	*Staphylococcus aureus*	0.25	0.5		0.25	1	0.25	0.12	1	≤0.06	0.5	1	4	16	0.5		0.06	2		0.25		0.5	0.25		0.5
1290	*Staphylococcus aureus*	0.5	8		1	2	8	0.12	0.5	≤0.06	0.5	2	4	8	0.25		0.06	2		0.25		0.25	0.12		0.12
1291	*Staphylococcus aureus*	0.5	64		0.5	1	32	0.12	0.5	≤0.06	0.25	16	4	16	1		0.06	2		0.25		0.25	0.12		0.12
1292	*Staphylococcus aureus*	0.5	32		2	4	32	0.12	1	≤0.06	0.5	2	8	32	0.25		0.06	2		0.25		0.5	0.5		0.12
1293	*Staphylococcus aureus*	0.5	0.5		0.5	1	0.5	0.06	1	0.12	0.5	2	4	8	0.25		0.06	2		0.5		0.25	0.12		0.06
1294	*Staphylococcus aureus*	0.25	4		1	2	4	0.12	0.5	≤0.06	0.25	2	16	32	0.25		0.03	2		0.25		0.25	0.25		0.12
1295	*Staphylococcus aureus*	0.25	4		1	1	2	0.12	0.5	≤0.06	0.5	2	16	16	0.25		0.06	4		0.5		0.5	0.12		0.12
1296	*Staphylococcus aureus*	0.5	8		0.5	1	1	0.12	1	≤0.06	0.5	2	4	8	0.25		0.03	2		0.25		64	0.12		0.12
1297	*Staphylococcus aureus*	1	≤0.03		0.06	0.25	0.03	0.03	0.5	≤0.06	0.5	1	2	4	1		0.12	2		0.25		0.12	0.06		0.12
1298	*Staphylococcus aureus*	1	16		1	2	16	0.06	1	≤0.06	0.25	2	4	8	0.5		0.12	2		>32		0.5	0.12		0.25
1299	*Staphylococcus aureus*	1	2		1	1	2	0.12	0.5	0.25	0.5	2	4	8	0.12		0.03	2		0.25		0.25	0.12		0.06
1300	*Staphylococcus aureus*	0.5	64		4	16	32	0.12	1	≤0.06	0.5	8	8	8	1		0.06	4		>32		0.5	0.5		0.12
1301	*Staphylococcus aureus*	1	8		1	1	4	0.12	1	≤0.06	0.5	1	4	8	1		0.25	2		0.12		0.25	0.12		0.12
1302	*Staphylococcus aureus*	0.5	0.12		0.5	1	0.25	0.06	1	≤0.06	0.5	2	4	16	0.25		0.06	2		>32		0.25	0.25		0.12
1303	*Staphylococcus aureus*	0.5	0.5		0.5	1	0.25	0.12	1	≤0.06	0.25	1	2	8	0.12		0.06	2		0.25		0.5	0.5		0.06
1304	*Staphylococcus aureus*	0.5	1		0.5	1	0.5	0.12	0.25	≤0.06	0.5	1	4	8	0.12		0.03	2		0.25		0.25	0.25		0.12
1305	*Staphylococcus aureus*	0.5	1		0.5	1	0.25	0.12	1	≤0.06	0.5	1	4	8	0.12		0.03	2		>32		32	0.25		0.12
1306	*Staphylococcus aureus*	0.5	0.06		0.25	1	0.12	0.12	0.5	≤0.06	0.5	1	4	8	0.12		0.03	1		0.25		0.25	0.12		0.12
1307	*Staphylococcus aureus*	0.5	0.25		0.25	0.5	0.12	0.06	1	≤0.06	0.5	0.5	1	4	0.12		0.06	2		0.25		0.25	0.25		0.06
1308	*Staphylococcus aureus*	0.5	1		0.25	1	0.5	0.12	1	≤0.06	0.5	1	4	8	0.25		0.03	2		0.25		0.25	0.25		0.12
1309	*Staphylococcus aureus*	0.5	0.03		0.12	0.5	0.06	0.06	0.5	≤0.06	0.25	1	4	8	0.25		0.06	1		0.12		0.25	0.25		0.12
1310	*Staphylococcus aureus*	0.5	0.5		0.5	0.5	0.25	0.25	0.5	≤0.06	0.5	1	2	8	0.25		0.03	2		0.25		0.25	0.12		0.12
1311	*Staphylococcus aureus*	0.5	16		1	2	8	0.12	1	≤0.06	0.5	1	4	8	0.25		0.03	2		0.25		0.25	0.25		0.12
1312	*Staphylococcus aureus*	0.5	8		1	2	1	0.06	0.5	≤0.06	0.25	2	4	16	0.5		0.06	2		0.12		64	0.25		0.06
1313	*Staphylococcus aureus*	0.5	1		0.5	1	0.12	0.12	0.5	≤0.06	0.25	1	2	8	0.25		0.03	2		0.25		0.12	0.06		0.06
1314	*Staphylococcus aureus*	0.5	2		1	1	1	0.12	0.5	≤0.06	2	1	2	8	0.12		0.06	0.25		0.25		0.5	0.5		0.06
1315	*Staphylococcus aureus*	0.5	32		0.25	0.5	16	0.5	1	≤0.06	0.5	1	2	8	0.12		0.03	2		0.25		0.25	0.12		0.06
1316	*Staphylococcus aureus*	0.25	0.06		0.25	1	0.12	0.12	0.5	≤0.06	0.5	1	4	8	0.5		0.12	2		>32		0.5	0.25		0.06

Isolate Number	*Staphylococcus* species	MIC (µg/ml) Values																							
			β Lactams					Carbapenem	Glycopeptides			Cephalosporins			Flouroquinolones			Oxazolidnone	Macrolides			Tetracyclines		Aminoglycoside	Antifolate
		XF-73	Penicillin	Oxacillin	Amoxicillin Clavulanate	Piperacillin/Tazobactam	Ampicillin	Meropenem	Vancomycin	Dalbavancin	Teicoplanin	Cefuroxime	Ceftriaxone	Ceftazidime	Levofloxacin	Ciprofloxacin	Moxifloxacin	Linezolid	Clindamycin	Clarithromycin	Erythromycin	Tetracycline	Tigecycline	Gentamicin	Trimethoprim/ Sulfmethoxazole
1317	*Staphylococcus aureus*	0.5	1		0.25	1	2	0.06	0.5	0.12	0.5	2	4	8	0.25		0.06	2		0.25		0.25	0.12		0.12
1318	*Staphylococcus aureus*	0.5	16		0.25	1	8	0.06	1	≤0.06	0.5	1	2	8	0.12		0.03	2		0.25		0.25	0.12		0.06
1319	*Staphylococcus aureus*	0.5	32		0.5	1	32	0.12	1	0.25	0.25	2	4	16	0.25		0.03	2		>32		1	0.12		0.06
1320	*Staphylococcus aureus*	0.5	16		0.5	1	16	0.06	1	≤0.06	0.5	1	4	16	0.25		0.03	2		0.25		0.25	0.12		0.06
1321	*Staphylococcus aureus*	0.5	8		0.5	1	4	0.06	1	≤0.06	0.5	1	4	8	0.25		0.06	2		0.25		0.5	0.12		0.06
1322	*Staphylococcus aureus*	0.5	16		0.12	0.5	4	0.06	0.5	≤0.06	0.5	1	2	8	0.12		0.015	2		0.25		0.25	0.12		0.06
1323	*Staphylococcus aureus*	0.5	0.03		0.06	0.5	0.12	0.12	1	≤0.06	0.5	1	2	8	0.12		0.03	2		0.25		0.25	0.12		0.06
1324	*Staphylococcus aureus*	0.5	32		0.5	1	16	0.12	1	≤0.06	0.5	1	2	8	0.25		0.06	2		>32		64	0.25		16
1325	*Staphylococcus aureus*	0.5	8		1	2	4	0.25	1	≤0.06	0.5	1	8	16	0.25		0.06	2		0.25		0.25	0.12		0.12
1326	*Staphylococcus aureus*	1	16		1	1	16	0.06	1	≤0.06	0.5	2	4	8	0.12		0.03	4		0.25		0.5	0.25		0.12
1327	*Staphylococcus aureus*	0.5	32		0.5	1	16	0.12	0.5	≤0.06	0.5	1	4	8	0.12		0.015	2		0.12		0.25	0.12		0.06
1328	*Staphylococcus aureus*	0.5	4		0.5	1	4	0.12	0.5	≤0.06	0.5	2	4	16	0.25		0.06	2		0.06		0.25	0.12		0.06
1329	*Staphylococcus aureus*	0.5	32		1	0.5	16	0.12	0.5	0.12	0.25	0.5	2	8	0.25		0.03	2		>32		0.25	0.12		0.12
1330	*Staphylococcus aureus*	0.5	32		1	4	16	0.12	1	0.12	0.5	1	4	8	0.06		0.015	2		0.25		32	0.5		0.12
1331	*Staphylococcus aureus*	1	4		0.5	1	2	0.12	0.5	≤0.06	1	1	2	8	0.12		0.03	2		0.12		0.25	0.12		0.25
1332	*Staphylococcus aureus*	0.5	0.06		0.25	1	0.06	0.12	0.5	≤0.06	0.25	2	4	16	0.12		0.03	2		0.12		0.25	0.12		0.12
1333	*Staphylococcus aureus*	0.5	0.06		0.12	1	0.12	0.06	0.5	≤0.06	0.25	1	4	16	0.12		0.03	2		0.12		0.25	0.25		0.12
1334	*Staphylococcus aureus*	0.5	0.5		0.25	1	0.5	0.06	0.5	≤0.06	0.5	1	4	8	0.25		0.03	2		0.25		0.25	0.12		0.12
1335	*Staphylococcus aureus*	1	8		0.5	2	8	0.06	1	≤0.06	0.5	4	4	8	0.25		0.06	2		0.25		0.25	0.12		0.06
1336	*Staphylococcus aureus*	4	8		0.25	1	8	0.06	1	≤0.06	0.25	2	4	16	0.5		0.06	2		0.25		0.12	0.06		0.06
1337	*Staphylococcus aureus*	0.5	1		0.25	1	2	0.12	0.5	≤0.06	0.25	2	4	16	0.12		0.03	2		0.25		64	0.12		1
1338	*Staphylococcus aureus*	0.5	1		0.25	1	2	0.25	0.5	≤0.06	0.25	2	4	8	0.12		0.03	2		0.25		64	0.12		1
1339	*Staphylococcus aureus*	0.5	4		1	2	4	0.12	1	≤0.06	0.5	2	4	16	1		0.12	2		>32		64	0.12		0.12
1340	*Staphylococcus aureus*	0.25	0.25		0.5	2	0.25	0.5	1	≤0.06	0.25	1	4	16	0.12		0.03	1		>32		0.5	0.06		4
1341	*Staphylococcus aureus*	0.5	64		0.25	0.5	32	0.06	1	≤0.06	0.5	1	2	8	0.12		0.015	2		0.25		0.5	0.25		0.06
1342	*Staphylococcus aureus*	0.5	8		0.5	1	2	0.25	1	≤0.06	0.25	2	4	8	0.25		0.06	4		0.25		0.5	0.25		0.06
1343	*Staphylococcus aureus*	0.5	32		0.5	8	16	0.12	1	≤0.06	0.5	2	4	8	0.12		0.03	2		0.12		0.25	0.06		0.06
1344	*Staphylococcus aureus*	0.25	2		0.25	1	2	0.06	0.5	≤0.06	0.25	1	4	8	0.12		0.03	2		0.25		64	0.12		1
1345	*Staphylococcus aureus*	0.5	8		0.5	0.5	2	0.12	1	≤0.06	0.5	1	2	8	0.25		0.03	2		0.12		0.25	0.12		0.06
1346	*Staphylococcus aureus*	0.5	0.06		0.25	1	0.12	0.12	1	≤0.06	0.5	2	4	16	0.5		0.06	4		0.12		0.12	0.12		0.06
1347	*Staphylococcus aureus*	1	0.5		0.12	0.5	0.25	0.06	1	≤0.06	0.5	1	2	8	0.25		0.03	2		0.25		0.25	0.12		0.06
1348	*Staphylococcus aureus*	0.5	0.12		0.12	0.5	0.06	0.12	1	≤0.06	0.5	2	4	8	0.25		0.06	2		0.25		0.25	0.12		0.06
1349	*Staphylococcus aureus*	0.5	0.25		0.12	0.5	0.12	0.06	0.5	≤0.06	0.5	1	2	4	0.25		0.06	2		0.12		0.25	0.06		0.25
1350	*Staphylococcus aureus*	0.5	2		0.5	1	2	0.12	1	≤0.06	1	1	2	8	8		2	2		0.25		0.25	0.12		0.06
1351	*Staphylococcus aureus*	1	8		0.5	1	4	0.12	1	≤0.06	0.5	2	4	16	0.5		0.06	2		0.25		0.25	0.12		0.06
1352	*Staphylococcus aureus*	0.5	≤0.03		0.12	0.5	0.06	0.06	1	≤0.06	0.5	1	2	8	0.5		0.12	2		0.12		0.12	0.12		0.06
1353	*Staphylococcus aureus*	0.5	8		0.5	1	4	0.06	1	≤0.06	0.5	2	4	8	0.25		0.03	2		0.25		32	0.25		0.06
1354	*Staphylococcus aureus*	0.5	8		0.5	1	4	0.12	1	≤0.06	0.5	1	4	8	0.25		0.03	2		0.12		0.5	0.12		0.06
1355	*Staphylococcus aureus*	0.5	0.12		0.12	0.5	0.12	0.06	0.5	0.12	0.25	1	2	8	1		0.12	2		0.25		16	0.12		0.12
1356	*Staphylococcus aureus*	0.5	1		0.5	1	1	0.12	1	≤0.06	0.5	1	4	16	8		2	2		0.25		0.25	0.06		0.12
1357	*Staphylococcus aureus*	0.5	64		1	1	32	0.25	1	≤0.06	0.5	2	4	8	0.12		0.03	2		0.25		32	0.25		0.06
1358	*Staphylococcus aureus*	0.5	16		1	2	16	0.12	1	≤0.06	0.5	2	4	8	0.12		0.03	4		0.25		0.25	0.12		0.12
1359	*Staphylococcus aureus*	0.25	0.03		0.25	1	0.12	0.12	0.5	0.12	0.25	1	4	8	0.25		0.06	4		0.25		0.5	0.12		0.12
1360	*Staphylococcus aureus*	0.5	16		1	1	8	0.12	0.5	0.12	0.5	2	4	8	0.12		0.03	4		0.25		0.5	0.12		0.12
1361	*Staphylococcus aureus*	0.25	2		1	2	2	0.25	1	≤0.06	0.25	2	4	8	0.25		0.03	2		0.25		0.25	0.12		0.12
1362	*Staphylococcus aureus*	0.5	16		1	1	16	0.12	1	≤0.06	1	1	2	8	0.5		0.12	1		>32		0.12	0.06		0.12
1363	*Staphylococcus aureus*	1	16		1	2	16	0.06	1	0.12	0.5	2	4	8	0.12		0.06	2		0.25		0.25	0.25		0.06

Isolate Number	*Staphylococcus* species	MIC (µg/ml) Values	β Lactams					Carbapenem	Glycopeptides			Cephalosporins			Flouroquinolones			Oxazolidnone	Macrolides			Tetracyclines		Aminoglycoside	Antifolate
		XF-73	Penicillin	Oxacillin	Amoxicillin Clavulanate	Piperacillin/Tazobactam	Ampicillin	Meropenem	Vancomycin	Dalbavancin	Teicoplanin	Cefuroxime	Ceftriaxone	Ceftazidime	Levofloxacin	Ciprofloxacin	Moxifloxacin	Linezolid	Clindamycin	Clarithromycin	Erythromycin	Tetracycline	Tigecycline	Gentamicin	Trimethoprim/ Sulfmethoxazole
1364	*Staphylococcus aureus*	1	4		1	1	2	0.25	1	≤0.06	0.5	2	8	16	0.5		0.06	2		32		0.25	0.12		0.12
1365	*Staphylococcus aureus*	0.25	16		1	1	>32	0.12	1	≤0.06	0.25	2	4	16	0.25		0.03	4		0.25		0.25	0.25		0.12
1366	*Staphylococcus aureus*	0.5	16		1	1	>32	0.12	1	0.12	0.5	2	4	8	0.12		0.03	2		0.25		0.25	0.12		0.12
1367	*Staphylococcus aureus*	0.5	16		1	1	32	0.12	1	≤0.06	0.5	2	4	8	0.12		0.03	2		>32		0.25	0.25		0.12
1368	*Staphylococcus aureus*	0.5	32		1	1	16	0.12	1	≤0.06	0.5	1	4	8	0.12		0.06	2		0.25		0.25	0.12		0.12
1369	*Staphylococcus aureus*	0.25	16		0.5	1	4	0.12	1	0.12	0.5	1	4	8	0.12		0.06	2		>32		64	0.25		0.12
1370	*Staphylococcus aureus*	0.5	0.06		0.12	0.5	0.06	0.06	1	≤0.06	0.5	1	4	8	0.25		0.06	2		0.25		0.25	0.12		0.12
1371	*Staphylococcus aureus*	0.5	64		1	2	8	0.12	1	≤0.06	0.5	2	4	8	0.25		0.06	2		0.25		0.25	0.12		0.25
1372	*Staphylococcus aureus*	1	32		0.5	1	16	0.12	1	0.12	0.5	1	4	8	0.25		0.03	2		0.25		0.25	0.12		0.12
1373	*Staphylococcus aureus*	1	2		1	1	2	0.12	0.5	≤0.06	0.5	2	4	8	0.12		0.03	2		0.25		0.25	0.12		0.06
1374	*Staphylococcus aureus*	0.5	64		2	2	8	0.12	1	≤0.06	0.5	2	4	16	0.25		0.06	2		>32		0.25	0.12		0.06
1375	*Staphylococcus aureus*	0.5	4		1	1	2	0.12	1	≤0.06	0.5	2	4	8	0.25		0.06	4		0.25		0.5	0.12		0.06
1376	*Staphylococcus aureus*	1	1		1	1	2	0.06	1	0.12	0.25	2	4	16	0.12		0.03	2		32		0.25	0.12		0.12
1377	*Staphylococcus aureus*	0.5	32		1	1	16	0.06	0.5	≤0.06	0.5	2	4	8	0.12		0.06	2		0.12		0.25	0.06		0.06
1378	*Staphylococcus aureus*	0.5	32		1	2	32	0.12	1	≤0.06	0.5	2	4	16	0.25		0.06	4		0.25		0.25	0.12		0.06
1379	*Staphylococcus aureus*	0.5	16		1	2	8	0.12	1	≤0.06	0.5	2	4	16	0.5		0.12	2		0.25		0.25	0.25		0.12
1380	*Staphylococcus aureus*	0.5	0.03		0.12	1	0.12	0.06	0.5	≤0.06	0.25	1	2	8	0.25		0.06	2		>32		0.25	0.25		0.06
1381	*Staphylococcus aureus*	0.5	16		1	2	16	0.06	1	≤0.06	0.5	2	4	16	0.25		0.06	2		0.25		0.25	0.25		0.12
1382	*Staphylococcus aureus*	0.5	64		1	2	32	0.12	0.5	≤0.06	0.5	1	2	8	0.12		0.03	2		0.12		0.25	0.25		0.12
1383	*Staphylococcus aureus*	0.5	0.12		0.5	1	0.25	0.12	1	0.12	0.5	1	4	8	0.25		0.03	2		0.25		0.25	0.25		0.12
1384	*Staphylococcus aureus*	0.5	16		1	2	16	0.12	1	0.12	0.5	2	4	16	0.5		0.12	2		0.25		0.5	0.25		0.25
1385	*Staphylococcus aureus*	0.5	0.06		0.25	1	0.12	0.12	0.5	0.12	0.25	1	4	16	0.25		0.06	2		0.25		0.25	0.25		0.12
1386	*Staphylococcus aureus*	0.5	1		1	1	1	0.12	0.5	0.12	0.5	1	4	16	0.12		0.03	4		0.25		0.25	0.25		0.06
1387	*Staphylococcus aureus*	0.5	64		0.5	1	8	0.12	1	0.12	1	1	4	8	0.12		0.03	4		0.25		0.5	0.25		0.12
1388	*Staphylococcus aureus*	0.5	0.06		0.25	1	0.12	0.12	1	≤0.06	0.5	1	4	8	0.25		0.03	8		0.25		0.25	0.12		0.12
1389	*Staphylococcus aureus*	0.5	0.06		0.25	1	0.12	0.12	0.5	≤0.06	0.25	1	2	8	0.25		0.06	1		0.12		0.25	0.25		0.12
1390	*Staphylococcus aureus*	0.5	16		0.5	1	8	0.12	1	0.12	1	1	2	8	0.12		0.03	2		0.12		0.12	0.06		0.06
1391	*Staphylococcus aureus*	1	1		1	1	2	0.12	0.5	≤0.06	0.25	1	2	8	0.12		0.03	2		0.12		0.25	0.25		0.06
1392	*Staphylococcus aureus*	0.5	0.06		0.12	0.5	0.06	0.06	0.5	≤0.06	0.5	1	4	8	0.25		0.06	2		0.25		0.12	0.12		0.12
1393	*Staphylococcus aureus*	0.5	8		1	2	4	0.12	0.5	≤0.06	0.5	2	4	8	0.25		0.06	2		0.25		0.25	0.25		0.06
1394	*Staphylococcus aureus*	0.5	32		1	2	16	0.12	1	0.12	0.5	2	4	16	0.5		0.12	2		0.25		0.25	0.25		0.03
1395	*Staphylococcus aureus*	0.5	16		0.5	1	16	0.12	0.5	0.06	0.5	1	4	8	0.25		0.03	1		0.12		0.12	0.06		0.12
1396	*Staphylococcus aureus*	0.5	0.03		0.06	0.5	0.12	0.06	0.5	≤0.06	0.5	0.5	4	8	0.12		0.03	2		0.12		0.25	0.25		0.06
1397	*Staphylococcus aureus*	0.5	0.5		0.25	1	0.25	0.12	0.5	≤0.06	0.5	1	2	8	0.12		0.015	1		0.12		0.12	0.06		0.06
1398	*Staphylococcus aureus*	0.5	0.5		0.25	1	0.5	0.06	0.5	0.12	0.25	2	4	8	0.5		0.06	2		0.12		0.25	0.25		0.12
1399	*Staphylococcus aureus*	1	0.03		0.25	1	0.12	0.12	0.5	0.12	0.5	1	2	8	0.12		0.03	2		0.25		0.25	0.12		0.06
1400	*Staphylococcus aureus*	1	0.06		0.25	1	0.12	0.12	0.5	≤0.06	0.5	1	2	8	0.12		0.03	2		0.25		32	0.5		0.06
1401	*Staphylococcus aureus*	0.5	32		1	2	32	0.12	1	0.12	0.5	1	4	8	0.25		0.12	4		0.25		0.25	0.12		0.06
1402	*Staphylococcus aureus*	0.5	16		1	1	16	0.06	1	0.25	0.5	1	2	8	0.12		0.03	2		0.25		0.25	0.12		0.06
1403	*Staphylococcus aureus*	0.5	8		1	1	4	0.25	1	>0.5	0.5	2	4	16	0.25		0.03	2		0.25		0.25	0.12		0.06
1404	*Staphylococcus aureus*	1	0.25		0.25	1	0.5	0.12	1	0.12	1	1	4	16	1		0.12	2		>32		0.25	0.12		0.06
1405	*Staphylococcus aureus*	0.5	8		1	1	8	0.06	1	≤0.06	0.5	2	4	8	0.25		0.06	2		0.25		0.25	0.06		2
1406	*Staphylococcus aureus*	0.5	2		1	1	2	0.06	0.5	≤0.06	0.5	2	4	8	0.25		0.06	2		0.25		0.25	0.12		0.12
1407	*Staphylococcus aureus*	0.5	2		1	1	2	0.25	1	≤0.06	0.5	2	4	16	0.25		0.06	2		0.25		0.25	0.12		0.06
1408	*Staphylococcus aureus*	0.5	1		0.5	1	1	0.06	1	0.25	0.5	2	4	8	0.25		0.03	2		0.12		0.25	0.12		0.06
1409	*Staphylococcus aureus*	0.5	0.06		0.12	0.5	0.06	0.12	1	0.25	0.25	1	2	8	0.12		0.03	2		>32		1	0.25		0.06
1410	*Staphylococcus aureus*	0.5	8		0.5	1	4	0.12	1	≤0.06	0.25	1	2	8	0.12		0.06	2		0.12		0.25	0.12		0.03

		MIC (µg/ml) Values																							
			β Lactams					Carbapenem	Glycopeptides			Cephalosporins			Flouroquinolones			Oxazolidnone	Macrolides			Tetracyclines		Aminoglycoside	Antifolate
Isolate Number	*Staphylococcus* species	XF-73	Penicillin	Oxacillin	Amoxicillin Clavulanate	Piperacillin/Tazobactam	Ampicillin	Meropenem	Vancomycin	Dalbavancin	Teicoplanin	Cefuroxime	Ceftriaxone	Ceftazidime	Levofloxacin	Ciprofloxacin	Moxifloxacin	Linezolid	Clindamycin	Clarithromycin	Erythromycin	Tetracycline	Tigecycline	Gentamicin	Trimethoprim/ Sulfmethoxazole
1411	*Staphylococcus aureus*	0.5	2		0.5	1	2	0.06	0.5	0.25	0.25	2	4	8	0.25		0.06	2		0.12		0.25	0.12		0.12
1412	*Staphylococcus aureus*	0.25	1		1	32	2	0.12	0.25	≤0.06	0.25	2	4	32	4		2	0.25		0.06		0.12	0.03		16
1413	*Staphylococcus aureus*	1	0.25		0.25	1	0.25	0.12	0.5	0.12	0.5	1	4	8	0.25		0.06	2		0.25		0.25	0.06		0.12
1414	*Staphylococcus aureus*	0.5	>64		1	1	>32	0.12	1	≤0.06	0.5	2	4	8	0.25		0.06	4		1		64	0.25		0.25
1415	*Staphylococcus aureus*	0.5	1		0.5	1	1	0.12	0.5	≤0.06	0.25	2	4	8	0.25		0.06	2		0.25		0.5	0.06		0.06
1416	*Staphylococcus aureus*	0.5	≤0.03		0.25	1	0.12	0.12	1	0.12	0.5	2	4	8	0.5		0.06	2		0.25		0.25	0.12		0.06
1417	*Staphylococcus aureus*	0.5	32		1	2	32	0.25	1	0.25	0.5	2	4	16	0.25		0.06	2		0.25		0.5	0.12		0.12
1418	*Staphylococcus aureus*	0.5	≤0.03		0.25	1	0.12	0.12	0.5	≤0.06	0.5	2	4	16	0.12		0.03	2		0.25		0.25	0.12		0.25
1419	*Staphylococcus aureus*	0.5	32		1	1	32	0.12	1	≤0.06	0.5	2	4	8	0.25		0.06	4		0.5		0.5	0.25		0.12
1420	*Staphylococcus aureus*	1	32		0.5	1	16	0.12	0.5	≤0.06	0.25	2	4	8	0.25		0.06	2		0.12		0.25	0.12		0.06
1421	*Staphylococcus aureus*	0.5	1		0.25	1	0.5	0.12	0.5	0.25	0.5	2	4	8	0.5		0.03	2		>32		0.25	0.12		0.06
1422	*Staphylococcus aureus*	0.5	2		0.5	1	2	0.12	0.5	0.12	0.25	2	4	8	0.12		0.03	4		0.25		0.5	0.12		0.06
1423	*Staphylococcus aureus*	0.5	0.06		0.12	0.25	0.12	0.12	0.5	0.12	0.5	2	2	8	0.12		0.03	4		0.25		0.5	0.25		0.06
1424	*Staphylococcus aureus*	0.5	≤0.03		0.12	0.5	0.12	0.06	1	0.25	0.5	1	4	8	0.5		0.12	1		>32		0.25	0.25		0.06
1425	*Staphylococcus aureus*	0.5	8		0.25	0.5	2	0.06	0.5	0.12	0.12	1	2	4	0.06		0.008	1		0.12		0.12	0.12		0.06
1426	*Staphylococcus aureus*	0.5	≤0.03		0.12	0.5	0.06	0.06	1	0.12	0.5	1	2	8	0.25		0.06	2		>32		0.25	0.12		0.03
1427	*Staphylococcus aureus*	0.5	0.5		0.5	0.5	0.5	0.06	1	0.12	0.5	1	2	8	0.25		0.03	1		0.12		0.25	0.12		0.06
1428	*Staphylococcus aureus*	0.5	≤0.03		0.12	0.5	0.12	0.06	1	0.12	0.5	0.5	2	8	>8		8	2		>32		0.25	0.12		0.03
1429	*Staphylococcus aureus*	0.5	8		0.25	0.5	4	0.12	0.5	0.12	0.5	1	2	8	0.5		0.06	1		0.12		0.12	0.12		0.06
1430	*Staphylococcus aureus*	0.5	≤0.03		0.25	0.5	0.06	0.06	1	0.12	0.5	2	4	8	>8		8	2		>32		0.25	0.12		0.06
1431	*Staphylococcus aureus*	0.5	0.5		0.5	1	0.5	0.06	0.5	≤0.06	0.5	1	2	16	0.25		0.06	1		0.12		0.12	0.5		0.06
1432	*Staphylococcus aureus*	0.5	0.06		0.12	0.5	0.12	0.06	0.5	0.12	0.25	1	2	8	0.12		0.03	1		>32		0.12	0.12		0.06
1433	*Staphylococcus aureus*	0.5	4		1	1	4	0.25	1	0.12	0.5	2	4	16	0.25		0.06	2		0.25		0.25	2		0.06
1434	*Staphylococcus aureus*	1	0.12		0.25	1	0.06	0.12	1	0.12	0.5	2	4	16	0.12		0.03	2		0.25		0.25	1		0.12
1435	*Staphylococcus aureus*	0.5	2		0.5	1	1	0.12	2	0.25	0.5	2	4	16	0.5		0.12	2		4		0.12	0.5		>32
1436	*Staphylococcus aureus*	0.5	8		0.12	0.5	0.5	0.06	1	0.12	0.25	1	2	4	0.12		0.015	2		0.25		0.25	0.25		0.12
1437	*Staphylococcus aureus*	0.5	8		0.5	1	8	0.12	1	0.12	0.5	1	4	8	0.25		0.03	2		0.12		0.25	0.25		0.06
1438	*Staphylococcus aureus*	0.5	0.06		0.25	1	0.12	0.12	1	0.12	0.5	2	4	8	0.12		0.03	2		8		0.25	1		0.06
1439	*Staphylococcus aureus*	0.5	1		0.5	1	1	0.12	1	0.25	0.5	1	4	8	0.12		0.03	2		0.25		0.25	0.25		0.06
1440	*Staphylococcus aureus*	0.5	0.5		0.5	1	1	0.12	1	0.12	0.5	1	4	8	8		2	2		>32		0.25	0.25		1
1441	*Staphylococcus aureus*	0.5	0.5		0.5	1	0.25	0.12	1	0.12	1	2	4	16	0.25		0.12	2		0.25		0.25	1		0.12
1442	*Staphylococcus aureus*	0.5	≤0.03		0.12	0.5	0.12	0.06	1	0.12	0.5	2	4	16	0.25		0.06	2		0.25		0.25	0.25		0.06
1443	*Staphylococcus aureus*	0.5	32		1	2	16	0.25	1	0.12	0.5	2	4	16	0.25		0.06	2		0.25		0.25	0.25		0.06
1444	*Staphylococcus aureus*	0.5	0.06		0.25	1	0.12	0.12	1	≤0.06	0.5	1	4	16	0.25		0.03	2		0.12		0.25	0.5		0.06
1445	*Staphylococcus aureus*	0.5	4		1	4	2	0.12	0.5	≤0.06	0.25	2	4	8	0.25		0.06	2		32		0.25	0.12		0.12
1446	*Staphylococcus aureus*	0.5	32		0.5	1	16	0.12	1	≤0.06	0.5	1	4	8	0.12		0.015	2		>32		0.25	0.12		0.12
1447	*Staphylococcus aureus*	1	64		1	1	32	0.12	0.5	≤0.06	1	1	2	16	8		4	2		0.25		0.5	0.12		0.12
1448	*Staphylococcus aureus*	0.5	>64		1	1	>32	0.06	1	≤0.06	0.5	1	2	4	0.12		0.03	2		>32		0.25	0.12		0.06
1449	*Staphylococcus aureus*	0.5	0.06		0.25	0.5	0.06	0.12	1	0.25	1	1	2	8	0.12		0.03	2		0.25		0.12	0.12		0.06
1450	*Staphylococcus aureus*	0.5	32		1	1	16	0.12	0.5	≤0.06	0.5	1	2	8	8		2	2		0.25		0.25	0.12		0.5
1451	*Staphylococcus aureus*	0.5	0.06		0.25	1	0.06	0.12	1	0.12	0.5	2	8	8	0.12		0.015	2		0.25		0.25	0.12		0.06
1452	*Staphylococcus aureus*	0.5	4		0.5	1	2	0.06	0.5	0.12	0.25	1	2	8	0.25		0.03	2		0.25		0.25	0.12		0.12
1453	*Staphylococcus aureus*	0.5	32		2	2	32	0.12	1	0.12	0.5	2	4	8	0.25		0.06	2		0.25		0.25	0.12		0.12
1454	*Staphylococcus aureus*	0.25	>64		2	1	>32	0.25	1	0.12	0.5	2	4	16	0.12		0.03	2		0.25		0.25	0.12		0.12
1455	*Staphylococcus aureus*	0.5	2		2	4	2	0.25	1	0.25	1	4	8	32	0.25		0.03	4		>32		8	0.25		0.5
1456	*Staphylococcus aureus*	0.5	≤0.03		0.12	1	0.06	0.06	0.5	0.12	0.5	1	2	8	0.25		0.06	2		0.25		0.25	0.12		0.12
1457	*Staphylococcus aureus*	1	16		1	2	8	0.12	0.5	0.12	0.5	2	4	16	0.12		0.03	2		0.25		0.25	0.06		0.06

Isolate Number	*Staphylococcus* species	MIC (µg/ml) Values																							
			β Lactams					Carbapenem	Glycopeptides			Cephalosporins			Flouroquinolones			Oxazolidnone	Macrolides			Tetracyclines		Aminoglycoside	Antifolate
		XF-73	Penicillin	Oxacillin	Amoxicillin Clavulanate	Piperacillin/Tazobactam	Ampicillin	Meropenem	Vancomycin	Dalbavancin	Teicoplanin	Cefuroxime	Ceftriaxone	Ceftazidime	Levofloxacin	Ciprofloxacin	Moxifloxacin	Linezolid	Clindamycin	Clarithromycin	Erythromycin	Tetracycline	Tigecycline	Gentamicin	Trimethoprim/ Sulfmethoxazole
1458	*Staphylococcus aureus*	0.5	≤0.03		0.12	1	0.06	0.06	0.5	≤0.06	0.25	1	4	8	0.25		0.06	2		32		0.25	0.12		0.5
1459	*Staphylococcus aureus*	1	0.06		0.5	1	0.25	0.12	1	0.12	2	2	2	1	0.25		0.06	2		0.25		0.25	0.12		0.12
1460	*Staphylococcus aureus*	1	≤0.03		0.12	1	0.12	0.06	0.5	≤0.06	1	1	2	1	0.25		0.06	2		0.25		0.25	0.12		0.5
1461	*Staphylococcus aureus*	1	1		0.25	1	0.5	0.12	1	0.12	0.5	1	2	2	0.25		0.03	2		0.25		0.25	0.25		0.12
1462	*Staphylococcus aureus*	0.5	16		1	1	8	0.12	1	≤0.06	0.5	2	2	1	0.5		0.03	2		32		0.25	0.12		0.06
1463	*Staphylococcus aureus*	0.5	≤0.03		0.12	1	0.06	0.12	0.5	≤0.06	0.5	2	2	1	0.12		0.06	2		0.25		0.25	0.12		0.25
1464	*Staphylococcus aureus*	0.5	16		0.5	0.5	4	0.12	1	0.12	0.25	1	2	1	0.25		0.06	2		0.25		0.25	0.12		0.12
1465	*Staphylococcus aureus*	1	2		0.25	1	4	0.12	1	≤0.06	0.5	1	2	2	0.12		0.03	2		>32		64	0.25		0.12
1466	*Staphylococcus aureus*	0.5	2		0.5	1	1	0.12	1	≤0.06	0.5	1	2	8	0.12		0.03	2		0.25		0.25	0.12		0.06
1467	*Staphylococcus aureus*	0.5	8		1	2	4	0.12	1	≤0.06	0.5	2	4	8	0.25		0.06	2		0.25		0.25	0.12		0.12
1468	*Staphylococcus aureus*	0.5	32		0.5	1	16	0.12	1	0.12	0.5	1	2	8	0.12		0.015	8		0.25		0.25	0.12		0.12
1469	*Staphylococcus aureus*	0.5	64		0.5	1	32	0.12	2	≤0.06	0.5	2	2	8	0.25		0.015	2		0.25		0.25	0.12		0.06
1470	*Staphylococcus aureus*	0.5	0.06		0.25	1	0.06	0.12	1	0.12	0.5	2	4	8	0.25		0.06	2		0.25		0.25	0.25		0.06
1471	*Staphylococcus aureus*	0.5	0.06		0.25	0.5	0.12	0.12	1	0.12	0.5	1	4	8	0.25		0.06	2		0.25		0.25	0.12		0.06
1472	*Staphylococcus aureus*	0.5	32		1	1	32	0.06	1	0.25	0.5	1	4	8	0.25		0.03	2		0.25		0.25	0.5		0.06
1473	*Staphylococcus aureus*	0.5	16		0.5	1	8	0.12	2	0.12	0.5	1	4	8	0.12		0.03	2		0.12		0.25	0.25		0.06
1474	*Staphylococcus aureus*	0.5	4		1	1	2	0.12	1	0.5	0.5	2	4	8	0.25		0.03	2		0.25		0.25	0.25		0.06
1475	*Staphylococcus aureus*	0.5	1		0.5	1	2	0.12	1	0.12	0.5	2	4	16	0.12		0.015	2		0.25		0.5	0.5		0.06
1476	*Staphylococcus aureus*	0.5	0.06		0.25	1	0.12	0.06	1	≤0.06	0.5	1	4	8	0.25		0.03	2		0.25		0.12	0.06		0.06
1477	*Staphylococcus aureus*	0.5	4		1	1	2	0.12	1	≤0.06	0.5	2	4	8	0.25		0.06	2		0.25		0.25	0.25		0.12
1478	*Staphylococcus aureus*	0.5	16		1	1	8	0.12	1	≤0.06	0.5	2	4	16	0.12		0.03	2		0.25		0.25	0.25		0.06
1479	*Staphylococcus aureus*	0.5	0.06		0.25	1	0.12	0.12	1	≤0.06	1	2	4	16	0.12		0.03	2		0.25		0.25	0.25		0.12
1480	*Staphylococcus aureus*	0.5	0.5		0.5	1	1	0.12	1	0.12	0.5	2	4	16	0.25		0.06	2		>32		0.25	0.25		0.06
1481	*Staphylococcus aureus*	0.5	32		1	2	8	0.12	1	0.06	1	2	4	16	0.5		0.12	2		0.25		0.5	1		0.06
1482	*Staphylococcus aureus*	0.5	4		0.5	1	2	0.25	1	0.12	1	1	4	16	8		2	16		2		1	4		8
1483	*Staphylococcus aureus*	0.5	0.06		0.25	1	0.06	0.12	1	≤0.06	0.5	2	4	16	1		0.5	4		0.25		8	1		8
1484	*Staphylococcus aureus*	0.5	8		1	1	8	0.25	1	≤0.06	0.5	2	4	16	0.25		2	4		>32		8	1		8
1485	*Staphylococcus aureus*	0.5	64		1	16	>32	0.12	1	0.25	0.5	2	4	8	0.25		0.06	16		0.25		16	>4		0.12
1486	*Staphylococcus aureus*	1	>64		2	2	>32	0.25	2	0.12	2	2	8	16	2		2	8		16		1	1		0.25
1487	*Staphylococcus aureus*	1	64		1	2	>32	0.25	1	0.25	0.5	2	4	16	>8		1	8		>32		0.5	0.5		4
1488	*Staphylococcus aureus*	0.5	8		0.5	1	4	0.12	1	0.12	0.5	1	4	16	0.25		0.06	2		>32		0.5	0.25		0.12
1489	*Staphylococcus aureus*	0.5	≤0.03		0.25	1	0.12	0.12	1	≤0.06	0.5	2	4	8	0.25		0.06	4		0.25		0.5	0.5		0.12
1490	*Staphylococcus aureus*	0.5	32		0.5	1	8	0.12	1	0.25	0.5	2	4	16	0.25		0.03	4		0.25		0.5	0.25		0.12
1491	*Staphylococcus aureus*	0.5	16		0.5	1	8	0.12	1	0.12	0.5	1	4	16	0.5		0.5	4		0.25		0.5	0.25		0.06
1492	*Staphylococcus aureus*	0.5	8		1	2	4	0.12	1	0.12	1	2	4	16	0.12		0.25	2		0.25		0.25	0.25		0.12
1493	*Staphylococcus aureus*	0.25	0.25		0.25	1	0.5	0.12	1	0.12	0.25	1	4	8	0.25		0.03	2		0.25		0.25	0.12		0.06
1494	*Staphylococcus aureus*	0.5	1		0.5	1	1	0.12	1	0.25	0.5	1	4	8	0.25		0.06	2		0.25		0.25	0.12		0.06
1495	*Staphylococcus aureus*	0.5	≤0.03		0.12	1	0.12	0.06	1	≤0.06	1	1	4	8	1		0.06	2		0.25		0.25	0.12		0.06
1496	*Staphylococcus aureus*	1	0.25		0.25	1	0.25	0.06	1	0.25	0.5	1	4	8	0.25		0.03	2		0.25		0.12	0.06		0.12
1497	*Staphylococcus aureus*	0.5	4		1	1	2	0.25	0.5	0.12	0.25	2	4	8	0.12		0.03	2		0.25		0.25	0.06		0.06
1498	*Staphylococcus aureus*	1	1		0.5	1	1	0.12	1	0.5	0.5	2	4	8	0.25		0.03	2		0.25		0.25	0.12		0.06
1499	*Staphylococcus aureus*	0.5	1		0.5	1	1	0.12	1	0.12	0.5	1	4	8	0.25		0.03	2		0.12		0.25	0.12		0.06
1500	*Staphylococcus aureus*	0.5	32		1	1	32	0.25	1	0.25	0.5	1	4	8	0.25		0.12	2		0.25		0.25	0.12		0.12
1501	*Staphylococcus aureus*	0.5	0.12		0.25	1	0.12	0.12	1	0.25	0.5	2	4	16	0.12		0.06	2		0.25		0.25	0.12		0.06
1502	*Staphylococcus aureus*	0.5	32		1	2	16	0.12	1	0.12	0.5	2	4	8	0.25		0.03	2		0.25		0.25	0.12		0.12
1503	*Staphylococcus aureus*	0.5	64		1	2	>32	0.12	1	0.25	0.5	2	4	8	0.25		0.06	2		0.25		0.25	0.12		0.06
1504	*Staphylococcus aureus*	0.5	8		1	1	8	0.06	1	0.12	0.5	2	4	8	0.12		0.03	2		0.25		0.25	0.12		0.06

		MIC (µg/ml) Values																							
			β Lactams					Carbapenem	Glycopeptides			Cephalosporins			Flouroquinolones			Oxazolidnone	Macrolides			Tetracyclines		Aminoglycoside	Antifolate
Isolate Number	***Staphylococcus* species**	**XF-73**	**Penicillin**	**Oxacillin**	**Amoxicillin Clavulanate**	**Piperacillin/Tazobactam**	**Ampicillin**	**Meropenem**	**Vancomycin**	**Dalbavancin**	**Teicoplanin**	**Cefuroxime**	**Ceftriaxone**	**Ceftazidime**	**Levofloxacin**	**Ciprofloxacin**	**Moxifloxacin**	**Linezolid**	**Clindamycin**	**Clarithromycin**	**Erythromycin**	**Tetracycline**	**Tigecycline**	**Gentamicin**	**Trimethoprim/ Sulfmethoxazole**
1505	*Staphylococcus aureus*	0.5	≤0.03		0.25	1	0.06	0.06	1	≤0.06	1	1	4	8	0.25		0.06	2		0.25		2	1		0.25
1506	*Staphylococcus aureus*	0.5	1		0.5	1	1	0.12	1	0.25	0.5	2	4	8	0.25		0.06	2		0.12		0.5	0.12		0.12
1507	*Staphylococcus aureus*	0.5	16		1	1	8	0.06	1	≤0.06	0.5	1	4	4	0.25		0.06	2		0.25		0.25	0.25		0.06
1508	*Staphylococcus aureus*	0.5	8		1	1	4	0.12	0.5	0.5	0.5	2	4	8	0.25		0.06	2		0.12		0.25	0.12		0.06
1509	*Staphylococcus aureus*	1	16		0.5	0.5	4	0.06	1	0.5	0.25	1	2	8	0.5		0.015	2		>32		0.25	0.12		0.12
1510	*Staphylococcus aureus*	0.5	8		0.5	1	2	0.12	0.5	0.25	0.5	2	4	8	0.5		0.06	2		0.25		0.25	0.12		0.12
1511	*Staphylococcus aureus*	0.5	32		0.5	1	16	0.12	0.5	0.12	0.5	1	4	8	0.25		0.06	2		>32		0.25	0.25		0.12
1512	*Staphylococcus aureus*	1	4		0.5	1	2	0.12	1	0.12	0.5	2	4	8	0.25		0.06	2		0.25		16	0.25		0.12
1513	*Staphylococcus aureus*	0.5	4		1	1	2	0.12	1	0.5	0.5	2	4	8	0.25		0.03	2		0.25		0.25	0.12		0.12
1514	*Staphylococcus aureus*	0.5	≤0.03		0.12	0.5	0.06	0.12	1	0.25	1	2	2	8	0.25		0.06	4		0.25		0.25	0.5		0.12
1515	*Staphylococcus aureus*	0.5	64		1	2	>32	0.12	1	0.12	0.5	2	4	8	0.25		0.06	4		0.25		0.5	0.25		0.12
1516	*Staphylococcus aureus*	1	16		1	1	8	0.12	1	0.12	0.5	2	4	8	0.25		0.06	2		0.12		0.25	0.25		0.06
1517	*Staphylococcus aureus*	1	2		1	1	2	0.12	1	≤0.06	0.5	2	4	16	0.25		0.06	2		0.25		0.25	0.25		0.06
1518	*Staphylococcus aureus*	1	≤0.03		0.25	1	0.12	0.12	1	≤0.06	1	2	4	16	0.25		0.06	2		0.25		0.25	0.12		0.06
1519	*Staphylococcus aureus*	1	32		1	2	32	0.12	1	0.25	0.5	2	4	8	0.25		0.03	2		0.25		0.25	0.12		0.06
1520	*Staphylococcus aureus*	0.5	32		1	2	32	0.12	1	0.12	0.5	2	4	16	0.25		0.06	2		0.25		0.25	0.12		0.06
1521	*Staphylococcus aureus*	0.5	16		1	2	8	0.12	1	≤0.06	0.5	2	4	8	0.5		0.06	2		0.25		0.25	1		0.06
1522	*Staphylococcus aureus*	0.5	≤0.03		0.25	0.5	0.12	0.12	1	0.25	1	2	4	8	0.12		0.03	1		0.12		0.25	2		0.06
1523	*Staphylococcus aureus*	0.5	32		1	2	32	0.06	1	0.25	0.5	2	4	8	0.5		0.06	2		0.25		0.25	1		0.12
1524	*Staphylococcus aureus*	1	0.5		0.25	1	0.5	0.12	1	0.12	1	1	4	8	0.25		0.06	2		0.25		0.25	1		0.12
1525	*Staphylococcus aureus*	0.5	4		1	1	2	0.25	1	0.5	0.5	2	4	16	0.12		0.03	2		0.25		0.25	1		0.06
1526	*Staphylococcus aureus*	0.5	32		1	2	32	0.12	1	≤0.06	0.5	2	4	8	0.5		0.06	2		0.25		0.25	0.5		0.12
1527	*Staphylococcus aureus*	0.5	8		1	1	4	0.12	1	≤0.06	1	2	4	8	0.12		0.03	2		0.25		64	1		0.12
1528	*Staphylococcus aureus*	0.5	32		1	2	32	0.12	1	0.12	0.5	1	4	16	0.25		0.06	4		0.25		0.5	0.25		0.25
1529	*Staphylococcus aureus*	0.25	4		0.5	1	2	0.06	1	≤0.06	0.5	1	4	8	0.25		0.06	4		32		1	0.25		0.12
1530	*Staphylococcus aureus*	0.5	2		0.5	1	2	0.06	0.5	0.5	0.25	1	4	8	0.25		0.06	2		0.12		0.25	0.12		0.12
1531	*Staphylococcus aureus*	0.5	32		1	2	16	0.12	1	0.25	0.5	2	4	16	0.25		0.03	2		0.25		0.5	0.25		0.12
1532	*Staphylococcus aureus*	0.25	64		1	2	32	0.12	0.5	≤0.06	0.5	2	4	16	0.25		0.12	2		0.25		0.5	0.5		0.12
1533	*Staphylococcus aureus*	0.5	32		1	2	32	0.12	1	≤0.06	0.25	2	4	16	0.25		0.06	2		0.25		0.25	0.25		0.12
1534	*Staphylococcus aureus*	0.5	64		0.5	0.06	>32	0.12	0.5	0.12	0.5	1	4	16	0.25		0.06	2		0.25		0.5	0.12		0.25
1535	*Staphylococcus aureus*	0.25	16		1	0.12	16	0.12	0.5	0.25	0.25	2	4	8	0.25		0.03	2		0.25		0.25	0.12		0.12
1536	*Staphylococcus aureus*	4	4		1	0.25	4	0.25	1	0.5	1	2	4	8	0.25		0.06	2		0.12		0.25	0.12		0.12
1537	*Staphylococcus aureus*	0.5	1		1	≤0.03	1	0.12	0.5	≤0.06	0.5	2	4	16	0.12		0.12	2		0.12		0.25	0.12		0.12
1538	*Staphylococcus aureus*	0.5	16		1	2	8	0.12	0.5	≤0.06	0.5	2	4	16	0.25		0.03	2		0.25		0.25	0.25		0.06
1539	*Staphylococcus aureus*	2	2		1	1	2	0.12	1	≤0.06	0.5	2	4	8	0.25		0.03	2		0.25		0.25	0.12		0.12
1540	*Staphylococcus aureus*	0.5	2		0.5	1	2	0.12	0.5	0.12	0.25	1	2	8	0.12		0.03	2		0.12		0.12	0.06		0.06
1541	*Staphylococcus aureus*	1	≤0.03		0.25	0.5	0.12	0.12	1	0.12	0.5	1	4	8	0.25		0.06	2		0.25		0.25	0.12		0.06
1542	*Staphylococcus aureus*	0.25	1		0.5	1	1	0.12	0.5	≤0.06	0.5	1	2	8	0.12		0.03	2		0.12		0.12	0.06		0.06
1543	*Staphylococcus aureus*	1	16		1	2	8	0.12	0.5	≤0.06	0.25	2	4	8	0.25		0.06	2		0.25		0.25	0.12		0.12
1544	*Staphylococcus aureus*	0.5	2		0.5	0.5	2	0.12	1	0.12	1	1	4	8	0.25		0.03	1		0.12		0.25	0.12		0.06
1545	*Staphylococcus aureus*	0.5	2		0.5	1	2	0.12	1	0.12	0.5	2	2	8	0.25		0.03	2		0.12		0.25	0.12		0.06
1546	*Staphylococcus aureus*	0.5	1		0.5	0.5	1	0.12	0.5	0.12	0.25	1	2	8	0.12		0.03	2		0.12		0.12	0.06		0.06
1547	*Staphylococcus aureus*	0.5	2		0.5	1	2	0.12	0.5	≤0.06	0.5	1	2	8	0.12		0.03	2		0.12		0.25	0.06		0.06
1548	*Staphylococcus aureus*	0.5	16		1	1	16	0.06	1	0.5	0.5	2	4	8	0.25		0.03	2		32		0.25	0.12		0.12
1549	*Staphylococcus aureus*	0.5	2		0.5	1	2	0.12	1	0.12	0.5	1	4	8	0.12		0.03	2		0.25		0.25	0.12		0.06
1550	*Staphylococcus aureus*	0.5	32		2	2	32	0.12	1	0.12	0.5	2	4	8	0.12		0.03	2		0.25		0.25	0.12		0.06
1551	*Staphylococcus aureus*	0.25	16		1	2	8	0.25	1	0.12	0.5	2	4	16	0.25		0.03	2		0.25		0.25	0.12		0.12

		MIC (µg/ml) Values																							
			β Lactams					Carbapenem	Glycopeptides			Cephalosporins			Flouroquinolones			Oxazolidnone	Macrolides			Tetracyclines		Aminoglycoside	Antifolate
**Isolate Number**	***Staphylococcus* species**	**XF-73**	**Penicillin**	**Oxacillin**	**Amoxicillin Clavulanate**	**Piperacillin/Tazobactam**	**Ampicillin**	**Meropenem**	**Vancomycin**	**Dalbavancin**	**Teicoplanin**	**Cefuroxime**	**Ceftriaxone**	**Ceftazidime**	**Levofloxacin**	**Ciprofloxacin**	**Moxifloxacin**	**Linezolid**	**Clindamycin**	**Clarithromycin**	**Erythromycin**	**Tetracycline**	**Tigecycline**	**Gentamicin**	**Trimethoprim/ Sulfmethoxazole**
1552	*Staphylococcus aureus*	0.5	2		1	1	2	0.12	0.5	0.12	0.5	1	4	8	0.25		0.03	4		0.25		0.5	0.12		0.12
1553	*Staphylococcus aureus*	0.5	16		1	2	16	0.12	1	0.12	0.5	2	4	8	0.25		0.03	4		0.25		0.5	0.5		0.12
1554	*Staphylococcus aureus*	0.25	0.5		0.5	1	0.5	0.12	1	0.12	0.5	2	4	16	0.06		0.015	2		>32		0.25	0.12		0.12
1555	*Staphylococcus aureus*	0.5	32		1	1	32	0.12	1	>0.5	0.5	1	2	8	0.06		0.015	2		0.25		0.25	0.12		0.06
1556	*Staphylococcus aureus*	0.5	0.06		0.12	1	0.12	0.12	1	0.25	0.5	1	2	4	0.25		0.03	2		4		0.12	0.06		0.06
1557	*Staphylococcus aureus*	0.5	16		1	2	16	0.12	1	0.12	0.25	2	4	8	0.25		0.06	2		0.5		0.25	0.12		0.12
1558	*Staphylococcus aureus*	0.5	2		0.5	1	2	0.12	1	0.12	0.5	1	4	8	0.12		0.03	2		>32		0.25	0.12		0.12
1559	*Staphylococcus aureus*	0.5	0.5		0.5	1	0.5	0.12	1	0.25	0.5	1	4	16	>8		4	2		0.25		0.25	0.12		0.12
1560	*Staphylococcus aureus*	0.5	1		0.5	1	1	0.12	1	0.12	0.5	1	4	8	0.12		0.03	2		0.25		0.25	0.25		0.06
1561	*Staphylococcus aureus*	0.5	0.06		0.25	1	0.12	0.12	0.5	0.25	0.5	2	4	8	0.12		0.015	2		>32		0.25	0.25		0.12
1562	*Staphylococcus aureus*	0.25	16		1	2	16	0.12	2	0.12	0.5	2	4	8	0.25		0.06	2		0.25		0.25	0.25		0.12
1563	*Staphylococcus aureus*	0.5	8		1	1	8	0.06	1	0.12	0.5	2	4	16	0.25		0.06	2		0.25		0.12	0.12		0.06
1564	*Staphylococcus aureus*	0.5	0.12		0.12	0.5	0.12	0.12	1	0.12	0.5	2	4	8	0.12		0.03	2		0.25		0.25	0.12		0.12
1565	*Staphylococcus aureus*	0.5	16		1	2	8	0.06	1	0.12	0.5	2	4	8	8		2	2		0.25		0.25	0.12		0.06
1566	*Staphylococcus aureus*	1	1		0.5	1	1	0.06	1	0.06	2	1	4	8	0.25		0.03	2		>32		0.25	0.12		0.12
1567	*Staphylococcus aureus*	0.5	2		1	1	2	0.12	1	0.12	0.5	2	4	16	0.25		0.03	2		0.25		0.25	0.12		0.12
1568	*Staphylococcus aureus*	0.5	16		1	2	16	0.12	1	0.25	0.5	2	4	16	0.25		0.03	2		0.25		0.25	1		0.12
1569	*Staphylococcus aureus*	0.5	16		1	2	16	0.06	1	≤0.06	0.5	2	4	8	0.12		0.03	2		16		0.25	0.25		0.06
1570	*Staphylococcus aureus*	0.5	≤0.03		0.12	0.5	0.06	0.06	1	0.12	0.5	2	4	8	8		1	2		0.25		0.25	0.25		0.12
1571	*Staphylococcus aureus*	0.5	16		1	2	8	0.12	1	0.12	0.5	2	4	16	0.25		0.06	2		>32		0.25	0.25		2
1572	*Staphylococcus aureus*	0.5	16		1	2	16	0.06	1	0.12	0.5	2	4	8	2		1	2		0.25		0.25	0.12		0.06
1573	*Staphylococcus aureus*	0.5	32		0.5	1	8	0.12	1	0.25	2	2	4	16	0.12		0.03	2		>32		0.25	0.12		0.12
1574	*Staphylococcus aureus*	0.25	32		1	1	32	0.12	1	0.06	0.5	4	1	4	0.25		0.03	2		0.25		0.25	0.12		0.06
1575	*Staphylococcus aureus*	0.5	0.25		0.25	1	0.25	0.12	0.5	0.12	0.5	2	4	16	0.25		0.06	2		>32		16	0.25		0.06
1576	*Staphylococcus aureus*	2	64		8	2	32	0.25	0.5	0.12	0.25	2	8	16	0.5		0.06	2		0.25		0.5	0.25		0.06
1577	*Staphylococcus aureus*	0.5	2		0.5	1	2	0.25	0.5	0.12	0.5	2	4	16	0.25		0.06	4		0.25		0.5	0.25		0.06
1578	*Staphylococcus aureus*	0.5	8		1	2	2	0.12	1	0.12	0.5	2	4	16	1		0.12	2		>32		0.25	0.25		0.12
1579	*Staphylococcus aureus*	1	64		1	2	32	0.12	0.5	0.12	0.5	2	8	16	0.25		0.06	2		0.25		0.5	0.25		0.06
1580	*Staphylococcus aureus*	0.25	16		1	2	32	0.25	0.5	0.25	0.5	2	4	16	0.12		0.03	2		0.12		0.25	0.25		0.06
1581	*Staphylococcus aureus*	0.25	16		1	2	16	0.12	1	0.12	0.25	2	4	16	0.25		0.03	2		0.25		0.25	0.25		0.12
1582	*Staphylococcus aureus*	0.5	32		1	2	32	0.12	1	0.25	1	2	4	8	0.25		0.06	4		0.25		64	0.5		0.06
1583	*Staphylococcus aureus*	0.5	32		1	2	16	0.12	0.5	0.5	0.5	2	8	16	0.25		0.06	2		0.12		0.25	0.12		0.06
1584	*Staphylococcus aureus*	0.5	8		1	1	8	0.12	0.5	0.25	0.5	1	4	8	0.12		0.06	2		0.25		0.25	0.12		0.12
1585	*Staphylococcus aureus*	0.5	4		1	2	4	0.25	1	0.12	1	2	4	8	0.12		0.03	2		0.25		0.25	0.12		0.12
1586	*Staphylococcus aureus*	0.5	64		2	0.5	>32	0.25	1	0.25	0.5	2	4	16	0.12		0.03	2		0.25		0.5	0.25		0.06
1587	*Staphylococcus aureus*	0.5	4		1	≤0.03	2	0.12	1	0.25	0.5	2	4	16	0.25		0.06	2		0.25		0.25	0.25		0.12
1588	*Staphylococcus aureus*	0.25	0.06		0.5	0.12	0.25	0.12	0.5	0.06	1	2	4	16	0.12		0.03	2		0.25		0.25	0.12		0.12
1589	*Staphylococcus aureus*	0.25	16		1	0.06	16	0.12	1	0.06	1	2	4	16	0.25		0.12	2		0.25		0.5	0.25		0.12
1590	*Staphylococcus aureus*	1	64		1	0.25	>32	0.12	1	0.06	0.5	1	4	8	0.25		0.06	2		>32		0.25	0.12		0.06
1591	*Staphylococcus aureus*	0.5	8		1	2	8	0.25	1	0.06	0.5	2	4	16	0.25		0.06	2		0.12		0.25	0.12		0.12
1592	*Staphylococcus aureus*	0.25	32		2	2	32	0.25	0.5	0.12	0.5	2	4	16	0.12		0.03	2		0.25		0.25	0.25		0.12
1593	*Staphylococcus aureus*	0.25	32		1	2	16	0.12	1	0.12	0.5	2	4	16	0.25		0.03	2		0.25		0.25	0.25		0.06
1594	*Staphylococcus aureus*	0.5	32		1	2	32	0.12	0.5	0.12	0.5	2	4	8	0.25		0.03	2		32		0.5	0.12		0.06
1595	*Staphylococcus aureus*	0.5	1		0.5	1	1	0.12	1	0.12	0.5	2	4	16	0.25		0.06	2		0.25		0.5	0.25		0.06
1596	*Staphylococcus aureus*	1	4		0.25	1	0.5	0.12	1	0.5	1	1	2	8	0.25		0.03	2		>32		0.25	0.12		0.06
1597	*Staphylococcus aureus*	0.25	4		0.5	1	2	0.12	1	0.06	1	2	4	8	0.12		0.015	2		32		16	0.25		0.12
1598	*Staphylococcus aureus*	0.25	8		0.5	1	8	0.06	1	0.12	0.5	1	2	8	0.25		0.06	2		0.12		0.12	0.12		0.06

Isolate Number	*Staphylococcus* species	MIC (µg/ml) Values																							
			β Lactams					Carbapenem	Glycopeptides			Cephalosporins			Flouroquinolones			Oxazolidnone	Macrolides			Tetracyclines		Aminoglycoside	Antifolate
		XF-73	Penicillin	Oxacillin	Amoxicillin Clavulanate	Piperacillin/Tazobactam	Ampicillin	Meropenem	Vancomycin	Dalbavancin	Teicoplanin	Cefuroxime	Ceftriaxone	Ceftazidime	Levofloxacin	Ciprofloxacin	Moxifloxacin	Linezolid	Clindamycin	Clarithromycin	Erythromycin	Tetracycline	Tigecycline	Gentamicin	Trimethoprim/ Sulfmethoxazole
1599	*Staphylococcus aureus*	1	64		0.5	1	>32	0.06	1	0.12	0.5	1	2	8	0.12		0.03	2		0.12		0.25	0.12		0.03
1600	*Staphylococcus aureus*	0.5	8		1	1	8	0.06	1	0.12	0.5	2	2	8	0.12		0.03	1		0.12		64	0.25		0.03
1601	*Staphylococcus aureus*	0.5	4		1	1	4	0.06	0.5	≤0.06	0.5	1	4	8	0.25		0.03	2		0.12		0.25	0.12		0.06
1602	*Staphylococcus aureus*	0.25	32		1	2	32	0.06	1	≤0.06	0.5	1	4	16	0.25		0.06	2		0.25		0.25	0.12		0.06
1603	*Staphylococcus aureus*	0.5	≤0.03		0.25	0.5	0.06	0.03	1	0.12	0.5	1	2	8	0.25		0.06	1		0.12		0.25	0.12		0.06
1604	*Staphylococcus aureus*	2	16		0.5	1	16	0.12	1	0.12	0.5	1	2	16	0.12		0.06	2		0.5		0.25	0.12		0.06
1605	*Staphylococcus aureus*	0.5	16		1	1	16	0.12	1	0.12	1	1	2	8	0.12		0.03	0.5		0.06		32	0.25		0.06
1606	*Staphylococcus aureus*	0.5	16		1	1	8	0.12	1	0.12	0.5	2	2	8	0.25		0.06	1		0.12		64	0.5		0.06
1607	*Staphylococcus aureus*	2	0.5		0.5	1	1	0.06	1	0.12	0.5	1	4	8	0.25		0.12	1		0.12		32	0.25		0.25
1608	*Staphylococcus aureus*	0.25	0.06		0.12	0.5	0.03	0.12	1	≤0.06	0.5	1	2	16	0.25		0.03	1		0.12		0.06	0.12		0.03
1609	*Staphylococcus aureus*	0.5	8		0.5	1	4	0.12	1	0.12	1	1	2	16	0.25		0.03	1		>32		0.06	0.12		0.06
1610	*Staphylococcus aureus*	0.5	8		0.5	1	8	0.06	1	≤0.06	0.5	1	2	16	0.25		0.03	1		0.12		64	0.25		0.03
1611	*Staphylococcus aureus*	0.5	2		1	2	2	0.12	1	≤0.06	0.5	2	4	16	0.25		0.06	2		0.25		0.25	2		0.06
1612	*Staphylococcus aureus*	0.5	16		1	2	16	0.12	1	≤0.06	1	2	4	16	0.12		0.06	2		0.25		0.25	0.12		0.12
1613	*Staphylococcus aureus*	1	32		1	1	16	0.25	1	≤0.06	0.5	2	4	16	0.12		0.03	2		0.25		0.25	0.5		0.06
1614	*Staphylococcus aureus*	1	16		1	2	16	0.06	1	0.12	0.5	2	4	16	0.5		0.12	4		0.25		0.5	2		0.12
1615	*Staphylococcus aureus*	2	32		1	2	32	0.25	1	≤0.06	0.5	2	4	8	0.25		0.06	2		0.25		0.12	1		0.06
1616	*Staphylococcus aureus*	0.5	>64		2	2	>32	0.12	1	0.12	1	2	4	16	0.25		0.06	2		0.25		64	1		0.12
1617	*Staphylococcus aureus*	0.5	0.06		0.25	1	0.12	0.12	1	≤0.06	1	2	4	8	0.25		0.06	1		0.12		32	1		0.06
1618	*Staphylococcus aureus*	0.5	16		1	2	8	0.12	1	0.12	0.5	2	4	16	0.25		0.06	2		0.25		0.12	0.5		0.12
1619	*Staphylococcus aureus*	0.5	16		1	2	16	0.12	2	≤0.06	1	2	4	16	0.12		0.03	2		0.25		>64	0.5		0.06
1620	*Staphylococcus aureus*	0.5	16		1	2	32	0.12	1	≤0.06	1	2	4	8	0.12		0.03	2		0.25		0.25	1		0.12
1621	*Staphylococcus aureus*	1	4		1	1	2	0.12	1	≤0.06	1	1	4	8	0.25		0.03	2		0.25		0.25	0.5		0.12
1622	*Staphylococcus aureus*	1	2		0.5	1	2	0.12	1	0.12	1	2	4	8	0.12		0.03	1		>32		32	0.25		0.12
1623	*Staphylococcus aureus*	0.5	1		0.5	1	1	0.12	0.5	0.5	0.5	2	4	16	4		1	2		>32		0.25	0.12		0.25
1624	*Staphylococcus aureus*	0.5	≤0.03		0.25	0.5	0.12	0.06	1	≤0.06	1	0.5	2	8	0.25		0.03	2		0.06		0.25	0.12		0.06
1625	*Staphylococcus aureus*	0.5	32		1	2	4	0.12	1	≤0.06	0.5	1	4	16	0.25		0.06	2		>32		0.25	0.12		0.12
1626	*Staphylococcus aureus*	0.5	16		0.5	1	4	0.12	1	≤0.06	0.5	1	4	16	0.25		0.03	2		0.12		0.25	0.25		0.12
1627	*Staphylococcus aureus*	0.5	≤0.03		0.12	0.5	0.06	0.06	0.5	≤0.06	0.5	2	4	8	0.12		0.03	1		>32		0.12	0.12		0.5
1628	*Staphylococcus aureus*	0.5	16		0.5	0.5	8	0.12	0.5	≤0.06	0.5	1	4	8	0.12		0.03	2		0.12		0.25	0.25		0.12
1629	*Staphylococcus aureus*	0.5	1		1	1	1	0.12	0.5	0.12	0.25	1	4	8	0.12		0.03	2		0.12		0.25	0.25		0.12
1630	*Staphylococcus aureus*	0.5	1		1	1	1	0.06	0.5	0.12	0.25	2	4	8	0.12		0.03	2		0.25		0.25	0.25		0.06
1631	*Staphylococcus aureus*	0.5	1		0.5	1	1	0.03	1	0.12	0.5	2	4	8	0.25		0.06	2		>32		64	0.5		0.12
1632	*Staphylococcus aureus*	0.5	1		0.5	1	1	0.12	1	≤0.06	0.25	1	2	8	0.25		0.03	2		0.12		0.25	1		0.12
1633	*Staphylococcus aureus*	0.5	0.25		0.5	1	1	0.12	1	≤0.06	0.5	1	4	8	0.25		0.06	2		0.25		0.25	0.25		0.06
1634	*Staphylococcus aureus*	0.25	16		2	4	16	0.25	1	≤0.06	0.5	4	8	16	0.25		0.03	2		0.12		0.25	0.25		0.06
1635	*Staphylococcus aureus*	1	4		1	1	4	0.06	1	≤0.06	0.5	1	2	8	0.12		0.03	2		0.25		0.25	0.25		0.06
1636	*Staphylococcus aureus*	0.5	16		1	2	16	0.25	1	≤0.06	2	2	4	8	0.12		0.03	2		0.25		0.12	0.12		0.06
1637	*Staphylococcus aureus*	0.25	32		1	2	16	0.12	1	≤0.06	1	2	4	8	0.25		0.06	4		>32		0.5	0.25		0.06
1638	*Staphylococcus aureus*	0.25	16		0.5	1	8	0.25	1	≤0.06	0.5	1	4	8	0.12		0.03	2		0.25		0.25	0.12		0.12
1639	*Staphylococcus aureus*	1	1		0.5	1	2	0.25	1	0.12	1	2	4	16	0.12		0.03	2		0.25		0.25	0.12		0.12
1640	*Staphylococcus aureus*	0.25	32		1	2	32	0.06	1	≤0.06	1	2	4	8	0.25		0.03	2		>32		0.25	0.12		0.06
1641	*Staphylococcus aureus*	1	16		0.5	1	8	0.12	1	0.5	1	2	4	8	0.5		0.03	2		0.25		0.25	0.12		0.12
1642	*Staphylococcus aureus*	0.5	≤0.03		0.12	0.5	0.06	0.12	1	≤0.06	0.5	2	4	8	0.25		0.06	2		0.25		0.12	0.12		0.12
1643	*Staphylococcus aureus*	0.25	32		1	2	16	0.12	1	0.12	0.5	2	4	8	0.25		0.06	2		0.25		0.25	0.12		0.12
1644	*Staphylococcus aureus*	0.5	4		0.5	1	2	0.06	1	0.12	0.5	2	4	8	0.25		0.03	2		0.25		0.12	0.06		0.06
1645	*Staphylococcus aureus*	0.5	0.5		0.25	1	0.5	0.12	1	0.12	0.25	1	2	16	0.5		0.12	2		0.25		16	0.12		0.06

Isolate Number	*Staphylococcus* species	MIC (µg/ml) Values																							
			β Lactams					Carbapenem	Glycopeptides			Cephalosporins			Flouroquinolones			Oxazolidnone	Macrolides			Tetracyclines		Aminoglycoside	Antifolate
		XF-73	Penicillin	Oxacillin	Amoxicillin Clavulanate	Piperacillin/Tazobactam	Ampicillin	Meropenem	Vancomycin	Dalbavancin	Teicoplanin	Cefuroxime	Ceftriaxone	Ceftazidime	Levofloxacin	Ciprofloxacin	Moxifloxacin	Linezolid	Clindamycin	Clarithromycin	Erythromycin	Tetracycline	Tigecycline	Gentamicin	Trimethoprim/ Sulfmethoxazole
1646	*Staphylococcus aureus*	0.5	32		0.5	0.5	16	0.12	0.5	≤0.06	0.25	1	2	4	0.25		0.03	1		0.12		0.12	0.12		0.03
1647	*Staphylococcus aureus*	0.5	≤0.03		0.25	1	0.12	0.12	1	0.12	0.5	1	4	2	0.25		0.06	2		0.25		0.12	0.12		0.06
1648	*Staphylococcus aureus*	0.5	0.12		0.25	0.5	0.12	0.06	1	0.5	1	1	4	2	0.25		0.06	2		0.12		0.25	0.12		0.12
1649	*Staphylococcus aureus*	0.5	32		0.5	0.5	16	0.06	1	0.12	0.5	1	2	2	0.12		0.03	2		>32		0.12	0.12		0.06
1650	*Staphylococcus aureus*	0.5	8		1	2	4	0.06	0.5	≤0.06	0.25	2	4	4	0.25		0.06	2		0.12		0.12	0.12		0.06
1651	*Staphylococcus aureus*	0.5	>64		2	4	>32	0.06	1	≤0.06	0.5	1	2	8	0.25		0.06	2		0.12		0.12	0.12		0.06
1652	*Staphylococcus aureus*	0.5	16		0.5	0.5	2	0.06	1	0.12	0.5	1	2	8	0.12		0.015	1		0.12		0.12	0.06		0.03
1653	*Staphylococcus aureus*	0.5	32		0.5	0.5	16	0.06	0.5	0.12	0.25	1	2	16	0.12		0.03	2		8		0.12	0.12		0.03
1654	*Staphylococcus aureus*	0.25	8		0.25	0.5	4	0.12	0.5	0.12	0.25	1	4	16	0.25		0.03	2		0.12		0.12	0.06		0.06
1655	*Staphylococcus aureus*	0.25	0.5		0.25	1	0.5	0.06	1	0.12	0.25	1	2	16	0.5		0.25	2		0.12		16	0.12		0.06
1656	*Staphylococcus aureus*	0.25	≤0.03		0.06	0.12	0.06	0.03	0.25	≤0.06	0.12	0.5	1	4	0.25		0.03	1		0.06		0.06	0.06		0.03
1657	*Staphylococcus aureus*	0.5	8		2	1	8	0.12	2	≤0.06	0.25	1	4	16	0.12		0.03	1		0.12		0.12	0.12		0.03
1658	*Staphylococcus aureus*	0.5	>64		0.5	1	32	0.12	1	≤0.06	0.5	1	4	16	0.25		0.03	2		0.5		0.12	0.12		0.06
1659	*Staphylococcus aureus*	0.5	0.5		0.25	0.5	1	0.06	0.5	≤0.06	0.5	1	2	8	0.12		0.015	2		0.12		0.12	0.12		0.06
1660	*Staphylococcus aureus*	0.25	64		0.5	0.5	>32	0.12	1	≤0.06	0.5	1	2	16	0.25		0.03	2		>32		0.12	0.12		0.06
1661	*Staphylococcus aureus*	0.25	4		1	1	2	0.12	1	0.12	0.5	2	4	16	0.25		0.03	2		0.25		0.12	0.25		0.06
1662	*Staphylococcus aureus*	0.5	32		0.5	0.5	16	0.12	1	0.12	0.5	1	2	16	0.5		0.06	1		32		0.25	0.12		2
1663	*Staphylococcus aureus*	0.5	16		1	2	8	0.06	1	0.12	0.25	2	4	16	0.25		0.03	2		0.12		0.12	0.12		0.06
1664	*Staphylococcus aureus*	0.5	16		0.5	0.5	8	0.12	1	0.12	0.5	1	2	16	0.12		0.015	1		8		0.12	0.12		0.06
1665	*Staphylococcus aureus*	0.25	≤0.03		0.06	0.12	0.03	0.03	0.25	≤0.06	0.12	0.5	1	2	0.12		0.03	1		0.06		0.06	0.03		0.03
1666	*Staphylococcus aureus*	0.25	32		0.5	0.5	32	0.06	1	0.12	0.5	1	2	2	0.12		0.03	2		8		0.12	0.12		0.06
1667	*Staphylococcus aureus*	1	32		1	1	32	0.12	1	0.12	0.5	2	4	16	0.25		0.03	2		0.12		0.12	0.12		0.06
1668	*Staphylococcus aureus*	0.5	1		0.25	1	1	0.12	1	0.12	0.5	2	4	16	0.5		0.12	1		0.25		16	0.12		0.06
1669	*Staphylococcus aureus*	0.5	16		0.5	0.5	8	0.06	1	0.12	0.5	1	2	8	0.12		0.03	1		0.12		0.12	0.12		0.06
1670	*Staphylococcus aureus*	0.5	32		0.5	0.5	16	0.06	0.5	0.12	0.5	1	2	8	0.25		0.03	2		>32		0.12	0.25		0.06
1671	*Staphylococcus aureus*	0.5	4		0.5	2	4	0.06	0.5	0.12	0.25	2	4	8	0.25		0.06	2		0.12		0.25	0.12		0.06
1672	*Staphylococcus aureus*	0.5	32		0.5	1	16	0.06	0.5	≤0.06	0.5	1	2	4	0.25		0.06	2		0.12		0.12	0.12		0.12
1673	*Staphylococcus aureus*	0.5	0.06		0.06	0.5	0.06	0.06	0.5	0.12	1	1	2	4	0.5		0.06	2		0.12		0.25	0.25		0.12
1674	*Staphylococcus aureus*	0.5	16		0.5	0.5	8	0.06	1	0.12	0.5	1	2	4	0.12		0.03	2		>32		0.25	0.25		0.06
1675	*Staphylococcus aureus*	0.5	0.25		0.25	0.5	0.5	0.06	1	0.12	1	1	4	16	0.25		0.06	2		0.25		32	0.25		0.12
1676	*Staphylococcus aureus*	0.5	32		0.5	0.5	32	0.12	0.5	0.12	0.25	1	2	4	0.25		0.03	1		0.12		0.12	0.12		0.06
1677	*Staphylococcus aureus*	0.5	32		0.5	0.5	16	0.12	0.5	0.12	0.5	1	2	4	0.25		0.03	2		>32		0.12	0.12		0.06
1678	*Staphylococcus aureus*	0.5	2		0.5	1	2	0.06	1	0.25	1	2	4	16	0.25		0.06	2		0.25		0.25	0.12		0.06
1679	*Staphylococcus aureus*	0.5	0.5		0.25	0.5	0.5	0.06	0.5	≤0.06	0.5	1	2	8	0.12		0.03	2		0.25		0.12	0.12		0.06
1680	*Staphylococcus aureus*	0.5	8		0.5	1	8	0.06	0.5	≤0.06	0.5	2	4	4	0.25		0.06	2		0.12		0.25	0.25		0.06
1681	*Staphylococcus aureus*	0.5	16		1	0.5	8	0.5	1	0.12	0.5	2	8	8	0.5		0.06	1		>32		64	0.12		2
1682	*Staphylococcus aureus*	0.5	1		0.25	0.5	1	0.06	1	0.12	0.5	2	4	16	1		0.25	2		0.25		32	0.25		0.06
1683	*Staphylococcus aureus*	0.5	8		1	1	8	0.12	0.5	0.25	0.5	1	2	4	0.25		0.03	2		0.12		0.12	0.12		0.06
1684	*Staphylococcus aureus*	0.5		0.5					1	0.12	0.25					0.25		2	≤ 0.5		0.25		0.06	0.25	≤ 0.5
1685	*Staphylococcus aureus*	0.5		0.5					1	0.12	0.5					0.25		2	≤ 0.5		0.25		0.06	0.5	≤ 0.5
1686	*Staphylococcus aureus*	0.5		0.25					1	≤0.06	0.25					0.25		2	≤ 0.5		0.25		0.12	0.25	≤ 0.5
1687	*Staphylococcus aureus*	0.5		0.25					1	≤0.06	0.25					0.25		2	≤ 0.5		0.25		0.25	0.5	≤ 0.5
1688	*Staphylococcus aureus*	0.5		0.5					1	≤0.06	0.5					0.5		2	≤ 0.5		0.25		0.06	0.25	≤ 0.5
1689	*Staphylococcus aureus*	1		0.25					0.5	≤0.06	0.25					0.5		2	≤ 0.5		0.25		0.06	0.25	≤ 0.5
1690	*Staphylococcus aureus*	0.5		0.25					1	0.12	0.5					0.25		2	≤ 0.5		0.25		0.06	0.25	≤ 0.5
1691	*Staphylococcus aureus*	0.5		0.25					1	≤0.06	0.5					0.25		2	≤ 0.5		0.5		0.06	0.25	≤ 0.5
1692	*Staphylococcus aureus*	1		0.5					1	0.12	0.5					0.25		2	≤ 0.5		0.25		0.25	0.25	≤ 0.5

Table s2: MIC Data for the Staphylococcus species tested

Isolate Number	*Staphylococcus* species	MIC (µg/ml) Values																							
			β Lactams					Carbapenem	Glycopeptides			Cephalosporins			Flouroquinolones			Oxazolidnone	Macrolides			Tetracyclines		Aminoglycoside	Antifolate
		XF-73	Penicillin	Oxacillin	Amoxicillin Clavulanate	Piperacillin/Tazobactam	Ampicillin	Meropenem	Vancomycin	Dalbavancin	Teicoplanin	Cefuroxime	Ceftriaxone	Ceftazidime	Levofloxacin	Ciprofloxacin	Moxifloxacin	Linezolid	Clindamycin	Clarithromycin	Erythromycin	Tetracycline	Tigecycline	Gentamicin	Trimethoprim/ Sulfmethoxazole
1693	*Staphylococcus aureus*	0.5		0.5					1	≤0.06	0.25					0.25		2	≤ 0.5		0.25		0.06	0.5	≤ 0.5
1694	*Staphylococcus aureus*	0.5		0.5					1	0.25	0.5					0.25		2	≤ 0.5		0.25		0.06	0.25	≤ 0.5
1695	*Staphylococcus aureus*	0.5		0.5					1	0.12	0.5					0.5		2	≤ 0.5		0.25		0.06	0.5	≤ 0.5
1696	*Staphylococcus aureus*	0.5		0.5					2	≤0.06	0.5					0.5		4	≤ 0.5		0.5		0.12	0.5	≤ 0.5
1697	*Staphylococcus aureus*	0.5		1					1	0.25	0.5					8		4	≤ 0.5		0.5		0.06	1	≤ 0.5
1698	*Staphylococcus aureus*	2		1					0.5	0.12	0.5					0.5		2	≤ 0.5		0.25		0.12	0.25	≤ 0.5
1699	*Staphylococcus aureus*	1		0.5					1	0.12	0.5					0.25		4	≤ 0.5		> 16		0.12	0.25	≤ 0.5
1700	*Staphylococcus aureus*	1		0.5					1	0.12	0.25					8		2	≤ 0.5		16		0.5	> 16	≤ 0.5
1701	*Staphylococcus aureus*	1		0.5					1	≤0.06	0.5					0.5		2	≤ 0.5		0.25		0.12	0.5	≤ 0.5
1702	*Staphylococcus aureus*	1		0.25					1	0.12	0.5					0.5		2	≤ 0.5		0.25		0.5	> 16	≤ 0.5
1703	*Staphylococcus aureus*	1		1					1	0.12	0.5					0.5		2	≤ 0.5		0.25		0.12	0.5	≤ 0.5
1704	*Staphylococcus aureus*	1		0.5					1	0.25	0.5					≤ 0.06		4	≤ 0.5		> 16		0.12	0.25	≤ 0.5
1705	*Staphylococcus aureus*	1		0.5					1	0.12	0.25					0.5		2	≤ 0.5		0.25		0.06	1	≤ 0.5
1706	*Staphylococcus aureus*	1		0.5					0.5	0.25	0.5					0.5		2	≤ 0.5		0.25		0.06	0.25	≤ 0.5
1707	*Staphylococcus aureus*	1		0.5					1	0.12	0.25					0.25		4	≤ 0.5		> 16		0.12	0.25	≤ 0.5
1708	*Staphylococcus aureus*	0.25		0.5					1	0.12	0.5					0.25		4	≤ 0.5		0.25		0.12	0.5	≤ 0.5
1709	*Staphylococcus aureus*	0.25		0.5					1	0.12	0.5					0.25		2	≤ 0.5		0.25		0.12	0.5	≤ 0.5
1710	*Staphylococcus aureus*	0.5		1					1	0.12	0.5					0.12		2	≤ 0.5		0.5		0.12	2	≤ 0.5
1711	*Staphylococcus aureus*	0.5		0.25					1	0.06	0.25					0.25		2	≤ 0.5		0.25		0.12	0.25	≤ 0.5
1712	*Staphylococcus aureus*	0.5		0.5					1	0.12	0.5					0.5		2	≤ 0.5		0.25		0.12	0.5	≤ 0.5
1713	*Staphylococcus aureus*	0.25		0.25					1	0.12	0.5					0.5		2	≤ 0.5		0.25		0.12	0.25	≤ 0.5
1714	*Staphylococcus aureus*	0.5		0.5					1	0.12	0.5					0.25		2	≤ 0.5		0.25		0.06	0.5	≤ 0.5
1715	*Staphylococcus aureus*	0.25		0.5					0.5	0.12	0.5					0.12		2	≤ 0.5		> 16		0.12	0.25	≤ 0.5
1716	*Staphylococcus aureus*	0.25		0.25					2	0.12	0.5					0.25		2	≤ 0.5		0.25		0.06	0.5	≤ 0.5
1717	*Staphylococcus aureus*	0.5		0.5					1	0.12	0.5					0.25		2	≤ 0.5		0.25		0.25	0.25	≤ 0.5
1718	*Staphylococcus aureus*	0.25		1					1	0.06	0.25					4		2	≤ 0.5		> 16		0.12	0.5	≤ 0.5
1719	*Staphylococcus aureus*	0.5		0.5					1	0.12	0.5					0.25		2	≤ 0.5		0.25		0.12	0.5	≤ 0.5
1720	*Staphylococcus aureus*	1		0.5					1	≤0.06	0.5					0.25		2	≤ 0.5		0.25		0.12	0.5	≤ 0.5
1721	*Staphylococcus aureus*	0.25		0.5					1	0.12	0.5					0.12		2	≤ 0.5		0.25		0.12	1	≤ 0.5
1722	*Staphylococcus aureus*	0.5		0.5					0.5	≤0.06	0.25					0.5		4	≤ 0.5		0.5		0.12	0.5	≤ 0.5
1723	*Staphylococcus aureus*	0.25		0.25					1	0.12	1					> 8		2	≤ 0.5		0.25		0.12	0.25	≤ 0.5
1724	*Staphylococcus aureus*	0.5		0.5					1	0.12	0.5					0.5		2	≤ 0.5		0.25		0.25	0.5	≤ 0.5
1725	*Staphylococcus aureus*	0.5		0.5					1	0.25	1					0.25		2	≤ 0.5		0.25		0.12	0.25	≤ 0.5
1726	*Staphylococcus aureus*	0.5		0.25					1	≤0.06	0.5					0.25		4	≤ 0.5		> 16		0.25	0.5	≤ 0.5
1727	*Staphylococcus aureus*	0.25		0.5					1	0.12	0.5					> 8		2	≤ 0.5		0.25		0.06	0.5	≤ 0.5
1728	*Staphylococcus aureus*	0.25		0.5					1	0.12	0.5					0.25		2	≤ 0.5		> 16		0.12	0.5	≤ 0.5
1729	*Staphylococcus aureus*	0.25		0.5					1	0.12	0.5					0.25		4	≤ 0.5		0.5		0.12	0.5	≤ 0.5
1730	*Staphylococcus aureus*	0.25		0.5					1	≤0.06	0.5					2		2	≤ 0.5		0.5		0.12	0.5	≤ 0.5
1731	*Staphylococcus aureus*	0.5		0.5					1	≤0.06	0.5					0.25		2	≤ 0.5		> 16		0.12	0.5	≤ 0.5
1732	*Staphylococcus aureus*	0.25		0.25					1	0.25	0.5					1		4	≤ 0.5		> 16		0.12	0.5	≤ 0.5
1733	*Staphylococcus aureus*	0.25		0.5					1	0.25	0.5					2		4	≤ 0.5		> 16		0.12	0.25	≤ 0.5
1734	*Staphylococcus aureus*	0.25		0.5					1	≤0.06	0.25					0.12		2	≤ 0.5		0.25		0.06	0.5	≤ 0.5
1735	*Staphylococcus aureus*	0.5		0.25					1	≤0.06	0.25					0.5		2	≤ 0.5		> 16		0.12	0.5	≤ 0.5
1736	*Staphylococcus aureus*	0.5		1					0.5	0.06	0.25					0.5		2	≤ 0.5		0.25		0.12	0.25	≤ 0.5
1737	*Staphylococcus aureus*	0.5		0.5					1	0.12	0.5					0.25		2	≤ 0.5		0.25		0.25	0.5	≤ 0.5
1738	*Staphylococcus aureus*	0.5		0.5					1	0.12	0.5					2		4	≤ 0.5		0.5		0.12	0.5	≤ 0.5
1739	*Staphylococcus aureus*	0.5		0.25					1	0.12	0.25					0.25		4	≤ 0.5		0.5		0.06	0.5	≤ 0.5

Isolate Number	*Staphylococcus* species	MIC (µg/ml) Values																							
			β Lactams					Carbapenem	Glycopeptides			Cephalosporins			Flouroquinolones			Oxazolidnone	Macrolides			Tetracyclines		Aminoglycoside	Antifolate
		XF-73	Penicillin	Oxacillin	Amoxicillin Clavulanate	Piperacillin/Tazobactam	Ampicillin	Meropenem	Vancomycin	Dalbavancin	Teicoplanin	Cefuroxime	Ceftriaxone	Ceftazidime	Levofloxacin	Ciprofloxacin	Moxifloxacin	Linezolid	Clindamycin	Clarithromycin	Erythromycin	Tetracycline	Tigecycline	Gentamicin	Trimethoprim/ Sulfmethoxazole
1740	*Staphylococcus aureus*	0.5		0.25					1	0.12	0.5					0.5		4	≤ 0.5		0.5		0.12	0.5	≤ 0.5
1741	*Staphylococcus aureus*	0.25		0.5					1	0.12	0.5					0.5		2	≤ 0.5		0.25		0.12	0.25	≤ 0.5
1742	*Staphylococcus aureus*	0.25		1					1	0.12	0.5					0.5		2	≤ 0.5		0.25		0.12	0.25	≤ 0.5
1743	*Staphylococcus aureus*	1		0.25					1	≤0.06	0.5					0.5		2	≤ 0.5		0.25		0.06	0.5	≤ 0.5
1744	*Staphylococcus aureus*	0.25		0.25					1	0.12	0.25					0.25		2	≤ 0.5		0.25		0.12	1	≤ 0.5
1745	*Staphylococcus aureus*	0.25		0.5					2	≤0.06	1					0.25		4	≤ 0.5		0.5		0.25	0.25	≤ 0.5
1746	*Staphylococcus aureus*	0.25		0.5					1	0.12	0.25					0.5		4	≤ 0.5		0.5		0.12	0.5	≤ 0.5
1747	*Staphylococcus aureus*	0.5		1					1	0.12	0.5					0.5		2	≤ 0.5		0.25		0.06	1	≤ 0.5
1748	*Staphylococcus aureus*	0.25		0.5					1	0.12	0.25					0.5		2	≤ 0.5		0.25		0.12	0.5	≤ 0.5
1749	*Staphylococcus aureus*	0.5		0.5					1	≤0.06	0.5					1		4	≤ 0.5		0.5		0.12	0.5	≤ 0.5
1750	*Staphylococcus aureus*	0.5		1					1	0.12	0.25					> 8		2	> 4		> 16		0.12	0.5	≤ 0.5
1751	*Staphylococcus aureus*	0.5		0.5					1	0.12	0.25					0.5		1	≤ 0.5		0.25		0.12	> 16	≤ 0.5
1752	*Staphylococcus aureus*	0.25		0.25					1	0.06	0.25					0.25		2	≤ 0.5		0.25		0.06	0.25	≤ 0.5
1753	*Staphylococcus aureus*	0.5		0.5					1	0.06	0.25					0.5		2	2		0.25		0.06	0.5	≤ 0.5
1754	*Staphylococcus aureus*	0.25		0.5					1	0.06	0.5					0.25		2	≤ 0.5		0.25		0.06	0.5	≤ 0.5
1755	*Staphylococcus aureus*	0.25		0.5					1	≤0.06	0.5					0.25		2	≤ 0.5		0.5		0.12	1	≤ 0.5
1756	*Staphylococcus aureus*	0.25		1					1	0.12	0.5					0.5		2	≤ 0.5		0.25		0.12	0.5	≤ 0.5
1757	*Staphylococcus aureus*	0.5		0.25					1	0.06	0.25					0.25		2	≤ 0.5		0.25		0.06	0.5	≤ 0.5
1758	*Staphylococcus aureus*	0.5		0.5					1	≤0.06	0.5					> 8		2	≤ 0.5		> 16		0.25	0.5	≤ 0.5
1759	*Staphylococcus aureus*	0.5		0.5					1	0.12	0.5					0.25		2	≤ 0.5		> 16		0.12	0.5	≤ 0.5
1760	*Staphylococcus aureus*	0.25		0.5					1	≤0.06	0.5					0.5		4	≤ 0.5		0.5		0.12	0.5	≤ 0.5
1761	*Staphylococcus aureus*	0.5		2					1	0.12	0.5					0.25		2	≤ 0.5		> 16		0.25	0.5	≤ 0.5
1762	*Staphylococcus aureus*	0.5		1					1	≤0.06	0.5					0.25		2	≤ 0.5		0.25		0.06	0.5	≤ 0.5
1763	*Staphylococcus aureus*	0.5		0.25					1	0.12	0.25					0.25		2	≤ 0.5		0.25		0.06	0.5	≤ 0.5
1764	*Staphylococcus aureus*	0.5		0.25					1	0.12	0.5					0.25		2	≤ 0.5		0.5		0.12	0.5	≤ 0.5
1765	*Staphylococcus aureus*	0.5		0.25					1	0.12	0.5					0.5		2	≤ 0.5		0.5		0.06	0.12	≤ 0.5
1766	*Staphylococcus aureus*	0.25		0.5					1	0.12	0.5					0.25		2	≤ 0.5		0.5		0.12	0.25	≤ 0.5
1767	*Staphylococcus aureus*	0.5		0.5					1	0.12	0.25					0.25		2	≤ 0.5		0.5		0.12	0.5	≤ 0.5
1768	*Staphylococcus aureus*	0.5		0.25					1	0.12	0.5					> 8		2	≤ 0.5		> 16		0.12	2	≤ 0.5
1769	*Staphylococcus aureus*	0.5		0.25					1	≤0.06	0.25					0.25		2	≤ 0.5		0.25		0.06	0.5	≤ 0.5
1770	*Staphylococcus aureus*	0.25		0.25					1	≤0.06	0.25					0.5		4	≤ 0.5		> 16		0.06	0.5	≤ 0.5
1771	*Staphylococcus aureus*	0.5		0.5					1	0.12	0.5					0.5		2	≤ 0.5		0.25		0.06	0.25	≤ 0.5
1772	*Staphylococcus aureus*	0.5		0.25					1	≤0.06	0.25					0.12		1	≤ 0.5		> 16		0.06	0.25	≤ 0.5
1773	*Staphylococcus aureus*	0.25		0.5					1	0.12	0.5					0.25		4	≤ 0.5		0.25		0.06	0.5	≤ 0.5
1774	*Staphylococcus aureus*	0.5		0.25					1	≤0.06	0.25					≤ 0.06		2	≤ 0.5		0.5		0.06	0.25	≤ 0.5
1775	*Staphylococcus aureus*	0.25		0.12					1	0.12	0.5					0.12		2	≤ 0.5		0.25		0.06	0.5	≤ 0.5
1776	*Staphylococcus aureus*	0.5		0.5					2	≤0.06	0.5					0.25		4	≤ 0.5		0.5		0.12	0.5	≤ 0.5
1777	*Staphylococcus aureus*	0.25		0.5					1	≤0.06	0.5					0.5		4	≤ 0.5		0.5		0.12	2	≤ 0.5
1778	*Staphylococcus aureus*	0.5		0.5					1	≤0.06	0.25					1		2	≤ 0.5		0.25		0.06	0.5	≤ 0.5
1779	*Staphylococcus aureus*	1		0.5					1	0.12	0.5					8		2	≤ 0.5		0.25		0.06	0.5	≤ 0.5
1780	*Staphylococcus aureus*	0.5		1					1	≤0.06	1					0.5		2	≤ 0.5		0.25		0.12	0.5	≤ 0.5
1781	*Staphylococcus aureus*	0.5		0.5					1	≤0.06	0.5					0.25		2	≤ 0.5		> 16		0.06	0.25	≤ 0.5
1782	*Staphylococcus aureus*	0.5		0.5					1	0.12	0.5					0.25		2	≤ 0.5		0.25		0.06	0.5	≤ 0.5
1783	*Staphylococcus aureus*	0.5		1					1	0.12	0.5					> 8		2	≤ 0.5		> 16		0.12	> 16	≤ 0.5
1784	*Staphylococcus aureus*	0.5		0.5					1	0.25	1					0.12		2	≤ 0.5		> 16		0.12	0.5	≤ 0.5
1785	*Staphylococcus aureus*	0.25		0.5					1	≤0.06	1					0.25		2	≤ 0.5		0.25		0.06	0.5	≤ 0.5
1786	*Staphylococcus aureus*	0.5		1					1	0.12	0.5					≤ 0.06		2	≤ 0.5		0.25		0.06	0.5	≤ 0.5

		MIC (µg/ml) Values																							
			β Lactams					Carbapenem	Glycopeptides			Cephalosporins			Flouroquinolones			Oxazolidnone	Macrolides			Tetracyclines		Aminoglycoside	Antifolate
Isolate Number	***Staphylococcus* species**	XF-73	Penicillin	Oxacillin	Amoxicillin Clavulanate	Piperacillin/Tazobactam	Ampicillin	Meropenem	Vancomycin	Dalbavancin	Teicoplanin	Cefuroxime	Ceftriaxone	Ceftazidime	Levofloxacin	Ciprofloxacin	Moxifloxacin	Linezolid	Clindamycin	Clarithromycin	Erythromycin	Tetracycline	Tigecycline	Gentamicin	Trimethoprim/ Sulfmethoxazole
1787	*Staphylococcus aureus*	0.25		0.5					1	≤0.06	0.25					≤ 0.06		2	≤ 0.5		0.25		0.06	0.5	≤ 0.5
1788	*Staphylococcus aureus*	0.5		1					1	0.12	0.5					0.12		2	≤ 0.5		0.25		0.06	0.5	≤ 0.5
1789	*Staphylococcus aureus*	0.5		0.5					1	0.12	0.5					0.25		2	≤ 0.5		0.25		0.06	0.5	≤ 0.5
1790	*Staphylococcus aureus*	0.5		0.5					1	0.12	0.25					0.25		2	≤ 0.5		0.25		0.12	0.5	≤ 0.5
1791	*Staphylococcus aureus*	0.25		1					1	0.12	0.25					0.5		2	≤ 0.5		0.5		0.12	0.5	≤ 0.5
1792	*Staphylococcus aureus*	0.25		1					2	0.12	1					0.25		4	≤ 0.5		> 16		0.12	0.5	≤ 0.5
1793	*Staphylococcus aureus*	0.5		1					1	0.12	0.5					0.25		2	≤ 0.5		0.25		0.06	0.5	≤ 0.5
1794	*Staphylococcus aureus*	0.5		0.25					1	0.12	0.5					0.25		2	≤ 0.5		0.5		0.12	0.5	≤ 0.5
1795	*Staphylococcus aureus*	0.5		0.5					1	0.12	0.5					0.25		2	≤ 0.5		0.25		0.06	0.5	≤ 0.5
1796	*Staphylococcus aureus*	0.5		0.25					1	0.12	0.5					0.25		2	≤ 0.5		0.5		0.12	0.5	≤ 0.5
1797	*Staphylococcus aureus*	0.5		0.5					1	≤0.06	0.5					0.5		2	≤ 0.5		0.5		0.12	1	≤ 0.5
1798	*Staphylococcus aureus*	0.5		0.5					1	≤0.06	0.5					0.5		2	≤ 0.5		0.5		0.12	0.25	≤ 0.5
1799	*Staphylococcus aureus*	0.5		0.12					1	≤0.06	0.25					0.25		2	≤ 0.5		0.25		0.06	0.25	≤ 0.5
1800	*Staphylococcus aureus*	0.25		0.5					1	0.12	0.25					0.12		2	> 4		> 16		0.06	0.5	≤ 0.5
1801	*Staphylococcus aureus*	0.5		0.25					1	≤0.06	0.5					0.5		2	≤ 0.5		0.25		0.12	0.5	≤ 0.5
1802	*Staphylococcus aureus*	0.25		0.25					1	≤0.06	0.5					0.5		2	≤ 0.5		0.5		0.12	0.5	≤ 0.5
1803	*Staphylococcus aureus*	0.5		0.25					1	≤0.06	0.25					0.25		2	≤ 0.5		0.25		0.06	> 16	≤ 0.5
1804	*Staphylococcus aureus*	0.5		0.25					1	≤0.06	0.25					0.25		2	≤ 0.5		0.5		0.12	0.5	≤ 0.5
1805	*Staphylococcus aureus*	0.25		0.25					1	≤0.06	0.5					0.25		2	> 4		> 16		0.12	0.5	≤ 0.5
1806	*Staphylococcus aureus*	0.5		0.5					1	≤0.06	0.5					0.5		4	≤ 0.5		0.5		0.12	0.5	≤ 0.5
1807	*Staphylococcus aureus*	0.5		0.5					1	≤0.06	0.5					0.5		4	≤ 0.5		4		0.12	0.5	≤ 0.5
1808	*Staphylococcus aureus*	0.25		1					1	≤0.06	0.5					1		4	≤ 0.5		0.5		0.5	0.5	≤ 0.5
1809	*Staphylococcus aureus*	0.5		0.5					1	≤0.06	0.5					0.5		4	≤ 0.5		> 16		0.25	0.25	≤ 0.5
1810	*Staphylococcus aureus*	0.25		0.5					2	≤0.06	0.5					0.25		2	≤ 0.5		0.5		0.12	0.5	≤ 0.5
1811	*Staphylococcus aureus*	1		0.25					1	≤0.06	0.25					0.5		2	≤ 0.5		0.25		0.12	0.5	≤ 0.5
1812	*Staphylococcus aureus*	0.5		0.5					1	≤0.06	0.5					0.25		2	≤ 0.5		0.5		0.12	0.5	≤ 0.5
1813	*Staphylococcus aureus*	0.5		0.5					2	≤0.06	0.5					0.25		4	≤ 0.5		0.5		0.12	0.5	≤ 0.5
1814	*Staphylococcus aureus*	0.5		0.25					1	≤0.06	0.25					0.25		2	≤ 0.5		0.5		0.12	0.25	≤ 0.5
1815	*Staphylococcus aureus*	0.5		1					1	≤0.06	0.25					0.25		2	≤ 0.5		0.5		0.12	0.5	≤ 0.5
1816	*Staphylococcus aureus*	0.5		0.25					1	≤0.06	0.25					0.5		2	≤ 0.5		0.25		0.12	0.25	≤ 0.5
1817	*Staphylococcus aureus*	0.5		0.5					1	≤0.06	0.5					0.25		2	≤ 0.5		0.5		0.06	0.25	≤ 0.5
1818	*Staphylococcus aureus*	0.5		0.25					1	≤0.06	0.5					0.25		2	≤ 0.5		0.25		0.12	0.25	≤ 0.5
1819	*Staphylococcus aureus*	0.5		0.25					1	≤0.06	0.25					0.25		2	≤ 0.5		0.5		0.06	0.25	≤ 0.5
1820	*Staphylococcus aureus*	0.5		0.5					1	0.12	0.5					0.5		2	≤ 0.5		0.25		0.06	0.5	≤ 0.5
1821	*Staphylococcus aureus*	0.5		0.5					1	≤0.06	0.5					0.5		2	≤ 0.5		0.25		0.06	0.25	≤ 0.5
1822	*Staphylococcus aureus*	0.5		0.25					1	≤0.06	0.25					0.25		2	≤ 0.5		0.5		0.25	0.25	≤ 0.5
1823	*Staphylococcus aureus*	1		0.25					1	0.12	0.5					0.25		2	> 4		> 16		0.12	1	≤ 0.5
1824	*Staphylococcus aureus*	0.5		2					1	≤0.06	0.5					0.25		2	≤ 0.5		0.25		0.06	0.5	≤ 0.5
1825	*Staphylococcus aureus*	0.5		1					1	≤0.06	0.5					0.12		2	≤ 0.5		0.5		0.12	1	≤ 0.5
1826	*Staphylococcus aureus*	0.5		0.5					1	≤0.06	0.25					0.25		2	≤ 0.5		0.25		0.25	0.25	≤ 0.5
1827	*Staphylococcus aureus*	0.5		0.5					1	≤0.06	0.5					0.25		2	≤ 0.5		0.5		0.12	0.5	≤ 0.5
1828	*Staphylococcus aureus*	0.5		0.25					1	≤0.06	0.25					0.25		2	≤ 0.5		0.5		0.12	0.5	≤ 0.5
1829	*Staphylococcus aureus*	0.5		0.25					0.5	≤0.06	0.25					0.12		2	≤ 0.5		0.25		0.25	0.5	≤ 0.5
1830	*Staphylococcus aureus*	0.5		0.25					1	≤0.06	0.5					0.5		2	≤ 0.5		0.25		0.06	0.5	≤ 0.5
1831	*Staphylococcus aureus*	0.5		1					1	≤0.06	0.25					> 8		4	≤ 0.5		0.5		0.25	0.5	≤ 0.5
1832	*Staphylococcus aureus*	0.5		0.25					1	≤0.06	0.25					0.5		2	≤ 0.5		0.25		0.12	0.5	≤ 0.5
1833	*Staphylococcus aureus*	0.5		0.5					1	≤0.06	0.5					0.5		4	≤ 0.5		> 16		0.5	0.25	≤ 0.5

Isolate Number	*Staphylococcus* species	MIC (µg/ml) Values																							
			β Lactams					Carbapenem	Glycopeptides			Cephalosporins			Flouroquinolones			Oxazolidnone	Macrolides			Tetracyclines		Aminoglycoside	Antifolate
		XF-73	Penicillin	Oxacillin	Amoxicillin Clavulanate	Piperacillin/Tazobactam	Ampicillin	Meropenem	Vancomycin	Dalbavancin	Teicoplanin	Cefuroxime	Ceftriaxone	Ceftazidime	Levofloxacin	Ciprofloxacin	Moxifloxacin	Linezolid	Clindamycin	Clarithromycin	Erythromycin	Tetracycline	Tigecycline	Gentamicin	Trimethoprim/ Sulfmethoxazole
1834	*Staphylococcus aureus*	0.5		0.5					1	≤0.06	0.5					0.5		2	≤ 0.5		0.5		0.12	0.5	≤ 0.5
1835	*Staphylococcus aureus*	0.5		0.25					1	≤0.06	0.5					> 8		2	≤ 0.5		0.25		0.12	0.5	≤ 0.5
1836	*Staphylococcus aureus*	0.5		0.25					1	≤0.06	0.5					0.5		4	≤ 0.5		> 16		0.12	0.5	≤ 0.5
1837	*Staphylococcus aureus*	1		0.5					1	≤0.06	0.5					0.5		2	≤ 0.5		0.5		0.12	0.5	≤ 0.5
1838	*Staphylococcus aureus*	0.5		0.5					1	≤0.06	1					0.25		2	≤ 0.5		> 16		0.12	0.25	≤ 0.5
1839	*Staphylococcus aureus*	0.5		0.5					1	≤0.06	0.5					0.5		2	≤ 0.5		0.5		0.12	0.5	≤ 0.5
1840	*Staphylococcus aureus*	0.25		0.5					1	≤0.06	0.5					0.5		4	≤ 0.5		0.5		0.25	0.5	≤ 0.5
1841	*Staphylococcus aureus*	0.5		0.25					1	≤0.06	0.5					0.5		2	≤ 0.5		0.5		0.12	> 16	≤ 0.5
1842	*Staphylococcus aureus*	0.5		0.5					2	≤0.06	0.25					0.5		2	≤ 0.5		0.25		0.12	1	≤ 0.5
1843	*Staphylococcus aureus*	0.5		0.5					1	0.12	1					0.5		4	≤ 0.5		0.5		0.12	0.5	≤ 0.5
1844	*Staphylococcus aureus*	0.5		0.5					1	≤0.06	0.5					0.5		4	≤ 0.5		0.5		0.25	0.5	≤ 0.5
1845	*Staphylococcus aureus*	0.5		1					1	0.25	1					> 8		2	≤ 0.5		0.5		0.12	> 16	≤ 0.5
1846	*Staphylococcus aureus*	0.5		0.5					1	≤0.06	0.25					0.5		2	≤ 0.5		0.25		0.06	0.25	≤ 0.5
1847	*Staphylococcus aureus*	0.5		0.5					1	0.12	0.5					0.5		4	≤ 0.5		> 16		0.25	0.5	≤ 0.5
1848	*Staphylococcus aureus*	0.5		1					1	≤0.06	0.5					0.25		4	≤ 0.5		0.5		0.25	0.5	≤ 0.5
1849	*Staphylococcus aureus*	0.5		0.5					1	≤0.06	0.5					0.5		2	> 4		> 16		0.06	0.25	≤ 0.5
1850	*Staphylococcus aureus*	1		0.5					0.5	≤0.06	0.25					0.5		2	≤ 0.5		0.5		0.12	0.25	≤ 0.5
1851	*Staphylococcus aureus*	0.5		0.5					1	0.12	0.5					0.25		1	> 4		> 16		0.06	0.25	≤ 0.5
1852	*Staphylococcus aureus*	0.5		1					1	≤0.06	0.5					0.5		2	≤ 0.5		0.5		0.12	> 16	≤ 0.5
1853	*Staphylococcus aureus*	0.5		1					1	≤0.06	0.25					0.5		4	≤ 0.5		> 16		0.25	0.25	≤ 0.5
1854	*Staphylococcus aureus*	0.5		0.5					1	≤0.06	0.5					0.25		2	≤ 0.5		0.5		0.12	0.5	≤ 0.5
1855	*Staphylococcus aureus*	0.5		1					1	0.12	1					0.5		2	≤ 0.5		0.25		0.12	0.25	≤ 0.5
1856	*Staphylococcus aureus*	0.5		0.25					0.5	0.12	0.5					0.25		2	≤ 0.5		0.5		0.12	0.25	≤ 0.5
1857	*Staphylococcus aureus*	0.5		0.25					1	0.12	0.25					0.25		2	≤ 0.5		0.5		0.12	0.5	≤ 0.5
1858	*Staphylococcus aureus*	0.5		0.5					1	≤0.06	0.5					0.25		4	≤ 0.5		0.5		0.12	0.25	≤ 0.5
1859	*Staphylococcus aureus*	0.5		0.5					1	0.12	0.5					0.5		2	≤ 0.5		0.5		0.12	0.25	≤ 0.5
1860	*Staphylococcus aureus*	0.5		0.5					1	≤0.06	0.25					0.5		2	≤ 0.5		0.25		0.06	0.5	≤ 0.5
1861	*Staphylococcus aureus*	0.5		0.5					1	≤0.06	0.5					0.5		4	≤ 0.5		0.5		0.12	0.5	≤ 0.5
1862	*Staphylococcus aureus*	1		0.25					0.5	≤0.06	0.25					0.5		2	≤ 0.5		0.5		0.06	0.5	≤ 0.5
1863	*Staphylococcus aureus*	0.5		0.5					1	≤0.06	0.5					0.5		4	≤ 0.5		0.5		0.12	1	≤ 0.5
1864	*Staphylococcus aureus*	0.25		0.5					1	≤0.06	0.5					0.25		2	≤ 0.5		0.5		0.12	0.25	≤ 0.5
1865	*Staphylococcus aureus*	0.5		0.5					1	≤0.06	1					0.25		2	≤ 0.5		0.25		0.12	0.25	≤ 0.5
1866	*Staphylococcus aureus*	0.5		0.5					1	≤0.06	0.25					0.12		2	≤ 0.5		0.25		0.06	0.25	≤ 0.5
1867	*Staphylococcus aureus*	0.5		0.25					0.5	≤0.06	0.25					0.25		4	≤ 0.5		0.5		0.12	0.5	≤ 0.5
1868	*Staphylococcus aureus*	0.25		0.5					0.5	0.12	0.25					0.5		4	≤ 0.5		0.5		0.06	1	≤ 0.5
1869	*Staphylococcus aureus*	0.5		0.25					1	≤0.06	0.5					0.5		2	≤ 0.5		0.5		0.12	0.5	≤ 0.5
1870	*Staphylococcus aureus*	0.5		0.5					1	≤0.06	0.5					0.25		2	≤ 0.5		0.25		0.06	1	≤ 0.5
1871	*Staphylococcus aureus*	0.5		0.5					0.5	≤0.06	0.5					0.5		4	> 4		> 16		0.25	0.5	≤ 0.5
1872	*Staphylococcus aureus*	0.5		0.5					1	≤0.06	0.25					0.25		2	≤ 0.5		> 16		0.06	0.5	≤ 0.5
1873	*Staphylococcus aureus*	0.5		0.5					1	≤0.06	0.25					0.5		4	≤ 0.5		16		0.12	0.5	≤ 0.5
1874	*Staphylococcus aureus*	0.5		0.5					1	≤0.06	0.25					> 8		2	≤ 0.5		0.25		0.06	0.5	≤ 0.5
1875	*Staphylococcus aureus*	0.5		0.5					1	≤0.06	0.25					0.5		2	≤ 0.5		0.25		0.12	2	≤ 0.5
1876	*Staphylococcus aureus*	0.5		0.5					1	≤0.06	0.5					0.5		2	≤ 0.5		4		0.06	0.5	≤ 0.5
1877	*Staphylococcus aureus*	0.5		0.5					1	≤0.06	0.5					0.5		2	≤ 0.5		0.25		0.06	0.5	≤ 0.5
1878	*Staphylococcus aureus*	4		0.5					1	≤0.06	0.5					0.12		4	1		0.5		0.25	0.5	≤ 0.5
1879	*Staphylococcus aureus*	0.5		0.25					1	0.12	0.5					0.25		2	≤ 0.5		> 16		0.12	0.25	≤ 0.5
1880	*Staphylococcus aureus*	0.5		0.5					1	≤0.06	0.5					0.5		4	≤ 0.5		> 16		0.12	0.5	≤ 0.5

Table s2: MIC Data for the Staphylococcus species tested

Isolate Number	*Staphylococcus* species	MIC (µg/ml) Values																							
			β Lactams					Carbapenem	Glycopeptides			Cephalosporins			Flouroquinolones			Oxazolidnone	Macrolides			Tetracyclines		Aminoglycoside	Antifolate
		XF-73	Penicillin	Oxacillin	Amoxicillin Clavulanate	Piperacillin/Tazobactam	Ampicillin	Meropenem	Vancomycin	Dalbavancin	Teicoplanin	Cefuroxime	Ceftriaxone	Ceftazidime	Levofloxacin	Ciprofloxacin	Moxifloxacin	Linezolid	Clindamycin	Clarithromycin	Erythromycin	Tetracycline	Tigecycline	Gentamicin	Trimethoprim/ Sulfmethoxazole
1881	*Staphylococcus aureus*	0.5		0.5					1	≤0.06	1					0.5		2	> 4		> 16		0.12	0.5	≤ 0.5
1882	*Staphylococcus aureus*	0.5		0.25					1	≤0.06	0.25					0.5		2	≤ 0.5		0.5		0.12	0.5	1
1883	*Staphylococcus aureus*	0.5		0.5					1	≤0.06	0.5					0.5		4	≤ 0.5		0.5		0.12	0.5	≤ 0.5
1884	*Staphylococcus aureus*	0.5		0.25					1	≤0.06	0.5					0.25		1	≤ 0.5		0.25		0.06	1	≤ 0.5
1885	*Staphylococcus aureus*	0.5		0.5					1	≤0.06	0.5					0.25		4	≤ 0.5		0.5		0.25	0.5	≤ 0.5
1886	*Staphylococcus aureus*	0.5		0.5					1	≤0.06	0.5					0.5		2	≤ 0.5		0.5		0.12	0.5	≤ 0.5
1887	*Staphylococcus aureus*	0.5		0.5					1	≤0.06	0.5					0.5		2	≤ 0.5		0.25		0.12	0.25	≤ 0.5
1888	*Staphylococcus aureus*	0.5		0.25					1	≤0.06	0.5					0.25		2	≤ 0.5		0.25		0.06	0.5	≤ 0.5
1889	*Staphylococcus aureus*	0.5		1					1	≤0.06	0.5					0.25		2	≤ 0.5		0.5		0.06	0.5	≤ 0.5
1890	*Staphylococcus aureus*	0.5		0.25					1	≤0.06	0.25					0.25		4	≤ 0.5		0.5		0.25	0.5	≤ 0.5
1891	*Staphylococcus aureus*	0.5		0.25					1	≤0.06	0.25					0.5		4	≤ 0.5		0.5		0.12	0.5	≤ 0.5
1892	*Staphylococcus aureus*	0.5		0.25					1	≤0.06	0.5					0.25		4	≤ 0.5		1		0.12	0.5	≤ 0.5
1893	*Staphylococcus aureus*	0.5		0.25					1	≤0.06	2					0.25		2	≤ 0.5		0.5		0.12	0.25	≤ 0.5
1894	*Staphylococcus aureus*	0.5		0.5					1	≤0.06	0.5					0.5		4	≤ 0.5		0.5		0.25	0.5	≤ 0.5
1895	*Staphylococcus aureus*	0.5		1					1	≤0.06	0.5					2		4	≤ 0.5		0.5		0.25	1	≤ 0.5
1896	*Staphylococcus aureus*	0.5		0.5					0.5	≤0.06	0.25					> 8		2	≤ 0.5		> 16		0.06	0.25	≤ 0.5
1897	*Staphylococcus aureus*	1		0.5					1	≤0.06	0.5					0.5		2	≤ 0.5		0.5		0.12	0.5	≤ 0.5
1898	*Staphylococcus aureus*	0.5		0.25					1	≤0.06	0.25					0.12		2	≤ 0.5		0.5		0.06	0.5	≤ 0.5
1899	*Staphylococcus aureus*	1		0.5					1	≤0.06	0.5					0.25		2	≤ 0.5		0.5		0.12	0.5	≤ 0.5
1900	*Staphylococcus aureus*	1		0.5					1	0.12	0.5					> 8		4	≤ 0.5		> 16		0.12	0.5	≤ 0.5
1901	*Staphylococcus aureus*	1		0.5					1	≤0.06	0.5					0.5		2	≤ 0.5		0.25		0.12	0.5	≤ 0.5
1902	*Staphylococcus aureus*	0.5		1					0.5	≤0.06	0.25					> 8		2	≤ 0.5		0.25		0.06	> 16	≤ 0.5
1903	*Staphylococcus aureus*	0.5		0.5					1	≤0.06	0.25					1		2	≤ 0.5		0.5		0.12	0.5	≤ 0.5
1904	*Staphylococcus aureus*	1		0.5					1	≤0.06	0.5					0.5		2	1		> 16		0.06	1	≤ 0.5
1905	*Staphylococcus aureus*	1		0.5					1	≤0.06	0.5					0.12		2	≤ 0.5		0.5		0.12	0.25	≤ 0.5
1906	*Staphylococcus aureus*	1		0.5					1	≤0.06	0.5					0.5		2	≤ 0.5		0.25		0.06	0.5	≤ 0.5
1907	*Staphylococcus aureus*	0.5		0.5					1	≤0.06	0.5					0.25		4	≤ 0.5		0.5		0.25	1	≤ 0.5
1908	*Staphylococcus aureus*	0.5		0.5					1	≤0.06	0.25					2		4	≤ 0.5		0.5		0.12	0.5	≤ 0.5
1909	*Staphylococcus aureus*	0.5		1					1	≤0.06	1					0.5		4	≤ 0.5		0.5		0.06	0.25	≤ 0.5
1910	*Staphylococcus aureus*	1		0.5					1	≤0.06	0.5					0.5		2	≤ 0.5		0.5		0.12	1	≤ 0.5
1911	*Staphylococcus aureus*	0.5		0.25					1	≤0.06	0.5					0.25		2	≤ 0.5		0.5		0.06	0.25	≤ 0.5
1912	*Staphylococcus aureus*	1		0.25					1	0.12	0.25					0.25		2	≤ 0.5		0.25		0.12	0.25	≤ 0.5
1913	*Staphylococcus aureus*	1		1					1	≤0.06	0.5					1		2	≤ 0.5		0.5		0.12	0.5	≤ 0.5
1914	*Staphylococcus aureus*	1		0.25					1	0.12	1					0.5		2	≤ 0.5		0.5		0.12	0.5	≤ 0.5
1915	*Staphylococcus aureus*	0.5		0.25					1	≤0.06	0.5					≤ 0.06		2	≤ 0.5		0.5		0.12	0.25	≤ 0.5
1916	*Staphylococcus aureus*	0.5		0.5					1	≤0.06	0.25					0.25		2	≤ 0.5		> 16		0.06	0.5	≤ 0.5
1917	*Staphylococcus aureus*	1		0.5					1	≤0.06	0.25					0.12		2	≤ 0.5		> 16		0.06	0.25	≤ 0.5
1918	*Staphylococcus aureus*	1		0.5					0.5	0.12	0.25					0.25		2	≤ 0.5		0.5		0.12	0.25	≤ 0.5
1919	*Staphylococcus aureus*	1		0.5					2	0.12	1					0.25		2	≤ 0.5		> 16		0.5	1	≤ 0.5
1920	*Staphylococcus capitis*	0.25	0.5		0.06	0.25	0.25	0.03	0.5	0.06	0.25	0.25	1	2	0.12		0.03	1		0.12		0.25	0.12		0.03
1921	*Staphylococcus capitis*	0.5	64		8	64	>32	16	1	0.12	1	>64	>32	>64	8		2	1		>32		0.25	0.25		0.5
1922	*Staphylococcus capitis*	0.25	0.12		0.06	0.12	0.06	0.06	1	≤0.06	0.25	0.12	1	2	0.12		0.06	1		0.12		0.25	0.12		0.06
1923	*Staphylococcus capitis*	0.5	0.12		0.12	0.12	0.12	0.06	0.5	≤0.06	0.25	0.25	2	4	0.12		0.03	1		32		1	0.25		0.06
1924	*Staphylococcus capitis*	1	1		0.12	0.5	0.5	0.12	1	≤0.06	0.5	0.5	2	4	0.25		0.06	1		0.12		32	0.25		0.06
1925	*Staphylococcus capitis*	0.5	≤0.03		0.03	0.12	0.03	0.12	1	0.12	0.25	0.25	1	4	0.25		0.06	1		0.12		0.25	0.12		0.06
1926	*Staphylococcus capitis*	0.5	64		4	8	32	16	2	0.06	8	>64	>32	64	0.12		0.015	1		>32		0.25	0.12		0.06
1927	*Staphylococcus capitis*	0.5	0.03		0.06	0.12	0.06	0.12	1	0.12	0.25	0.25	1	4	0.25		0.12	1		0.12		0.25	0.12		0.06

Table s2: MIC Data for the Staphylococcus species tested

Isolate Number	***Staphylococcus* species**	MIC (µg/ml) Values																							
			**β Lactams**					**Carbapenem**	**Glycopeptides**			**Cephalosporins**			**Flouroquinolones**			**Oxazolidnone**	**Macrolides**			**Tetracyclines**		**Aminoglycoside**	**Antifolate**
		**XF-73**	**Penicillin**	**Oxacillin**	**Amoxicillin Clavulanate**	**Piperacillin/Tazobactam**	**Ampicillin**	**Meropenem**	**Vancomycin**	**Dalbavancin**	**Teicoplanin**	**Cefuroxime**	**Ceftriaxone**	**Ceftazidime**	**Levofloxacin**	**Ciprofloxacin**	**Moxifloxacin**	**Linezolid**	**Clindamycin**	**Clarithromycin**	**Erythromycin**	**Tetracycline**	**Tigecycline**	**Gentamicin**	**Trimethoprim/ Sulfmethoxazole**
1928	*Staphylococcus capitis*	0.5	≤0.03		0.03	0.12	0.03	0.12	1	≤0.06	0.5	0.25	2	4	0.5		0.12	1		0.25		0.5	0.25		0.12
1929	*Staphylococcus capitis*	0.25	4		1	1	2	0.06	1	≤0.06	0.5	1	4	8	0.12		0.03	2		0.25		0.25	0.12		0.06
1930	*Staphylococcus capitis*	0.5	0.25		0.06	0.12	0.12	0.03	1	≤0.06	0.25	0.12	0.5	2	0.25		0.06	1		0.06		0.25	0.06		0.06
1931	*Staphylococcus capitis*	0.5	≤0.03		0.03	0.06	0.03	0.06	1	≤0.06	0.12	0.12	0.5	2	0.25		0.06	1		0.06		0.12	0.12		0.06
1932	*Staphylococcus capitis*	0.5	0.5		0.12	0.5	0.25	0.06	1	≤0.06	0.5	0.25	2	4	0.25		0.06	1		0.12		0.25	0.25		0.06
1933	*Staphylococcus capitis*	0.25	0.03		0.06	0.25	0.03	0.12	1	≤0.06	1	0.25	2	4	0.25		0.12	1		0.12		0.25	0.12		0.06
1934	*Staphylococcus capitis*	0.25	32		4	64	32	8	2	≤0.06	4	>64	>32	64	0.25		0.06	1		0.12		1	0.25		0.06
1935	*Staphylococcus capitis*	0.5	4		4	0.5	4	4	1	≤0.06	0.5	8	16	32	2		0.5	1		>32		0.25	0.12		4
1936	*Staphylococcus capitis*	0.25	0.5		0.25	0.5	0.25	0.25	2	≤0.06	2	0.5	2	8	0.25		0.12	1		>32		0.12	0.06		0.06
1937	*Staphylococcus capitis*	1	0.03		0.06	0.25	0.03	0.12	2	≤0.06	1	0.5	2	8	0.5		0.06	1		0.12		0.25	0.12		0.06
1938	*Staphylococcus capitis*	0.25	>64		8	>64	>32	8	1	≤0.06	1	64	>32	32	4		1	2		>32		0.5	0.25		2
1939	*Staphylococcus capitis*	≤0.12	0.12		0.06	0.12	0.06	0.06	1	≤0.06	0.25	0.25	1	4	0.5		0.12	1		0.12		0.25	0.12		0.06
1940	*Staphylococcus capitis*	0.25	8		0.12	0.5	0.5	0.12	1	≤0.06	0.25	0.5	2	4	0.25		0.12	1		0.12		0.25	0.12		0.03
1941	*Staphylococcus capitis*	0.5	>64		2	32	>32	4	2	≤0.06	0.5	16	32	64	0.25		0.06	1		0.12		1	0.12		1
1942	*Staphylococcus capitis*	0.25	32		4	64	32	16	1	0.12	0.5	>64	>32	>64	0.25		0.06	1		0.12		0.5	0.12		0.06
1943	*Staphylococcus capitis*	0.5	32		4	>64	>32	16	1	≤0.06	0.5	>64	>32	>64	0.25		0.06	0.5		0.12		0.5	0.12		0.06
1944	*Staphylococcus capitis*	0.25	64		8	>64	32	>16	1	0.12	0.5	>64	>32	>64	0.25		0.06	1		0.12		0.5	0.25		0.06
1945	*Staphylococcus capitis*	0.5	>64		2	64	>32	16	1	0.25	0.5	>64	>32	64	4		1	1		>32		0.25	0.12		0.25
1946	*Staphylococcus capitis*	1	>64		1	32	>32	8	1	0.12	0.5	8	32	32	0.25		0.06	1		0.12		0.5	0.25		0.06
1947	*Staphylococcus capitis*	0.25	>64		8	64	>32	8	1	0.12	0.5	>64	>32	>64	2		2	1		>32		0.25	0.12		0.25
1948	*Staphylococcus capitis*	0.5	64		16	>64	>32	>16	1	>0.5	1	>64	>32	>64	>8		4	1		>32		2	0.25		0.06
1949	*Staphylococcus capitis*	0.5	≤0.03		≤0.015	≤0.03	≤0.015	0.03	1	0.25	0.25	0.12	0.5	2	0.25		0.06	1		1		0.25	0.12		0.06
1950	*Staphylococcus capitis*	0.25	4		0.12	0.25	0.5	0.12	1	0.12	0.5	0.25	1	4	0.25		0.06	1		0.12		0.5	0.12		0.03
1951	*Staphylococcus capitis*	0.5	16		0.5	4	4	2	1	0.06	0.5	4	32	32	8		2	0.5		>32		1	0.12		0.06
1952	*Staphylococcus capitis*	0.5	32		0.5	4	16	2	1	≤0.06	0.5	0.5	32	16	0.25		0.06	1		>32		>64	0.5		16
1953	*Staphylococcus capitis*	0.5	8		0.5	2	4	1	1	≤0.06	0.25	0.5	16	16	4		0.5	1		>32		64	0.5		2
1954	*Staphylococcus capitis*	0.5	32		1	4	32	2	1	≤0.06	1	4	16	16	0.25		0.06	1		0.25		0.25	0.25		0.06
1955	*Staphylococcus capitis*	1	64		4	64	32	0.25	1	≤0.06	0.5	16	32	64	2		0.5	1		>32		0.25	0.06		0.03
1956	*Staphylococcus capitis*	≤0.12	64		8	64	32	0.25	1	≤0.06	0.25	>64	>32	64	2		0.5	1		>32		0.25	0.06		0.03
1957	*Staphylococcus capitis*	0.5	2		0.25	0.5	1	0.12	1	≤0.06	0.5	0.5	2	8	0.25		0.06	1		0.12		0.25	0.12		0.03
1958	*Staphylococcus caprae*	0.5	32		2	8	16	8	1	0.12	0.5	0.25	>32	64	0.25		0.06	1		>32		0.25	0.12		0.06
1959	*Staphylococcus caprae*	0.25	32		8	32	16	16	2	0.12	8	>64	>32	>64	0.25		0.06	1		>32		0.5	0.12		0.03
1960	*Staphylococcus caprae*	2	≤0.03		0.03	0.25	0.06	0.25	1	0.5	0.5	0.25	1	4	0.25		0.06	2		0.12		0.25	0.12		0.03
1961	*Staphylococcus caprae*	0.5	32		0.12	0.5	2	0.25	1	≤0.06	0.5	0.5	2	8	0.25		0.12	2		0.25		0.25	0.25		0.03
1962	*Staphylococcus caprae*	0.5	32		0.12	0.25	8	0.12	2	0.12	1	0.25	1	8	0.12		0.06	1		0.12		0.25	0.12		0.5
1963	*Staphylococcus caprae*	0.25	4		0.25	0.5	2	0.12	2	0.25	0.5	0.5	2	8	0.5		0.12	1		0.12		0.25	0.06		0.06
1964	*Staphylococcus caprae*	0.5	8		0.12	0.25	0.5	0.12	0.5	≤0.06	0.25	0.25	1	4	0.25		0.06	1		0.06		0.12	0.06		0.03
1965	*Staphylococcus cohnii*	0.25	0.25		0.12	1	0.12	0.25	1	0.12	1	2	4	16	0.25		0.06	1		0.12		0.25	0.12		0.03
1966	*Staphylococcus condimenti*	0.25	0.12		0.06	0.5	0.06	0.12	1	0.12	0.5	1	4	8	0.12		0.03	1		0.12		0.25	0.12		0.12
1967	*Staphylococcus epidermidis*	0.25	8		1	2	4	4	2	0.12	8	8	32	32	>8		4	1		32		0.25	0.5		0.12
1968	*Staphylococcus epidermidis*	≤0.12	4		0.5	0.5	2	0.5	2	0.12	4	2	8	16	>8		>8	1		0.12		2	0.25		8
1969	*Staphylococcus epidermidis*	≤0.12	4		0.5	1	2	2	2	0.12	2	4	8	16	>8		4	1		>32		2	0.25		16
1970	*Staphylococcus epidermidis*	≤0.12	≤0.03		≤0.015	≤0.03	≤0.015	0.015	1	0.12	1	≤0.03	1	1	0.12		0.06	0.25		>32		1	0.12		0.12
1971	*Staphylococcus epidermidis*	0.5	0.5		0.06	0.12	0.25	0.06	2	0.12	2	0.25	1	4	0.25		0.06	1		0.06		0.25	0.06		0.25
1972	*Staphylococcus epidermidis*	0.5	16		1	1	8	4	2	0.12	1	8	16	16	4		1	1		>32		0.25	0.06		0.25
1973	*Staphylococcus epidermidis*	0.5	0.12		0.03	≤0.03	0.06	0.03	1	0.06	1	0.06	0.5	2	0.25		0.12	0.5		4		32	0.12		0.06
1974	*Staphylococcus epidermidis*	≤0.12	32		8	64	32	>16	2	0.06	2	>64	>32	>64	8		2	0.5		>32		2	0.12		1

Table s2: MIC Data for the Staphylococcus species tested

		MIC (µg/ml) Values																							
			β Lactams					Carbapenem	Glycopeptides			Cephalosporins			Flouroquinolones			Oxazolidnone	Macrolides			Tetracyclines		Aminoglycoside	Antifolate
**Isolate Number**	***Staphylococcus* species**	**XF-73**	**Penicillin**	**Oxacillin**	**Amoxicillin Clavulanate**	**Piperacillin/Tazobactam**	**Ampicillin**	**Meropenem**	**Vancomycin**	**Dalbavancin**	**Teicoplanin**	**Cefuroxime**	**Ceftriaxone**	**Ceftazidime**	**Levofloxacin**	**Ciprofloxacin**	**Moxifloxacin**	**Linezolid**	**Clindamycin**	**Clarithromycin**	**Erythromycin**	**Tetracycline**	**Tigecycline**	**Gentamicin**	**Trimethoprim/ Sulfmethoxazole**
1975	*Staphylococcus epidermidis*	0.25	8		1	2	4	4	1	0.12	4	16	16	32	>8		>8	0.5		16		0.25	0.06		0.12
1976	*Staphylococcus epidermidis*	0.25	4		1	2	4	4	1	0.12	4	8	16	32	>8		>8	0.5		16		0.25	0.06		0.12
1977	*Staphylococcus epidermidis*	0.25	4		0.5	1	4	1	2	0.12	4	4	8	8	0.25		0.06	0.5		0.12		2	0.12		2
1978	*Staphylococcus epidermidis*	0.5	2		0.5	1	2	2	1	0.06	1	4	16	16	4		1	0.5		0.06		0.12	0.06		0.12
1979	*Staphylococcus epidermidis*	0.25	≤0.03		≤0.015	≤0.03	0.03	0.03	0.5	0.12	0.5	0.06	0.25	1	0.12		0.03	0.5		0.12		0.25	0.06		0.06
1980	*Staphylococcus epidermidis*	0.25	8		2	4	8	8	2	0.25	0.5	16	16	32	0.12		0.03	0.5		0.06		1	0.06		0.25
1981	*Staphylococcus epidermidis*	0.25	8		2	4	4	4	2	≤0.06	8	4	16	32	>8		>8	0.5		>32		0.12	0.06		0.06
1982	*Staphylococcus epidermidis*	≤0.12	8		4	4	8	16	2	0.25	4	32	32	64	8		4	1		>32		1	0.12		4
1983	*Staphylococcus epidermidis*	0.5	0.12		0.06	0.25	0.06	0.06	2	0.12	0.5	0.5	1	4	0.25		0.06	1		0.12		0.12	0.03		0.12
1984	*Staphylococcus epidermidis*	1	1		0.12	0.12	0.25	0.06	2	≤0.06	2	0.25	0.5	16	0.25		0.06	1		0.12		0.12	0.06		≤0.015
1985	*Staphylococcus epidermidis*	0.25	8		4	4	8	8	2	≤0.06	4	8	16	32	>8		4	0.5		>32		0.25	0.06		0.12
1986	*Staphylococcus epidermidis*	0.5	4		1	4	2	4	2	≤0.06	4	8	16	32	0.12		0.03	0.5		>32		0.25	0.06		0.12
1987	*Staphylococcus epidermidis*	0.25	0.25		0.06	0.25	0.12	0.06	2	≤0.06	1	0.5	1	8	0.12		0.06	0.5		4		0.12	0.03		0.12
1988	*Staphylococcus epidermidis*	0.5	0.12		0.12	0.12	0.06	0.06	1	0.25	1	0.25	0.5	8	4		1	0.5		>32		0.5	0.12		4
1989	*Staphylococcus epidermidis*	≤0.12	8		8	8	16	16	2	≤0.06	2	>64	>32	64	8		2	0.5		>32		1	0.12		4
1990	*Staphylococcus epidermidis*	0.5	0.5		0.5	0.5	1	1	1	≤0.06	1	2	4	16	8		2	0.5		>32		0.12	0.03		4
1991	*Staphylococcus epidermidis*	≤0.12	0.5		0.25	0.5	0.5	0.25	1	≤0.06	2	0.5	2	8	4		0.5	0.5		8		0.12	0.03		0.06
1992	*Staphylococcus epidermidis*	1	16		2	4	16	8	2	≤0.06	2	8	16	32	>8		>8	1		>32		1	0.12		4
1993	*Staphylococcus epidermidis*	0.5	4		1	0.5	2	1	1	≤0.06	0.5	4	8	32	0.25		0.06	0.5		4		1	0.25		8
1994	*Staphylococcus epidermidis*	0.25	2		1	0.5	2	0.5	1	≤0.06	1	4	16	16	0.12		0.03	1		4		1	0.12		0.12
1995	*Staphylococcus epidermidis*	0.25	2		1	0.5	2	>16	1	≤0.06	1	4	>32	64	0.25		0.06	1		4		1	0.12		16
1996	*Staphylococcus epidermidis*	0.25	1		1	4	1	1	1	≤0.06	1	0.5	8	16	0.25		0.06	0.5		0.06		0.12	0.03		0.12
1997	*Staphylococcus epidermidis*	0.5	16		8	64	8	16	2	≤0.06	2	>64	>32	>64	8		4	0.5		>32		0.25	0.06		8
1998	*Staphylococcus epidermidis*	0.5	2		0.5	0.5	2	1	2	≤0.06	2	4	8	16	0.12		0.03	1		8		0.12	0.06		2
1999	*Staphylococcus epidermidis*	1	0.25		0.12	0.25	0.25	0.06	2	≤0.06	4	0.25	1	8	0.25		0.06	1		8		1	0.12		8
2000	*Staphylococcus epidermidis*	0.5	≤0.03		0.06	0.06	0.03	0.03	2	≤0.06	1	0.25	0.5	4	0.12		0.06	0.5		>32		0.5	0.06		32
2001	*Staphylococcus epidermidis*	0.5	2		1	0.5	4	0.5	2	≤0.06	2	4	8	16	8		2	0.5		8		1	0.12		0.25
2002	*Staphylococcus epidermidis*	0.25	4		1	1	4	2	2	0.25	4	4	8	16	>8		8	2		>32		2	0.5		32
2003	*Staphylococcus epidermidis*	0.25	16		0.5	0.5	8	1	2	0.25	4	2	4	8	0.25		0.06	1		>32		>64	0.5		0.06
2004	*Staphylococcus epidermidis*	≤0.12	32		4	8	32	8	2	≤0.06	8	>64	32	32	8		1	2		0.25		0.5	0.12		0.25
2005	*Staphylococcus epidermidis*	0.5	2		1	0.5	2	2	2	≤0.06	4	4	8	32	8		2	1		>32		1	0.12		2
2006	*Staphylococcus epidermidis*	0.5	4		1	2	4	1	2	≤0.06	4	0.5	16	16	0.12		0.03	1		>32		64	0.5		0.12
2007	*Staphylococcus epidermidis*	0.5	2		0.25	0.25	1	0.12	2	≤0.06	4	0.25	1	4	8		2	1		0.12		1	0.25		4
2008	*Staphylococcus epidermidis*	0.5	4		1	1	4	1	2	≤0.06	4	4	8	16	0.12		0.03	1		8		1	0.25		0.12
2009	*Staphylococcus epidermidis*	0.5	16		2	2	32	4	2	≤0.06	4	32	16	32	4		1	1		32		>64	0.5		4
2010	*Staphylococcus epidermidis*	0.25	4		0.5	0.5	2	0.5	1	≤0.06	1	0.5	2	8	0.25		0.12	1		16		0.25	0.25		32
2011	*Staphylococcus epidermidis*	0.25	1		0.25	0.25	0.25	0.06	2	≤0.06	2	0.25	1	4	0.25		0.06	0.5		0.06		64	0.25		0.25
2012	*Staphylococcus epidermidis*	0.5	4		0.5	0.5	2	0.5	2	≤0.06	2	1	4	8	0.12		0.06	1		2		>64	0.5		4
2013	*Staphylococcus epidermidis*	0.25	16		16	64	16	>16	4	0.06	4	>64	>32	64	8		2	1		>32		1	0.25		8
2014	*Staphylococcus epidermidis*	0.5	4		0.5	0.5	2	2	2	≤0.06	2	4	8	16	0.5		0.12	1		32		64	0.25		0.12
2015	*Staphylococcus epidermidis*	0.25	0.25		0.5	0.5	0.5	0.5	2	≤0.06	2	2	4	16	0.5		0.12	1		>32		64	0.25		16
2016	*Staphylococcus epidermidis*	0.5	0.25		0.12	0.25	0.12	0.06	2	≤0.06	1	0.25	1	8	0.25		0.12	1		0.12		0.25	0.06		0.12
2017	*Staphylococcus epidermidis*	0.5	2		0.12	0.25	0.5	0.06	2	≤0.06	2	0.5	1	8	0.12		0.06	1		0.12		0.25	0.06		0.12
2018	*Staphylococcus epidermidis*	0.25	16		8	32	8	16	4	≤0.06	16	8	>32	64	8		4	1		32		2	0.25		16
2019	*Staphylococcus epidermidis*	0.25	16		16	64	16	>16	4	≤0.06	4	>64	>32	64	8		2	1		>32		2	0.25		8
2020	*Staphylococcus epidermidis*	0.5	2		0.5	1	1	1	2	≤0.06	4	2	4	16	4		1	1		0.12		2	0.25		4
2021	*Staphylococcus epidermidis*	0.5	4		2	2	4	4	2	≤0.06	8	8	16	32	4		1	1		0.12		0.25	0.06		0.12

Isolate Number	*Staphylococcus* species	MIC (µg/ml) Values																							
			β Lactams					Carbapenem	Glycopeptides			Cephalosporins			Flouroquinolones			Oxazolidnone	Macrolides			Tetracyclines		Aminoglycoside	Antifolate
		XF-73	Penicillin	Oxacillin	Amoxicillin Clavulanate	Piperacillin/Tazobactam	Ampicillin	Meropenem	Vancomycin	Dalbavancin	Teicoplanin	Cefuroxime	Ceftriaxone	Ceftazidime	Levofloxacin	Ciprofloxacin	Moxifloxacin	Linezolid	Clindamycin	Clarithromycin	Erythromycin	Tetracycline	Tigecycline	Gentamicin	Trimethoprim/ Sulfmethoxazole
2022	*Staphylococcus epidermidis*	0.5	16		4	2	16	8	2	≤0.06	2	64	16	64	0.12		0.03	1		8		32	0.25		32
2023	*Staphylococcus epidermidis*	0.5	8		0.25	0.25	2	0.06	2	≤0.06	2	0.25	1	8	0.5		0.12	1		0.12		0.25	0.12		0.12
2024	*Staphylococcus epidermidis*	0.5	4		0.5	0.5	4	1	1	≤0.06	1	2	8	32	4		1	1		>32		0.25	0.06		1
2025	*Staphylococcus epidermidis*	0.5	8		1	4	8	2	2	≤0.06	4	4	16	32	0.12		0.06	1		0.12		2	0.25		0.06
2026	*Staphylococcus epidermidis*	0.25	8		2	4	8	4	2	≤0.06	4	8	16	32	0.25		0.06	1		>32		32	0.12		8
2027	*Staphylococcus epidermidis*	0.5	2		1	4	2	4	2	≤0.06	4	8	16	32	4		1	0.5		0.06		0.12	0.06		8
2028	*Staphylococcus epidermidis*	0.5	2		0.5	0.5	1	1	1	≤0.06	1	0.25	4	16	0.12		0.06	0.5		0.12		16	0.06		4
2029	*Staphylococcus epidermidis*	0.5	1		0.5	0.5	1	0.5	2	≤0.06	2	2	4	16	0.25		0.06	1		0.12		32	0.12		0.25
2030	*Staphylococcus epidermidis*	0.5	≤0.03		0.06	0.12	0.03	0.06	2	≤0.06	4	0.25	1	8	0.12		0.03	1		>32		0.25	0.06		0.03
2031	*Staphylococcus epidermidis*	0.5	0.03		0.06	0.12	0.03	0.06	2	0.25	8	0.25	1	8	0.25		0.12	1		0.12		2	0.25		0.25
2032	*Staphylococcus epidermidis*	0.5	0.03		0.06	0.12	0.03	0.06	2	≤0.06	2	0.25	1	8	0.25		0.06	1		0.06		0.12	0.03		0.06
2033	*Staphylococcus epidermidis*	0.5	8		4	4	16	8	2	≤0.06	4	16	32	64	0.25		0.06	1		0.12		2	0.25		0.12
2034	*Staphylococcus epidermidis*	0.5	4		2	4	4	4	2	≤0.06	8	8	16	32	4		1	1		>32		0.25	0.06		0.5
2035	*Staphylococcus epidermidis*	0.5	16		8	32	16	16	2	≤0.06	4	>64	>32	64	>8		>8	1		>32		4	0.25		8
2036	*Staphylococcus epidermidis*	≤0.12	16		8	16	16	16	1	≤0.06	1	64	>32	64	>8		>8	0.5		>32		1	0.12		4
2037	*Staphylococcus epidermidis*	0.25	32		16	64	16	>16	4	0.12	8	>64	>32	>64	4		1	1		>32		2	0.25		16
2038	*Staphylococcus epidermidis*	0.5	8		2	8	8	8	2	≤0.06	4	16	32	64	4		1	1		>32		8	0.12		0.12
2039	*Staphylococcus epidermidis*	0.5	≤0.03		0.03	0.12	0.03	0.06	2	≤0.06	1	0.5	1	8	0.25		0.06	1		0.12		0.25	0.06		0.12
2040	*Staphylococcus epidermidis*	0.5	16		4	4	16	16	2	≤0.06	2	8	32	32	>8		8	0.5		>32		0.25	0.06		8
2041	*Staphylococcus epidermidis*	0.5	8		2	2	4	4	2	≤0.06	4	8	16	32	8		1	1		>32		0.25	0.06		0.5
2042	*Staphylococcus epidermidis*	0.5	0.5		0.12	0.25	0.25	0.06	2	≤0.06	1	0.25	0.5	8	0.25		0.06	1		0.12		1	0.25		0.25
2043	*Staphylococcus epidermidis*	≤0.12	8		4	4	8	16	2	≤0.06	16	32	32	32	>8		2	1		>32		1	0.25		8
2044	*Staphylococcus epidermidis*	0.5	>64		0.25	0.5	>32	0.06	2	≤0.06	4	0.5	2	8	0.25		0.06	4		0.25		1	0.25		0.06
2045	*Staphylococcus epidermidis*	0.25	64		32	>64	>32	>16	4	≤0.06	8	>64	>32	>64	>8		4	2		>32		4	0.5		32
2046	*Staphylococcus epidermidis*	0.25	4		4	4	4	4	4	≤0.06	8	8	16	32	>8		4	1		>32		2	0.25		8
2047	*Staphylococcus epidermidis*	0.5	0.25		0.12	0.25	0.12	0.06	2	≤0.06	4	0.5	1	8	0.25		0.06	1		0.12		2	0.25		0.25
2048	*Staphylococcus epidermidis*	0.5	0.5		0.12	0.25	0.25	0.12	2	≤0.06	4	0.5	1	4	4		1	1		0.12		>64	0.5		16
2049	*Staphylococcus epidermidis*	0.5	0.25		0.12	0.25	0.12	0.06	2	≤0.06	4	0.25	1	8	0.25		0.25	1		0.12		2	0.25		0.25
2050	*Staphylococcus epidermidis*	0.5	≤0.03		0.06	0.12	0.06	0.12	2	≤0.06	2	0.25	1	8	0.25		0.12	2		0.25		0.5	0.12		0.06
2051	*Staphylococcus epidermidis*	0.5	16		8	16	16	>16	4	≤0.06	8	32	>32	64	>8		2	1		>32		2	0.25		16
2052	*Staphylococcus epidermidis*	0.25	32		2	4	16	8	2	≤0.06	8	32	32	64	0.25		0.06	2		>32		0.5	0.12		0.12
2053	*Staphylococcus epidermidis*	0.25	4		1	0.5	2	2	2	≤0.06	4	4	8	32	>8		2	1		0.12		2	0.25		0.25
2054	*Staphylococcus epidermidis*	0.25	8		8	16	8	16	2	≤0.06	4	32	>32	64	>8		8	1		>32		2	0.25		8
2055	*Staphylococcus epidermidis*	0.25	0.25		0.12	0.12	0.25	0.06	2	≤0.06	4	0.25	1	8	0.25		0.06	2		0.25		0.25	0.12		16
2056	*Staphylococcus epidermidis*	0.25	8		4	4	8	16	4	≤0.06	8	16	32	64	>8		4	1		>32		2	0.25		16
2057	*Staphylococcus epidermidis*	1	4		2	4	2	4	2	≤0.06	8	8	16	32	4		1	1		0.12		0.25	0.12		0.06
2058	*Staphylococcus epidermidis*	0.25	0.06		0.06	0.06	0.06	0.06	2	≤0.06	2	0.12	0.5	4	4		1	1		0.12		1	0.12		16
2059	*Staphylococcus epidermidis*	0.12	0.12		0.12	0.25	0.12	0.06	2	0.12	2	0.5	1	8	0.12		0.03	0.5		32		0.12	0.03		0.12
2060	*Staphylococcus epidermidis*	0.12	16		8	32	16	8	2	0.12	2	>64	>32	64	8		2	1		>32		2	0.25		16
2061	*Staphylococcus epidermidis*	0.25	8		4	4	8	16	4	≤0.06	8	32	32	64	8		2	1		>32		1	0.25		16
2062	*Staphylococcus epidermidis*	0.25	0.25		0.12	0.25	0.5	0.06	2	≤0.06	4	0.25	0.5	8	0.5		0.06	1		32		0.25	0.06		4
2063	*Staphylococcus epidermidis*	0.5	8		2	4	4	4	2	0.12	4	8	16	32	8		4	1		0.12		1	0.25		4
2064	*Staphylococcus epidermidis*	0.5	32		1	2	16	0.5	2	≤0.06	4	2	4	16	0.12		0.03	2		32		8	0.12		16
2065	*Staphylococcus epidermidis*	1	16		8	16	16	16	2	0.12	4	64	>32	64	>8		>8	1		>32		2	0.25		16
2066	*Staphylococcus epidermidis*	0.25	16		8	32	16	>16	2	≤0.06	4	>64	>32	64	>8		>8	1		>32		2	0.25		16
2067	*Staphylococcus epidermidis*	0.5	8		2	4	8	8	2	≤0.06	4	16	32	64	>8		2	1		>32		0.25	0.06		8
2068	*Staphylococcus epidermidis*	0.25	0.12		0.06	0.12	0.06	0.03	0.5	≤0.06	2	0.25	0.5	4	0.25		0.06	0.5		32		1	0.12		0.06

Table s2: MIC Data for the Staphylococcus species tested

Isolate Number	*Staphylococcus* species	MIC (µg/ml) Values																							
			β Lactams					Carbapenem	Glycopeptides			Cephalosporins			Flouroquinolones			Oxazolidnone	Macrolides			Tetracyclines		Aminoglycoside	Antifolate
		XF-73	Penicillin	Oxacillin	Amoxicillin Clavulanate	Piperacillin/Tazobactam	Ampicillin	Meropenem	Vancomycin	Dalbavancin	Teicoplanin	Cefuroxime	Ceftriaxone	Ceftazidime	Levofloxacin	Ciprofloxacin	Moxifloxacin	Linezolid	Clindamycin	Clarithromycin	Erythromycin	Tetracycline	Tigecycline	Gentamicin	Trimethoprim/ Sulfmethoxazole
2069	*Staphylococcus epidermidis*	0.25	4		1	0.5	4	4	1	≤0.06	1	0.5	16	16	0.25		0.06	1		>32		32	0.5		0.5
2070	*Staphylococcus epidermidis*	1	8		2	4	4	4	2	≤0.06	4	8	16	32	>8		>8	1		>32		0.25	0.06		0.12
2071	*Staphylococcus epidermidis*	0.25	8		4	4	8	8	2	≤0.06	8	8	32	32	0.25		0.06	0.5		>32		64	0.25		0.06
2072	*Staphylococcus epidermidis*	0.25	2		0.5	1	2	1	1	≤0.06	1	1	4	16	0.12		0.03	0.5		32		1	0.12		0.06
2073	*Staphylococcus epidermidis*	0.25	1		1	1	2	2	1	≤0.06	2	4	16	16	2		0.5	0.5		32		0.5	0.06		0.12
2074	*Staphylococcus epidermidis*	0.25	1		0.25	0.5	0.5	0.12	1	≤0.06	4	1	2	8	8		1	0.5		16		1	0.12		4
2075	*Staphylococcus epidermidis*	0.25	1		0.5	0.5	1	0.5	1	≤0.06	0.5	1	2	8	2		0.5	0.5		0.06		1	0.12		0.12
2076	*Staphylococcus epidermidis*	0.25	0.25		0.12	0.12	0.12	0.06	2	≤0.06	4	0.12	0.5	4	0.25		0.06	1		4		1	0.12		0.25
2077	*Staphylococcus epidermidis*	0.5	16		8	16	8	16	2	≤0.06	4	64	>32	64	>8		>8	1		0.06		2	0.25		8
2078	*Staphylococcus epidermidis*	0.25	16		16	32	16	16	2	0.12	2	>64	>32	64	8		2	32		0.25		2	0.25		8
2079	*Staphylococcus epidermidis*	0.25	0.03		0.06	0.12	0.06	0.12	1	≤0.06	2	0.25	1	4	0.25		0.06	1		0.06		0.25	0.06		0.03
2080	*Staphylococcus epidermidis*	0.5	4		2	4	4	4	2	≤0.06	4	16	16	32	4		1	2		>32		16	0.12		0.5
2081	*Staphylococcus epidermidis*	0.25	8		4	4	8	16	2	≤0.06	4	32	32	32	4		1	1		>32		0.12	0.06		0.12
2082	*Staphylococcus epidermidis*	0.5	≤0.03		≤0.015	≤0.03	≤0.015	0.03	2	≤0.06	0.12	0.25	0.25	2	0.25		0.12	2		0.25		0.25	0.015		1
2083	*Staphylococcus epidermidis*	0.25	32		8	64	16	16	1	≤0.06	0.5	>64	>32	>64	8		2	0.5		>32		0.25	0.03		16
2084	*Staphylococcus epidermidis*	0.25	16		8	64	16	16	2	≤0.06	0.5	32	>32	64	8		2	1		>32		0.12	0.03		16
2085	*Staphylococcus epidermidis*	0.25	8		2	4	8	8	2	≤0.06	8	16	32	32	4		1	1		>32		0.12	0.06		0.12
2086	*Staphylococcus epidermidis*	0.5	32		4	2	32	8	2	0.12	2	16	16	32	0.12		0.06	1		>32		0.12	0.06		1
2087	*Staphylococcus epidermidis*	0.5	4		0.12	0.5	1	0.12	2	0.12	4	0.5	2	8	1		0.25	1		0.12		0.25	0.06		0.12
2088	*Staphylococcus epidermidis*	0.25	8		4	4	8	8	2	0.12	4	16	32	32	0.25		0.06	1		0.12		16	0.06		0.06
2089	*Staphylococcus epidermidis*	1	32		2	1	32	2	2	≤0.06	1	8	16	32	0.06		0.015	1		0.12		0.25	0.06		0.06
2090	*Staphylococcus epidermidis*	0.5	32		2	8	32	4	4	0.25	16	8	32	64	>8		>8	1		>32		0.25	0.12		0.12
2091	*Staphylococcus epidermidis*	0.5	4		4	4	4	8	2	≤0.06	4	16	16	32	>8		>8	1		>32		2	0.25		0.12
2092	*Staphylococcus epidermidis*	0.5	16		8	16	32	16	2	≤0.06	4	32	32	64	>8		>8	0.5		>32		1	0.12		0.25
2093	*Staphylococcus epidermidis*	0.5	0.03		0.12	0.5	0.06	0.12	0.5	0.12	2	1	4	8	0.12		0.06	1		0.12		0.25	0.12		0.12
2094	*Staphylococcus epidermidis*	0.5	8		8	8	8	16	2	≤0.06	4	16	>32	64	8		2	1		>32		64	0.25		8
2095	*Staphylococcus epidermidis*	0.25	2		1	2	2	4	1	≤0.06	4	8	16	32	4		1	0.5		>32		0.25	0.06		4
2096	*Staphylococcus epidermidis*	0.5	8		8	8	8	16	2	0.25	2	16	>32	64	8		2	0.5		>32		64	0.25		8
2097	*Staphylococcus epidermidis*	0.5	0.5		0.12	0.25	0.25	0.06	2	≤0.06	8	8	8	8	0.25		0.06	1		2		0.12	0.12		0.06
2098	*Staphylococcus epidermidis*	0.5	8		2	2	4	4	2	≤0.06	4	8	16	32	4		1	0.5		>32		0.25	0.12		0.12
2099	*Staphylococcus epidermidis*	0.25	2		1	2	2	0.5	1	≤0.06	2	0.5	8	16	0.12		0.06	0.5		0.06		1	0.12		0.12
2100	*Staphylococcus epidermidis*	0.5	2		0.25	0.25	1	0.06	2	≤0.06	4	0.25	1	4	0.25		0.06	1		0.06		0.25	0.12		8
2101	*Staphylococcus epidermidis*	0.5	0.06		0.06	0.25	0.06	0.12	2	≤0.06	4	0.25	1	8	0.25		0.12	1		0.06		0.25	0.12		0.03
2102	*Staphylococcus epidermidis*	0.5	4		4	4	8	8	2	≤0.06	8	16	>32	64	>8		>8	0.5		>32		0.25	0.12		4
2103	*Staphylococcus epidermidis*	0.5	4		0.5	1	2	1	1	≤0.06	4	8	8	32	0.12		0.06	1		4		64	0.5		0.25
2104	*Staphylococcus epidermidis*	0.5	8		2	2	4	4	2	≤0.06	4	8	16	32	8		2	0.5		>32		0.25	0.12		0.12
2105	*Staphylococcus epidermidis*	0.25	8		1	0.5	4	1	2	≤0.06	8	4	8	16	4		1	1		16		32	1		0.25
2106	*Staphylococcus epidermidis*	0.5	16		16	2	16	16	2	≤0.06	4	16	>32	64	8		2	1		>32		2	0.5		8
2107	*Staphylococcus epidermidis*	0.5	8		1	1	4	1	2	≤0.06	2	4	8	16	0.12		0.06	1		0.06		64	0.5		0.25
2108	*Staphylococcus epidermidis*	0.5	16		8	8	8	16	2	≤0.06	4	8	>32	64	8		2	1		>32		2	0.5		16
2109	*Staphylococcus epidermidis*	0.5	16		4	8	8	8	2	≤0.06	4	32	32	32	4		1	1		>32		0.25	0.06		0.12
2110	*Staphylococcus epidermidis*	0.5	8		8	16	8	16	2	≤0.06	16	32	>32	64	>8		2	1		>32		2	0.5		16
2111	*Staphylococcus epidermidis*	0.5	0.03		0.06	0.12	0.03	0.06	1	≤0.06	1	0.25	1	2	0.12		0.06	0.5		2		1	0.12		0.12
2112	*Staphylococcus epidermidis*	0.25	8		1	1	4	1	1	≤0.06	4	4	8	16	0.12		0.06	1		4		16	0.5		0.12
2113	*Staphylococcus epidermidis*	0.5	0.06		0.06	0.25	0.06	0.12	2	≤0.06	2	0.25	1	4	0.25		0.06	1		32		0.25	0.12		0.06
2114	*Staphylococcus epidermidis*	0.5	16		4	16	16	16	2	≤0.06	2	32	32	64	4		0.5	1		>32		2	0.25		8
2115	*Staphylococcus epidermidis*	0.5	0.06		0.06	0.12	0.06	0.06	2	≤0.06	2	0.25	1	8	0.25		0.06	1		0.12		0.25	0.12		0.12

Isolate Number	*Staphylococcus* species	MIC (µg/ml) Values																							
			β Lactams					Carbapenem	Glycopeptides			Cephalosporins			Flouroquinolones			Oxazolidnone	Macrolides			Tetracyclines		Aminoglycoside	Antifolate
		XF-73	Penicillin	Oxacillin	Amoxicillin Clavulanate	Piperacillin/Tazobactam	Ampicillin	Meropenem	Vancomycin	Dalbavancin	Teicoplanin	Cefuroxime	Ceftriaxone	Ceftazidime	Levofloxacin	Ciprofloxacin	Moxifloxacin	Linezolid	Clindamycin	Clarithromycin	Erythromycin	Tetracycline	Tigecycline	Gentamicin	Trimethoprim/ Sulfmethoxazole
2116	*Staphylococcus epidermidis*	0.5	8		2	2	4	8	2	≤0.06	4	8	32	32	4		1	0.5		>32		0.25	0.06		0.12
2117	*Staphylococcus epidermidis*	0.5	2		1	1	2	2	2	0.12	4	4	16	16	4		1	0.5		>32		0.12	0.06		4
2118	*Staphylococcus epidermidis*	0.25	16		1	1	16	2	1	≤0.06	4	4	8	32	0.5		0.06	1		>32		32	0.06		0.06
2119	*Staphylococcus epidermidis*	0.5	2		0.5	0.5	2	1	2	≤0.06	4	2	4	16	0.12		0.06	0.5		>32		1	0.12		0.12
2120	*Staphylococcus epidermidis*	0.5	≤0.03		0.06	0.12	0.03	0.06	1	≤0.06	4	0.25	1	4	0.25		0.12	1		0.12		0.25	0.12		0.25
2121	*Staphylococcus epidermidis*	0.25	32		8	32	32	>16	2	≤0.06	4	>64	>32	64	4		1	1		>32		2	0.25		8
2122	*Staphylococcus epidermidis*	0.25	16		8	16	16	16	2	≤0.06	2	>64	>32	64	>8		>8	1		0.12		2	0.25		8
2123	*Staphylococcus epidermidis*	0.5	4		2	4	4	4	2	≤0.06	2	0.5	16	16	>8		>8	1		0.12		2	0.5		8
2124	*Staphylococcus epidermidis*	0.12	16		16	64	16	>16	2	0.12	4	>64	>32	>64	4		1	1		>32		2	0.25		8
2125	*Staphylococcus epidermidis*	0.5	16		1	1	4	1	2	0.12	2	4	8	16	0.12		0.06	0.5		0.12		1	0.25		0.12
2126	*Staphylococcus epidermidis*	0.5	2		0.5	0.5	2	0.5	1	≤0.06	1	0.25	4	4	0.12		0.06	0.5		0.06		1	0.25		0.25
2127	*Staphylococcus epidermidis*	0.12	0.5		0.25	≤0.03	0.5	0.06	2	≤0.06	4	0.12	0.5	4	0.25		0.06	0.5		0.06		0.12	0.06		0.03
2128	*Staphylococcus epidermidis*	0.25	8		8	2	4	4	2	≤0.06	4	16	32	32	4		0.5	0.5		>32		0.12	0.06		4
2129	*Staphylococcus epidermidis*	0.5	8		4	4	8	16	2	≤0.06	8	32	32	64	4		2	1		>32		8	0.25		0.25
2130	*Staphylococcus epidermidis*	0.5	4		2	2	4	2	1	≤0.06	4	8	16	32	4		1	0.5		>32		0.12	0.06		0.12
2131	*Staphylococcus epidermidis*	1	4		1	2	4	2	2	≤0.06	4	4	8	32	0.12		0.06	0.5		0.06		1	0.12		0.12
2132	*Staphylococcus epidermidis*	0.5	0.12		0.06	0.12	0.12	0.06	2	≤0.06	2	0.12	0.5	4	0.25		0.06	0.5		0.06		0.12	0.06		0.12
2133	*Staphylococcus epidermidis*	0.5	2		0.5	0.25	2	2	1	≤0.06	2	1	4	8	0.25		0.06	1		0.06		>64	0.5		0.25
2134	*Staphylococcus epidermidis*	0.25	2		0.5	0.5	1	1	2	≤0.06	1	4	4	16	0.25		0.12	0.5		0.06		0.12	0.03		0.12
2135	*Staphylococcus epidermidis*	0.5	2		1	1	2	2	1	≤0.06	4	8	8	16	4		1	0.5		>32		8	0.06		0.12
2136	*Staphylococcus epidermidis*	0.25	4		2	2	4	4	2	≤0.06	8	8	16	32	4		1	0.5		0.06		0.25	0.12		8
2137	*Staphylococcus epidermidis*	0.25	0.03		0.06	0.12	0.03	0.06	2	≤0.06	2	0.25	1	4	0.25		0.06	1		0.25		1	0.12		0.12
2138	*Staphylococcus epidermidis*	0.5	8		2	2	4	4	2	≤0.06	4	8	16	32	2		0.5	1		0.06		0.12	0.06		4
2139	*Staphylococcus epidermidis*	0.25	16		16	64	16	16	2	≤0.06	4	>64	>32	64	8		2	1		0.12		2	0.25		8
2140	*Staphylococcus epidermidis*	0.5	0.5		0.12	0.12	0.25	0.06	2	≤0.06	4	0.25	0.5	4	0.25		0.06	1		>32		2	0.25		0.25
2141	*Staphylococcus epidermidis*	0.5	1		0.5	0.5	1	0.5	1	≤0.06	4	1	4	16	0.12		0.06	1		32		1	0.25		0.12
2142	*Staphylococcus epidermidis*	0.25	0.03		0.06	0.25	0.06	0.06	1	≤0.06	4	0.25	1	8	0.25		0.06	0.5		0.12		0.25	0.12		0.03
2143	*Staphylococcus epidermidis*	0.25	8		4	4	16	8	2	≤0.06	4	16	16	32	4		1	1		0.06		0.25	0.06		0.12
2144	*Staphylococcus epidermidis*	≤0.12	4		2	2	2	2	2	≤0.06	8	8	16	32	4		1	0.5		>32		0.12	0.06		0.06
2145	*Staphylococcus epidermidis*	0.25	32		32	64	16	>16	2	≤0.06	2	>64	>32	64	>8		8	1		>32		1	0.25		8
2146	*Staphylococcus epidermidis*	0.25	0.5		0.25	0.25	1	0.12	1	≤0.06	1	0.25	2	4	0.12		0.06	0.5		≤0.03		1	0.25		0.12
2147	*Staphylococcus epidermidis*	0.5	0.25		0.12	0.12	0.12	0.06	2	≤0.06	2	0.25	0.5	4	4		1	1		>32		2	0.25		8
2148	*Staphylococcus epidermidis*	0.25	1		0.5	0.5	1	0.5	1	≤0.06	1	2	4	8	0.12		0.06	0.5		4		1	0.25		0.12
2149	*Staphylococcus epidermidis*	0.25	0.25		0.12	0.25	0.12	0.06	2	≤0.06	2	0.25	1	4	0.12		0.06	1		16		0.25	0.12		0.12
2150	*Staphylococcus epidermidis*	0.25	0.06		0.06	0.12	0.06	0.06	2	≤0.06	2	0.25	0.5	4	0.25		0.06	1		0.12		32	0.12		0.12
2151	*Staphylococcus epidermidis*	0.25	8		2	4	8	4	2	≤0.06	4	16	16	32	0.25		0.06	0.5		>32		2	0.25		0.12
2152	*Staphylococcus epidermidis*	≤0.12	0.25		0.12	0.25	0.12	0.06	1	≤0.06	4	0.25	1	4	0.25		0.06	1		4		2	0.25		0.12
2153	*Staphylococcus epidermidis*	0.25	0.12		0.12	0.25	0.12	0.06	2	≤0.06	2	0.5	1	4	0.25		0.06	0.5		0.06		0.5	0.12		0.12
2154	*Staphylococcus epidermidis*	0.25	0.5		0.25	0.25	0.5	0.12	2	≤0.06	8	0.5	1	8	0.5		0.12	1		>32		0.5	0.12		0.06
2155	*Staphylococcus epidermidis*	0.25	16		4	4	8	8	2	≤0.06	4	32	32	64	>8		>8	1		>32		2	0.5		16
2156	*Staphylococcus epidermidis*	0.5	2		0.25	0.25	1	0.12	4	≤0.06	4	0.5	2	4	0.25		0.06	1		>32		0.25	0.06		0.25
2157	*Staphylococcus epidermidis*	1	64		32	>64	32	>16	2	0.12	2	>64	>32	>64	4		1	1		>32		4	0.25		16
2158	*Staphylococcus epidermidis*	0.5	4		0.25	0.25	2	0.06	2	≤0.06	2	0.5	2	4	0.25		0.06	1		0.25		0.5	0.12		1
2159	*Staphylococcus epidermidis*	0.5	16		2	4	8	8	2	≤0.06	8	32	32	32	8		1	1		0.12		0.5	0.12		0.5
2160	*Staphylococcus epidermidis*	0.25	8		2	2	8	4	2	≤0.06	4	8	16	32	>8		4	2		>32		4	1		16
2161	*Staphylococcus epidermidis*	0.25	16		4	8	16	16	2	0.12	2	32	32	32	4		0.5	1		>32		2	0.25		16
2162	*Staphylococcus epidermidis*	0.5	1		0.12	0.25	0.5	0.06	2	≤0.06	2	0.25	1	4	0.25		0.06	1		0.12		2	0.25		0.25
2163	*Staphylococcus epidermidis*	0.25	32		2	4	16	8	2	≤0.06	8	32	32	64	>8		4	1		16		2	0.25		16

Isolate Number	*Staphylococcus* species	MIC (µg/ml) Values																							
			β Lactams					Carbapenem	Glycopeptides			Cephalosporins			Flouroquinolones			Oxazolidnone	Macrolides			Tetracyclines		Aminoglycoside	Antifolate
		XF-73	Penicillin	Oxacillin	Amoxicillin Clavulanate	Piperacillin/Tazobactam	Ampicillin	Meropenem	Vancomycin	Dalbavancin	Teicoplanin	Cefuroxime	Ceftriaxone	Ceftazidime	Levofloxacin	Ciprofloxacin	Moxifloxacin	Linezolid	Clindamycin	Clarithromycin	Erythromycin	Tetracycline	Tigecycline	Gentamicin	Trimethoprim/ Sulfmethoxazole
2164	*Staphylococcus epidermidis*	≤0.12	4		2	2	2	2	2	0.12	8	8	16	32	4		1	1		0.12		0.25	0.12		0.12
2165	*Staphylococcus epidermidis*	0.5	8		2	2	4	4	2	0.12	2	8	16	32	8		2	1		>32		1	0.12		0.25
2166	*Staphylococcus epidermidis*	0.25	16		0.5	0.5	8	1	1	≤0.06	0.5	4	8	16	0.25		0.06	0.5		>32		1	0.12		0.25
2167	*Staphylococcus epidermidis*	0.25	16		1	1	16	1	2	≤0.06	4	4	8	16	0.25		0.06	1		0.06		2	0.5		0.25
2168	*Staphylococcus epidermidis*	0.25	0.5		0.12	0.25	0.25	0.12	2	≤0.06	4	0.25	1	4	0.12		0.03	1		0.06		0.25	0.06		0.12
2169	*Staphylococcus epidermidis*	0.5	32		16	>64	32	>16	4	0.25	16	>64	>32	>64	4		1	2		>32		4	0.5		8
2170	*Staphylococcus epidermidis*	0.25	32		8	64	16	16	2	≤0.06	4	64	>32	64	8		4	1		>32		0.25	0.12		0.12
2171	*Staphylococcus epidermidis*	0.25	16		16	64	16	16	4	≤0.06	2	>64	>32	>64	8		4	1		>32		0.25	0.06		4
2172	*Staphylococcus epidermidis*	0.25	4		0.12	0.25	0.5	0.25	1	≤0.06	0.25	0.5	1	8	0.25		0.12	0.5		0.06		0.25	0.06		0.12
2173	*Staphylococcus epidermidis*	0.5	32		32	>64	32	>16	2	≤0.06	8	>64	>32	>64	4		1	2		>32		4	0.5		4
2174	*Staphylococcus epidermidis*	0.25	0.5		0.12	0.12	0.25	0.06	1	0.12	1	0.12	0.5	4	0.25		0.06	1		0.12		0.5	0.12		0.25
2175	*Staphylococcus epidermidis*	0.25	2		1	2	2	1	2	≤0.06	4	4	8	16	0.25		0.06	1		>32		64	0.5		0.12
2176	*Staphylococcus epidermidis*	0.25	0.25		0.06	0.12	0.12	0.03	2	≤0.06	4	0.25	1	4	0.12		0.06	1		16		0.25	0.06		0.12
2177	*Staphylococcus epidermidis*	≤0.12	32		16	64	32	>16	2	0.12	8	>64	>32	>64	4		1	2		>32		4	0.5		8
2178	*Staphylococcus epidermidis*	0.25	16		16	64	16	16	2	≤0.06	4	64	>32	64	8		2	0.5		>32		0.25	0.06		8
2179	*Staphylococcus epidermidis*	≤0.12	32		16	>64	32	>16	4	0.25	16	>64	>32	>64	4		1	2		>32		4	0.5		8
2180	*Staphylococcus epidermidis*	0.25	0.12		0.12	0.25	0.12	0.06	1	≤0.06	2	0.25	1	4	0.12		0.06	0.5		0.06		2	0.25		0.12
2181	*Staphylococcus epidermidis*	0.25	8		2	2	8	2	2	≤0.06	4	8	16	32	0.25		0.06	0.5		0.06		2	0.25		0.12
2182	*Staphylococcus epidermidis*	0.25	8		2	2	8	2	2	≤0.06	4	8	16	32	0.25		0.06	0.5		0.06		1	0.12		0.06
2183	*Staphylococcus epidermidis*	0.5	1		1	1	2	1	2	≤0.06	4	2	8	16	>8		2	1		>32		1	0.25		8
2184	*Staphylococcus epidermidis*	1	16		16	32	16	>16	2	0.25	8	>64	>32	>64	2		0.5	0.5		>32		1	0.25		4
2185	*Staphylococcus epidermidis*	0.5	1		1	1	2	2	2	0.12	2	0.5	8	16	8		1	1		0.12		0.25	0.12		2
2186	*Staphylococcus epidermidis*	0.5	4		1	1	4	2	2	≤0.06	4	8	8	32	>8		4	1		>32		2	0.5		8
2187	*Staphylococcus epidermidis*	0.5	16		8	32	8	16	2	0.25	4	32	>32	64	8		2	0.5		>32		0.25	0.06		0.5
2188	*Staphylococcus epidermidis*	0.5	16		8	64	16	16	2	≤0.06	4	64	>32	64	8		2	0.5		>32		0.25	0.06		0.5
2189	*Staphylococcus epidermidis*	0.25	32		4	8	32	8	2	0.12	4	64	32	64	8		2	1		0.25		2	0.25		16
2190	*Staphylococcus epidermidis*	0.25	4		1	1	4	2	2	≤0.06	8	4	8	32	>8		2	2		>32		2	0.25		16
2191	*Staphylococcus epidermidis*	0.25	16		2	4	16	4	2	≤0.06	4	8	16	32	8		2	1		>32		2	0.25		16
2192	*Staphylococcus epidermidis*	2	32		1	1	32	2	2	≤0.06	2	4	8	16	0.25		0.06	2		32		0.25	0.06		0.25
2193	*Staphylococcus epidermidis*	0.25	4		1	1	4	1	2	≤0.06	4	4	8	16	>8		8	2		>32		4	0.5		32
2194	*Staphylococcus epidermidis*	0.5	32		4	8	32	16	2	≤0.06	8	>64	>32	64	>8		>8	1		>32		2	0.5		32
2195	*Staphylococcus epidermidis*	≤0.12	16		4	4	16	16	2	≤0.06	8	64	32	64	8		2	1		>32		2	0.25		16
2196	*Staphylococcus epidermidis*	0.25	32		8	16	32	16	2	≤0.06	4	>64	>32	64	>8		2	2		>32		4	0.5		32
2197	*Staphylococcus epidermidis*	0.25	64		16	>64	32	16	2	≤0.06	4	>64	>32	>64	>8		4	1		>32		2	0.25		8
2198	*Staphylococcus epidermidis*	0.5	32		4	4	32	8	2	≤0.06	8	32	32	32	8		2	0.5		>32		1	0.12		4
2199	*Staphylococcus epidermidis*	0.25	8		2	1	4	1	1	≤0.06	0.25	1	8	8	4		1	0.25		0.06		0.12	0.12		0.12
2200	*Staphylococcus epidermidis*	0.03	8		1	1	4	4	2	≤0.06	1	8	16	32	4		1	0.5		>32		0.25	0.12		0.12
2201	*Staphylococcus epidermidis*	0.5	≤0.03		0.03	0.12	0.03	0.06	1	≤0.06	1	0.12	0.5	4	0.25		0.06	0.5		0.06		0.12	0.06		0.03
2202	*Staphylococcus epidermidis*	0.5	32		2	4	32	2	2	≤0.06	8	16	32	32	>8		4	2		0.25		2	0.5		8
2203	*Staphylococcus epidermidis*	0.5	≤0.03		0.03	0.12	0.03	0.03	2	0.12	4	0.25	1	4	0.25		0.12	2		16		2	0.5		0.25
2204	*Staphylococcus epidermidis*	0.25	≤0.03		0.03	0.06	0.03	0.06	2	0.12	4	0.25	0.5	4	0.25		0.06	0.5		8		1	0.25		0.25
2205	*Staphylococcus epidermidis*	0.25	4		1	4	4	4	1	≤0.06	2	8	32	64	8		2	0.5		>32		0.5	0.06		8
2206	*Staphylococcus epidermidis*	0.5	1		0.12	0.12	0.5	0.06	2	0.06	4	0.25	1	4	8		1	1		0.12		2	0.25		16
2207	*Staphylococcus epidermidis*	0.5	8		1	2	8	4	1	≤0.06	4	8	16	32	4		1	1		>32		8	0.12		0.12
2208	*Staphylococcus epidermidis*	0.25	4		0.5	1	2	2	2	≤0.06	4	4	8	16	>8		8	2		>32		2	0.5		16
2209	*Staphylococcus epidermidis*	0.5	32		4	64	16	>16	2	≤0.06	8	>64	>32	>64	>8		>8	1		>32		0.25	0.12		16
2210	*Staphylococcus epidermidis*	0.25	≤0.03		≤0.015	≤0.03	0.03	0.03	0.5	≤0.06	1	0.06	0.25	2	0.12		0.03	2		0.12		0.12	0.06		0.03
2211	*Staphylococcus epidermidis*	0.5	8		0.06	0.25	2	0.12	2	≤0.06	8	0.5	2	8	0.5		0.12	1		0.12		0.5	0.12		0.25
2212	*Staphylococcus epidermidis*	0.5	64		8	64	32	>16	1	≤0.06	0.5	>64	>32	>64	8		2	1		0.06		0.12	0.06		0.12
2213	*Staphylococcus epidermidis*	0.5	2		0.12	0.12	1	0.06	2	≤0.06	8	0.25	1	4	0.25		0.06	1		>32		4	0.25		0.5

Isolate Number	*Staphylococcus* species	MIC (µg/ml) Values																							
			β Lactams					Carbapenem	Glycopeptides			Cephalosporins			Flouroquinolones			Oxazolidnone	Macrolides			Tetracyclines		Aminoglycoside	Antifolate
		XF-73	Penicillin	Oxacillin	Amoxicillin Clavulanate	Piperacillin/Tazobactam	Ampicillin	Meropenem	Vancomycin	Dalbavancin	Teicoplanin	Cefuroxime	Ceftriaxone	Ceftazidime	Levofloxacin	Ciprofloxacin	Moxifloxacin	Linezolid	Clindamycin	Clarithromycin	Erythromycin	Tetracycline	Tigecycline	Gentamicin	Trimethoprim/ Sulfmethoxazole
2214	*Staphylococcus epidermidis*	0.5	4		0.25	0.5	2	0.25	2	≤0.06	4	1	4	8	0.25		0.06	1		8		2	0.25		0.25
2215	*Staphylococcus epidermidis*	0.25	0.12		0.06	0.25	0.12	0.12	2	0.12	16	0.5	2	4	0.5		0.12	1		>32		0.5	0.12		0.25
2216	*Staphylococcus epidermidis*	0.5	4		1	1	4	1	2	0.25	8	2	8	16	>8		2	1		>32		2	0.25		16
2217	*Staphylococcus epidermidis*	0.5	32		8	64	16	16	2	≤0.06	8	>64	>32	>64	>8		>8	1		>32		0.25	0.06		8
2218	*Staphylococcus epidermidis*	0.25	8		1	2	4	4	2	0.12	16	2	16	32	>8		>8	1		>32		0.5	0.12		32
2219	*Staphylococcus epidermidis*	0.5	2		0.25	0.25	1	0.12	2	≤0.06	4	0.5	1	4	0.25		0.06	1		>32		0.25	0.06		0.25
2220	*Staphylococcus epidermidis*	0.25	16		8	32	16	16	2	≤0.06	4	>64	>32	>64	>8		2	1		>32		2	0.25		16
2221	*Staphylococcus epidermidis*	0.5	>64		32	>64	>32	>16	1	0.25	2	>64	>32	>64	>8		8	1		>32		2	0.5		>32
2222	*Staphylococcus epidermidis*	0.5	8		2	4	8	4	2	≤0.06	2	16	16	32	0.25		0.06	2		>32		2	0.25		0.12
2223	*Staphylococcus epidermidis*	0.5	8		1	1	4	1	2	≤0.06	4	4	4	16	8		1	1		>32		64	0.25		16
2224	*Staphylococcus epidermidis*	0.5	8		1	1	8	2	2	≤0.06	2	4	8	16	0.25		0.12	0.5		16		2	0.25		16
2225	*Staphylococcus epidermidis*	0.5	16		1	2	8	2	2	≤0.06	8	4	8	32	>8		8	2		>32		4	0.5		32
2226	*Staphylococcus epidermidis*	0.5	8		1	1	8	2	2	≤0.06	1	8	8	16	0.25		0.12	1		0.12		>64	0.5		0.5
2227	*Staphylococcus epidermidis*	≤0.12	16		0.5	1	8	8	2	≤0.06	8	8	16	32	>8		2	1		>32		2	0.25		8
2228	*Staphylococcus epidermidis*	0.25	16		0.5	1	8	2	2	≤0.06	4	4	8	16	0.12		0.03	1		>32		2	0.25		0.25
2229	*Staphylococcus epidermidis*	≤0.12	32		8	>64	32	>16	2	0.12	4	>64	>32	>64	4		1	1		>32		2	0.25		8
2230	*Staphylococcus epidermidis*	0.5	32		16	>64	32	>16	2	0.12	4	>64	>32	>64	4		1	1		0.06		2	0.25		8
2231	*Staphylococcus epidermidis*	0.25	>64		1	32	>32	4	1	≤0.06	2	16	32	32	0.5		0.25	1		0.12		1	0.12		0.5
2232	*Staphylococcus epidermidis*	1	32		2	8	32	16	2	0.5	8	16	32	64	8		2	1		32		2	0.25		8
2233	*Staphylococcus epidermidis*	0.25	8		1	1	8	2	2	≤0.06	8	4	8	32	>8		4	1		32		2	0.5		16
2234	*Staphylococcus epidermidis*	0.25	8		0.5	1	2	1	1	≤0.06	2	4	8	16	4		1	1		>32		1	0.25		0.12
2235	*Staphylococcus epidermidis*	0.25	8		2	4	16	8	2	≤0.06	8	16	>32	32	4		1	1		8		0.25	0.12		0.12
2236	*Staphylococcus epidermidis*	0.25	16		8	64	16	>16	2	≤0.06	2	>64	>32	>64	>8		>8	2		>32		4	0.5		32
2237	*Staphylococcus epidermidis*	0.25	≤0.03		0.03	0.06	0.03	0.06	2	≤0.06	1	0.25	0.5	4	0.25		0.06	1		0.12		0.25	0.12		0.06
2238	*Staphylococcus epidermidis*	0.25	0.12		0.25	0.5	0.5	1	2	0.12	1	2	8	16	4		1	1		>32		2	0.25		0.25
2239	*Staphylococcus epidermidis*	0.25	4		0.12	0.12	1	0.03	1	0.12	1	0.25	0.5	4	0.12		0.06	1		0.12		2	0.12		0.03
2240	*Staphylococcus epidermidis*	0.5	0.25		0.06	0.12	0.25	0.06	2	0.12	2	0.25	1	4	0.25		0.06	1		>32		0.25	0.12		0.25
2241	*Staphylococcus epidermidis*	0.25	8		0.12	0.12	2	0.06	2	0.25	4	0.25	1	4	0.25		0.06	2		>32		0.5	0.12		2
2242	*Staphylococcus epidermidis*	0.5	64		1	2	>32	4	2	≤0.06	16	8	32	32	8		1	1		0.12		0.5	0.12		32
2243	*Staphylococcus epidermidis*	0.25	32		2	4	16	8	1	0.12	8	16	32	32	>8		>8	1		>32		0.25	0.06		32
2244	*Staphylococcus epidermidis*	0.25	4		1	1	4	4	2	≤0.06	8	8	16	32	>8		>8	1		>32		1	0.12		4
2245	*Staphylococcus epidermidis*	0.5	2		0.25	0.5	2	1	2	0.12	8	2	8	16	0.25		0.06	0.5		0.06		1	0.12		0.12
2246	*Staphylococcus epidermidis*	0.25	8		0.5	1	4	2	2	0.25	8	4	16	16	4		1	0.5		>32		2	0.25		0.12
2247	*Staphylococcus epidermidis*	0.5	1		0.12	0.25	0.5	0.25	1	0.12	2	1	8	8	>8		>8	0.5		0.12		1	0.12		0.06
2248	*Staphylococcus epidermidis*	0.25	16		0.5	1	16	0.5	2	0.12	2	4	8	16	>8		4	1		16		0.25	0.06		0.12
2249	*Staphylococcus epidermidis*	0.25	16		4	64	16	16	2	0.12	2	>64	>32	64	4		0.5	1		>32		2	0.25		8
2250	*Staphylococcus epidermidis*	0.5	16		1	1	16	2	4	0.12	4	8	16	32	2		0.5	1		>32		2	0.12		0.25
2251	*Staphylococcus epidermidis*	0.5	2		0.25	0.5	2	1	2	0.12	8	2	8	16	8		4	1		0.12		1	0.25		0.12
2252	*Staphylococcus epidermidis*	0.25	16		4	64	16	16	2	≤0.06	4	32	32	64	8		1	1		>32		2	0.25		16
2253	*Staphylococcus epidermidis*	0.25	16		0.5	0.5	8	2	2	0.5	4	2	8	16	0.25		0.06	1		16		0.25	0.06		0.25
2254	*Staphylococcus epidermidis*	0.25	2		0.5	1	2	2	1	0.12	1	4	8	16	8		2	0.5		>32		1	0.25		4
2255	*Staphylococcus epidermidis*	0.5	16		4	64	16	16	1	0.06	0.5	>64	>32	>64	8		1	1		>32		1	0.25		16
2256	*Staphylococcus epidermidis*	0.5	2		0.25	0.25	2	0.06	2	≤0.06	8	0.5	2	8	0.12		0.03	1		0.12		2	0.25		0.25
2257	*Staphylococcus epidermidis*	0.25	16		4	32	16	16	2	0.06	4	>64	>32	64	>8		8	1		>32		2	0.5		16
2258	*Staphylococcus epidermidis*	0.25	4		0.25	0.5	4	0.5	1	0.25	0.5	0.25	4	8	>8		>8	0.5		0.12		1	0.12		0.12
2259	*Staphylococcus epidermidis*	1	16		4	32	16	>16	2	0.12	8	>64	>32	64	8		2	0.5		>32		0.25	0.12		16
2260	*Staphylococcus epidermidis*	0.5	1		0.5	4	1	1	2	0.12	8	2	8	32	>8		8	1		>32		32	0.12		8
2261	*Staphylococcus epidermidis*	0.5	2		0.5	2	2	1	2	0.12	2	4	8	16	4		1	0.5		>32		32	0.25		4
2262	*Staphylococcus epidermidis*	1	8		0.5	0.5	4	2	2	0.12	2	4	8	16	1		0.25	1		>32		1	0.12		0.25
2263	*Staphylococcus epidermidis*	0.25	8		1	2	8	8	2	0.12	8	8	32	32	4		1	1		>32		64	0.25		0.06

Isolate Number	*Staphylococcus* species	MIC (µg/ml) Values																							
			β Lactams					Carbapenem	Glycopeptides			Cephalosporins			Flouroquinolones			Oxazolidnone	Macrolides			Tetracyclines		Aminoglycoside	Antifolate
		XF-73	Penicillin	Oxacillin	Amoxicillin Clavulanate	Piperacillin/Tazobactam	Ampicillin	Meropenem	Vancomycin	Dalbavancin	Teicoplanin	Cefuroxime	Ceftriaxone	Ceftazidime	Levofloxacin	Ciprofloxacin	Moxifloxacin	Linezolid	Clindamycin	Clarithromycin	Erythromycin	Tetracycline	Tigecycline	Gentamicin	Trimethoprim/ Sulfmethoxazole
2264	*Staphylococcus epidermidis*	2	32		4	32	16	>16	2	≤0.06	4	>64	>32	>64	>8		>8	0.5		>32		0.25	0.06		16
2265	*Staphylococcus epidermidis*	1	4		2	2	4	16	2	0.5	4	64	>32	64	>8		4	1		>32		0.25	0.06		8
2266	*Staphylococcus epidermidis*	0.5	4		0.25	0.5	2	0.5	2	≤0.06	8	1	4	8	0.12		0.06	1		>32		0.5	0.12		0.25
2267	*Staphylococcus epidermidis*	0.5	8		4	16	8	16	2	0.12	4	64	>32	64	>8		4	1		>32		2	0.25		16
2268	*Staphylococcus epidermidis*	0.5	0.06		0.06	0.06	0.12	0.06	2	≤0.06	1	0.12	0.5	4	0.12		0.06	0.5		0.06		0.12	0.06		0.03
2269	*Staphylococcus epidermidis*	1	1		0.25	0.5	1	1	2	≤0.06	1	2	8	8	4		1	1		0.06		>64	0.25		0.25
2270	*Staphylococcus epidermidis*	0.5	2		0.25	0.5	1	1	2	0.12	4	2	8	8	0.12		0.06	1		16		2	0.25		1
2271	*Staphylococcus epidermidis*	0.25	8		4	32	8	16	2	≤0.06	8	64	>32	64	>8		2	0.5		>32		2	0.25		8
2272	*Staphylococcus epidermidis*	0.25	16		1	2	16	4	2	≤0.06	16	16	32	32	4		1	1		>32		0.25	0.12		32
2273	*Staphylococcus epidermidis*	1	0.5		0.25	0.5	0.5	0.5	1	≤0.06	0.5	4	4	16	4		1	0.5		>32		1	0.12		16
2274	*Staphylococcus epidermidis*	0.25	32		1	4	16	4	2	≤0.06	16	16	32	32	4		1	0.5		>32		0.12	0.12		32
2275	*Staphylococcus epidermidis*	1	32		2	4	32	4	2	≤0.06	16	16	32	32	4		1	0.5		>32		0.12	0.06		32
2276	*Staphylococcus epidermidis*	0.5	2		0.25	0.5	2	1	2	≤0.06	4	2	4	8	0.25		0.06	0.5		>32		8	0.25		2
2277	*Staphylococcus epidermidis*	0.5	4		0.5	0.5	2	1	2	≤0.06	2	4	8	8	0.5		0.25	0.5		0.06		>64	0.25		0.25
2278	*Staphylococcus epidermidis*	0.25	2		0.12	0.12	0.5	0.06	2	≤0.06	2	0.25	1	4	0.25		0.06	1		8		8	0.25		0.25
2279	*Staphylococcus epidermidis*	0.5	1		0.06	0.12	1	0.06	2	≤0.06	4	0.25	0.5	4	0.25		0.06	1		0.12		2	0.25		0.25
2280	*Staphylococcus epidermidis*	0.5	0.5		0.06	0.25	0.25	0.06	2	≤0.06	4	0.5	2	4	0.25		0.06	0.5		0.06		1	0.25		0.06
2281	*Staphylococcus epidermidis*	0.5	16		1	4	8	8	2	≤0.06	4	16	32	32	>8		4	1		>32		1	0.12		0.25
2282	*Staphylococcus epidermidis*	1	4		0.5	0.5	2	2	2	≤0.06	8	4	4	16	0.25		0.06	1		>32		2	0.25		0.25
2283	*Staphylococcus epidermidis*	0.5	4		0.25	0.5	2	1	1	0.12	1	4	8	8	1		0.25	1		0.06		>64	0.25		0.25
2284	*Staphylococcus epidermidis*	0.5	1		0.06	0.12	0.5	0.06	1	≤0.06	1	0.25	1	4	0.25		0.06	1		8		1	0.25		0.25
2285	*Staphylococcus epidermidis*	0.5	4		0.5	1	4	1	2	≤0.06	4	4	8	16	0.25		0.06	2		>32		8	0.25		2
2286	*Staphylococcus epidermidis*	0.25	0.5		0.06	0.12	0.5	0.03	2	≤0.06	4	0.25	1	4	0.25		0.06	1		0.12		2	0.25		0.25
2287	*Staphylococcus epidermidis*	0.5	1		0.06	0.12	0.25	0.03	2	0.12	4	0.25	1	4	0.25		0.06	1		0.06		1	0.25		0.06
2288	*Staphylococcus epidermidis*	1	32		2	16	16	16	2	0.12	4	32	32	32	>8		4	1		>32		2	0.25		0.25
2289	*Staphylococcus haemolyticus*	0.5	0.5		0.12	1	0.5	0.12	1	0.12	2	2	8	16	0.12		0.06	1		0.12		0.25	0.12		0.25
2290	*Staphylococcus haemolyticus*	0.5	8		2	16	8	2	1	≤0.06	8	8	32	>64	0.12		0.06	1		0.12		0.25	0.12		0.25
2291	*Staphylococcus haemolyticus*	0.5	0.06		0.25	1	0.12	0.25	0.25	≤0.06	0.5	2	8	16	0.06		0.03	1		0.12		0.12	0.12		0.12
2292	*Staphylococcus haemolyticus*	0.5	4		1	4	4	1	1	≤0.06	4	8	32	32	0.12		0.06	1		>32		0.25	0.12		0.5
2293	*Staphylococcus haemolyticus*	1	0.25		0.25	1	0.5	0.12	0.5	0.25	4	1	8	16	0.12		0.06	1		0.12		0.25	0.25		0.12
2294	*Staphylococcus haemolyticus*	0.5	1		0.25	1	0.5	0.25	1	0.12	4	2	8	16	0.25		0.06	1		32		0.25	0.25		0.25
2295	*Staphylococcus haemolyticus*	0.5	1		0.5	2	1	0.25	1	≤0.06	4	8	8	32	0.12		0.06	1		32		32	0.25		0.25
2296	*Staphylococcus haemolyticus*	1	0.06		0.12	1	0.12	0.25	0.5	≤0.06	2	2	8	32	0.12		0.06	1		8		32	0.25		0.25
2297	*Staphylococcus haemolyticus*	0.25	>64		16	64	>32	16	1	≤0.06	4	>64	>32	>64	>8		8	1		0.12		1	0.25		32
2298	*Staphylococcus haemolyticus*	0.5	>64		32	>64	>32	>16	2	0.25	4	>64	>32	>64	4		1	1		>32		1	0.25		32
2299	*Staphylococcus haemolyticus*	0.5	>64		32	>64	>32	>16	2	0.25	4	>64	>32	>64	4		1	1		32		0.5	0.12		32
2300	*Staphylococcus haemolyticus*	0.5	>64		32	>64	>32	>16	1	0.12	2	>64	>32	>64	4		1	1		16		0.5	0.12		1
2301	*Staphylococcus haemolyticus*	0.5	>64		32	>64	>32	>16	1	≤0.06	4	>64	>32	>64	4		1	1		>32		1	0.25		16
2302	*Staphylococcus haemolyticus*	0.5	>64		32	>64	>32	>16	2	0.25	2	>64	>32	>64	8		2	1		32		1	0.25		16
2303	*Staphylococcus haemolyticus*	0.5	>64		32	>64	>32	>16	2	≤0.06	8	>64	>32	>64	8		2	1		>32		1	0.25		32
2304	*Staphylococcus haemolyticus*	0.5	>64		32	>64	>32	>16	1	0.25	8	>64	>32	>64	4		1	1		16		1	0.25		0.5
2305	*Staphylococcus haemolyticus*	1	>64		>32	>64	>32	>16	1	≤0.06	2	>64	>32	>64	4		1	1		16		32	0.25		0.5
2306	*Staphylococcus haemolyticus*	0.5	0.03		0.12	0.5	0.06	0.12	1	≤0.06	2	1	8	8	0.12		0.06	1		32		0.12	0.06		0.12
2307	*Staphylococcus haemolyticus*	1	64		8	16	32	16	1	≤0.06	2	64	>32	>64	>8		4	1		32		0.12	0.06		32
2308	*Staphylococcus haemolyticus*	1	8		2	8	4	1	1	≤0.06	2	8	32	32	0.12		0.03	1		0.12		0.25	0.12		0.25
2309	*Staphylococcus haemolyticus*	0.5	16		1	2	4	0.25	1	≤0.06	4	2	8	16	0.12		0.06	1		>32		64	0.25		0.25
2310	*Staphylococcus haemolyticus*	0.25	1		0.25	1	0.5	0.12	0.5	≤0.06	2	2	8	8	0.25		0.06	2		>32		0.25	0.25		0.5
2311	*Staphylococcus haemolyticus*	0.5	64		32	>64	>32	>16	1	0.12	32	>64	>32	>64	>8		4	2		>32		>64	2		>32
2312	*Staphylococcus haemolyticus*	0.25	>64		>32	>64	>32	>16	1	≤0.06	2	>64	>32	>64	>8		4	0.5		>32		0.5	0.12		>32
2313	*Staphylococcus haemolyticus*	0.5	64		4	16	32	4	1	≤0.06	8	32	>32	>64	>8		4	1		>32		4	0.5		>32

Isolate Number	*Staphylococcus* species	MIC (µg/ml) Values	β Lactams					Carbapenem	Glycopeptides			Cephalosporins			Flouroquinolones			Oxazolidnone	Macrolides			Tetracyclines		Aminoglycoside	Antifolate
		XF-73	Penicillin	Oxacillin	Amoxicillin Clavulanate	Piperacillin/Tazobactam	Ampicillin	Meropenem	Vancomycin	Dalbavancin	Teicoplanin	Cefuroxime	Ceftriaxone	Ceftazidime	Levofloxacin	Ciprofloxacin	Moxifloxacin	Linezolid	Clindamycin	Clarithromycin	Erythromycin	Tetracycline	Tigecycline	Gentamicin	Trimethoprim/ Sulfmethoxazole
2314	*Staphylococcus haemolyticus*	0.5	>64		>32	>64	>32	>16	1	≤0.06	2	>64	>32	>64	>8		4	0.5		>32		1	0.25		1
2315	*Staphylococcus haemolyticus*	0.5	>64		32	>64	>32	>16	2	0.25	8	>64	>32	>64	8		1	1		16		1	0.25		1
2316	*Staphylococcus haemolyticus*	1	64		32	>64	32	>16	1	0.25	4	>64	>32	>64	>8		4	1		>32		0.5	0.12		32
2317	*Staphylococcus haemolyticus*	0.5	>64		32	>64	>32	>16	1	≤0.06	1	>64	>32	>64	4		1	0.5		>32		0.5	0.12		32
2318	*Staphylococcus haemolyticus*	0.5	>64		>32	>64	>32	>16	1	0.12	2	>64	>32	>64	8		1	1		>32		0.5	0.12		0.5
2319	*Staphylococcus haemolyticus*	0.5	≤0.03		0.12	0.25	0.06	0.12	2	0.25	4	1	4	8	>8		4	1		>32		0.12	0.12		16
2320	*Staphylococcus haemolyticus*	0.5	0.03		0.12	0.5	0.06	0.12	0.5	≤0.06	4	1	4	8	0.12		0.06	1		32		0.12	0.12		0.25
2321	*Staphylococcus haemolyticus*	0.5	0.5		0.5	1	1	0.25	1	≤0.06	2	2	8	16	0.25		0.06	1		0.12		32	0.12		0.25
2322	*Staphylococcus haemolyticus*	1	1		0.25	1	0.5	0.12	0.5	≤0.06	2	2	8	16	0.12		0.06	0.5		0.06		1	0.5		0.5
2323	*Staphylococcus haemolyticus*	1	64		8	64	32	16	1	0.25	4	>64	>32	>64	>8		2	1		8		4	0.5		32
2324	*Staphylococcus haemolyticus*	0.5	2		0.5	0.5	2	0.25	1	≤0.06	2	2	8	16	0.12		0.06	1		>32		0.25	0.25		0.25
2325	*Staphylococcus haemolyticus*	0.5	2		0.5	1	2	0.5	4	0.12	16	2	16	32	0.12		0.06	1		32		64	1		0.12
2326	*Staphylococcus haemolyticus*	0.5	64		32	>64	>32	>16	1	≤0.06	2	>64	>32	>64	8		1	1		>32		64	0.12		2
2327	*Staphylococcus haemolyticus*	1	>64		32	>64	>32	>16	2	≤0.06	4	>64	>32	>64	>8		4	1		8		0.5	0.5		>32
2328	*Staphylococcus haemolyticus*	2	32		16	64	16	16	2	0.12	4	>64	>32	>64	>8		4	1		>32		0.12	0.06		16
2329	*Staphylococcus haemolyticus*	0.5	64		8	64	32	8	1	≤0.06	4	>64	>32	>64	>8		4	1		16		4	0.25		32
2330	*Staphylococcus haemolyticus*	0.25	64		4	16	32	4	1	0.12	4	64	>32	>64	>8		2	1		>32		4	0.5		>32
2331	*Staphylococcus haemolyticus*	0.5	64		8	64	32	8	1	0.25	4	>64	>32	>64	>8		4	1		32		4	0.25		>32
2332	*Staphylococcus haemolyticus*	0.5	>64		16	>64	>32	16	1	0.12	4	>64	>32	>64	>8		2	1		16		4	2		>32
2333	*Staphylococcus haemolyticus*	0.25	64		32	>64	32	>16	1	0.12	4	>64	>32	>64	8		2	1		16		1	0.12		1
2334	*Staphylococcus haemolyticus*	0.5	>64		32	>64	>32	>16	2	0.25	16	>64	>32	>64	0.5		0.12	2		16		1	0.25		0.5
2335	*Staphylococcus haemolyticus*	0.5	0.12		0.25	2	0.25	0.5	1	≤0.06	2	4	16	64	0.12		0.03	1		16		64	1		>32
2336	*Staphylococcus haemolyticus*	0.5	16		8	8	4	4	1	0.12	4	64	>32	64	0.12		0.06	1		>32		8	0.12		1
2337	*Staphylococcus haemolyticus*	0.5	64		4	32	32	4	2	0.25	4	64	>32	64	8		2	1		16		32	1		>32
2338	*Staphylococcus haemolyticus*	1	>64		32	>64	>32	>16	1	0.12	4	>64	>32	>64	>8		8	1		16		1	0.25		>32
2339	*Staphylococcus haemolyticus*	1	64		16	>64	>32	>16	1	0.5	4	>64	>32	>64	>8		4	1		>32		1	0.25		>32
2340	*Staphylococcus haemolyticus*	0.5	32		2	8	16	2	0.5	≤0.06	2	16	32	64	4		1	8		>32		0.25	0.12		0.12
2341	*Staphylococcus haemolyticus*	0.5	0.12		0.12	0.5	0.12	0.015	1	0.25	2	2	0.25	0.12	4		1	1		16		2	0.5		2
2342	*Staphylococcus haemolyticus*	0.5	>64		>32	>64	>32	>16	2	0.12	2	>64	>32	>64	>8		8	1		16		1	0.25		0.5
2343	*Staphylococcus haemolyticus*	0.5	64		16	>64	>32	>16	1	0.5	4	>64	>32	>64	8		1	1		16		1	0.25		32
2344	*Staphylococcus haemolyticus*	0.5	0.03		0.12	0.5	0.06	0.12	1	0.12	2	1	8	8	0.12		0.06	1		32		0.25	0.12		0.12
2345	*Staphylococcus haemolyticus*	0.5	0.03		0.12	0.5	0.06	0.06	0.5	0.12	16	1	4	16	0.12		0.06	1		32		0.25	0.25		0.06
2346	*Staphylococcus haemolyticus*	0.5	16		2	8	16	1	1	≤0.06	0.5	16	32	64	8		2	2		0.25		0.25	0.25		0.5
2347	*Staphylococcus haemolyticus*	0.5	0.25		0.25	0.5	0.25	0.12	2	≤0.06	2	1	4	8	0.12		0.06	1		32		1	0.06		0.5
2348	*Staphylococcus haemolyticus*	0.25	16		1	4	8	1	1	≤0.06	2	8	32	32	0.12		0.03	2		>32		32	0.12		0.25
2349	*Staphylococcus haemolyticus*	1	64		8	32	32	8	1	0.25	4	64	>32	>64	>8		4	1		16		4	0.25		>32
2350	*Staphylococcus haemolyticus*	0.5	32		1	4	8	1	1	0.25	2	4	16	16	0.12		0.06	1		0.12		0.12	0.12		0.25
2351	*Staphylococcus haemolyticus*	0.5	≤0.03		0.06	0.25	0.06	0.06	0.5	0.5	2	1	4	8	0.06		0.03	1		0.12		0.12	0.12		0.12
2352	*Staphylococcus haemolyticus*	1	>64		32	>64	>32	>16	1	0.12	4	>64	>32	>64	>8		8	1		>32		2	0.25		16
2353	*Staphylococcus haemolyticus*	0.25	≤0.03		0.06	0.5	0.06	0.06	1	0.25	2	1	4	8	0.12		0.06	1		16		0.25	0.12		0.25
2354	*Staphylococcus haemolyticus*	0.5	64		16	>64	32	>16	2	0.25	8	>64	>32	>64	8		1	1		16		1	0.25		>32
2355	*Staphylococcus haemolyticus*	4	64		8	64	32	>16	2	0.25	8	>64	>32	>64	0.12		0.06	1		16		1	0.5		0.5
2356	*Staphylococcus haemolyticus*	0.5	>64		16	>64	>32	>16	1	0.12	4	>64	>32	>64	4		1	0.5		>32		1	0.12		1
2357	*Staphylococcus haemolyticus*	1	1		0.12	1	0.5	0.12	0.5	0.12	2	1	4	8	0.25		0.06	0.5		0.06		0.12	0.12		0.25
2358	*Staphylococcus haemolyticus*	0.25	>64		16	>64	>32	>16	1	0.12	4	>64	>32	>64	0.12		0.06	1		16		1	0.25		1
2359	*Staphylococcus haemolyticus*	0.5	32		2	8	16	4	2	0.12	4	16	32	64	1		0.25	1		32		32	0.12		0.25
2360	*Staphylococcus haemolyticus*	0.5	>64		>32	>64	>32	>16	2	≤0.06	4	>64	>32	>64	4		1	1		16		1	0.25		2
2361	*Staphylococcus haemolyticus*	0.5	2		0.25	0.5	0.5	0.12	2	0.12	4	2	8	16	0.25		0.06	2		32		0.25	0.12		0.25
2362	*Staphylococcus haemolyticus*	1	>64		>32	>64	>32	>16	2	0.12	4	>64	>32	>64	>8		2	1		8		1	0.25		1
2363	*Staphylococcus haemolyticus*	0.5	>64		32	>64	>32	>16	4	0.25	8	>64	>32	>64	>8		4	1		16		>64	0.5		>32

Isolate Number	*Staphylococcus* species	MIC (µg/ml) Values																							
			β Lactams					Carbapenem	Glycopeptides			Cephalosporins			Flouroquinolones			Oxazolidnone	Macrolides			Tetracyclines		Aminoglycoside	Antifolate
		XF-73	Penicillin	Oxacillin	Amoxicillin Clavulanate	Piperacillin/Tazobactam	Ampicillin	Meropenem	Vancomycin	Dalbavancin	Teicoplanin	Cefuroxime	Ceftriaxone	Ceftazidime	Levofloxacin	Ciprofloxacin	Moxifloxacin	Linezolid	Clindamycin	Clarithromycin	Erythromycin	Tetracycline	Tigecycline	Gentamicin	Trimethoprim/ Sulfmethoxazole
2364	*Staphylococcus haemolyticus*	0.5	64		32	>64	>32	>16	2	0.25	4	>64	>32	>64	8		2	1		>32		1	0.25		>32
2365	*Staphylococcus haemolyticus*	0.25	1		0.12	1	1	0.25	1	0.12	4	1	4	8	0.12		0.06	1		8		0.25	0.12		0.5
2366	*Staphylococcus haemolyticus*	0.5	>64		16	>64	>32	>16	1	0.12	2	>64	>32	>64	4		1	1		>32		0.5	0.12		0.5
2367	*Staphylococcus haemolyticus*	0.5	>64		16	>64	>32	>16	2	0.5	4	>64	>32	>64	>8		4	1		>32		>64	0.5		>32
2368	*Staphylococcus haemolyticus*	0.5	>64		32	>64	>32	>16	2	0.25	4	>64	>32	>64	>8		4	1		>32		>64	0.5		>32
2369	*Staphylococcus haemolyticus*	0.5	32		1	4	16	1	2	0.12	8	8	32	64	0.12		0.12	1		>32		0.12	0.06		0.5
2370	*Staphylococcus haemolyticus*	0.5	32		4	16	32	8	2	≤0.06	8	16	32	64	>8		4	1		>32		4	0.25		>32
2371	*Staphylococcus haemolyticus*	0.5	>64		32	>64	>32	>16	2	≤0.06	4	>64	>32	>64	8		2	1		>32		1	0.25		1
2372	*Staphylococcus haemolyticus*	0.5	>64		>32	>64	>32	>16	2	≤0.06	4	>64	>32	>64	>8		8	2		16		2	0.5		>32
2373	*Staphylococcus haemolyticus*	1	32		4	32	8	0.25	1	0.25	2	1	32	64	2		0.5	1		>32		1	0.25		0.5
2374	*Staphylococcus haemolyticus*	0.5	>64		>32	>64	>32	>16	2	≤0.06	4	>64	>32	>64	>8		4	1		>32		2	0.5		>32
2375	*Staphylococcus haemolyticus*	1	>64		32	>64	>32	>16	2	0.25	8	>64	>32	>64	8		4	1		>32		>64	0.25		>32
2376	*Staphylococcus haemolyticus*	0.5	>64		>32	>64	>32	>16	1	≤0.06	4	>64	>32	>64	8		1	1		>32		>64	0.5		1
2377	*Staphylococcus haemolyticus*	0.5	>64		>32	>64	>32	>16	1	0.25	8	>64	>32	>64	8		1	1		>32		>64	0.25		1
2378	*Staphylococcus haemolyticus*	0.25	0.12		0.25	0.5	0.25	0.12	1	≤0.06	1	0.5	4	8	0.12		0.06	1		16		64	0.12		0.25
2379	*Staphylococcus haemolyticus*	0.5	2		0.25	1	1	0.25	1	0.12	2	1	8	16	0.12		0.06	1		32		64	0.12		0.25
2380	*Staphylococcus haemolyticus*	0.5	≤0.03		0.06	0.5	0.06	0.25	1	0.12	1	1	8	16	0.12		0.06	1		>32		0.25	0.12		0.12
2381	*Staphylococcus haemolyticus*	0.5		> 4					1	0.5	4					0.25		1	≤ 0.5		0.25		0.06	≤ 0.06	≤ 0.5
2382	*Staphylococcus hominis*	0.5	4		2	8	4	8	0.5	≤0.06	0.25	2	16	64	8		2	1		>32		0.25	0.12		8
2383	*Staphylococcus hominis*	0.25	0.25		0.12	0.12	0.25	0.12	1	0.25	0.5	0.5	2	8	0.12		0.03	1		0.25		0.25	0.12		0.5
2384	*Staphylococcus hominis*	0.5	0.06		0.06	0.25	0.06	0.12	1	0.25	0.5	0.5	2	8	1		0.25	1		0.25		0.25	0.06		0.12
2385	*Staphylococcus hominis*	0.5	0.25		0.25	1	0.5	0.5	1	≤0.06	0.5	0.5	4	16	0.12		0.03	1		32		0.25	0.12		0.5
2386	*Staphylococcus hominis*	0.5	≤0.03		0.03	≤0.03	0.03	0.015	1	≤0.06	0.5	0.25	2	8	0.12		0.06	1		0.12		0.25	0.12		0.5
2387	*Staphylococcus hominis*	0.25	0.12		0.12	0.5	0.25	0.12	1	0.25	0.25	0.5	4	8	0.12		0.03	1		32		0.25	0.06		0.06
2388	*Staphylococcus hominis*	0.5	0.5		1	4	2	1	1	0.12	0.5	2	16	64	>8		>8	1		>32		64	0.12		4
2389	*Staphylococcus hominis*	0.25	8		1	4	4	4	1	≤0.06	0.25	2	16	64	0.12		0.06	1		32		64	0.12		4
2390	*Staphylococcus hominis*	0.25	0.12		0.06	0.25	0.25	0.06	0.5	≤0.06	0.25	0.25	2	4	0.12		0.06	1		0.06		1	0.25		0.06
2391	*Staphylococcus hominis*	0.5	2		2	4	4	4	2	≤0.06	4	2	16	64	0.12		0.06	1		>32		0.25	0.12		8
2392	*Staphylococcus hominis*	0.25	4		0.5	1	2	0.5	1	≤0.06	0.25	0.5	4	16	0.12		0.03	1		32		64	0.12		8
2393	*Staphylococcus hominis*	0.5	8		1	2	4	1	1	≤0.06	2	16	16	32	0.12		0.06	1		32		0.25	0.25		0.25
2394	*Staphylococcus hominis*	0.25	16		2	16	16	16	1	≤0.06	0.25	2	>32	>64	8		2	1		32		64	0.5		4
2395	*Staphylococcus hominis*	0.25	16		16	64	16	>16	0.5	≤0.06	0.25	64	>32	>64	4		1	2		4		1	0.12		16
2396	*Staphylococcus hominis*	0.5	8		8	16	8	16	1	0.25	0.5	16	>32	64	0.5		0.12	0.5		>32		0.12	0.03		0.06
2397	*Staphylococcus hominis*	0.5	1		1	2	1	1	1	≤0.06	0.5	1	8	16	0.25		0.06	1		>32		32	0.12		8
2398	*Staphylococcus hominis*	0.5	16		4	8	16	4	1	≤0.06	0.5	2	32	>64	2		1	2		0.25		64	0.12		4
2399	*Staphylococcus hominis*	0.25	0.12		0.25	0.5	0.5	0.5	0.5	≤0.06	0.25	0.5	8	16	0.008		0.03	1		32		0.25	0.12		4
2400	*Staphylococcus hominis*	0.25	8		4	8	16	8	2	0.12	4	4	32	>64	2		0.5	2		>32		>64	0.25		16
2401	*Staphylococcus hominis*	0.25	0.12		0.06	0.06	0.03	0.03	0.5	0.12	0.5	0.12	0.5	4	0.12		0.03	1		16		0.25	0.12		0.06
2402	*Staphylococcus hominis*	0.5	1		0.5	2	1	1	1	≤0.06	2	0.5	8	16	0.12		0.03	1		16		1	0.12		0.5
2403	*Staphylococcus hominis*	0.25	2		2	8	2	2	1	≤0.06	0.5	2	16	>64	>8		8	2		32		0.5	0.12		8
2404	*Staphylococcus hominis*	0.5	4		2	4	4	4	1	≤0.06	4	8	16	64	>8		2	1		>32		64	0.12		8
2405	*Staphylococcus hominis*	0.5	1		1	4	2	2	1	≤0.06	4	2	16	64	>8		>8	1		>32		64	0.12		4
2406	*Staphylococcus hominis*	0.5	2		1	4	2	2	1	≤0.06	4	8	16	>64	>8		2	1		>32		64	0.12		4
2407	*Staphylococcus hominis*	0.5	0.25		0.25	0.5	0.25	0.12	0.5	≤0.06	0.25	0.5	2	8	0.12		0.06	1		0.12		64	0.25		0.06
2408	*Staphylococcus hominis*	0.5	0.25		0.12	0.5	0.25	0.06	0.25	≤0.06	0.25	32	32	2	0.12		0.03	1		>32		0.5	0.12		0.06
2409	*Staphylococcus hominis*	0.5	0.25		1	1	0.5	1	0.5	≤0.06	0.25	2	16	64	>8		2	1		>32		64	0.12		4
2410	*Staphylococcus hominis*	0.5	0.25		0.5	2	0.5	1	0.5	≤0.06	0.25	1	8	32	8		2	0.5		0.06		0.12	0.12		4
2411	*Staphylococcus hominis*	0.5	≤0.03		0.03	0.06	0.03	0.03	0.5	≤0.06	0.25	0.12	1	4	0.06		0.03	1		>32		8	0.06		0.06
2412	*Staphylococcus hominis*	0.5	16		2	8	8	8	1	≤0.06	0.5	2	16	>64	0.5		0.12	0.5		>32		1	0.25		4
2413	*Staphylococcus hominis*	0.25	1		1	4	2	2	0.5	≤0.06	0.25	2	16	64	>8		4	1		>32		0.25	0.12		4

Isolate Number	***Staphylococcus* species**	**MIC (µg/ml) Values**																							
			**β Lactams**					**Carbapenem**	**Glycopeptides**			**Cephalosporins**			**Flouroquinolones**			**Oxazolidnone**	**Macrolides**			**Tetracyclines**		**Aminoglycoside**	**Antifolate**
		**XF-73**	**Penicillin**	**Oxacillin**	**Amoxicillin Clavulanate**	**Piperacillin/Tazobactam**	**Ampicillin**	**Meropenem**	**Vancomycin**	**Dalbavancin**	**Teicoplanin**	**Cefuroxime**	**Ceftriaxone**	**Ceftazidime**	**Levofloxacin**	**Ciprofloxacin**	**Moxifloxacin**	**Linezolid**	**Clindamycin**	**Clarithromycin**	**Erythromycin**	**Tetracycline**	**Tigecycline**	**Gentamicin**	**Trimethoprim/ Sulfmethoxazole**
2414	*Staphylococcus hominis*	0.5	0.5		1	2	2	1	1	≤0.06	0.5	2	16	64	>8		4	2		>32		0.25	0.12		4
2415	*Staphylococcus hominis*	0.25	8		1	4	8	2	0.5	≤0.06	0.25	2	8	64	0.5		0.12	1		>32		64	0.5		4
2416	*Staphylococcus hominis*	0.5	1		0.5	2	1	1	1	≤0.06	0.5	0.5	8	16	0.5		0.12	1		0.06		64	0.12		0.06
2417	*Staphylococcus hominis*	0.25	2		2	8	4	4	1	≤0.06	0.5	2	16	16	8		2	1		0.12		64	0.25		8
2418	*Staphylococcus hominis*	1	32		2	8	16	4	1	0.12	0.25	2	16	64	8		1	1		0.12		64	0.12		4
2419	*Staphylococcus hominis*	0.25	1		2	4	4	8	1	≤0.06	0.5	2	32	>64	>8		>8	2		0.12		0.25	0.12		8
2420	*Staphylococcus hominis*	0.25	0.25		0.12	0.25	0.25	0.12	1	≤0.06	0.5	0.5	2	8	0.06		0.015	1		32		0.25	0.06		0.12
2421	*Staphylococcus hominis*	0.25	1		1	2	1	1	1	≤0.06	0.25	1	8	32	0.12		0.03	1		16		0.25	0.06		4
2422	*Staphylococcus hominis*	2	0.25		0.5	2	1	0.5	1	≤0.06	0.25	2	16	64	>8		>8	2		8		64	0.25		8
2423	*Staphylococcus hominis*	0.5	2		1	8	2	4	1	0.12	0.25	1	32	64	8		2	2		>32		2	0.5		16
2424	*Staphylococcus hominis*	0.25	0.25		0.12	0.12	0.25	0.06	0.25	≤0.06	0.5	0.25	1	4	0.12		0.06	1		16		0.5	0.06		0.06
2425	*Staphylococcus hominis*	0.25	0.5		0.5	2	1	1	1	≤0.06	0.5	2	8	64	>8		>8	2		>32		64	0.12		4
2426	*Staphylococcus hominis*	0.25	0.5		0.25	1	0.5	0.25	1	≤0.06	0.5	0.5	4	8	0.12		0.03	1		0.12		2	0.25		0.25
2427	*Staphylococcus hominis*	0.25	≤0.03		0.06	0.06	0.03	0.06	1	≤0.06	0.5	0.25	2	8	0.12		0.03	1		16		0.12	0.06		0.06
2428	*Staphylococcus hominis*	0.5	0.12		0.25	1	0.5	0.25	0.5	≤0.06	0.25	0.25	4	8	0.5		0.12	1		>32		0.25	0.5		0.25
2429	*Staphylococcus hominis*	0.5	8		1	4	8	4	1	≤0.06	0.5	2	16	64	8		2	0.5		>32		0.12	0.06		8
2430	*Staphylococcus hominis*	0.5	0.25		0.25	0.5	0.5	0.25	1	≤0.06	0.5	0.5	4	8	0.12		0.06	1		>32		16	0.06		0.06
2431	*Staphylococcus hominis*	0.5	0.12		0.12	0.12	0.25	0.06	1	≤0.06	0.5	0.5	2	8	0.12		0.03	1		32		0.12	0.03		0.03
2432	*Staphylococcus hominis*	0.25	0.5		0.25	1	0.5	0.25	1	≤0.06	0.5	1	4	16	0.12		0.06	1		>32		0.25	0.06		0.5
2433	*Staphylococcus hominis*	0.5	64		2	8	16	4	1	≤0.06	0.5	4	16	>64	8		2	1		>32		0.25	0.06		4
2434	*Staphylococcus hominis*	0.5	0.12		0.06	0.25	0.06	0.12	1	≤0.06	0.5	0.5	2	8	0.06		0.03	1		0.06		0.25	0.03		0.06
2435	*Staphylococcus hominis*	0.25	0.12		0.12	0.5	0.12	0.12	0.5	≤0.06	0.5	0.5	2	8	0.12		0.06	1		16		0.25	0.06		0.25
2436	*Staphylococcus hominis*	1	0.25		0.25	0.5	0.5	0.12	2	0.5	0.5	1	4	16	0.12		0.03	1		0.12		16	0.12		0.5
2437	*Staphylococcus hominis*	0.25	64		4	64	32	16	1	0.12	0.5	>64	>32	>64	0.12		0.06	2		0.12		0.25	0.12		0.5
2438	*Staphylococcus hominis*	1	0.25		0.25	0.5	0.25	0.12	1	0.12	0.5	0.5	8	8	0.12		0.06	1		0.12		0.25	0.06		0.25
2439	*Staphylococcus hominis*	0.25	4		2	8	4	4	1	≤0.06	2	4	16	>64	0.12		0.03	1		16		64	0.12		0.25
2440	*Staphylococcus hominis*	0.25	0.25		0.12	0.5	0.25	0.12	1	0.12	0.5	0.5	4	16	0.12		0.06	1		0.12		>64	0.25		0.25
2441	*Staphylococcus hominis*	1	0.5		0.25	1	0.5	0.5	1	≤0.06	0.25	0.5	8	16	0.06		0.03	1		16		0.25	0.06		0.06
2442	*Staphylococcus hominis*	0.5	0.5		0.25	1	0.5	0.5	1	≤0.06	0.25	0.5	4	8	0.12		0.03	1		16		0.25	0.06		0.06
2443	*Staphylococcus hominis*	0.5	≤0.03		0.03	0.06	0.06	0.06	0.5	≤0.06	0.5	0.25	2	4	0.12		0.03	1		0.12		0.25	0.06		0.12
2444	*Staphylococcus hominis*	0.5	≤0.03		0.03	≤0.03	0.06	0.06	1	≤0.06	0.5	0.5	2	8	0.12		0.03	1		0.06		0.12	0.06		0.03
2445	*Staphylococcus hominis*	0.25	4		2	8	4	2	1	0.12	0.5	4	16	>64	>8		>8	1		>32		64	0.12		8
2446	*Staphylococcus hominis*	0.25	0.5		0.25	0.5	0.25	0.06	1	≤0.06	0.5	0.5	2	8	0.12		0.06	1		0.12		0.25	0.06		0.03
2447	*Staphylococcus hominis*	1	4		2	4	4	4	1	0.12	8	2	16	64	0.5		0.12	1		>32		0.5	0.12		0.5
2448	*Staphylococcus hominis*	0.5	0.25		0.25	0.5	0.25	0.03	1	≤0.06	8	0.5	2	8	0.12		0.03	1		>32		64	0.06		4
2449	*Staphylococcus hominis*	0.25	4		0.5	0.5	2	16	1	0.12	2	16	32	32	8		2	1		>32		0.25	0.06		2
2450	*Staphylococcus hominis*	0.5	0.06		0.25	0.5	0.25	0.12	1	≤0.06	1	0.5	4	8	0.12		0.03	1		8		64	0.12		0.25
2451	*Staphylococcus hominis*	0.25	1		0.25	0.5	0.5	0.25	4	≤0.06	4	0.5	4	8	0.12		0.03	4		32		0.25	0.06		4
2452	*Staphylococcus intermedius*	0.25	32		1	2	32	0.25	1	≤0.06	0.5	0.25	16	16	8		2	1		>32		0.25	0.12		>32
2453	*Staphylococcus lugdunensis*	0.25	8		0.5	0.5	4	0.5	1	≤0.06	0.25	1	4	16	0.06		0.03	1		0.12		0.12	0.06		0.25
2454	*Staphylococcus lugdunensis*	0.25	1		0.5	1	1	0.25	1	≤0.06	0.5	1	4	16	0.12		0.06	1		0.12		0.12	0.06		0.12
2455	*Staphylococcus lugdunensis*	0.25	1		0.5	1	1	0.25	1	0.25	0.5	2	4	16	0.12		0.06	1		0.12		0.12	0.06		0.12
2456	*Staphylococcus lugdunensis*	1	0.06		0.12	0.5	0.12	0.25	1	0.25	0.5	0.5	2	16	0.12		0.06	1		0.06		0.25	0.06		0.25
2457	*Staphylococcus lugdunensis*	0.5	≤0.03		0.12	0.5	0.06	0.12	1	≤0.06	0.5	1	2	8	0.12		0.06	0.5		≤0.03		0.06	0.03		0.25
2458	*Staphylococcus lugdunensis*	0.5	0.06		0.12	0.5	0.12	0.25	1	≤0.06	0.5	1	4	16	0.06		0.06	1		>32		0.12	0.03		0.25
2459	*Staphylococcus lugdunensis*	0.5	0.06		0.25	0.5	0.12	0.5	1	≤0.06	0.5	1	4	16	0.12		0.06	0.5		0.06		0.12	0.03		0.25
2460	*Staphylococcus lugdunensis*	≤0.12	0.06		0.25	0.5	0.12	0.5	1	≤0.06	0.5	1	4	16	0.12		0.06	1		0.06		0.12	0.06		0.5
2461	*Staphylococcus lugdunensis*	0.25	0.12		0.25	0.5	0.25	0.25	1	≤0.06	0.5	1	4	16	0.25		0.06	1		0.12		0.12	0.06		0.12
2462	*Staphylococcus lugdunensis*	0.25	0.12		0.25	0.5	0.12	0.25	0.5	≤0.06	0.25	1	4	16	0.25		0.12	1		0.06		0.25	0.015		0.25
2463	*Staphylococcus lugdunensis*	1	0.06		0.25	0.5	0.12	0.25	1	≤0.06	0.25	1	4	16	0.25		0.12	0.25		≤0.03		≤0.03	0.015		0.12

Isolate Number	*Staphylococcus* species	MIC (µg/ml) Values																							
			β Lactams					Carbapenem	Glycopeptides			Cephalosporins			Flouroquinolones			Oxazolidnone	Macrolides			Tetracyclines		Aminoglycoside	Antifolate
		XF-73	Penicillin	Oxacillin	Amoxicillin Clavulanate	Piperacillin/Tazobactam	Ampicillin	Meropenem	Vancomycin	Dalbavancin	Teicoplanin	Cefuroxime	Ceftriaxone	Ceftazidime	Levofloxacin	Ciprofloxacin	Moxifloxacin	Linezolid	Clindamycin	Clarithromycin	Erythromycin	Tetracycline	Tigecycline	Gentamicin	Trimethoprim/ Sulfmethoxazole
2464	*Staphylococcus lugdunensis*	0.25	0.06		0.25	1	0.12	0.25	1	≤0.06	0.5	1	4	16	0.25		0.12	1		0.06		0.06	0.06		0.25
2465	*Staphylococcus lugdunensis*	0.5	0.06		0.25	1	0.25	0.25	0.5	0.12	0.5	2	4	16	0.25		0.12	1		0.12		0.12	0.06		0.25
2466	*Staphylococcus lugdunensis*	0.5	0.03		0.06	0.5	0.06	0.12	0.5	≤0.06	0.25	0.5	2	8	0.25		0.12	1		0.06		0.06	0.06		0.25
2467	*Staphylococcus lugdunensis*	0.5	0.12		0.25	1	0.25	0.25	0.5	≤0.06	0.5	1	4	16	0.25		0.12	0.5		0.12		0.06	0.03		0.25
2468	*Staphylococcus lugdunensis*	1	0.06		0.25	1	0.12	0.25	0.5	≤0.06	0.5	1	4	16	0.12		0.06	0.5		0.12		0.12	0.12		0.12
2469	*Staphylococcus lugdunensis*	0.5	0.12		0.25	0.5	0.12	0.12	0.5	≤0.06	0.5	0.5	2	8	0.25		0.12	1		0.06		0.12	0.12		0.06
2470	*Staphylococcus lugdunensis*	0.5	0.12		0.25	0.5	0.25	0.12	0.5	≤0.06	0.5	1	4	8	0.25		0.12	0.5		0.06		0.12	0.12		0.25
2471	*Staphylococcus lugdunensis*	1	4		0.5	1	2	0.25	0.5	≤0.06	0.5	1	4	16	0.25		0.12	1		0.06		0.12	0.06		0.5
2472	*Staphylococcus lugdunensis*	0.5	0.06		0.25	1	0.12	0.12	0.5	≤0.06	0.25	0.5	2	8	0.12		0.06	0.5		0.06		0.06	0.06		0.12
2473	*Staphylococcus lugdunensis*	1	0.12		0.12	0.5	0.12	0.25	0.5	≤0.06	0.5	1	4	8	0.25		0.12	0.5		0.06		0.12	0.06		0.25
2474	*Staphylococcus lugdunensis*	0.5	0.06		0.12	1	0.12	0.25	0.5	0.12	0.5	1	4	8	0.12		0.06	0.5		0.06		0.12	0.03		0.25
2475	*Staphylococcus lugdunensis*	0.25	0.12		0.25	1	0.25	0.25	0.5	0.12	0.25	1	4	16	0.25		0.12	0.25		0.06		0.12	0.06		0.12
2476	*Staphylococcus lugdunensis*	0.25	0.12		0.12	0.5	0.25	0.25	0.5	≤0.06	0.5	1	4	16	0.25		0.12	0.5		>32		0.12	0.06		0.25
2477	*Staphylococcus lugdunensis*	0.25	1		0.25	0.5	1	0.03	0.5	≤0.06	0.25	0.5	2	4	0.12		0.03	0.25		≤0.03		0.06	0.015		0.25
2478	*Staphylococcus lugdunensis*	1	4		0.5	2	4	0.5	0.5	0.12	0.5	1	8	16	0.25		0.12	0.5		0.06		0.12	0.06		0.25
2479	*Staphylococcus lugdunensis*	1	≤0.03		0.12	0.5	0.12	0.25	0.5	≤0.06	0.5	1	4	16	0.25		0.12	0.25		≤0.03		0.12	0.06		0.12
2480	*Staphylococcus lugdunensis*	≤0.12	32		4	64	32	16	2	0.12	4	>64	>32	>64	4		1	0.5		>32		2	0.25		8
2481	*Staphylococcus lugdunensis*	0.25	0.12		0.12	0.5	0.25	0.25	0.5	0.12	1	1	4	16	0.25		0.12	1		0.06		0.12	0.06		0.12
2482	*Staphylococcus lugdunensis*	0.25	0.06		0.12	0.5	0.25	0.25	1	≤0.06	0.5	1	8	16	0.12		0.06	1		0.12		0.12	0.06		0.06
2483	*Staphylococcus lugdunensis*	0.5	2		0.5	1	2	0.12	0.5	≤0.06	0.25	1	4	8	1		0.5	0.5		>32		0.12	0.06		0.12
2484	*Staphylococcus lugdunensis*	0.5	≤0.03		0.06	0.25	0.06	0.12	0.5	≤0.06	0.5	1	4	8	0.25		0.12	0.5		>32		64	0.06		0.5
2485	*Staphylococcus lugdunensis*	0.5	64		0.5	1	32	0.25	0.5	≤0.06	0.5	1	4	8	0.12		0.06	0.5		0.06		0.12	0.03		0.25
2486	*Staphylococcus lugdunensis*	0.5	4		0.25	0.5	2	0.25	1	≤0.06	0.5	1	4	16	0.25		0.12	0.5		0.06		0.06	0.06		0.25
2487	*Staphylococcus lugdunensis*	0.5	4		1	1	4	0.25	1	≤0.06	0.5	1	4	16	0.06		0.03	0.5		>32		0.12	0.03		0.5
2488	*Staphylococcus lugdunensis*	0.25	0.25		0.5	0.5	0.5	0.5	1	≤0.06	0.5	1	4	8	0.12		0.06	1		0.06		0.12	0.03		0.5
2489	*Staphylococcus lugdunensis*	0.25	0.5		0.5	1	1	0.25	1	≤0.06	0.5	1	4	16	0.12		0.06	1		0.06		32	0.12		0.12
2490	*Staphylococcus lugdunensis*	0.25	1		0.5	1	1	0.25	0.5	≤0.06	0.5	1	4	16	0.12		0.06	0.5		0.06		0.12	0.06		0.12
2491	*Staphylococcus lugdunensis*	0.5	4		0.5	1	4	0.25	1	0.12	0.5	2	8	16	0.25		0.12	1		0.06		0.5	0.06		0.12
2492	*Staphylococcus pasteuri*	0.5	0.06		0.12	0.5	0.06	0.12	1	≤0.06	0.5	0.5	1	4	0.25		0.06	1		0.06		0.25	0.12		≤0.015
2493	*Staphylococcus pasteuri*	0.5	1		0.5	1	1	0.12	1	≤0.06	0.5	1	4	16	0.25		0.06	1		>32		0.12	0.06		0.03
2494	*Staphylococcus pasteuri*	1	0.12		0.12	0.5	0.12	0.12	1	≤0.06	0.5	0.25	4	16	0.25		0.12	1		0.06		0.5	0.06		0.03
2495	*Staphylococcus pettenkoferi*	0.25	0.12		0.06	0.5	0.12	0.25	2	0.12	2	4	8	16	4		1	0.5		0.12		0.06	0.06		4
2496	*Staphylococcus pettenkoferi*	0.25	0.5		0.12	1	0.25	0.25	2	0.12	2	2	8	16	>8		8	0.5		2		0.25	0.12		8
2497	*Staphylococcus pettenkoferi*	0.25	0.5		0.25	1	0.25	0.25	1	0.12	2	8	16	16	>8		4	1		2		0.25	0.12		2
2498	*Staphylococcus pettenkoferi*	0.25	1		0.25	1	0.25	0.25	1	0.12	1	4	16	16	>8		4	1		>32		8	0.12		4
2499	*Staphylococcus pettenkoferi*	0.25	0.12		0.25	2	0.12	0.25	2	≤0.06	4	4	16	16	0.25		0.06	1		0.25		0.25	0.25		0.5
2500	*Staphylococcus pettenkoferi*	0.5	8		4	8	8	8	2	≤0.06	2	4	32	>64	>8		>8	1		0.12		16	0.12		4
2501	*Staphylococcus pettenkoferi*	0.25	64		16	>64	32	16	1	0.12	1	>64	>32	>64	>8		4	0.5		4		0.12	0.12		0.25
2502	*Staphylococcus pettenkoferi*	0.25	0.12		0.12	1	0.12	0.25	2	0.12	2	4	8	16	0.12		0.03	1		>32		0.25	0.12		1
2503	*Staphylococcus pettenkoferi*	0.25	32		16	>64	16	16	1	≤0.06	2	>64	>32	>64	>8		4	0.5		4		0.12	0.06		0.25
2504	*Staphylococcus pettenkoferi*	0.25	0.12		0.25	1	0.12	0.25	1	0.12	4	4	16	16	>8		8	1		0.12		0.25	0.25		0.25
2505	*Staphylococcus pettenkoferi*	0.5	2		0.25	0.5	0.5	0.25	2	≤0.06	2	4	8	32	0.25		0.06	1		>32		0.25	0.12		0.25
2506	*Staphylococcus saprophyticus*	0.25	0.25		0.5	2	0.25	0.25	2	≤0.06	4	2	16	32	0.5		0.12	4		0.25		0.5	0.25		0.06
2507	*Staphylococcus saprophyticus*	0.5	0.25		0.5	2	0.25	0.25	1	0.5	4	4	16	32	0.5		0.12	1		>32		0.5	0.25		0.12
2508	*Staphylococcus saprophyticus*	0.25	16		16	64	16	2	2	0.12	2	>64	>32	>64	0.5		0.12	2		0.25		0.5	0.12		0.06
2509	*Staphylococcus saprophyticus*	0.5	0.25		0.12	1	0.25	0.25	1	≤0.06	2	2	8	32	0.5		0.12	2		0.25		0.5	0.25		0.06
2510	*Staphylococcus sciuri*	0.25	2		4	16	2	2	2	0.25	4	64	>32	>64	0.5		0.5	2		0.25		0.5	0.12		0.25
2511	*Staphylococcus simulans*	0.25	≤0.03		0.06	0.5	0.06	0.06	0.5	≤0.06	0.25	1	4	8	0.12		0.03	2		>32		0.25	0.12		1
2512	*Staphylococcus simulans*	0.5	≤0.03		0.06	0.12	0.06	0.03	0.5	≤0.06	0.25	≤0.03	0.06	0.25	0.5		0.06	0.25		0.5		32	0.008		0.12
2513	*Staphylococcus simulans*	0.5	≤0.03		0.03	0.25	0.03	0.03	0.5	≤0.06	0.25	0.5	2	4	0.12		0.03	2		0.25		0.25	0.25		0.5

Table s2: MIC Data for the Staphylococcus species tested

Isolate Number	*Staphylococcus* species	MIC (µg/ml) Values																							
			β Lactams					Carbapenem	Glycopeptides			Cephalosporins			Flouroquinolones			Oxazolidnone	Macrolides			Tetracyclines		Aminoglycoside	Antifolate
		XF-73	Penicillin	Oxacillin	Amoxicillin Clavulanate	Piperacillin/Tazobactam	Ampicillin	Meropenem	Vancomycin	Dalbavancin	Teicoplanin	Cefuroxime	Ceftriaxone	Ceftazidime	Levofloxacin	Ciprofloxacin	Moxifloxacin	Linezolid	Clindamycin	Clarithromycin	Erythromycin	Tetracycline	Tigecycline	Gentamicin	Trimethoprim/ Sulfmethoxazole
2514	*Staphylococcus simulans*	0.5	4		0.12	0.5	0.5	0.06	1	≤0.06	1	1	4	8	0.12		0.03	2		0.12		1	0.12		0.12
2515	*Staphylococcus simulans*	0.5	0.25		0.25	0.5	0.25	0.12	1	≤0.06	0.5	2	8	8	0.12		0.03	2		0.12		0.25	0.12		0.5
2516	*Staphylococcus simulans*	0.5	32		0.5	1	16	0.5	1	0.12	0.25	8	8	32	4		1	2		0.12		0.25	0.12		0.25
2517	*Staphylococcus warneri*	0.5	0.12		0.25	0.5	0.25	0.12	4	≤0.06	0.5	0.25	1	4	0.25		0.06	1		16		0.25	0.12		0.03
2518	*Staphylococcus warneri*	0.25	8		2	16	8	1	1	≤0.06	1	64	>32	16	0.25		0.06	1		>32		0.25	0.12		0.03
2519	*Staphylococcus warneri*	0.5	4		0.5	1	2	0.12	1	≤0.06	0.5	1	2	8	0.25		0.12	2		>32		0.25	0.12		0.06
2520	*Staphylococcus warneri*	2	1		0.25	0.25	0.5	0.12	1	≤0.06	1	0.25	2	4	0.12		0.06	1		0.12		0.25	0.06		0.03
2521	*Staphylococcus warneri*	0.25	0.5		0.25	0.5	0.5	0.12	1	≤0.06	1	0.5	2	8	0.12		0.03	1		0.12		0.25	0.12		0.03
2522	*Staphylococcus warneri*	0.25	1		0.25	0.5	1	0.12	2	≤0.06	2	0.5	2	8	0.25		0.06	2		0.25		0.5	0.12		0.03
2523	*Staphylococcus warneri*	0.5	2		0.5	0.5	1	0.12	0.5	≤0.06	0.25	0.5	2	8	0.12		0.03	1		0.06		32	0.5		0.03
2524	*Staphylococcus warneri*	0.5	0.06		0.25	0.5	0.12	0.12	2	0.25	1	0.5	2	16	0.25		0.06	1		0.12		0.25	0.12		0.06
2525	*Staphylococcus warneri*	0.5	8		2	16	4	0.5	1	0.12	2	16	32	32	0.25		0.06	1		>32		0.25	0.12		0.06
2526	*Staphylococcus warneri*	0.5	1		0.5	1	1	0.12	1	0.12	1	0.5	2	16	0.25		0.06	1		0.12		0.25	0.12		≤0.015
2527	*Staphylococcus warneri*	0.5	32		8	>64	32	8	1	≤0.06	0.5	>64	>32	>64	0.25		0.06	2		>32		1	0.25		1
